# Supplementary material for: An Efficient Approach to 2-CF3-Indoles Based on ortho-Nitrobenzaldehydes
Source: Molecules. 2021 Dec 4;26(23):7365. doi: 10.3390/molecules26237365 (PMC8658784; doi:10.3390/molecules26237365)
Supplement: Supplementary file 1 [file molecules-26-07365-s001.zip › molecules-1485260-supplementary.pdf]

Supporting Information

An efficient approach to 2-CF_3-indoles based on *ortho*-nitrobenzaldehydes

Vasiliy M. Muzalevskiy,[1] Zoia A. Sizova,[1] Vladimir T. Abaev[2,3] and Valentine G. Nenajdenko[1,*]

1 Department of Chemistry, Lomonosov Moscow State University, 119899 Moscow, Russia.; muzvas@mail.ru (V.M.M); syzova@mail.ru (Z.A.S.)

2 North Ossetian State University, 44-46 Vatutina St., Vladikavkaz, 362025, Russia; hampazero@mail.ru (V.T.A.)

3 North Caucasus Federal University, 1a Pushkin St., Stavropol 355009, Russia. (V.T.A.)

* Correspondence: nenajdenko@org.chem.msu.ru (V.G.N.)

Table of contents

Synthesis of styrenes 2 by catalytic olefination reaction in EtOH or DMSO (general procedure I, 5 mmol scale) S2

Synthesis of styrenes 2 by catalytic olefination reaction in ethylene glycol (general procedure II) S2

Synthesis of styrene 2a by catalytic olefination reaction in EtOH (150 mmol scale) S2

Synthesis of styrenes 2 by Wittig reaction (general procedure III, 5 mmol scale) S4

Characterization data of styrenes 2 S4-S11

Synthesis of α-CF_3-β-(2-nitroaryl)enamines by the reaction with pyrrolidine in neat S11-S13

Synthesis of indoles 4 by the reduction of nitro-substituted enamines 3 (general procedure IV) S13

Multi-gramm scale synthesis of indole 4a S13

One pot synthesis of indoles 4 from styrenes 2 (general procedure V) S13

Characterization data of indoles 4a-4n,10a S14-S17

One pot synthesis of indoles 10b-g from styrenes 2 (general procedure VI) S17-S18

Characterization data of styrenes indoles 10b-g S18-S20

Reactions of indole 4a with electrophiles S20-S23

Structures and ^{19}F NMR data of reaction intermediates and by products S24-25

References S26

Copies of all NMR spectra S27-S186

Synthesis of styrenes 2 by catalytic olefination reaction in EtOH or DMSO (general procedure I, 5 mmol scale). [1] One neck 100 mL round bottomed flask was charged with $N_2H_4 \cdot H_2O$ (0.265 g, 5.25 mmol), and solution of corresponding benzaldehyde (5 mmol in 25 mL of EtOH or DMSO) was added and stirred for 3 h until aldehyde disappeared (TLC control). Next, 1,2-ethylenediamine (0.65 mL, 7.5 mmol), CuCl (0.050 g, 0.5 mmol) were added and stirred for 1-2 min. After that CF_3CCl_3 (1.78 mL, 15 mmol) was added in one portion at cooling by cold water bath. Reaction mixture stirred overnight at room temperature, poured into water (100 mL) and extracted with CH_2Cl_2 (3x20 mL). Combined extract was washed with water (20 mL) and dried over Na_2SO_4. Solvents were evaporated *in vacuo*, the residue was purified by passing through a short silica gel pad using 3:1 mixture of hexane and CH_2Cl_2 as an eluent.

Synthesis of styrenes 2 by catalytic olefination reaction in ethylene glycol (general procedure II). [2] One neck 50 mL round bottomed flask was charged with 1 mmol of corresponding benzaldehyde, 10 mL of ethylene glycol, 0.25 mL (5 mmol) of $N_2H_4 \cdot H_2O$ and stirred 0.5-1h until aldehyde disappeared (TLC control). Next, 0.38 ml (4.4 mmol) of 1,2-ethylenediamine, 0.0086 g (0.05 mmol) of $CuCl_2 \cdot 2H_2O$ was added and stirred for 1-2 min. After that CF_3CCl_3 (0.71 mL, 6 mmol) was added in one portion at cooling by cold water bath. Reaction mixture stirred overnight at room temperature, poured into water (50 mL) and extracted with CH_2Cl_2 (3x20 mL). Combined extract was washed with water (20 mL) and dried over Na_2SO_4. Solvents were evaporated *in vacuo*, the residue was purified by passing through a short silica gel pad using 3:1 mixture of hexane and CH_2Cl_2 as an eluent.

Synthesis of styrene 2a by catalytic olefination reaction in EtOH (150 mmol scale). One neck 1000 mL round bottomed flask was charged with $N_2H_4 \cdot H_2O$ (5.25 g, 105 mmol), and solution of 2-nitrobenzaldehyde (15.11 g, 100 mmol in 175 mL of EtOH) was added at vigorous stirring. The reaction mixture was stirred for 3 h until aldehyde disappeared (TLC control). Next, 1,2-ethylenediamine (10 mL, 150 mmol), CuCl (1 g, 10 mmol) were added and stirred for 1-2 min. After that CF_3CCl_3 (18 mL, 150 mmol) was added in one portion at cooling by cold water bath. The reaction mixture stirred overnight at room temperature, poured into HCl water solution (1000 mL, ~0.4-0.5 M) and extracted with CH_2Cl_2 (3x150 mL). Combined extract was washed with water (200 mL) and dried over Na_2SO_4. Solvents were evaporated *in vacuo*, the residue was purified by

passing through a short silica gel pad (~120-150 cm^3 of silica gel) using 3:1 mixture of hexane and CH_2Cl_2 as an eluent. Evaporation of the solvents afforded pure **2a** as slightly yellow oil. Yield 17.1 g (68%).

Synthesis of styrenes 2 by Wittig reaction (general procedure III, 5 mmol scale).[3] One neck 20 mL vial with a screw cap was charged with corresponding benzaldehyde (2 mmol), PPh_3 (1.258 g, 4.8 mmol), K_2CO_3 (0.028 g, 0.2 mmol), MeCN (2 mL) and CF_3CCl_3 (0.561 g, 3 mmol). The reaction mixture was stirred for 3-5 h at 80 °C and then poured into water (100 mL) and extracted with CH_2Cl_2 (3x20 mL). Combined extract was washed with water (20 mL) and dried over Na_2SO_4. Solvents were evaporated *in vacuo*, the residue was purified by column chromatography on silica gel using (**2b,g,i,k,n**) and 1:1 (**2j,l,m**) 3:1 mixtures of hexane and CH_2Cl_2 as eluents.

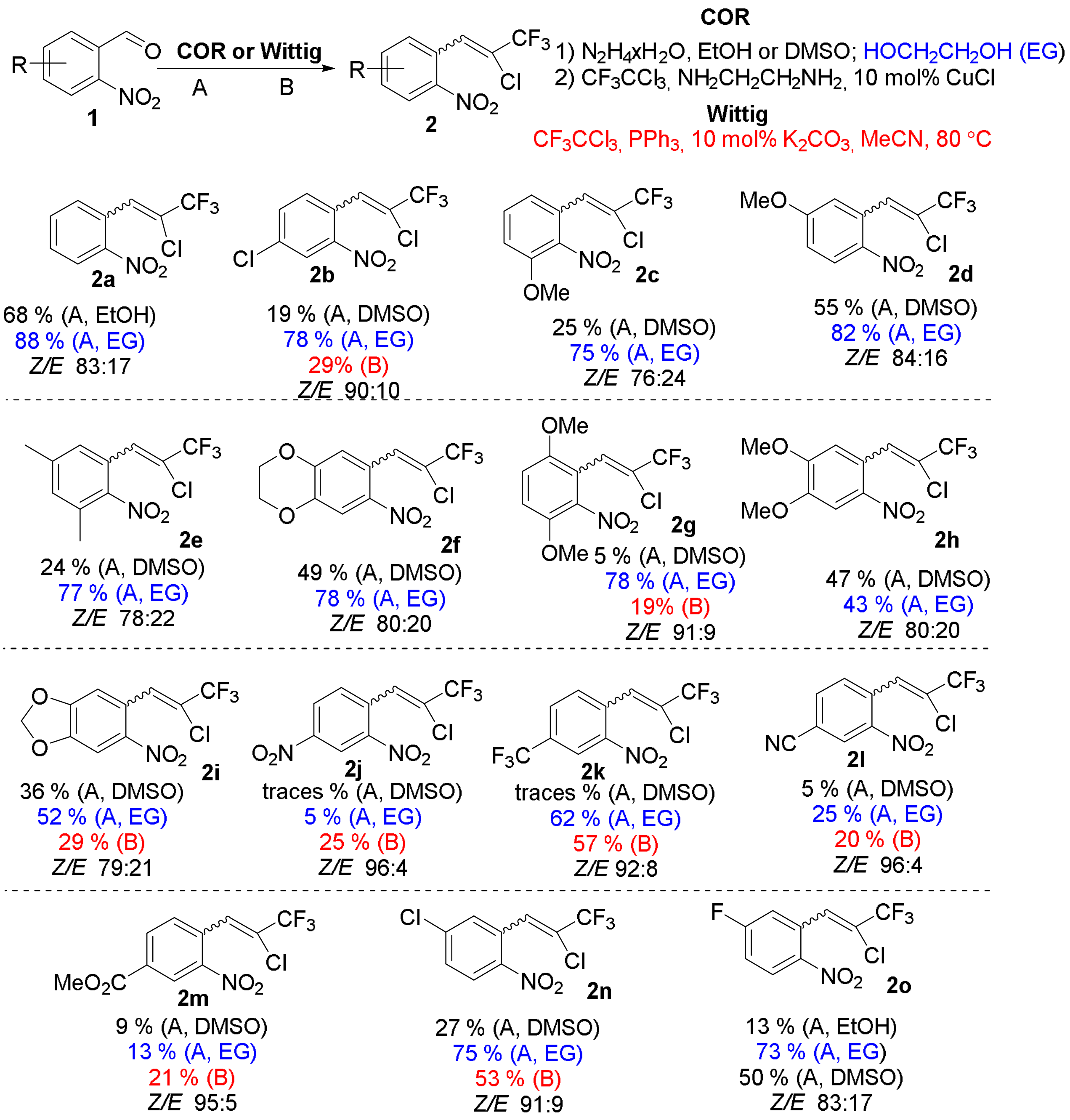

Scheme S1. Olefination of 2-nitrobenzaldehydes by various methods.

1-(2-Chloro-3,3,3-trifluoroprop-1-en-1-yl)-2-nitrobenzene (2a). Obtained from 2-**nitrobenzaldehyde**. Obtained from 2-nitrobenzaldehyde **1a** (0.151 g, 1 mmol) by procedure II. Colorless oil, yield 0.223 g (88%). Mixture of *Z*/*E* isomers (82:18; by ^{19}F NMR). For the mixture of isomers: *Z*-isomer: ^{1}H NMR ($CDCl_3$, 400.1 MHz): δ 8.21 (dd, 1H, ^{3}J = 8.2 Hz, ^{4}J = 1.2 Hz), 7.76-7.71 (m, 2H), 7.66-7.55 (m, 2H). $^{13}C\{^{1}H\}$

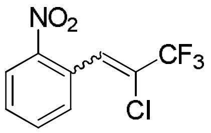

NMR ($CDCl_3$, 100.6 MHz): δ 147.1, 133.8, 131.2, 130.3, 129.4 (q, $^4J_{CF}$ = 4.7 Hz), 127.5, 125.1, 122.5 (q, $^2J_{CF}$ = 37.4 Hz), 120.3 (q, $^1J_{CF}$ = 272.6 Hz). ^{19}F NMR ($CDCl_3$, 376.5 MHz): δ -70.3 (s, 3F). *E*-isomer: ^{1}H NMR ($CDCl_3$, 400.1 MHz): δ 7.70-7.66 (m, 1H), 7.54 (s, 1H), 7.37 (d, 1H, ^3J = 7.6 Hz). Other signals are overlapped with those of major isomer. ^{13}C{^{1}H} NMR ($CDCl_3$, 100.6 MHz): δ 146.2, 133.8, 133.6 (q, $^4J_{CF}$ = 2.4 Hz), 131.0, 130.1, 128.3, 124.9, 120.1 (q, $^1J_{CF}$ = 274.2 Hz). Other signals are overlapped with those of major isomer or can not be seen in the spectrum due to the low concentration of minor isomer. ^{19}F NMR ($CDCl_3$, 376.5 MHz): δ -63.2 (s, 3F). NMR data of enamine **2c** are in agreement with those in the literature.[4]

4-Chloro-1-(2-chloro-3,3,3-trifluoroprop-1-en-1-yl)-2-nitrobenzene (2b). Obtained from 4-chloro-2-nitrobenzaldehyde **1b** (0.185g, 1 mmol) by procedure II. Light yellow oil, yield 0.223 g (78%). Mixture of *Z*/*E* isomers (90:10; by ^{19}F NMR). For the mixture of isomers: *Z*-isomer: ^{1}H NMR ($CDCl_3$, 400.1 MHz): δ 8.21 (d, 1H, ^4J = 2.1 Hz), 7.75-7.67 (m, 2H), 7.61 (d, 1H, ^3J = 8.3 Hz). ^{13}C{^{1}H} NMR ($CDCl_3$, 100.6 MHz): δ 147.6, 136.4, 133.9, 132.4, 128.2 (q, $^3J_{CF}$ = 4.8 Hz), 125.9, 125.4, 123.4 (q, $^2J_{CF}$ = 38.0 Hz), 120.2 (q, $^1J_{CF}$ = 272.8 Hz). ^{19}F NMR ($CDCl_3$, 376.5 MHz): δ -70.4 (d, 3F, ^4J = 1.0 Hz). *E*- isomer: ^{1}H NMR ($CDCl_3$, 400.1 MHz): δ 7.65 (dd, 1H, ^3J = 8.3 Hz, ^4J = 2.2 Hz), 7.46 (s, 1H), 7.32 (d, 1H, ^3J = 8.3 Hz). Other signals are overlapped with those of major isomer. ^{13}C{^{1}H} NMR ($CDCl_3$, 100.6 MHz): δ 136.2, 133.8, 132.1, 126.8, 125.2. Other signals are overlapped with those of major isomer or can not be seen in the spectrum due to the low concentration of minor isomer. ^{19}F NMR ($CDCl_3$, 376.5 MHz): δ -63.2 (s, 3F). HRMS (ESI-TOF): m/z $[M+Ag]^+$ Calcd for $C_9H_4Cl_2F_3NO_2Ag^+$: 393.8610; found: 393.8619.

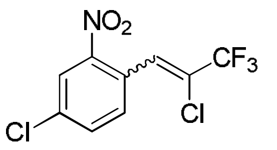

1-(2-Chloro-3,3,3-trifluoroprop-1-en-1-yl)-3-methoxy-2-nitrobenzene (2c). Obtained from 3-methoxy-2-nitrobenzaldehyde **1c** (0.188 g, 1.039 mmol) by procedure II. Yellow crystals, mp 42-44 °C, yield 0.211 g (75%). Mixture of *Z*/*E* isomers (76:24; by ^{19}F NMR). For the mixture of isomers: *Z*-isomer: ^{1}H NMR ($CDCl_3$, 400.1 MHz): δ 7.50 (t, 1H, ^3J = 8.2 Hz), 7.34 (d, 1H, ^3J = 7.8 Hz), 7.24 (s, 1H), 7.13 (d, 1H, ^3J = 8.5 Hz), 3.91 (s, 3H). ^{13}C{^{1}H} NMR ($CDCl_3$, 100.6 MHz): δ 151.26, 140.4, 131.43, 125.4, 125.0 (q, $^3J_{CF}$ = 4.5 Hz), 124.9 (q, $^2J_{CF}$ = 37.6 Hz), 121.0, 120.1 (q, $^1J_{CF}$ = 273.9 Hz), 114.0, 56.51. ^{19}F NMR ($CDCl_3$, 376.5 MHz): δ - 70.3 (s, 3F). *E*- isomer: ^{1}H NMR ($CDCl_3$, 400.1 MHz): δ 7.43 (t, 1H, ^3J = 8.2 Hz), 7.17 (s, 1H), 7.08 (d, 1H, ^3J = 8.5 Hz), 6.86 (d, 1H, ^3J = 7.8 Hz), 3.90 (s, 3H). ^{13}C{^{1}H} NMR ($CDCl_3$, 100.6 MHz): δ 151.24,

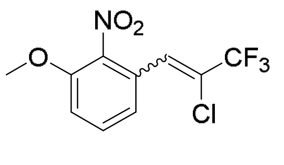

139.2, 131.48, 130.0 (q, $^3J_{CF}$ = 2.1 Hz), 126.8, 125.3 (q, $^2J_{CF}$ = 37.6 Hz), 120.8 (q, $^4J_{CF}$ = 2.5 Hz), 119.8 (q, $^1J_{CF}$ = 273.9 Hz), 113.4, 56.46. ^{19}F NMR ($CDCl_3$, 376.5 MHz): δ - 63.6 (s, 3F). HRMS (ESI-TOF): m/z $[M+NH_4]^+$ Calcd for $C_{10}H_{11}ClF_3N_2O_3^+$: 299.0405; found: 299.0404.

2-(2-Chloro-3,3,3-trifluoroprop-1-en-1-yl)-4-methoxy-1-nitrobenzene (2d). Obtained from 5-methoxy-2-nitrobenzaldehyde **1d** (0.183 g, 1.011 mmol) by procedure II. Yellow oil, yield 0.234 g (82%). Mixture of *Z*/*E* isomers (84:16; by ^{19}F NMR). For the mixture of isomers: *Z*-isomer: ^{1}H NMR ($CDCl_3$, 400.1 MHz): δ 8.25-8.21 (m, 1H), 7.75 (s, 1H), 7.05-6.99 (m, 2H), 3.92 (s, 3H). $^{13}C\{^1H\}$ NMR ($CDCl_3$, 100.6 MHz): δ 163.6, 140.0, 130.1 (q, $^3J_{CF}$ = 4.8 Hz), 130.0, 127.7, 120.4 (q, $^1J_{CF}$ = 272.6 Hz), 121.9 (q, $^2J_{CF}$ = 37.7 Hz), 116.2, 114.7, 56.1. ^{19}F NMR ($CDCl_3$, 376.5 MHz): δ - 70.2 (s, 3F). *E*- isomer: ^{1}H NMR ($CDCl_3$, 400.1 MHz): δ 8.20 (d, 1H, ^3J = 6.6 Hz), 7.51 (s, 1H), 6.97 (d, 1H, ^3J = 2.8 Hz), 6.77 (d, 1H, ^3J = 2.7 Hz), 3.90 (s, 3H). $^{13}C\{^1H\}$ NMR ($CDCl_3$, 100.6 MHz): δ 163.5, 139.2, 134.1 (q, $^3J_{CF}$ = 2.3 Hz), 130.9, 127.5, 120.1 (q, $^1J_{CF}$ = 274.2 Hz), 121.2 (q, $^2J_{CF}$ = 37.5 Hz), 115.9 (q, $^3J_{CF}$ = 2.6 Hz), 114.5, 56.04. ^{19}F NMR ($CDCl_3$, 376.5 MHz): δ - 63.0 (s, 3F). HRMS (ESI-TOF): m/z $[M+Na]^+$ Calcd for $C_{10}H_7ClF_3NO_3Na^+$: 303.9959; found: 303.9957.

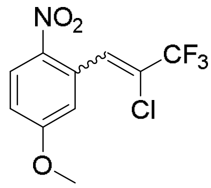

1-(2-Chloro-3,3,3-trifluoroprop-1-en-1-yl)-3,5-dimethyl-2-nitrobenzene (2e). Obtained from 3,5-dimethyl-2-nitrobenzaldehyde **1e** (0.174 g, 0.972 mmol) by procedure II. Yellow oil, yield 0.214 g (77%). Mixture of *Z*/*E* isomers (78:22; by ^{19}F NMR). For the mixture of isomers: *Z*-isomer: ^{1}H NMR ($CDCl_3$, 400.1 MHz): δ 7.36 (s, 1H), 7.31 (s, 1H), 7.17 (s, 1H), 2.40 (s, 3H), 2.36 (s, 3H). $^{13}C\{^1H\}$ NMR ($CDCl_3$, 100.6 MHz): δ 147.8, 141.5, 133.4, 131.4, 128.1, 126.8 (q, $^3J_{CF}$ = 4.6 Hz), 124.9, 123.7 (q, $^2J_{CF}$ = 37.5 Hz), 120.2 (q, $^1J_{CF}$ = 272.7 Hz), 21.1, 18.2. ^{19}F NMR ($CDCl_3$, 376.5 MHz): δ -70.3 (s, 3F). *E*- isomer: ^{1}H NMR ($CDCl_3$, 400.1 MHz): δ 7.23 (s, 1H), 7.13 (s, 1H), 6.94 (s, 1H). Other signals are overlapped with those of major isomer. $^{13}C\{^1H\}$ NMR ($CDCl_3$, 100.6 MHz): δ 146.6, 141.6, 133.1, 131.8 (q, $^3J_{CF}$ = 2.3 Hz), 131.6, 127.9 (q, $^4J_{CF}$ = 2.4 Hz), 126.4, 123.8 (q, $^2J_{CF}$ = 37.4 Hz), 119.9 (q, $^1J_{CF}$ = 274.5 Hz), 20.9, 18.3. ^{19}F NMR ($CDCl_3$, 376.5 MHz): δ -63.5 (s, 3F). HRMS (ESI-TOF): m/z $[M+H]^+$ Calcd for $C_{11}H_{10}ClF_3NO_2^+$: 280.0347; found: 280.0641.

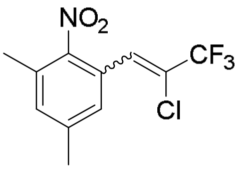

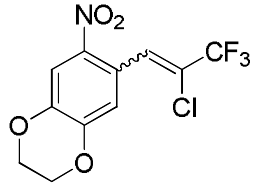

6-(2-Chloro-3,3,3-trifluoroprop-1-en-1-yl)-7-nitro-2,3-dihydrobenzo[b][1,4]dioxine (2f). Obtained from 7-nitro-2,3-dihydrobenzo[*b*][1,4]dioxine-6-carbaldehyde **1f** (0.212 g, 1.014 mmol) by procedure II. Pale yellow crystals, mp 104-106 °C, yield 0.241 g (78%). Mixture of *Z*/*E* isomers (80:20; by ^{19}F NMR). For the mixture of isomers: *Z*-isomer: ^{1}H NMR ($CDCl_3$, 400.1 MHz): δ 7.81 (s, 1H), 7.68 (*pseudo*-d, 1H, $^{4}J = 0.8$ Hz), 7.10 (s, 1H), 4.45-4.28 (m, 4H). ^{13}C{^{1}H} NMR ($CDCl_3$, 100.6 MHz): δ 148.2, 143.9, 129.2 (q, $^{3}J_{CF} = 4.6$ Hz), 121.5, 121.4 (q, $^{2}J_{CF} = 37.5$ Hz), 120.5 (q, $^{1}J_{CF} = 272.3$ Hz), 119.3, 115.0, 64.7, 64.3. ^{19}F NMR ($CDCl_3$, 376.5 MHz): δ -70.2 (d, 3F, $^{4}J = 1.0$ Hz). *E*- isomer: ^{1}H NMR ($CDCl_3$, 400.1 MHz): δ 7.80 (s,1H), 7.43 (*pseudo*-d, 1H, $^{4}J = 0.8$ Hz), 6.79 (s, 1H). Other signals are overlapped with those of major isomer. ^{13}C{^{1}H} NMR ($CDCl_3$, 100.6 MHz): δ 143.8, 140.5, 133.8 (q, $^{3}J_{CF} = 2.3$ Hz), 122.6, 121.5 (q, $^{2}J_{CF} = 37.3$ Hz), 120.2 (q, $^{1}J_{CF} = 274.1$ Hz), 119.0 (q, $^{3}J_{CF} = 2.5$ Hz), 114.8, 64.2. Other signals are overlapped with those of major isomer or can not be seen in the spectrum due to the low concentration of minor isomer. ^{19}F NMR ($CDCl_3$, 376.5 MHz): δ -63.1 (s, 3F). HRMS (ESI-TOF): m/z [M+H]$^+$ Calcd for $C_{11}H_8ClF_3NO_4^+$: 310.0088; found: 310.0086.

2-(2-Chloro-3,3,3-trifluoroprop-1-en-1-yl)-1,4-dimethoxy-3-nitrobenzene (2g). Obtained from 1,4-dimethoxy-3-nitrobenzaldehyde **1g** (0.222 g, 1.052 mmol) by procedure II. Pale yellow crystals, mp 72-73 °C, yield 0.254 g (78%). Mixture of *Z*/*E* isomers (91:9; by ^{19}F NMR). For the mixture of isomers: *Z*-isomer: ^{1}H NMR ($CDCl_3$, 400.1 MHz): δ 7.18 (s, 1H), 7.07 (d, 1H, $^{3}J = 9.3$ Hz), 7.03 (d, 1H, $^{3}J = 9.2$ Hz), 3.87 (s, 3H), 3.83 (s, 3H). ^{13}C{^{1}H} NMR ($CDCl_3$, 100.6 MHz): δ 150.2, 145.1, 126.8 (q, $^{2}J_{CF} = 37.8$ Hz), 124.0 (q, $^{3}J_{CF} = 4.6$ Hz), 119.9 (q, $^{1}J_{CF} = 272.9$ Hz), 115.4, 114.4, 113.9, 57.0, 56.5. ^{19}F NMR ($CDCl_3$, 376.5 MHz): δ -70.3 (d, 3F, $^{4}J = 1.0$ Hz). *E*- isomer: ^{1}H NMR ($CDCl_3$, 400.1 MHz): δ 6.99 (d, 1H, $^{3}J = 3.2$ Hz), 6.98 (d, 1H, $^{3}J = 3.2$ Hz), 6.88 (s, 1H), 3.85 (s, 3H), 3.79 (s, 3H). ^{13}C{^{1}H} NMR ($CDCl_3$, 100.6 MHz): δ 144.7, 140.4, 113.3, 56.9, 56.3. Other signals are overlapped with those of major isomer or can not be seen in the spectrum due to the low concentration of minor isomer. ^{19}F NMR ($CDCl_3$, 376.5 MHz): δ -67.8 (s, 3F). HRMS (ESI-TOF): m/z [M+H]$^+$ Calcd for $C_{11}H_{10}ClF_3NO_4^+$: 312.0245; found: 312.0251.

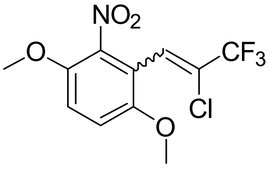

1-(2-Chloro-3,3,3-trifluoroprop-1-en-1-yl)-4,5-dimethoxy-2-nitrobenzene (2h). Obtained from

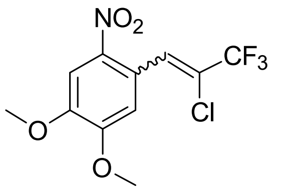

2,5-dimethoxy-3-nitrobenzaldehyde **1h** by procedure I (0.539 g, 2.55 mmol, DMSO) and by procedure II (0.245 g, 1.161 mmol). Pale yellow solid, mp 95-97 °C, yield 0.374 g (47%, I) yield 0.128 g (43%, II). Mixture of *Z*/*E* isomers (80:20; by ^{19}F NMR). For the mixture of isomers: *Z*-isomer: ^{1}H NMR ($CDCl_3$, 400.1 MHz): δ 7.79-7.74 (m, 2H), 7.02 (s, 1H), 3.99 (s, 3H), 3.99 (s, 3H). ^{13}C{^{1}H} NMR ($CDCl_3$, 100.6 MHz): δ 153.2, 149.4, 140.1, 129.9 (q, $^{3}J_{CF}$ = 4.7 Hz), 121.6, 121.4 (q, $^{2}J_{CF}$ = 37.3 Hz), 120.4 (q, $^{1}J_{CF}$ = 272.4 Hz), 112.1, 107.7, 56.6, 56.40. ^{19}F NMR ($CDCl_3$, 376.5 MHz): δ -70.1 (d, 3F, ^{4}J = 1.0 Hz). *E*-isomer: ^{1}H NMR ($CDCl_3$, 400.1 MHz): δ 7.52 (*pseudo*-d, 1H, ^{4}J = 0.6 Hz), 6.71 (s, 1H), 3.97 (s, 3H), 3.95 (s, 3H). Other signals are overlapped with those of major isomer. ^{13}C{^{1}H} NMR ($CDCl_3$, 100.6 MHz): δ 149.3, 138.9, 134.3 (q, $^{3}J_{CF}$ = 2.3 Hz), 122.7, 120.2 (q, $^{1}J_{CF}$ = 274.3 Hz), 121.1 (q, $^{2}J_{CF}$ = 37.2 Hz), 112.1, 107.5, 56.5, 56.38. ^{19}F NMR ($CDCl_3$, 376.5 MHz): δ -62.8 (s, 3F). HRMS (ESI-TOF): m/z $[M+H]^+$ Calcd for $C_{11}H_{10}ClF_3NO_4^+$: 312.0245; found: 312.0254.

5-(2-Chloro-3,3,3-trifluoroprop-1-en-1-yl)-6-nitrobenzo[d][1,3]dioxole (2i). Obtained from 4,5-

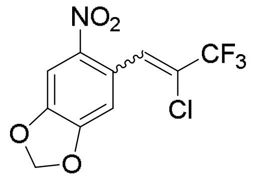

ethylendioxy-2-nitrobenzaldehyde **1i** (0.207 g, 1.062 mmol) by procedure II. Pale yellow solid, mp 100-103 °C, yield 0.155 g (52 %). Mixture of *Z*/*E* isomers (79:21; by ^{19}F NMR). For the mixture of isomers: *Z*-isomer: ^{1}H NMR ($CDCl_3$, 400.1 MHz): δ 7.68-7.66 (m, 2H), 6.99 (s, 1H). 6.19 (s, 2H). ^{13}C{^{1}H} NMR ($CDCl_3$, 100.6 MHz): δ 152.22, 148.9, 141.8, 129.7 (q, $^{3}J_{CF}$ = 4.8 Hz), 123.9, 121.8 (q, $^{2}J_{CF}$ = 37.5 Hz), 120.4 (q, $^{1}J_{CF}$ = 272.5 Hz), 109.6, 105.7, 103.64. ^{19}F NMR ($CDCl_3$, 376.5 MHz): δ -70.2 (s, 3F). *E*- isomer: ^{1}H NMR ($CDCl_3$, 400.1 MHz): δ 7.65 (s, 1H), 7.43 (d, 1H, ^{4}J = 0.6 Hz), 6.70 (s, 1H), 6.17 (s, 2H). ^{13}C{^{1}H} NMR ($CDCl_3$, 100.6 MHz): δ 152.19, 148.8, 140.6, 134.0 (q, $^{3}J_{CF}$ = 2.8 Hz), 124.9, 121.3 (q, $^{2}J_{CF}$ = 37.4 Hz), 120.1 (q, $^{1}J_{CF}$ = 274.5 Hz), 109.5 (q, $^{4}J_{CF}$ = 2.8 Hz), 105.4, 103.62. ^{19}F NMR ($CDCl_3$, 376.5 MHz): δ -63.2 (s, 3F). HRMS (ESI-TOF): m/z $[M+Na]^+$ Calcd for $C_{10}H_5ClF_3NO_4Na^+$: 317.9751; found: 317.9752.

1-(2-Chloro-3,3,3-trifluoroprop-1-en-1-yl)-2,4-dinitrobenzene (2j). Obtained from 2,4-

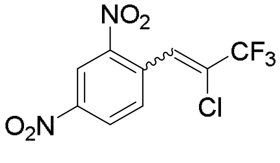

dinitrobenzaldehyde by procedure (II, 0.196 g) and (III, 0.65 g). Yellow viscous oil, yield 0.014 g (5 %, II), 0.248 (25 %, (III). Mixture of *Z*/*E* isomers (96:4; by ^{19}F NMR). For the mixture of isomers: *Z*-isomer: ^{1}H NMR ($CDCl_3$, 400.1 MHz): δ 9.03 (*pseudo*-d, 1H, ^{4}J ~ 1.5 Hz), 8.58 (dd, 1H, ^{3}J = 8.5 Hz, ^{4}J = 2.3 Hz), 7.91 (d, 1H,

^{3}J = 8.5 Hz), 7.78 (s, 1H). $^{13}C\{^{1}H\}$ NMR ($CDCl_3$, 100.6 MHz): δ 148.1, 147.4, 140.2, 133.0, 127.9, 127.6 (q, $^{3}J_{CF}$ = 4.5 Hz), 125.0 (q, $^{2}J_{CF}$ = 38.0 Hz), 120.6, 119.9 (q, $^{1}J_{CF}$ = 273.2 Hz). ^{19}F NMR ($CDCl_3$, 376.5 MHz): δ -69.4 (d, 3F, ^{4}J = 0.6 Hz). *E*- isomer: ^{1}H NMR ($CDCl_3$, 400.1 MHz): δ 7.78 (br.s, 1H), 8.52 (dd, 1H, ^{3}J = 8.5 Hz, ^{4}J = 2.3 Hz), 7.64 (d, 1H, ^{3}J = 8.5 Hz), 7.53 (s, 1H). $^{13}C\{^{1}H\}$ NMR ($CDCl_3$, 100.6 MHz): 127.0. Other signals are overlapped with those of major isomer or can not be seen in the spectrum due to the low concentration of minor isomer. ^{19}F NMR ($CDCl_3$, 376.5 MHz): δ -62.2 (s, 3F). HRMS (ESI-TOF): m/z $[M-H]^-$ Calcd for $C_9H_3ClF_3N_2O_4^-$: 294.9739; found: 294.9732.

1-(2-Chloro-3,3,3-trifluoroprop-1-en-1-yl)-2-nitro-4-(trifluoromethyl)benzene (2k). Obtained from 2-nitro-4-(trifluoromethyl)benzaldehyde **1k** by procedure II (0.438 g, 2 mmol) and by procedure III (0.438 g, 2 mmol). Yellow oil, yield 0.395 g (62%, II), 0.365 g (57 %, III). Mixture of *Z*/*E* isomers (92:8; by ^{19}F NMR). For the mixture of isomers: *Z*-isomer: ^{1}H NMR ($CDCl_3$, 400.1 MHz): δ 8.49 (*pseudo*-d, 1H, ^{4}J ~ 1.0 Hz), 7.99 (dd, 1H, ^{3}J = 8.0 Hz, ^{4}J = 0.7 Hz), 7.81 (d, 1H, ^{3}J = 8.1 Hz), 7.77 (s, 1H). $^{13}C\{^{1}H\}$ NMR ($CDCl_3$, 100.6 MHz): δ 147.2, 132.8 (q, $^{2}J_{CF}$ = 34.6 Hz), 132.4, 131.12, 130.3 (q, $^{3}J_{CF}$ = 3.4 Hz), 128.2 (q, $^{3}J_{CF}$ = 4.8 Hz), 124.3 (q, $^{2}J_{CF}$ = 38.1 Hz), 122.53 (q, $^{3}J_{CF}$ = 3.8 Hz), 122.46 (q, $^{1}J_{CF}$ = 273.1 Hz), 120.1 (q, $^{1}J_{CF}$ = 272.8 Hz). ^{19}F NMR ($CDCl_3$, 376.5 MHz): δ -69.5 (s, 3F), -63.3 (s, 3F). *E*- isomer: ^{1}H NMR ($CDCl_3$, 400.1 MHz): δ 7.94 (dd, 1H, ^{3}J = 8.0 Hz, ^{4}J = 0.7 Hz), 7.56 (s, 1H), 7.53 (d, 1H, ^{3}J = 8.2 Hz). Other signals are overlapped with those of major isomer. ^{19}F NMR ($CDCl_3$, 376.5 MHz): δ-62.3 (s, 3F). Other signals are overlapped with those of major isomer. HRMS (ESI-TOF): m/z $[M+Ag]^+$ Calcd for $C_{10}H_4ClF_6NO_2Ag^+$: 425.8880; found: 425.8874.

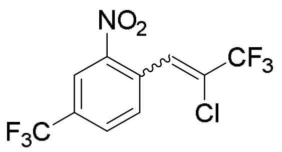

4-(2-Chloro-3,3,3-trifluoroprop-1-en-1-yl)-3-nitrobenzonitrile (2l). Obtained from 4-cyano-2-nitrobenzaldehyde **1l** by procedure II (0.176 g, 1 mmol) and by procedure III (0.88 g, 5 mmol). Yellow oil, yield 0.070 g (25 %) (II), 0.278 g (20 %, III). Mixture of *Z*/*E* isomers (96:4; by ^{19}F NMR). For the mixture of isomers: *Z*-isomer: ^{1}H NMR ($CDCl_3$, 400.1 MHz): δ 8.51 (d, 1H, ^{4}J = 1.6 Hz), 8.01 (dd, 1H, ^{3}J = 8.1 Hz, ^{4}J = 1.6 Hz), 7.81 (d, 1H, ^{3}J = 8.1 Hz), 7.75 (s, 1H). $^{13}C\{^{1}H\}$ NMR ($CDCl_3$, 100.6 MHz): δ 147.1, 136.6, 132.5, 131.7, 128.7, 127.9 (q, $^{3}J_{CF}$ = 4.7 Hz), 124.3 (q, $^{2}J_{CF}$ = 38.5 Hz), 119.8 (q, $^{1}J_{CF}$ = 273.0 Hz, CF_3), 115.9, 114.4. ^{19}F NMR ($CDCl_3$, 376.5 MHz): δ - 70.5 (s, 3F). *E*-isomer: ^{1}H NMR ($CDCl_3$, 400.1 MHz): δ 7.95 (dd, 1H, ^{3}J = 8.0 Hz, ^{4}J = 1.6 Hz), 7.50 (s, 1H). Other signals are overlapped with those of major

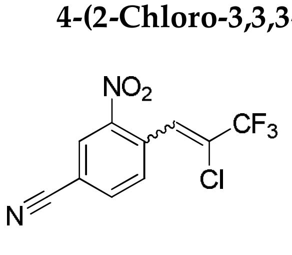

isomer. ^{19}F NMR ($CDCl_3$, 376.5 MHz): δ -63.3 (s, 3F). HRMS (ESI-TOF): m/z $[M+H]^+$ Calcd for $C_{10}H_5ClF_3N_2O_2^+$: 276.9986; found: 276.9986.

Methyl 4-(2-chloro-3,3,3-trifluoroprop-1-en-1-yl)-3-nitrobenzoate (2m). Obtained from methyl 4-formyl-3-nitrobenzoate **1m** by procedure II (0.209 g, 1 mmol) and by procedure III (0.209 g, 1 mmol). Beige crystals, yield 0.040 g (13%, II), 0.079 g (22 %, III). Mixture of *Z*/*E* isomers (95:5; by ^{19}F NMR). ^{1}H NMR ($CDCl_3$, 400.1 MHz): δ 8.81 (d, 1H, ^4J = 1.7 Hz), 8.35 (dd, 1H, ^3J = 8.0 Hz, ^4J = 1.7 Hz), 7.76 (s, 1H), 7.73 (d, 1H, ^3J = 8.1 Hz), 3.99 (s, 3H). ^{13}C{^{1}H} NMR ($CDCl_3$, 100.6 MHz): δ 164.3, 147.2, 134.2, 132.4, 131.7, 131.4, 128.6 (q, $^3J_{CF}$ = 4.7 Hz), 126.1, 123.7 (q, $^2J_{CF}$ = 38.0 Hz), 120.1 (q, $^1J_{CF}$ = 272.9 Hz), 53.0. ^{19}F NMR ($CDCl_3$, 376.5 MHz): δ -70.4 (s, 3F). HRMS (ESI-TOF): m/z $[M+H]^+$ Calcd for $C_{11}H_8ClF_3NO_4^+$: 310.0088; found: 310.0085.

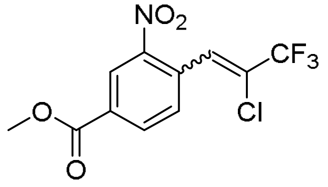

4-Chloro-2-(2-chloro-3,3,3-trifluoroprop-1-en-1-yl)-1-nitrobenzene (2n). Obtained from 5-chloro-2-nitrobenzaldehyde **1n** (0.191g, 1.03 mmol) by procedure II. Yellow crystals, mp 46-48 °C, yield 0.221 g (75 %). Mixture of *Z*/*E* isomers (91:9; by ^{19}F NMR). For the mixture of isomers: *Z*-isomer: ^{1}H NMR ($CDCl_3$, 400.1 MHz): δ 8.19 (d, 1H, ^3J = 8.8 Hz), 7.70 (s, 1H), 7.61 (d, 1H, ^4J = 2.1 Hz), 7.58-7.55 (m, 1H). ^{13}C{^{1}H} NMR ($CDCl_3$, 100.6 MHz): δ 145.3, 140.4, 131.1, 130.3, 129.2, 128.3 (q, $^3J_{CF}$ = 4.8 Hz), 126.5, 123.4 (q, $^2J_{CF}$ = 38.0 Hz), 120.1 (q, $^1J_{CF}$ = 272.9 Hz). ^{19}F NMR ($CDCl_3$, 376.5 MHz): δ - 70.5 (s, 3F). *E*- isomer: ^{1}H NMR ($CDCl_3$, 400.1 MHz): δ 7.46 (s, 1H). Other signals are overlapped with those of major isomer. ^{19}F NMR ($CDCl_3$, 376.5 MHz): δ - 63.3 (s, 3F). HRMS (ESI-TOF): m/z $[M+Ag]^+$ Calcd for $C_9H_4Cl_2F_3NO_2Ag^+$: 395.8583; found: 395.8587.

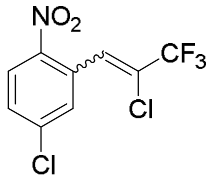

2-(2-Chloro-3,3,3-trifluoroprop-1-en-1-yl)-4-fluoro-1-nitrobenzene (2o). Obtained from 5-fluoro-2-nitrobenzaldehyde **1o** (0.175g, 1.04 mmol) by procedure II. White crystals, mp 35-38 °C, yield 0.204 g (73 %). Mixture of *Z*/*E* isomers (83:17; by ^{19}F NMR). For the mixture of isomers: *Z*-isomer: ^{1}H NMR ($CDCl_3$, 400.1 MHz): δ 8.28 (dd, 1H, ^3J = 9.1 Hz, ^3J = 5.1 Hz), 7.73 (s, 1H), 7.34 (dd, 1H, ^3J = 8.5 Hz, ^4J = 2.6 Hz), 7.31-7.25 (m, 1H). ^{13}C{^{1}H} NMR ($CDCl_3$, 100.6 MHz): δ 164.9 (d, $^1J_{CF}$ = 259.2 Hz), 143.3 (d, $^4J_{CF}$ = 2.5 Hz), 130.6 (d, $^3J_{CF}$ = 10.0 Hz), 128.5 (qd, $^3J_{CF}$ = 4.5 Hz, $^4J_{CF}$ = 0.9 Hz), 128.1 (d, $^3J_{CF}$ = 10.2 Hz), 123.4 (q, $^2J_{CF}$ = 37.9 Hz), 120.2 (q, $^1J_{CF}$ = 272.8 Hz), 118.4 (d, $^2J_{CF}$ = 25.1 Hz), 117.3 (d, $^2J_{CF}$ = 23.1 Hz). ^{19}F NMR ($CDCl_3$, 376.5

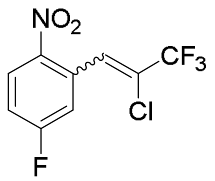

MHz): δ -70.5 (s, 3F), -102.55 - -102.71 (m, 1F). *E*- isomer: ^{1}H NMR ($CDCl_3$, 400.1 MHz): δ 7.48 (s, 1H), 7.25-7.22 (m, 1H) 7.07 (dd, 1H, ^3J = 8.2 Hz, ^4J = 2.7 Hz). Other signals are overlapped with those of major isomer. ^{13}C{^{1}H} NMR ($CDCl_3$, 100.6 MHz): δ 164.7 (d, $^1J_{CF}$ = 259.8 Hz), 142.4, 132.3 (br.s), 131.4 (d, $^3J_{CF}$ = 9.9 Hz), 127.9 (d, $^3J_{CF}$ = 10.2 Hz), 122.6 (q, $^2J_{CF}$ = 37.5 Hz), 120.0 (q, $^1J_{CF}$ = 274.4 Hz), 116.9, 118.0 (q, $^4J_{CF}$ = 2.4 Hz). ^{19}F NMR ($CDCl_3$, 376.5 MHz): δ -63.3 (s, 3F), -102.73 - -102.88 (m, 1F). HRMS (ESI-TOF): m/z [M-F]$^+$ Calcd for $C_9H_4ClF_3NO_2^+$: 249.9877; found: 249.9873.

Synthesis of α-CF_3-β-(2-nitroaryl)enamines by the reaction with pyrrolidine in neat (general procedure) [5]. A one neck 25 mL round bottomed flask was charged with dry pyrrolidine (8.5 mL, 100 mmol), cooled down to -18 °C and corresponding styrene **2** (10 mmol) was added in one portion with vigorous stirring. The reaction mixture was stirred at room temperature for 1-3 h until all starting styrene was consumed (TLC or NMR monitoring). The excess of pyrrolidine was evaporated in vacuum, the viscous residue was dissolved in CH_2Cl_2 (50 mL), washed with water (3×50 mL) and dried over Na_2SO_4. CH_2Cl_2 was removed *in vacuo*, and the residue was filtered through a short silica gel pad using 1:1 mixtures of hexane and CH_2Cl_2.

1-[(1Z)-2-(2-Nitrophenyl)-1-(trifluoromethyl)vinil]pyrrolidine (3a). Obtained from 1-(2-chloro-3,3,3-trifluoroprop-1-en-1-yl)-2-nitrobenzene **2a** (6.04 g, 24 mmol). Yellow oil, yield 6.733 g (98%). Mixture of *Z*/*E* isomers (86:14; ^{19}F NMR). For the mixture of isomers: *Z*-isomer: ^{1}H NMR ($CDCl_3$, 400.1 MHz): δ 7.94 (dd, 1H, ^3J = 8.2 Hz, ^4J = 1.1 Hz), 7.54 (td, 1H, ^3J = 7.7 Hz, ^4J = 1.1 Hz), 7.37-7.25 (m, 2H), 6.23 (s, 1H), 3.04-2.96 (m, 4H), 1.84-1.71 (m, 4H). ^{19}F NMR ($CDCl_3$, 376.5 MHz): δ -66.0 (s, 3F). *E*-isomer: ^{1}H NMR ($CDCl_3$, 400.1 MHz): δ 7.64-7.58 (m, 1H), 7.49 (td, 1H, ^3J = 7.6 Hz, ^4J = 1.2 Hz), 5.76 (s, 1H), 3.30-3.22 (m, 4H), 2.00-1.91 (m, 4H). Other signals are overlapped with those of major isomer. ^{19}F NMR ($CDCl_3$, 376.5 MHz): δ -59.9 (s, 3F). NMR data of enamine **3a** are in agreement with those in the literature.[5]

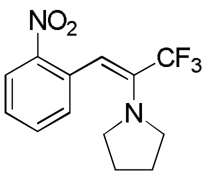

1-[(1Z)-2-(3-Methoxy-2-nitrophenyl)-1-(trifluoromethyl)vinil]pyrrolidine (3c). Obtained from 1-(2-chloro-3,3,3-trifluoroprop-1-en-1-yl)-3-methoxy-2-nitrobenzene **2c** (0.211 g, 0.75 mmol). Orange oil, yield 0.190 g (80%). Mixture of *Z*/*E* isomers (84:16; ^{19}F NMR). For the mixture of isomers: *Z*-isomer: ^{1}H NMR ($CDCl_3$, 400.1 MHz): δ 7.29 (t, 1H, ^3J = 8.1 Hz), 6.83 (d, 1H, ^3J = 8.1 Hz), 6.74 (d, 1H, ^3J = 8.1 Hz), 5.69 (s, 1H), 3.81 (s, 3H), 3.00-2.97 (m, 4H), 1.79-1.69 (m, 4H). ^{19}F NMR ($CDCl_3$, 376.5 MHz): δ -66.0 (s, 3F). *E*-isomer: ^{1}H

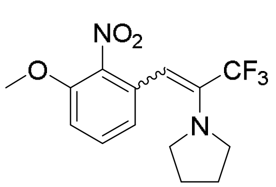

NMR ($CDCl_3$, 400.1 MHz): 7.23-7.20 (m, 1H), 6.84 (d, 1H, ^{3}J = 8.0 Hz), 6.79 (d, 1H, ^{3}J = 8.0 Hz), 5.21 (s, 1H), 3.80 (s, 3H), 3.16-3.12 (m, 4H), 1.87-1.84 (m, 4H). Other signals are overlapped with those of major isomer. ^{19}F NMR ($CDCl_3$, 376.5 MHz): δ -60.2 (s, 3F). NMR data of enamine **3c** are in agreement with those in the literature.[6]

1-Hydroxy-7-methoxy-2-(pyrrolidin-1-yl)-2-(trifluoromethyl)indolin-3-one (5c). Obtained from 1-[2-chloro-3,3,3-trifluoro-1-propenyl]-3-methoxy-2-nitrobenzene **2c** as an admixture in the synthesis of enamine **3c**. Orange oil, yield 0.036 g (15%). ^{1}H NMR ($CDCl_3$, 400.1 MHz): δ 7.74 (s, 1H), 7.24-7.28 (m, 1H), 7.01-7.15 (m, 2H), 3.90 (s, 3H), 3.11 (dd, 2H, ^{3}J = 7.2 Hz), 2.95 (q, 2H, ^{3}J = 6.9 Hz), 1.78 (t, 4H, ^{3}J = 6.2 Hz). $^{13}C\{^{1}H\}$ NMR ($CDCl_3$, 100.6 MHz): δ 192.4, 152.3, 149.3, 124.9, 122.6, 122.4 (q, $^{1}J_{CF}$ = 284.8 Hz), 118.9, 115.6, 86.4 (q, $^{2}J_{CF}$ = 28.1 Hz), 55.9, 47.8, 24.4. ^{19}F NMR ($CDCl_3$, 376.5 MHz): δ -73.6 (s, 3F). HRMS (ESI-TOF): m/z $[M+H]^+$ Calcd for $C_{14}H_{16}F_3N_2O_3^+$: 317.1108; found: 317.1109.

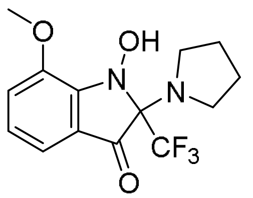

1-[(1*Z*)-2-(5-methoxy-2-nitrophenyl)-1-(trifluoromethyl)vinil]pyrrolidine (3d). Obtained from 2-(2-chloro-3,3,3-trifluoroprop-1-en-1-yl)-4-methoxy-1-nitrobenzene **2d** (0.976 g, 3.465 mmol). Orange oil, yield 1.074 g (98%). Mixture of *Z*/*E* isomers (86:14; ^{19}F NMR). For the mixture of isomers: *Z*-isomer: ^{1}H NMR ($CDCl_3$, 400.1 MHz): δ 8.08-7.97 (m,1H), 6.82-6.74 (m, 1H), 6.66 (d, 1H, ^{4}J = 2.7 Hz), 6.26 (s, 1H), 3.85 (s, 3H), 3.01-2.98 (m, 4H), 1.82-1.69 (m, 4H). ^{19}F NMR ($CDCl_3$, 376.5 MHz): δ -65.9 (s, 3F). *E*- isomer: ^{1}H NMR ($CDCl_3$, 400.1 MHz): δ 6.73 (d, 1H, ^{4}J = 2.6 Hz), 5.80 (s, 1H), 3.84 (s, 3H), 3.26-3.23 (m, 4H), 1.99-1.88 (m, 4H). Other signals are overlapped with those of major isomer. ^{19}F NMR ($CDCl_3$, 376.5 MHz): δ -59.7 (s, 3F). NMR data of enamine **3c** are in agreement with those in the literature.[6]

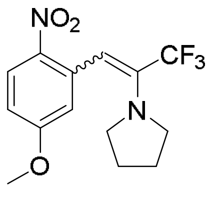

1-[(1*Z*)-(4-nitro-3-(3,3,3-trifluoro-2-(pyrrolidin-1-yl)prop-1-en-1-yl)phenyl]pyrrolidine (3n). Obtained from styrenes **2n** (0.396 g, 1.469 mmol) or from styrene **2o** (0.286 g, 1 mmol). Yellow orange solid, mp 145-147 °C, yield 0.255 g (90% from **2n**), 0.468 g (72% from **2o**). Mixture of *Z*/*E* isomers (84:16; ^{19}F). For the mixture of isomers: *Z*-isomer: ^{1}H NMR ($CDCl_3$, 400.1 MHz): δ 8.07 (d, 1H, ^{3}J = 9.3 Hz), 6.39-6.33 (m, 2H), 6.20 (d, 1H, ^{4}J = 2.4 Hz), 3.39-3.30 (m, 4H), 3.02 (t, 4H, ^{3}J = 6.4 Hz), 2.11-2.02 (m, 4H), 1.69-1.80 (m, 4H). $^{13}C\{^{1}H\}$ NMR ($CDCl_3$, 100.6 MHz): δ 150.2, 135.8, 135.0, 133.9 (q, $^{2}J_{CF}$ = 28.7 Hz), 127.7, 121.9 (q, $^{1}J_{CF}$ = 277.8 Hz), 112.9, 109.21, 104.1 (q, $^{3}J_{CF}$ = 6.8 Hz), 50.4 (d, $^{4}J_{CF}$ = 1.1 Hz), 47.7, 25.4, 25.3. ^{19}F NMR

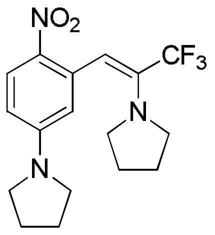

($CDCl_3$, 376.5 MHz): δ -65.8 (s, 3F). *E*- isomer: ^{1}H NMR ($CDCl_3$, 400.1 MHz): δ 8.08 (d, 1H, ^3J = 9.3 Hz), 5.97 (s, 1H), 6.27 (d, 1H, ^4J = 2.4 Hz), 3.24 (t, 4H, ^3J = 6.5 Hz), 1.97-1.90 (m, 4H). Other signals are overlapped with those of major isomer. $^{13}C\{^1H\}$ NMR ($CDCl_3$, 100.6 MHz): δ 135.5 (q, $^2J_{CF}$ = 27.3 Hz), 127.6, 113.8 (q, *J* = 3.4, $\underline{C}H=CCF_3$), 109.24, 106.2 (q, $^3J_{CF}$ = 3.4 Hz), 49.30 (d, $^4J_{CF}$ = 1.1 Hz), 47.6, 24.6. Other signals are overlapped with those of major isomer or can not be seen in the spectrum due to the low concentration of minor isomer. ^{19}F NMR ($CDCl_3$, 376.5 MHz): δ -59.2 (s, 3F). HRMS (ESI-TOF): m/z $[M+H]^+$ Calcd for $C_{17}H_{21}F_3N_3O_2^+$: 356.1580; found: 356.1581.

Synthesis of indoles 4 by the reduction of nitro-substituted enamines 3 (general procedure IV). A one neck 25 mL round bottomed flask was charged with enamine 3 (0.5 mmol), glacial acetic acid (2 mL), water (0.2 mL) and Fe powder (0.112 g, 2 mmol). Reaction mixture was kept at 80 °C under stirring for 1-2 hours until dissolving of Fe powder. Volatiles were evaporated in vacuo, the residue was suspended in CH_2Cl_2 (2-5 mL) and transferred on the short silica gel pad. The product was isolated using appropriate mixture of hexane and CH_2Cl_2 (3:1 for **4a**, **4d**); and mixture of CH_2Cl_2 and MeOH (100:1 for **4o**) as eluents.

Multi-gramm scale synthesis of indole 4a. A one neck 250 mL round bottomed flask was charged with enamine **3a** (7.01 g, 24.5 mmol), glacial acetic acid (100 mL), water (20 mL) and Fe powder (5.49 g, 98 mmol). Reaction mixture was kept at 80-90 °C under stirring for 2 hours until dissolving of Fe powder. The reaction mixture was poured into water (1000 mL), the precipitate formed was filtered off and washed by water (100 mL). Next, precipate was washed with CH_2Cl_2 (2x50 mL), organic phase was dried over Na_2SO_4 and evaporated *in vacuo* to give pure indole **4a** as colourless plates.

One pot synthesis of indoles 4 from styrenes 2 (general procedure V). A one neck 25 mL round bottomed flask was charged with pyrrolidine (1 mL, 11.8 mmol) and corresponding styrene **2** (0.5 mmol) was added in one portion with vigorous stirring. The reaction mixture was stirred at room temperature for 1-3 h until all starting styrene was consumed (TLC or NMR monitoring). The excess of pyrrolidine was evaporated in vacuum and the viscous residue was dissolved in glacial acetic acid (2 mL) and water (0.2 mL). After that Fe powder (0.112 g, 2 mmol) was added and the reaction mixture was kept at 80 °C under stirring for 1-2 hours until dissolving of Fe powder. Volatiles were evaporated in vacuo, the residue was suspended in CH_2Cl_2 (2-5 mL) and transferred

on the short silica gel pad. The product was isolated using appropriate mixtures of hexane and CH_2Cl_2 (3:1 for **4b**,**4c**,**4e**,**4k**,**4n**; 1:1 for **4f**,**4g**,**4h**,**4i**); CH_2Cl_2 (for **4l**,**4m**) and mixture of CH_2Cl_2 and MeOH (100:1 for **4j**,**10a**) as eluents.

2-(Trifluoromethyl)-1*H*-indole (4a). Obtained from enamine **3a** (0.107 g, 0.374 mmol) by procedure IV. White crystals, m.p. 111-112 °C, yield 0.059 g (85%). ^{1}H NMR ($CDCl_3$, 400.1 MHz): δ 8.32 (br.s, 1H), 7.71 (d, 1H, ^{3}J = 8.0 Hz), 7.42 (dd, 1H, ^{3}J = 8.3 Hz, ^{4}J = 0.9 Hz), 7.39-7.32 (m, 1H), 7.25-7.21 (m, 1H), 6.96 (*pseudo*-dt, 1H, ^{4}J ~ 2.1 Hz, ^{4}J ~ 1.1 Hz). ^{13}C{^{1}H} NMR ($CDCl_3$, 100.6 MHz): δ 136.1, 126.6, 125.7 (q, $^{2}J_{CF}$ = 39.2 Hz), 124.8, 122.1, 121.2 (q, $^{1}J_{CF}$ = 267.7 Hz), 121.1, 111.7, 104.3 (q, $^{3}J_{CF}$ = 3.2 Hz). ^{19}F NMR ($CDCl_3$, 376.5 MHz): δ -62.6 (d, 3F, ^{4}J = 1.1 Hz). NMR data of indole **4a** are in agreement with those in the literature.[7]

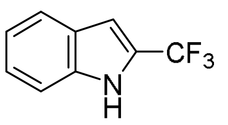

6-Chloro-2-(trifluoromethyl)-1*H*-indole (4b). Obtained from styrene **2b** (0.100 g, 0.35 mmol) by procedure V. Slightly yellow oil, yield 0.035 g (48%). ^{1}H NMR ($CDCl_3$, 400.1 MHz): δ 8.35 (br.s, 1H), 7.58 (d, 1H, ^{3}J = 8.5 Hz), 7.40 (*pseudo*-t, 1H, ^{4}J ~ 0.9 Hz), 7.17 (dd, 1H, ^{3}J = 8.5 Hz, ^{4}J = 1.8 Hz), 6.91-6.89 (m, 1H). ^{13}C{^{1}H} NMR ($CDCl_3$, 100.6 MHz): δ 136.4, 130.7, 126.4 (q, $^{2}J_{CF}$ = 39.4 Hz), 125.1, 123.0, 122.2, 120.9 (q, $^{1}J_{CF}$ = 267.8 Hz), 111.6, 104.3 (q, $^{3}J_{CF}$ = 3.4 Hz). ^{19}F NMR ($CDCl_3$, 376.5 MHz): δ -61.9 (d, 3F, ^{4}J = 1.1 Hz). NMR data of indole **4b** are in agreement with those in the literature.[7]

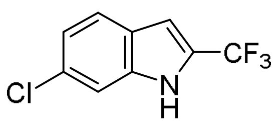

7-Methoxy-2-(trifluoromethyl)-1*H*-indole (4c). Obtained from styrene **2c** (0.149 g, 0.53 mmol) by procedure V. Colourless oil, yield 0.058 g (51%). ^{1}H NMR ($CDCl_3$, 400.1 MHz): δ 8.68 (br.s, 1H), 7.29 (d, 1H, ^{3}J = 8.1 Hz), 7.13 (t, 1H, ^{3}J = 7.9 Hz), 6.92 (*pseudo*-d, 1H, ^{4}J ~ 1.0 Hz), 6.76 (d, 1H, ^{3}J = 7.7 Hz), 3.99 (s, 3H). ^{13}C{^{1}H} NMR ($CDCl_3$, 100.6 MHz): δ 146.3, 127.8, 127.1, 125.4 (q, $^{2}J_{CF}$ = 39.3 Hz), 121.6, 121.2 (q, $^{1}J_{CF}$ = 267.6 Hz), 114.3, 104.4 (q, $^{3}J_{CF}$ = 3.2 Hz), 103.8, 55.4. ^{19}F NMR ($CDCl_3$, 376.5 MHz): δ -61.5 (d, 3F, ^{4}J = 1.0 Hz). NMR data of indole **4c** are in agreement with those in the literature.[7]

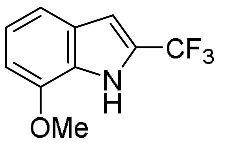

5-Methoxy-2-(trifluoromethyl)-1*H*-indole (4d). Obtained from enamine **3d** (0.088 g, 0.28 mmol) by procedure IV. Colourless crystals, m.p. 48-49 °C, yield 0.0382 g (64%). ^{1}H NMR ($CDCl_3$, 400.1 MHz): δ 8.35 (br.s, 1H), 7.30 (d, 1H, ^{3}J = 8.9 Hz), 7.10 (d, 1H, ^{4}J = 2.4 Hz), 7.00 (dd, 1H, ^{3}J = 8.9 Hz, ^{4}J = 2.4 Hz), 6.88-6.82 (m, 1H), 3.86 (s, 3H). ^{13}C{^{1}H} NMR ($CDCl_3$, 100.6 MHz): δ 154.9, 131.3, 127.1, 126.1 (q, $^{2}J_{CF}$ = 39.3 Hz),

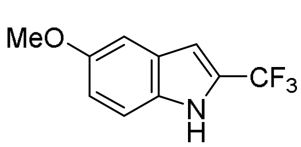

121.2 (q, $^{1}J_{CF}$ = 267.6 Hz), 115.8, 112.6, 103.9 (q, $^{3}J_{CF}$ = 3.3 Hz), 102.7, 55.7. ^{19}F NMR ($CDCl_3$, 376.5 MHz): δ -61.6 (d, 3F, ^{4}J = 0.9 Hz). NMR data of indole **4d** are in agreement with those in the literature.[7]

5,7-Dimethyl-2-(trifluoromethyl)-1*H*-indole (4e). Obtained from styrene **2e** (0.109 g, 0.391 mmol) by procedure V. Slightly yellow oil, yield 0.036 g (43%). ^{1}H NMR ($CDCl_3$, 400.1 MHz): δ 8.16 (br.s, 1H), 7.30 (s, 1H), 6.96 (s, 1H), 6.88-6.82 (m, 1H), 2.48 (s, 3H), 2.42 (s, 3H). ^{13}C{^{1}H} NMR ($CDCl_3$, 100.6 MHz): δ 134.3, 130.7, 127.0, 126.5, 125.4 (q, $^{2}J_{CF}$ = 38.9 Hz), 121.4 (q, $^{1}J_{CF}$ = 267.4 Hz), 120.6, 119.0, 104.3 (q, $^{3}J_{CF}$ = 3.4 Hz), 21.3, 16.5. ^{19}F NMR ($CDCl_3$, 376.5 MHz): δ -61.6 (d, 3F, ^{4}J = 1.0 Hz). HRMS (ESI-TOF): m/z [M-H]$^-$ Calcd for $C_{11}H_9F_3N^-$: 212.0693; found: 212.0690.

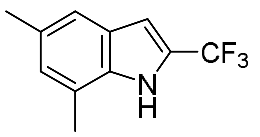

7-(Trifluoromethyl)-2,3-dihydro-6*H*-[1,4]dioxino[2,3-*f*]-indole (4f). Obtained from styrene **2f** (0.154 g, 0.497 mmol) by procedure V. White powder, m.p. 136-138 °C, yield 0.098 g (81%). ^{1}H NMR ($CDCl_3$, 400.1 MHz): δ 8.24 (br.s, 1H), 7.13 (s, 1H), 6.87 (s, 1H), 6.77 (s, 1H), 4.28 (q, 4H, ^{3}J = 5.2 Hz). ^{13}C{^{1}H} NMR ($CDCl_3$, 100.6 MHz): δ 143.1, 140.1, 131.7, 125.5 (q, $^{2}J_{CF}$ = 38.9 Hz), 121.2 (q, $^{1}J_{CF}$ = 267.3 Hz), 121.0, 107.9, 103.8 (q, $^{3}J_{CF}$ = 3.4 Hz), 98.6, 64.5, 64.1. ^{19}F NMR ($CDCl_3$, 376.5 MHz): δ -61.5 (s, 3F). HRMS (ESI-TOF): m/z [M-H]$^-$ Calcd for $C_{11}H_7F_3NO_2^-$: 242.0434; found: 242.0437.

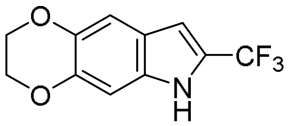

4,7-Dimethoxy-2-(trifluoromethyl)-1*H*-indole (4g). Obtained from styrene **2g** (0.107 g, 0.309 mmol) by procedure V. Light beige crystals, m.p. 74-76 °C, yield 0.053 g (70%). ^{1}H NMR ($CDCl_3$, 400.1 MHz): δ 8.73 (br.s, 1H), 7.05-7.01 (m, 1H), 6.62 (d, 1H, ^{3}J = 8.3 Hz), 6.42 (d, 1H, ^{3}J = 8.3 Hz), 3.92 (s, 3H), 3.91 (s, 3H). ^{13}C{^{1}H} NMR ($CDCl_3$, 100.6 MHz): δ 148.2, 140.9, 128.2, 124.3 (q, $^{2}J_{CF}$ = 39.5 Hz), 121.2 (q, $^{1}J_{CF}$ = 267.5 Hz), 119.0, 103.9, 102.2 (q, $^{3}J_{CF}$ = 3.3 Hz), 99.6, 55.7, 55.6. ^{19}F NMR ($CDCl_3$, 376.5 MHz): δ -61.4 (d, 3F, ^{4}J = 0.9 Hz). HRMS (ESI-TOF): m/z [M]$^+$ Calcd for $C_{11}H_{10}F_3NO_2^+$: 245.0658; found: 245.0667.

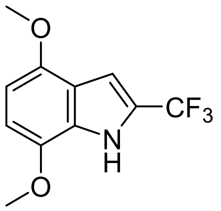

5,6-Dimethoxy-2-(trifluoromethyl)-1*H*-indole (4h). Obtained from styrene **2h** (0.129 g, 0.416 mmol) by procedure V. White crystals, m.p. 89-90 °C, yield 0.055 g (54%). ^{1}H NMR ($CDCl_3$, 400.1 MHz): δ 8.46 (br.s, 1H), 7.06 (s, 1H), 6.83 (s, 1H), 6.82-6.78 (m, 1H), 3.91 (s, 3H), 3.87 (s, 3H). ^{13}C{^{1}H} NMR ($CDCl_3$, 100.6 MHz): δ 149.1, 146.0, 130.9, 124.0 (q, $^{2}J_{CF}$ = 38.9 Hz), 121.3 (q, $^{1}J_{CF}$ = 267.0 Hz), 119.3, 104.0 (q, $^{3}J_{CF}$ = 3.4 Hz),

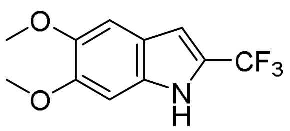

102.5, 94.1, 56.1, 56.0. ^{19}F NMR ($CDCl_3$, 376.5 MHz): δ -61.2 (d, 3F, ^{4}J = 1.1 Hz). NMR data of indole **4h** are in agreement with those in the literature.[7]

6-(Trifluoromethyl)-5*H*-[1,3]dioxolo[4.5-*f*]-indole (4i). Obtained from styrene **2i** (0.125 g, 0.38

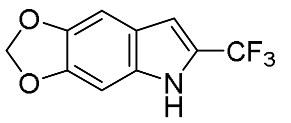

mmol) by procedure V. White crystals, m.p. 113-115 °C, yield 0.022 g (25%). ^{1}H NMR ($CDCl_3$, 400.1 MHz): δ 8.26 (br.s, 1H), 6.99 (s, 1H), 6.83 (s, 1H), 6.80-6.74 (m, 1H), 5.97 (s, 2H). ^{13}C{^{1}H} NMR ($CDCl_3$, 100.6 MHz): δ 147.1, 144.1, 131.4, 124.1 (q, $^{2}J_{CF}$ = 39.2 Hz), 121.2 (q, $^{1}J_{CF}$ = 267.0 Hz), 120.6, 104.5 (q, $^{3}J_{CF}$ = 3.3 Hz), 101.0, 99.8, 92.0. ^{19}F NMR ($CDCl_3$, 376.5 MHz): δ -61.3 (d, 3F, ^{4}J = 1.1 Hz). NMR data of indole **4i** are in agreement with those in the literature.[8]

2-(Trifluoromethyl)-1*H*-indole-6-amine (4j). Obtained from styrene **2j** (0.293 g, 0.99 mmol) by

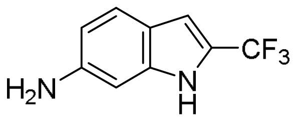

procedure V. 8 Equivalents of Fe (0.448 g, 8 mmol) was used due to the presence of second nitro-group in the styrene **2j**. Beige crystals, m.p. 124-126 °C, yield 0.119 g (60%). ^{1}H NMR ($CDCl_3$, 400.1 MHz): δ 9.56 (br.s, 1H), 7.37 (d, 1H, ^{3}J = 8.5 Hz), 6.77 (s, 1H), 6.65 (s, 1H), 6.58 (dd, 1H, ^{3}J = 8.5 Hz, ^{4}J = 1.9 Hz), 4.21 (br.s, 2H). ^{13}C{^{1}H} NMR ($CDCl_3$, 100.6 MHz): δ 146.9, 139.5, 123.2 (q, $^{2}J_{CF}$ = 38.7 Hz), 123.1, 122.8 (q, $^{1}J_{CF}$ = 265.7 Hz), 119.6, 112.8, 104.7 (q, $^{3}J_{CF}$ = 3.6 Hz). ^{19}F NMR ($CDCl_3$, 376.5 MHz): δ -59.1 (d, 3F, ^{4}J = 1.1 Hz). NMR data of indole **4j** are in agreement with those in the literature.[7]

2,6-Bis(trifluoromethyl)-1*H*-indole (4k). Obtained from styrene **2k** (0.240 g, 0.75 mmol) by

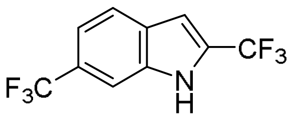

procedure V. Yellow crystals, m.p. 46-47 °C, yield 0.0896 g (47%). ^{1}H NMR ($CDCl_3$, 400.1 MHz): δ 8.61 (br.s, 1H), 7.76 (d, 1H, ^{3}J = 8.4 Hz), 7.71 (s, 1H), 7.43 (d, 1H, ^{3}J = 8.4 Hz), 6.98 (s, 1H). ^{13}C{^{1}H} NMR ($CDCl_3$, 100.6 MHz): δ 134.9, 128.8, 128.3 (q, $^{2}J_{CF}$ = 39.6 Hz), 127.0 (q, $^{2}J_{CF}$ = 32.2 Hz), 124.6 (q, $^{1}J_{CF}$ = 271.9 Hz), 122.7, 120.8 (q, $^{1}J_{CF}$ = 268.2 Hz), 117.9 (q, $^{3}J_{CF}$ = 3.3 Hz), 109.4 (q, $^{3}J_{CF}$ = 4.5 Hz), 104.3 (q, $^{3}J_{CF}$ = 3.1 Hz). ^{19}F NMR ($CDCl_3$, 376.5 MHz): δ -62.0 (s, 3F), -62.3 (s, 3F). NMR data of indol **4k** are in agreement with those in the literature.[7]

2-(Trifluoromethyl)-1*H*-indole-6-carbonitril (4l). Obtained from styrene **2l** (0.080 g, 0.291

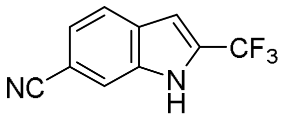

mmol) by procedure V. Slightly brown solid, m.p. 112-114 °C, yield 0.0305 g (50%). ^{1}H NMR ($CDCl_3$, 400.1 MHz): δ 9.18 (br.s, 1H), 7.85 (*pseudo*-d, 1H, ^{4}J ~ 1.1 Hz), 7.77 (d, 1H, ^{3}J = 8.3 Hz), 7.43 (dd, 1H, ^{3}J = 8.3 Hz, ^{4}J = 1.3 Hz), 6.99 (*pseudo*-dt, 1H, ^{4}J ~ 2.1 Hz, ^{4}J ~ 1.0 Hz). ^{13}C{^{1}H} NMR ($CDCl_3$, 100.6 MHz): δ 134.9, 129.7, 129.5 (q, $^{2}J_{CF}$ = 39.2 Hz), 123.7, 123.1,

120.6 (q, $^1J_{CF}$ = 268.6 Hz), 119.8, 117.0, 107.2, 104.4 (q, $^3J_{CF}$ = 3.2 Hz). ^{19}F NMR ($CDCl_3$, 376.5 MHz): δ -62.2 (d, 3F, ^4J = 0.9 Hz). HRMS (ESI-TOF): m/z [M-H]$^-$ Calcd for $C_{10}H_4F_3N_2^-$: 209.0332; found: 209.0323.

Methyl 2-(trifluoromethyl)-1*H*-indole-6-carboxylate (4m). Obtained from styrene **2m** (0.126 g, 0.408 mmol) by procedure V. Pale brown solid, yield 0.0525 g (53%). ^{1}H NMR ($CDCl_3$, 400.1 MHz): δ 10.38 (br.s, 1H), 8.15 (*pseudo*-d, 1H, ^4J ~ 0.5 Hz), 7.78 (dd, 1H, ^3J = 8.5 Hz, ^4J = 1.4 Hz), 7.73 (d, 1H, ^3J = 8.4 Hz), 7.08-6.91 (m, 1H), 3.89 (s, 3H). $^{13}C\{^1H\}$ NMR ($CDCl_3$, 100.6 MHz): δ 168.0, 136.9, 130.7, 129.3 (q, $^2J_{CF}$ = 38.8 Hz), 127.3, 122.7, 122.2 (q, $^1J_{CF}$ = 267.4 Hz), 122.1, 115.1, 104.5 (q, $^3J_{CF}$ = 3.5 Hz), 52.7. ^{19}F NMR ($CDCl_3$, 376.5 MHz): δ -60.0 (d, 3F, ^4J = 0.9 Hz). NMR data of indole **4m** are in agreement with those in the literature.[7]

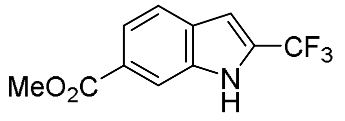

5-Cloro-2-(trifluoromethyl)-1*H*-indole (4n). Obtained from styrene **2n** (0.083 g, 0.29 mmol) by procedure V (piperidine was used instead of pyrrolidine). Pale yellow crystals, m.p. 59-61 °C, yield 0.0327 g (71%). ^{1}H NMR ($CDCl_3$, 400.1 MHz): δ 8.45 (br.s, 1H), 7.65 (d, 1H, ^4J = 1.9 Hz), 7.34 (d, 1H, ^3J = 8.8 Hz), 7.28 (dd, 1H, ^3J = 8.8 Hz, ^4J = 1.9 Hz), 6.90-6.83 (m, 1H). $^{13}C\{^1H\}$ NMR ($CDCl_3$, 100.6 MHz): δ 134.4, 127.6, 127.0 (q, $^2J_{CF}$ = 39.2 Hz), 126.9, 125.3, 121.4, 120.9 (q, $^1J_{CF}$ = 268.0 Hz), 112.8, 103.8 (q, $^3J_{CF}$ = 3.4 Hz). ^{19}F NMR ($CDCl_3$, 376.5 MHz): δ -61.9 (d, 3F, ^4J = 1.0 Hz). NMR data of indole **4n** are in agreement with those in the literature.[9]

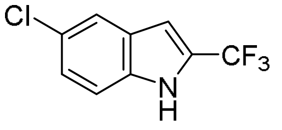

5-(Pyrrolidin-1-yl)-2-(trifluoromethyl)-1*H*-indole (10a). Obtained from enamine **3n** (0.160 g, 0.45 mmol) by procedure V. Orange crystals, m.p. 130-131 °C, yield 0.052 g (45%). ^{1}H NMR ($CDCl_3$, 400.1 MHz): δ 8.11 (br.s, 1H), 7.26 (d, 1H, ^3J = 9.1 Hz), 6.86-6.70 (m, 3H), 3.32 (t, 4H, ^3J = 6.6 Hz), 2.09-2.00 (m, 4H). $^{13}C\{^1H\}$ NMR ($CDCl_3$, 100.6 MHz): δ 143.9, 129.4, 127.9, 125.6 (q, $^2J_{CF}$ = 38.5 Hz), 121.4 (q, $^1J_{CF}$ = 267.5 Hz), 113.2, 112.1, 103.2 (q, $^3J_{CF}$ = 3.3 Hz), 101.7, 48.6, 25.3. ^{19}F NMR ($CDCl_3$, 376.5 MHz): δ -61.5 (s, 3F). HRMS (ESI-TOF): m/z [M+H]$^+$ Calcd for $C_{13}H_{14}F_3N_2^+$: 255.1104; found: 255.1109.

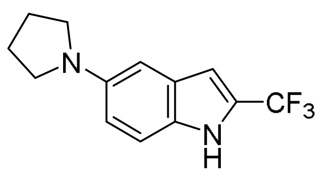

One pot synthesis of indoles 10 from styrenes 2 (general procedure VI). A 4 mL vial with a screw cup was charged with corresponding amine (5 mmol) and styrene **2o** (0.5 mmol). The reaction mixture was heated at appropriate temperature for several hours (see further) or at room temperature (for $MeNH_2$) until starting styrene was consumed (TLC or NMR monitoring). The

excess of amine was evaporated in vacuo, the viscous residue was dissolved in glacial acetic acid (2 mL) and transferred into a one neck 25 mL round bottomed flask. Next, water (0.2 mL), Fe powder (0.112 g, 2 mmol) was added and the reaction mixture was kept at 80 °C at stirring for 1-2 hours until dissolving of Fe powder. Volatiles were evaporated in vacuo, the residue was suspended in CH_2Cl_2 (2-5 mL) and filtered through a short celite pad. The filtrate was evaporated and the residue was purified by coloumn chromatography on silica gel using appropriate mixtures of CH_2Cl_2 and MeOH (100:1 and 30:1) as eluents.

5-(Piperidin-1-yl)-2-(trifluoromethyl)-1*H*-indole (10b). Obtained styrene **2o** (0.109 g, 0.404 mmol) and piperidine (0.572 g) by heating at 90 °C for 3 h. Pale green-brown solid, m.p. 104-106 °C, yield 0.048 g (44%). ^{1}H NMR ($CDCl_3$, 400.1 MHz): δ 8.46 (br.s, 1H), 7.25 (d, 1H, ^3J = 8.9 Hz), 7.17 (*pseudo*-d, 1H, ^4J ~ 2.1 Hz), 7.12 (dd, 1H, ^3J = 8.9 Hz, ^4J = 2.3 Hz), 6.82 (br.s, 1H), 3.14-3.07 (m, 4H), 1.77 (dt, 4H, ^3J = 11.3 Hz, ^3J = 5.7 Hz), 1.62-1.54 (m, 2H). $^{13}C\{^1H\}$ NMR ($CDCl_3$, 100.6 MHz): δ 147.9, 131.5, 127.2, 125.8 (q, $^2J_{CF}$ = 38.8 Hz), 121.3 (q, $^1J_{CF}$ = 267.6 Hz), 119.5, 112.1, 108.4, 103.9 (q, $^3J_{CF}$ = 3.4 Hz), 53.1, 26.2, 24.2. ^{19}F NMR ($CDCl_3$, 376.5 MHz): δ -61.4 (s, 3F). HRMS (ESI-TOF): m/z [M+H]$^+$ Calcd for $C_{14}H_{16}F_3N_2^+$: 269.1260; found: 269.1265.

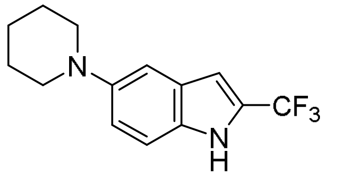

4-(2-(Trifluoromethyl)-1*H*-indol-5yl)morpholine (10c). Obtained from styrene **2o** (0.104 g, 0.385 mmol) and morpholine (0.530 g) by heating at 100 °C for 4 h. Pale green-brown solid, m.p. 167-169 °C, yield 0.061 g (59%). ^{1}H NMR ($CDCl_3$, 400.1 MHz): δ 9.91 (br.s, 1H), 7.42-7.36 (m, 1H), 7.12-7.07 (m, 2H), 6.84 (*pseudo*-dt, 1 H, ^4J ~ 2.1 Hz, ^4J ~ 1.0 Hz), 3.84-3.75 (m, 4H), 3.10-3.01 (m, 4H). $^{13}C\{^1H\}$ NMR ($CDCl_3$, 100.6 MHz): δ 147.8, 133.0, 127.9, 126.3 (q, $^2J_{CF}$ = 38.6 Hz), 122.6 (q, $^1J_{CF}$ = 266.5 Hz), 118.9, 113.5, 107.8, 104.1 (q, $^3J_{CF}$ = 3.4 Hz), 67.6, 52.1. ^{19}F NMR ($CDCl_3$, 376.5 MHz): δ -59.5 (d, 3F, ^4J = 1.0 Hz). HRMS (ESI-TOF): m/z [M+H]$^+$ Calcd for $C_{13}H_{14}F_3N_2O^+$: 271.1053; found: 271.1057.

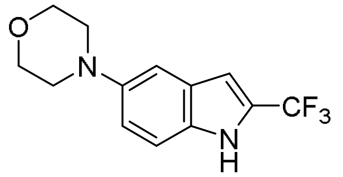

5-(Azepan-1-yl)-2-(trifluoromethyl)-1*H*-indole (10d). Obtained from styrene **2o** (0.107 g, 0.396 mmol) and hexamethyleneimine (0.480 g) by heating at 100 °C for 4 h. Pale yellow-brown solid, m.p. 65-67 °C, yield 0.060 g (54%). ^{1}H NMR ($CDCl_3$, 400.1 MHz): δ 8.12 (br.s, 1H), 7.23 (d, 1H, ^3J = 9.0 Hz), 6.92 (dd, 1H, ^3J = 9.0 Hz, ^4J = 2.4 Hz), 6.88 (*pseudo*-d, 1 H, ^4J ~ 2.2 Hz), 6.79 (br.s, 1H),

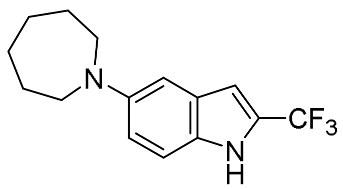

3.56-3.47 (m, 4H), 1.89-1.79 (m, 4H), 1.61-1.53 (m, 4H). $^{13}C\{^1H\}$ NMR ($CDCl_3$, 100.6 MHz): δ 144.6, 129.1, 128.0, 125.6 (q, $^2J_{CF}$ = 38.7 Hz), 121.4 (q, $^1J_{CF}$ = 267.4 Hz), 112.9, 112.2, 103.3 (q, $^3J_{CF}$ = 3.1 Hz), 101.5, 50.0, 27.9, 27.1. ^{19}F NMR ($CDCl_3$, 376.5 MHz): δ -61.5 (d, 3F, ^4J = 0.9 Hz). HRMS (ESI-TOF): m/z $[M+H]^+$ Calcd for $C_{15}H_{18}F_3N_2^+$: 283.1417; found: 283.1424.

***N,N*-Diethyl-2-(trifluoromethyl)-1*H*-indole-5-amine (10e)**. Obtained from styrene **2o** (0.101 g, 0.374 mmol) and diethylamine (0.480 g) by heating at 100 °C for 10 h. Pale brown oil, yield 0.041 g (43%). ^1H NMR ($CDCl_3$, 400.1 MHz): δ 8.29 (br.s, 1H), 7.26 (d, 1H, ^3J = 8.7 Hz), 7.02-6.93 (m, 2H), 6.79 (s, 1H), 3.33 (q, 4H, ^3J = 7.1 Hz), 1.13 (t, 6H, ^3J = 7.1 Hz). $^{13}C\{^1H\}$ NMR ($CDCl_3$, 100.6 MHz): δ 143.7, 130.3, 127.6, 125.7 (q, $^2J_{CF}$ = 38.9 Hz), 121.4 (q, $^1J_{CF}$ = 267.4 Hz), 116.4, 112.2, 106.0, 103.5 (q, $^3J_{CF}$ = 3.2 Hz), 45.9, 12.3. ^{19}F NMR ($CDCl_3$, 376.5 MHz): δ -61.6 (d, 3F, ^4J = 1.1 Hz). HRMS (ESI-TOF): m/z $[M+H]^+$ Calcd for $C_{13}H_{16}F_3N_2^+$: 257.1260; found: 257.1261.

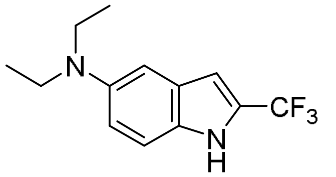

***N*-Methyl-2-(trifluoromethyl)-1*H*-indole-5-amine (10f)**. Obtained from styrene **2o** (0.116 g, 0.430 mmol) and *n*-methylamine (2 mL of 3.65 M solution in MeOH) by keeping the reaction mixture for 11 days. Pale green-brown solid, m.p. 133-135 °C, yield 0.040 g (44%). ^1H NMR (CD_3CN, 400.1 MHz): δ 9.74 (br.s, 1H), 7.26 (d, 1H, ^3J = 8.7 Hz), 6.80-6.68 (m, 3H), 2.77 (s, 3H). $^{13}C\{^1H\}$ NMR (CD_3CN, 100.6 MHz): δ 145.9, 131.4, 128.5, 125.7 (q, $^2J_{CF}$ = 38.5 Hz), 122.8 (q, $^1J_{CF}$ = 266.3 Hz), 116.2, 113.5, 103.4 (q, $^3J_{CF}$ = 3.4 Hz), 101.0, 31.4. ^{19}F NMR (CD_3CN, 376.5 MHz): δ -59.3 (d, 3F, ^4J = 0.9 Hz). HRMS (ESI-TOF): m/z $[M+H]^+$ Calcd for $C_{10}H_{10}F_3N_2^+$: 215.0791; found: 215.0792.

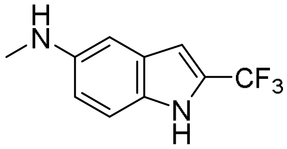

***N*-Hexyl-2-(trifluoromethyl)-1*H*-indole-5-amine (10g)**. Obtained from styrene **2o** (0.100 g, 0.370 mmol) and *n*-hexylamine (0.482 g) by heating at 100 °C for 4 h. Pale yellow-brown solid, m.p. 88-90 °C, yield 0.047 g (45%). ^1H NMR ($CDCl_3$, 400.1 MHz): δ 8.31 (br.s, 1H), 7.17 (d, 1H, ^3J = 8.8 Hz), 6.82 (d, 1H, ^4J = 2.1 Hz), 6.77-6.69 (m, 2H), 3.16-3.10 (m, 2H), 2.96 (br.s, 1H), 1.65 (dt, 2H, ^3J = 14.7 Hz, ^3J = 7.2 Hz), 1.48-1.29 (m, 6H), 0.91 (t, 3H, ^3J = 7.0 Hz). $^{13}C\{^1H\}$ NMR ($CDCl_3$, 100.6 MHz): δ 143.4, 130.3, 127.7, 125.6 (q, $^2J_{CF}$ = 38.6 Hz), 121.4 (q, $^1J_{CF}$ = 267.4 Hz), 115.4, 112.3, 103.3 (q, $^3J_{CF}$ = 3.3 Hz), 102.2, 45.1, 31.7, 29.5, 26.9, 22.6, 14.0. ^{19}F NMR ($CDCl_3$, 376.5 MHz): δ -61.5 (d, 3F, ^4J = 1.0 Hz). HRMS (ESI-TOF): m/z $[M+H]^+$ Calcd for $C_{15}H_{20}F_3N_2^+$: 285.1573; found: 285.1576.

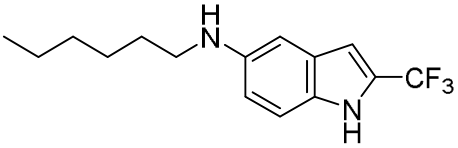

Reactions of indole 4a with electrophiles.

Synthesis of 2-(trifluoromethyl)-1*H*-indol-3-carbaldehyde (17). A 4 mL vial with a screw cup was charged with DMF (0.5 mL), cooled to -18 °C (in the fridge) and then $POCl_3$ (0.210 g, 1.37 mmol) was added. The reaction mixture was kept at 5-7 °C (in the fridge) for 30 min and then indole **4a** (0.108 g, 0.58 mmol). The reaction mixture was stirred for 6h at 80 °C, cooled down to room temperature and transferred to separating funnel with water (50 mL) using CH_2Cl_2 (30-40 mL). After shaking, organic phase was separated, water phase was extracted with CH_2Cl_2 (20 mL). Combined organic phase was washed with water (20 mL), and dried over Na_2SO_4. Volatiles were evaporated in vacuo, the residue formed was suspended in hexane-CH_2Cl_2 mixture (3:1, 2 mL). The precipate was filtred off and dried in vacuo to give pure X. Beige powder, m.p. 167-169 °C, yield 0.066 g (53%). ^{1}H NMR (DMSO-D$_6$, 400.1 MHz): δ 10.21 (*pseudo*-d, 1H, ^4J = 1.0 Hz), 8.23 (d, 1H, ^3J = 8.0 Hz), 7.58 (d, 1H, ^3J = 8.2 Hz), 7.43-7.37 (m, 1H), 7.36-7.28 (m, 1H). ^{13}C{^{1}H} NMR (DMSO-D$_6$, 100.6 MHz): δ 184.2 (q, $^4J_{CF}$ = 1.1 Hz), 134.4, 131.1 (q, $^2J_{CF}$ = 39.1 Hz), 125.7, 124.4, 123.8, 122.1, 120.9 (q, $^1J_{CF}$ = 270.7 Hz), 115.5 (q, $^3J_{CF}$ = 1.9 Hz), 113.2. ^{19}F NMR (DMSO-D$_6$, 376.5 MHz): δ -55.8 (d, 3F, ^4J = 0.8 Hz). NMR data of indole **7** are in agreement with those in the literature.[10]

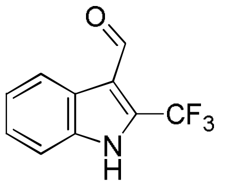

1-(2-(Trifluoromethyl)-1*H*-indol-3-yl)ethanone (18). A 8 mL vial with a screw cup was charged with 1,2-dichloroethane (1.5 mL), $AlCl_3$ (0.124 g, 0.93 mmol), cooled to -18 °C (in the fridge) and then AcCl (0.047 g, 0.60 mmol) was added. The reaction mixture was stirred at room temperature for 30 min and then indole **4a** (0.089 g, 0.48 mmol) was added. The reaction mixture was stirred overnight and poured into water (50 mL). Water phase was extracted with CH_2Cl_2 (3x20 mL). Combined organic phase was washed with water (20 mL), and dried over Na_2SO_4. Volatiles were evaporated in vacuo, the residue was purified by coloumn chromatography on silica gel using CH_2Cl_2 followed by mixture of CH_2Cl_2 and MeOH (100:1) as eluents. Beige powder, m.p. 125-127 °C, yield 0.070 g (64%). ^{1}H NMR (CD_3CN, 400.1 MHz): δ 10.77 (br.s, 1H), 8.11 (d, 1H, ^3J = 8.2 Hz), 7.60-7.56 (m, 1H), 7.41-7.36 (m, 1H), 7.35-7.30 (m, 1H), 2.66 (s, 3H). ^{13}C{^{1}H} NMR (DMSO-*d*$_6$, 100.6 MHz): δ 192.7, 134.8, 126.9 (q, $^2J_{CF}$ = 38.1 Hz), 125.4, 125.3, 124.8 (d, $^4J_{CF}$ = 3.0 Hz), 123.0, 121.9, 121.1 (q, $^1J_{CF}$ = 269.6 Hz), 116.9 (q,

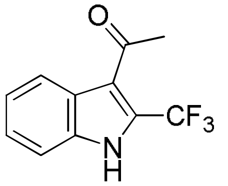

$^3J_{CF}$ = 1.5 Hz), 113.4 (d, $^3J_{CF}$ = 6.2 Hz), 31.0. ^{19}F NMR (CD_3CN, 376.5 MHz): δ -58.0 (s, 3F). HRMS (ESI-TOF): m/z $[M+H]^+$ Calcd for $C_{11}H_9F_3NO^+$: 228.0631; found: 228.0635.

(E)-1,1,1-Trifluoro-4-(2-(trifluoromethyl)-1H-indol-3-yl)but-3-en-2-one (20). A 8 mL vial with

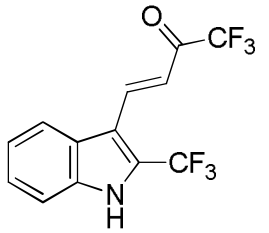

a screw cup was charged with indole **4a** (0.091 g, 0.49 mmol), (*E*)-4-ethoxy-1,1,1-trifluorobut-3-en-2-one **19** (0.090 g, 0.54 mmol), 1,2-dichloroethane (1 mL), and $BF_3·Et_2O$ (0.083 g, 0.059 mmol). The reaction mixture was stirred for 2h at 80 °C and poured into water (30 mL). Water phase was extracted with CH_2Cl_2 (3x20 mL). Combined organic phase was washed with water (20 mL), and dried over Na_2SO_4. Volatiles were evaporated in vacuo, the residue was purified by coloumn chromatography on silica gel using mixtures of hexane and CH_2Cl_2 (3:1 followed by 1:1) as eluents. Yellow powder, m.p. 125-127 °C, yield 0.0563 g (37%). ^{1}H NMR ($CDCl_3$, 400.1 MHz): δ 9.07 (br.s, 1H), 8.30 (d, 1H, ^3J = 15.9 Hz), 7.98 (d, 1H, ^3J = 8.0 Hz), 7.53 (d, 1H, ^3J = 8.0 Hz), 7.50-7.43 (m, 1H), 7.43-7.38 (m, 1H), 7.20 (d, 1H, ^3J = 15.9 Hz). $^{13}C\{^1H\}$ NMR ($CDCl_3$, 100.6 MHz): δ 180.1 (q, $^2J_{CF}$ = 35.1 Hz), 139.7, 135.3, 128.9 (q, $^2J_{CF}$ = 37.4 Hz), 126.2, 125.0, 123.7, 121.7, 120.7 (q, $^1J_{CF}$ = 270.5 Hz), 116.5 (q, $^1J_{CF}$ = 290.6 Hz), 116.6, 112.8, 112.7 (q, $^3J_{CF}$ = 2.3 Hz). ^{19}F NMR ($CDCl_3$, 376.5 MHz): δ -59.0 (d, 3F, ^4J = 0.8 Hz), -78.7 (s, 3F). HRMS (ESI-TOF): m/z $[M+H]^+$ Calcd for $C_{13}H_8F_6NO^+$: 308.0505; found: 308.0509.

Reactions of indole 4a with benzaldehydes in alcoholes under catalysis with $MeSO_3H$ (general procedure VII). A 4 mL vial with a screw cup was charged with indole **4a** (0.0925 g, 0.5 mmol), alcohol (MeOH or EtOH, 1 mL), corresponding benzaldehyde (0.6 mmol or 0.25 mmol for **23**) and $MeSO_3H$ (0.050g, 0.53 mmol). The reaction mixture was heated at 80 °C for appropriate time, volatiles were evaporated in vacuo, the residue was purified by coloumn chromatography on silica gel using mixtures of hexane and CH_2Cl_2 (3:1 followed by 1:1) as eluents.

3-(Methoxy(phenyl)methyl)-2-(trifluoromethyl)-1*H*-indole (21a). Obtained by the reaction of

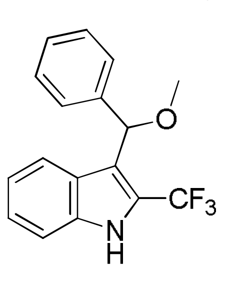

4a (0.0925 g, 0.5 mmol) with benzaldehyde (0.065 g, 0.6 mmol) in MeOH by heating for 8h. White crystals, m.p. 86-88 °C, yield 0.100 g (68%). ^{1}H NMR ($CDCl_3$, 400.1 MHz): δ 8.38 (br.s, 1H), 7.78 (d, 1H, ^3J = 8.2 Hz), 7.48 (d, 2H, ^3J = 7.4 Hz), 7.37 (d, 1H, ^3J = 8.3 Hz), 7.33-7.26 (m, 3H), 7.24-7.19 (m, 1H), 7.10 (ddd, 1H, ^3J = 8.1 Hz, ^3J = 7.0 Hz, ^4J = 1.0 Hz), 5.82 (s, 1H), 3.42 (s, 3H). ^{19}F NMR ($CDCl_3$, 376.5 MHz): δ -58.2 (s, 3F). NMR data of indole **21a** are in agreement with those in the literature.[6]

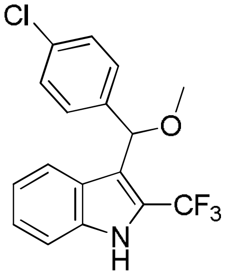

3-((4-Chlorophenyl)(methoxy)methyl)-2-(trifluoromethyl)-1H-indole (21b). Obtained by the reaction of **4a** (0.0925 g, 0.5 mmol) with 4-chlorobenzaldehyde (0.084 g, 0.6 mmol) in MeOH by heating for 10h. White crystals, m.p. 112-113 °C, yield 0.112 g (66%). ^{1}H NMR ($CDCl_3$, 400.1 MHz): δ 8.44 (br.s, 1H), 7.72 (d, 1H, ^3J = 8.1 Hz), 7.46-7.35 (m, 3H), 7.35-7.25 (m, 3H), 7.11 (ddd, 1H, ^3J = 8.1 Hz, ^3J = 7.0 Hz, ^4J = 1.0 Hz), 5.79 (s, 1H), 3.41 (s, 3H). ^{13}C{^{1}H} NMR ($CDCl_3$, 100.6 MHz): δ 139.8, 135.4, 133.0, 128.3, 127.7, 125.2, 125.1, 123.2 (q, $^2J_{CF}$ = 37.1 Hz), 122.7, 121.7 (q, $^1J_{CF}$ = 269.3 Hz), 121.2, 117.3 (q, $^3J_{CF}$ = 2.4 Hz), 111.7, 56.9. ^{19}F NMR ($CDCl_3$, 376.5 MHz): δ -58.2 (s, 3F). HRMS (ESI-TOF): m/z [M-MeO]$^-$ Calcd for $C_{16}H_{10}ClF_3N^+$: 308.0448; found: 308.0450.

3-(Methoxy(4-methoxyphenyl)methyl)-2-(trifluoromethyl)-1*H*-indole (21c). Obtained by the reaction of **4a** (0.098 g, 0.53 mmol) with 4-methoxybenzaldehyde (0.087 g, 0.636 mmol) in MeOH by heating for 12h. Pale brown powder, m.p. 138-140 °C, yield 0.092 g (52%). ^{1}H NMR ($CDCl_3$, 400.1 MHz): δ 8.43 (br.s, 1H), 7.80 (d, 1H, ^3J = 8.1 Hz), 7.40-7.34 (m, 3H), 7.31-7.26 (m, 1H), 7.10 (ddd, 1H, ^3J = 8.1 Hz, ^3J = 7.0 Hz, ^4J = 1.0 Hz), 6.83 (d, 2H, ^3J = 8.8 Hz), 5.77 (s, 1H), 3.76 (s, 3H), 3.39 (s, 3H). ^{19}F NMR ($CDCl_3$, 376.5 MHz): δ -58.3 (s, 3F). NMR data of indole **21c** are in agreement with those in the literature.[6]

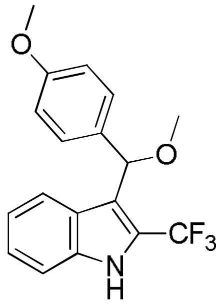

3-(Ethoxy(phenyl)methyl)-2-(trifluoromethyl)-1*H*-indole (22). Obtained by the reaction of **4a** (0.048 g, 0.259 mmol) with benzaldehyde (0.033 g, 0.306 mmol) in EtOH by heating for 8h. White crystals, m.p. 129-132 °C, yield 0.061 g (74%). ^{1}H NMR ($CDCl_3$, 400.1 MHz): δ 8.35 (br.s, 1H), 7.83 (d, 1H, ^3J = 8.2 Hz), 7.48 (d, 2H, ^3J = 7.3 Hz), 7.37 (d, 1H, ^3J = 8.3 Hz), 7.32-7.25 (m, 3H), 7.24-7.18 (m, 1H), 7.09 (ddd, 1H, ^3J = 8.1 Hz, ^3J = 7.0 Hz, ^4J = 1.0 Hz), 5.92 (s, 1H), 3.55 (qq, 2H, ^3J = 9.1 Hz, ^3J = 7.0 Hz), 1.26 (t, 3H, ^3J = 7.0 Hz). ^{19}F NMR ($CDCl_3$, 376.5 MHz): δ -58.2 (s, 3F). NMR data of indole **22** are in agreement with those in the literature.[6]

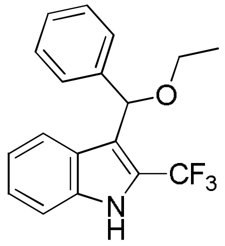

3,3'-(Phenylmethylene)bis(2-(trifluoromethyl)-1*H*-indole) (23). Obtained by the reaction of **4a** (0.087 g, 0.47 mmol) with benzaldehyde (0.026 g, 0.241 mmol) in EtOH by heating for 12h. Brown oil, yield 0.0486 g (45%). ^{1}H NMR ($CDCl_3$, 400.1 MHz):

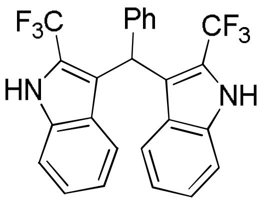

δ 8.41 (br.s, 2H), 7.39 (d, 2H, ^{3}J = 8.3 Hz), 7.27 (d, 2H, ^{4}J = 2.2 Hz), 7.25-7.16 (m, 5H), 6.84 (ddd, 2H, ^{3}J = 8.1 Hz, ^{3}J = 7.0 Hz, ^{4}J = 1.0 Hz), 6.72 (d, 2H, ^{3}J = 8.1 Hz), 6.54 (s, 1H). $^{13}C\{^{1}H\}$ NMR ($CDCl_3$, 100.6 MHz): δ 142.0, 135.0, 128.8, 128.3, 127.2, 126.8, 124.3, 122.4 (q, $^{2}J_{CF}$ = 37.5 Hz), 122.3, 121.7 (q, $^{1}J_{CF}$ = 269.6 Hz), 120.8, 118.8 (q, $^{3}J_{CF}$ = 1.5 Hz), 111,7, 38,0. ^{19}F NMR ($CDCl_3$, 376.5 MHz): δ -60.0 (s, 3F). HRMS (ESI-TOF): m/z $[M+H]^+$ Calcd for $C_{25}H_{17}F_6N_2^+$: 459.1290; found: 459.1290.

Structures and ^{19}F NMR data of reaction intermediates and by products

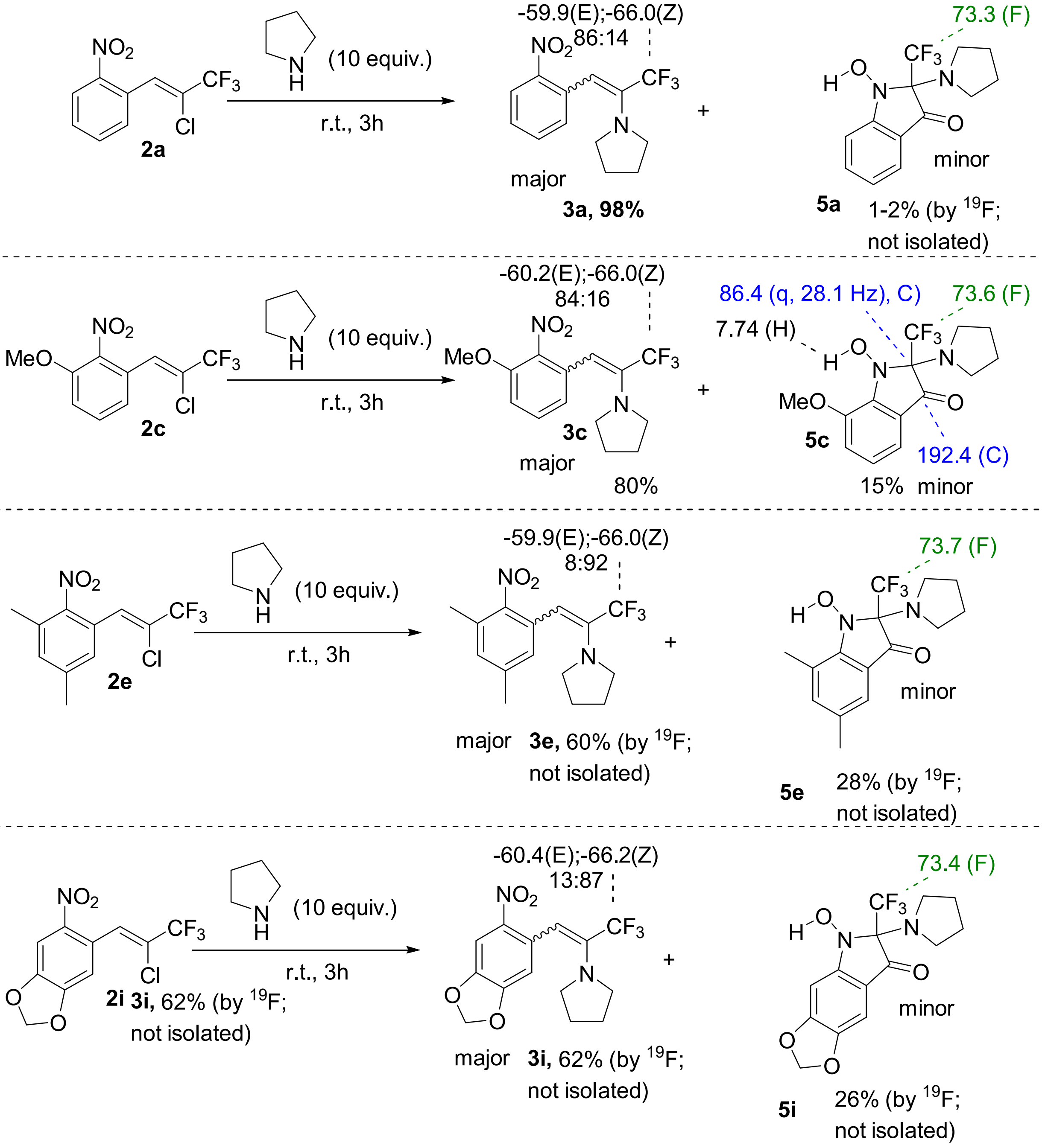

Scheme S2. Compositions of the reaction mixture in the synthesis of enamines **3**.

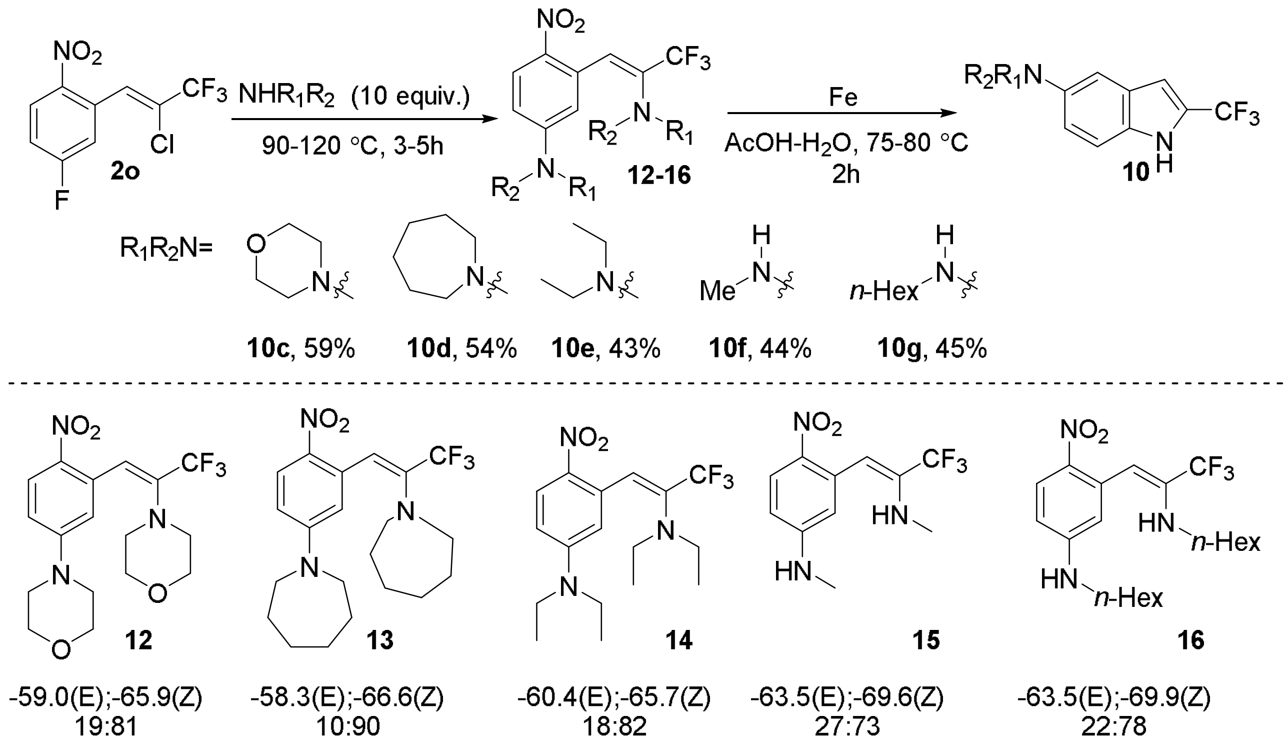

Scheme S3. Structure of enamines **12-16** in the synthesis of indoles **10**.

References

[1] Korotchenko, V.N.; Shastin, A.V.; Nenajdenko, V.G.; Balenkova, E.S. A novel approach to fluoro-containing alkenes. *Tetrahedron* **2001**, *57*, 7519-7527.

[2] Hirotaki, K.; Kawazoe, G.; Hanamoto, T. Facile synthesis of (*E*)-β-(trifluoromethyl)styrenes from halothane (HCFC-123B1). *J. Fluorine Chem.* **2015,** *171*, 169–173.

[3] Chen, M.-W.; Zhang, X.-G.; Zhong, P.; Hu, M.-L. Efficient One-Pot Synthesis of 2-Chloro-1,1, 1-trifluoro-2-alkenes Under Solvent-Free Conditions. *Synth. Commun*., **2009**, *39*, 756–763.

[4] Muzalevskiy, V.M.; Shastin, A.V.; Balenkova, E.S.; Nenajdenko, V.G. New approach to the synthesis of trifluoromethylvinyl sulfides *Russ. Chem. Bull.*, **2007**, *56*, 1526-1533.

[5] Muzalevskiy, V.M.; Nenajdenko, V.G.; Rulev, A.Yu.; Ushakov, I.A.; Romanenko, G.V.; Shastin, A.V.; Balenkova, E.S.; Haufe, G. Selective synthesis of α-trifluoromethyl-β-arylenamines or vinylogous guanidinium salts by treatment of β-halo-β-trifluoromethylstyrenes with secondary amines under different conditions. *Tetrahedron* **2009,** *65*, 6991–7000.

[6] Muzalevskiy, V.M.; Sizova, Z.A.; Nenajdenko, V.G. Modular Construction of Functionalized 2-CF_3-Indoles. *Org. Lett.*, **2021**, *23*, 5973–5977.

[7] Pedroni, J.; Cramer, N. 2-(Trifluoromethyl)indoles via Pd(0)-Catalyzed C(sp^3)-H Functionalization of Trifluoroacetimidoyl Chlorides. *Org. Lett.* **2016**, *18*, 1932-1935.

[8] Miyashita, K.; Kondoh, K.; Tsuchiya, K.; Miyabe, H.; Imanishi, T. Novel indole-ring formation by thermolysis of 2-(N-acylamino)-benzylphosphonium salts. Effective synthesis of 2-trifluoromethylindoles. *J. Chem. Soc., Perkin Trans. 1* **1996**, 1261-1268.

[9] Xie, J.-J.; Wang, Z.-Q.; Jiang, G.-F. Metal-free oxidative trifluoromethylation of indoles with CF_3SO_2Na on the C2 position. *RSC Adv.* **2019**, *9*, 35098-35101.

[10] Ye, Y.; Cheung, K.P.S.; He, L.; Tsui, G.C. Synthesis of 2-(Trifluoromethyl)indoles via Domino Trifluoromethylation/Cyclization of 2-Alkynylanilines. *Org. Lett.* **2018**, *20*, 1676-1679.

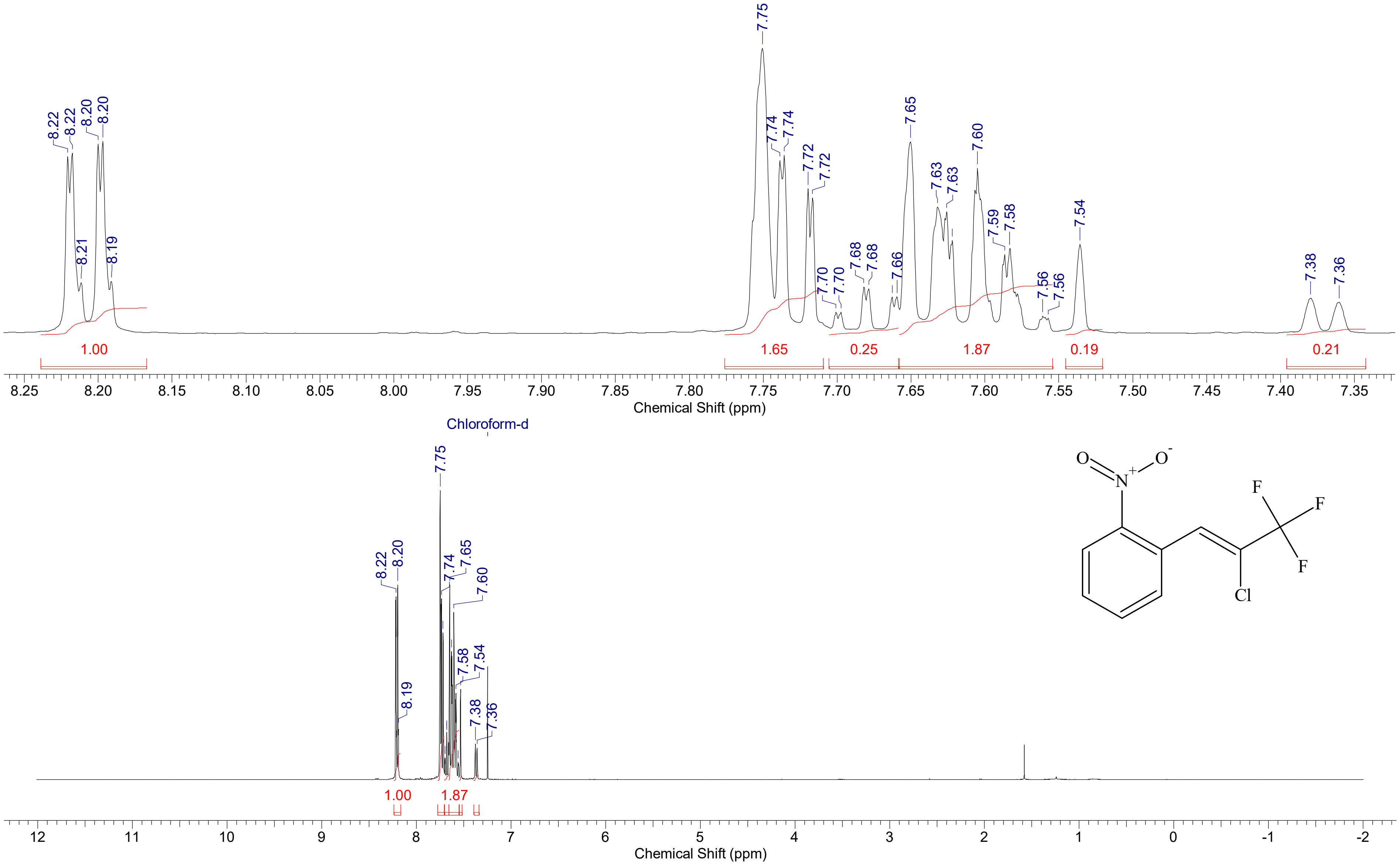

^{1}H NMR spectrum of **2a** (400.1 MHz, $CDCl_3$)

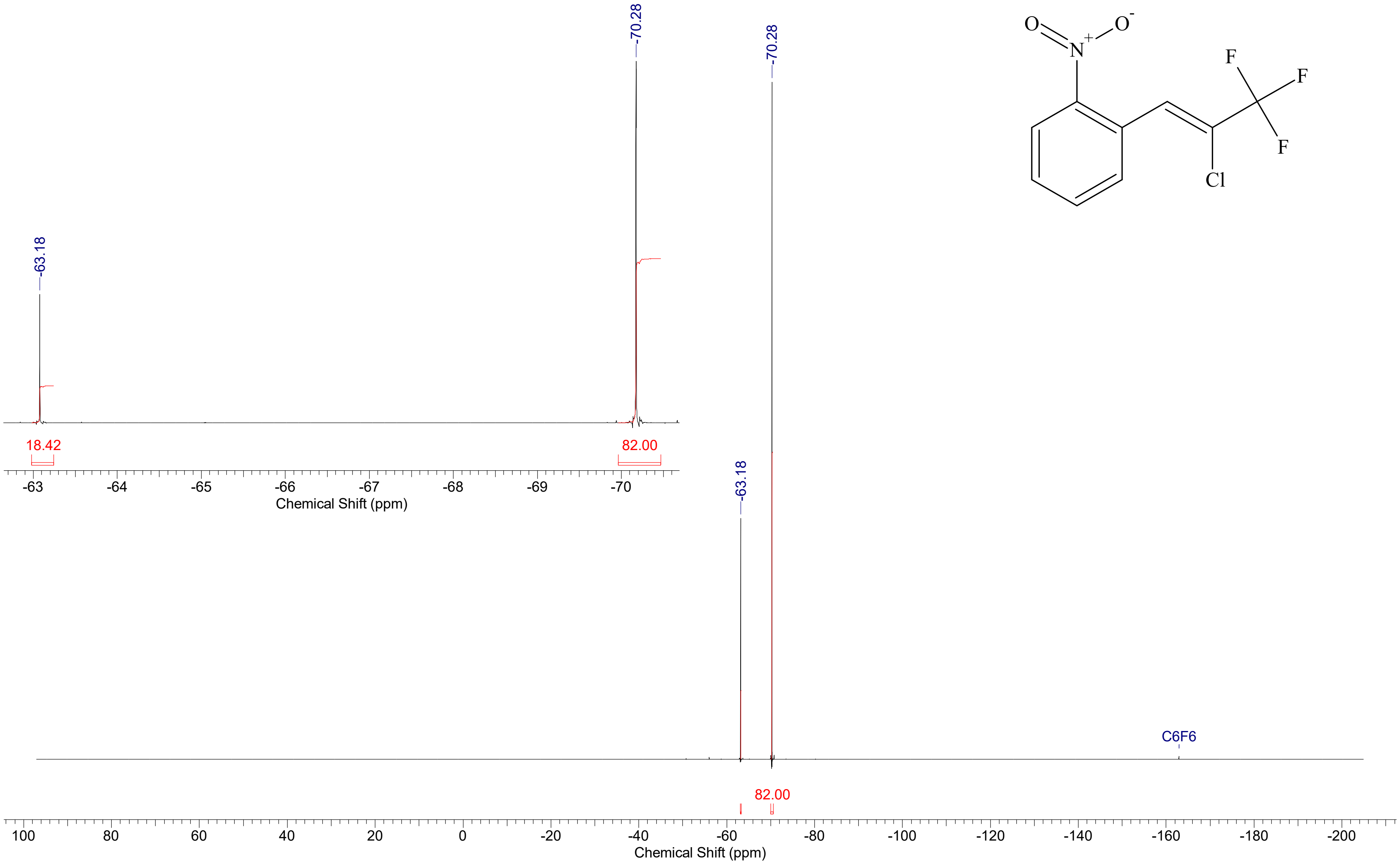

^{19}F NMR spectrum of **2a** (376.5 MHz, $CDCl_3$)

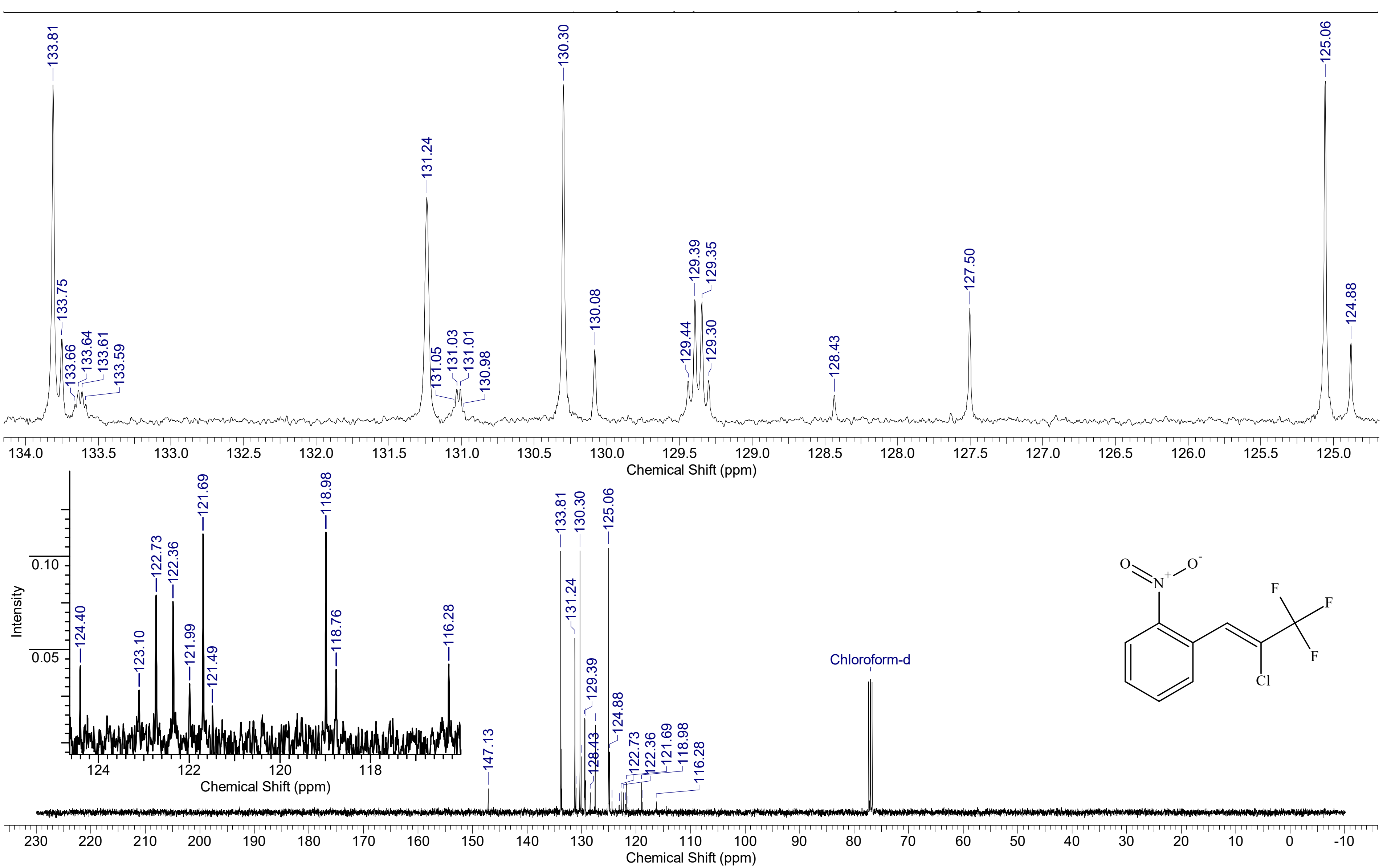

$^{13}C\{^1H\}$ NMR spectrum of **2a** (100.6 MHz, $CDCl_3$)

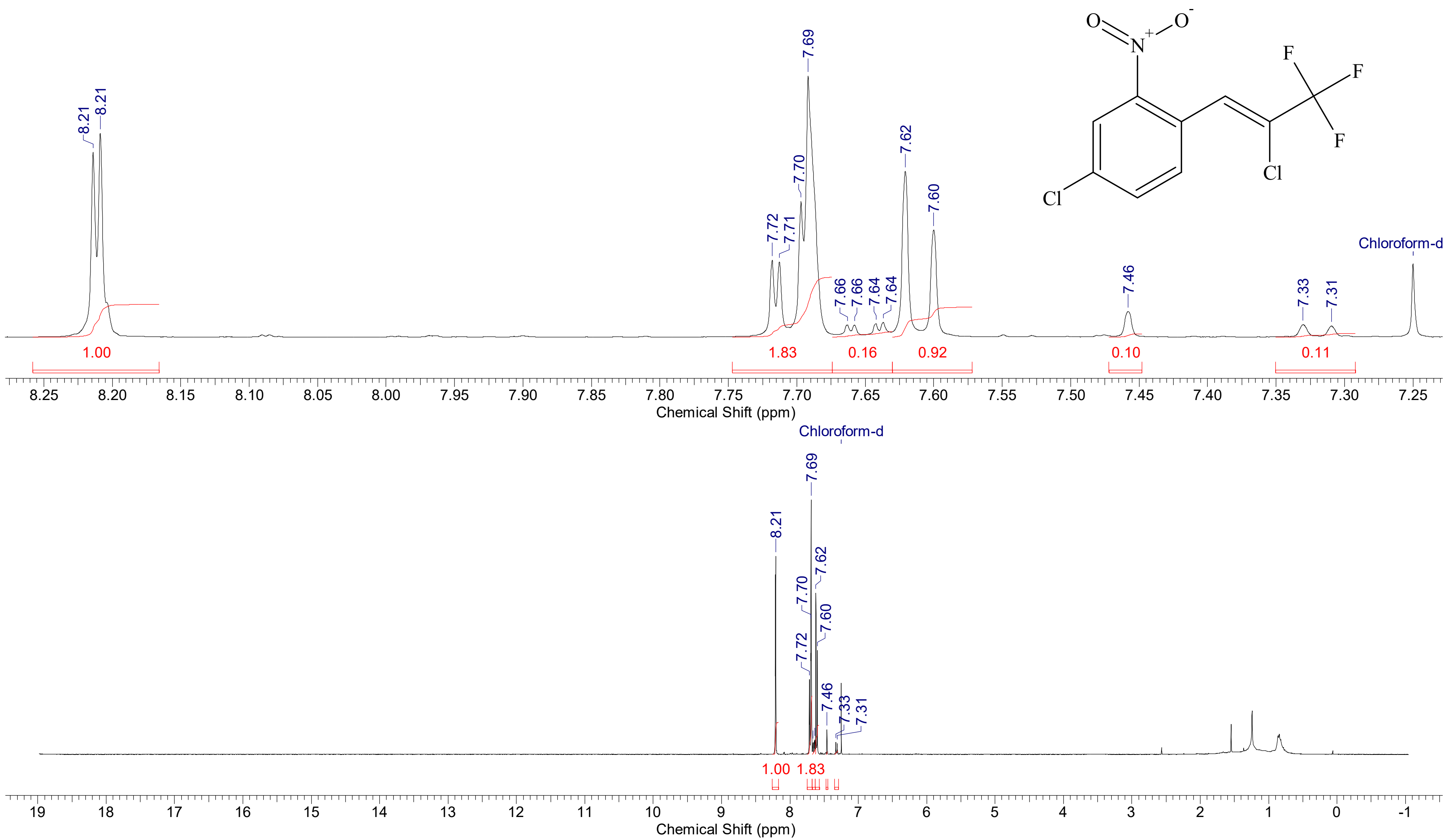

^{1}H NMR spectrum of **2b** (400.1 MHz, $CDCl_3$)

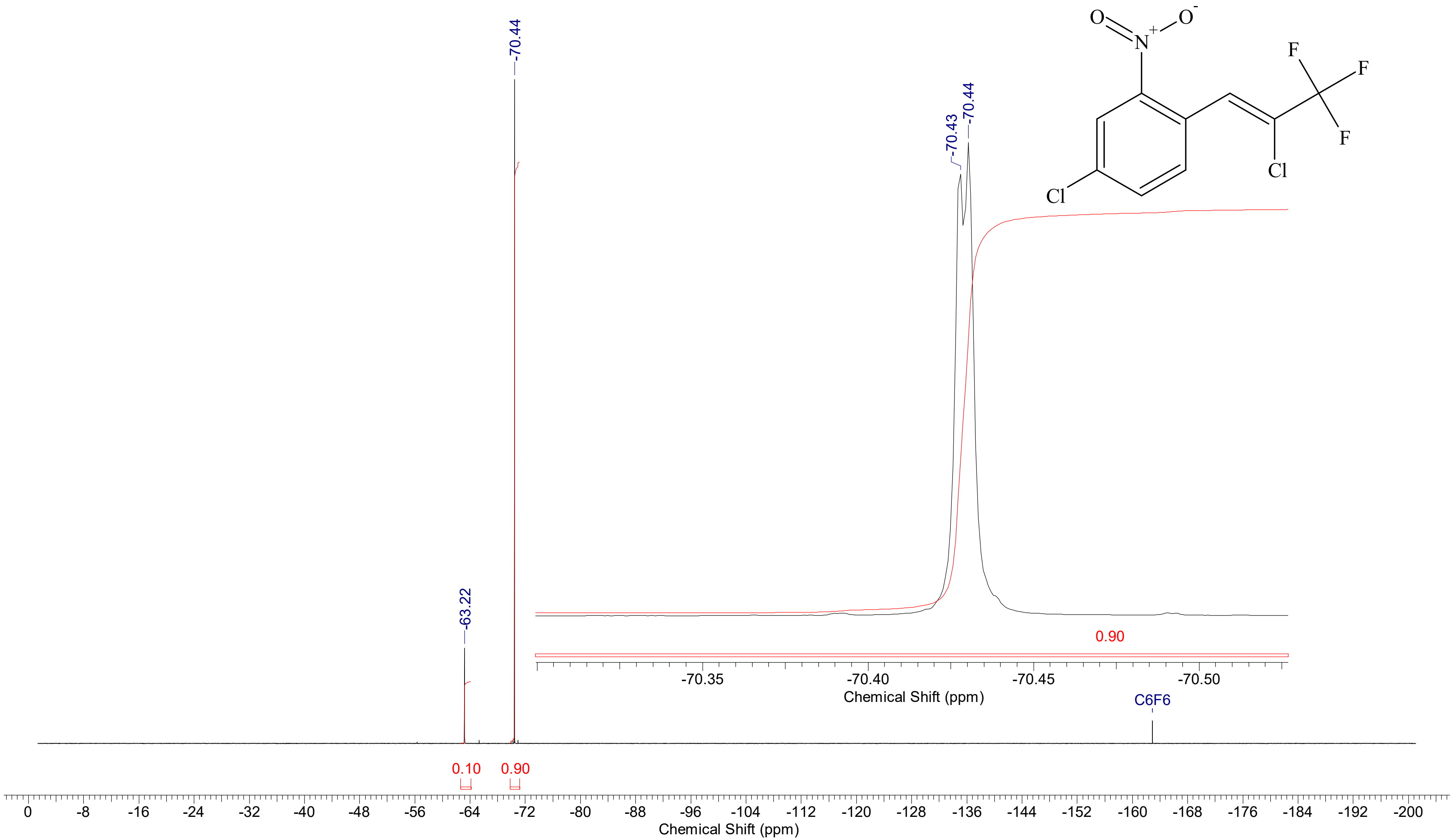

^{19}F NMR spectrum of **2b** (376.5 MHz, $CDCl_3$)

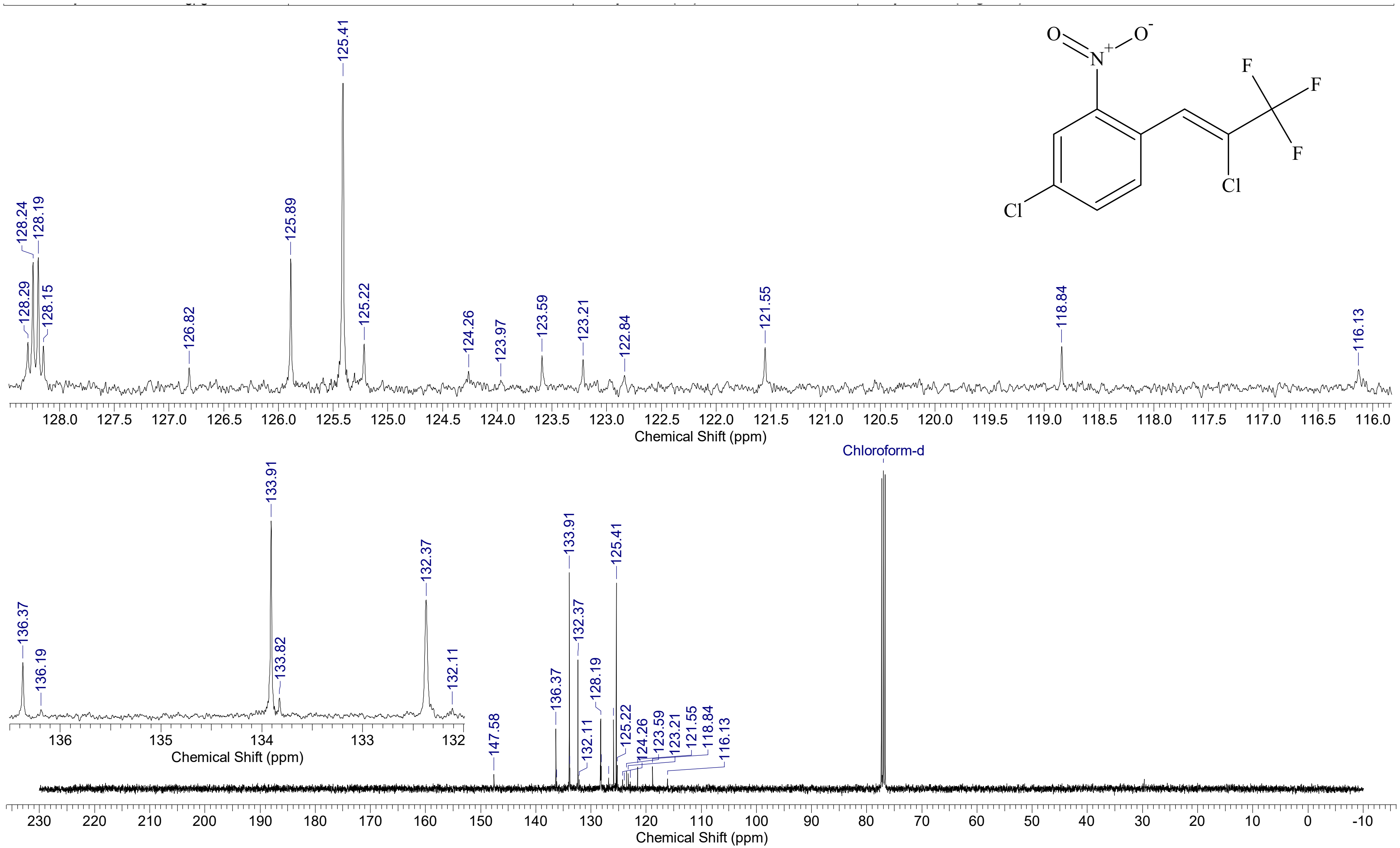

$^{13}C\{^{1}H\}$ NMR spectrum of **2b** (100.6 MHz, $CDCl_3$)

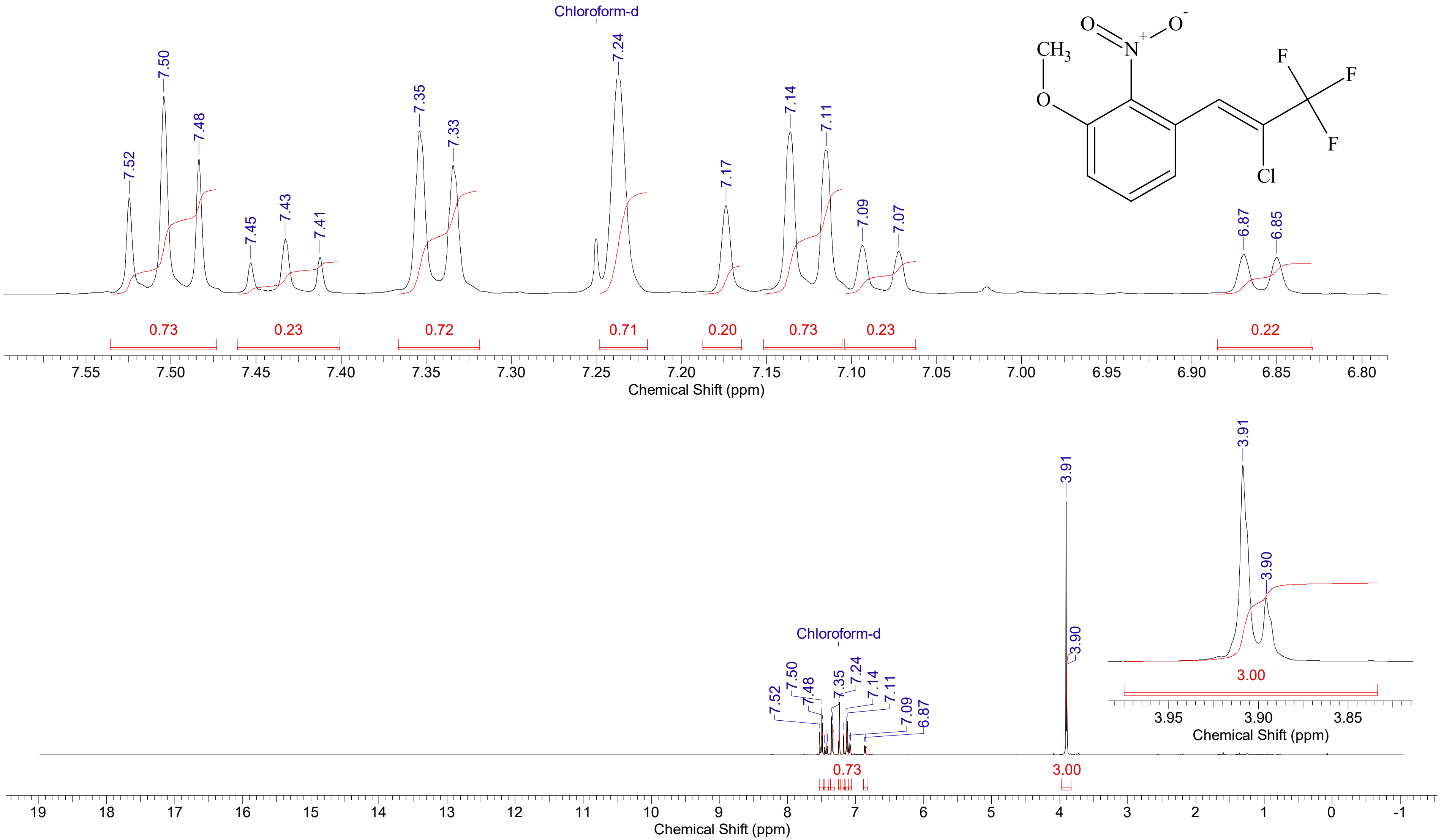

^{1}H NMR spectrum of **2c** (400.1 MHz, $CDCl_3$)

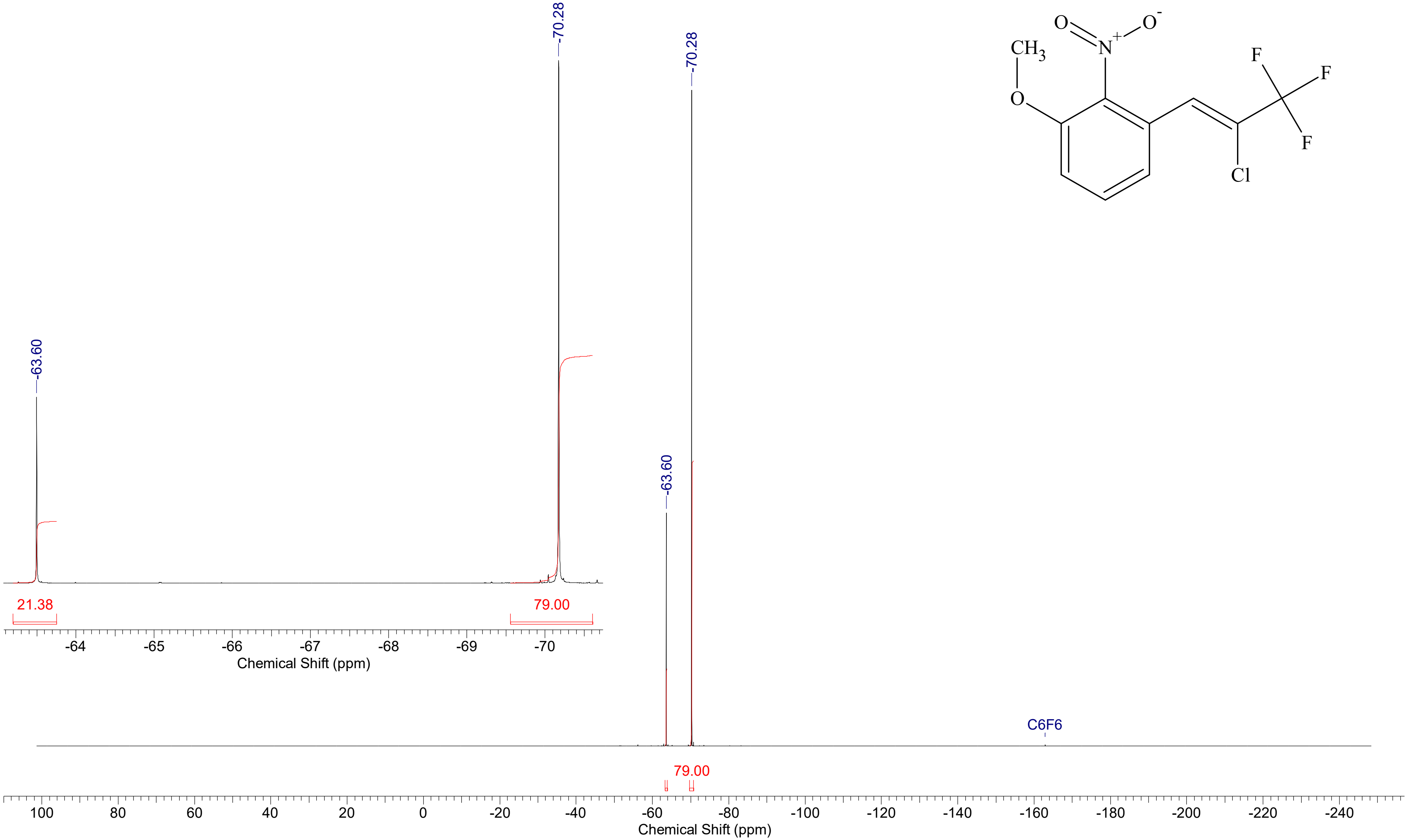

^{19}F NMR spectrum of **2c** (376.5 MHz, $CDCl_3$)

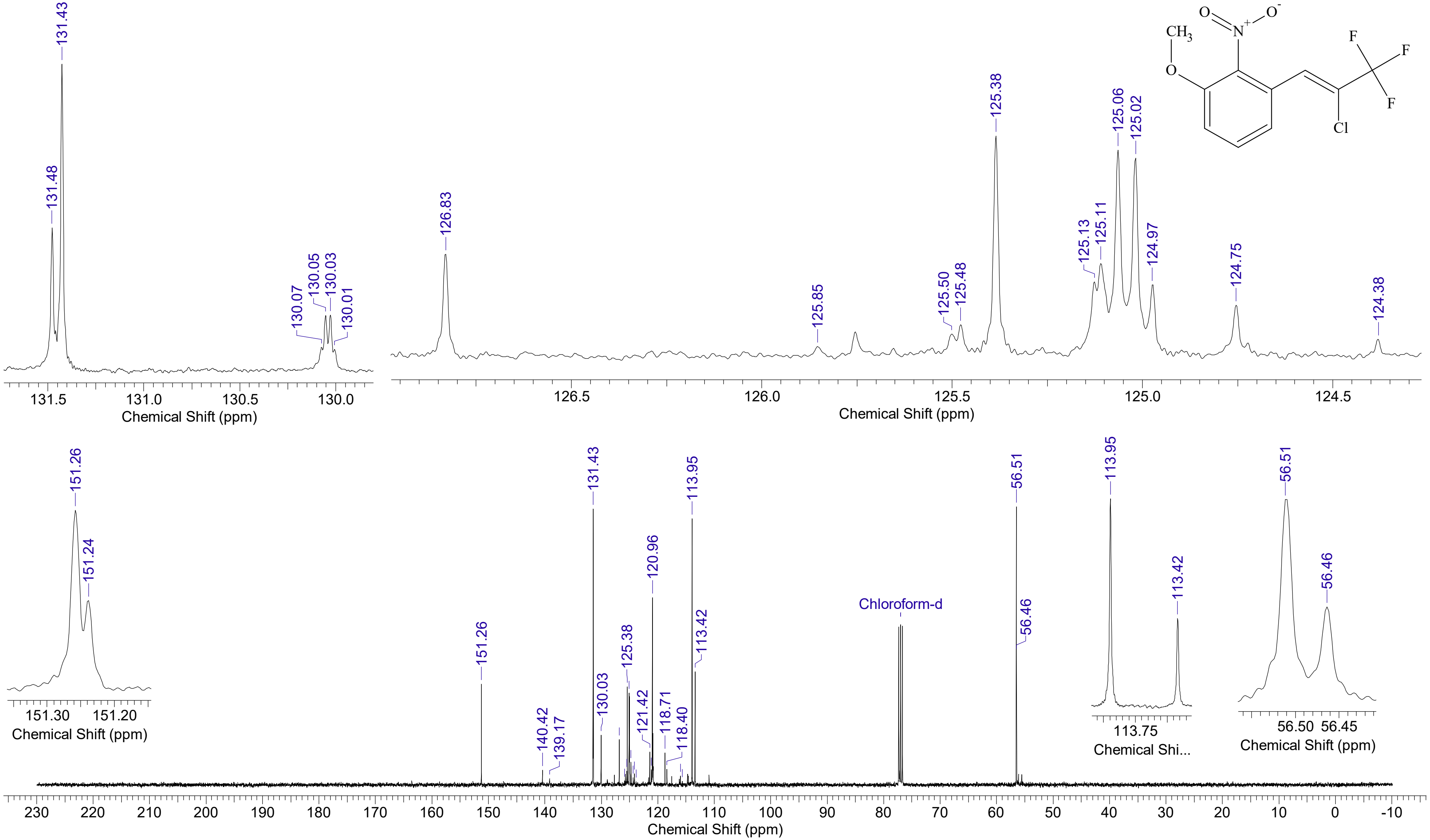

$^{13}C\{^{1}H\}$ NMR spectrum of **2c** (100.6 MHz, $CDCl_3$)

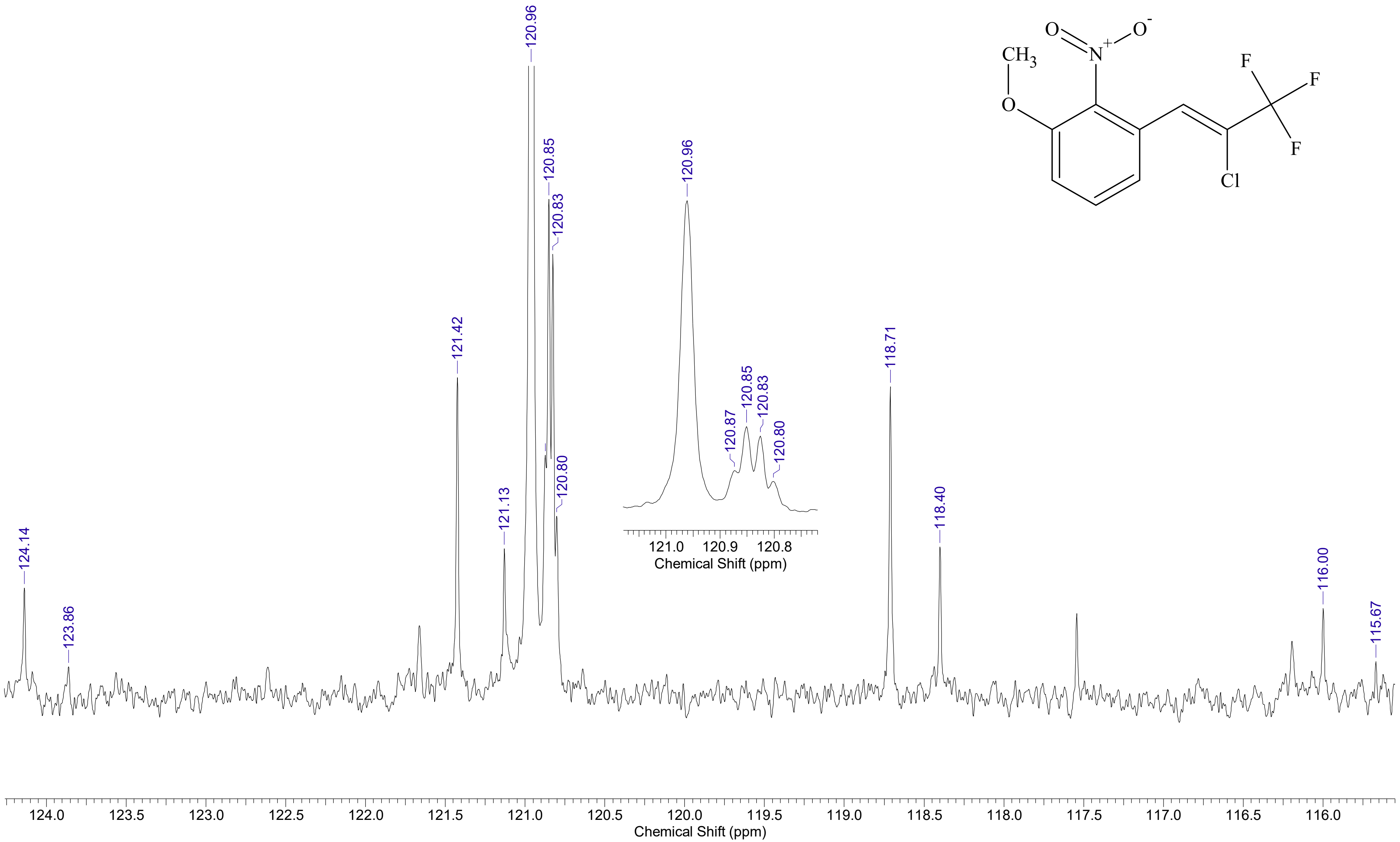

$^{13}C\{^1H\}$ NMR spectrum of **2c** (100.6 MHz, $CDCl_3$)

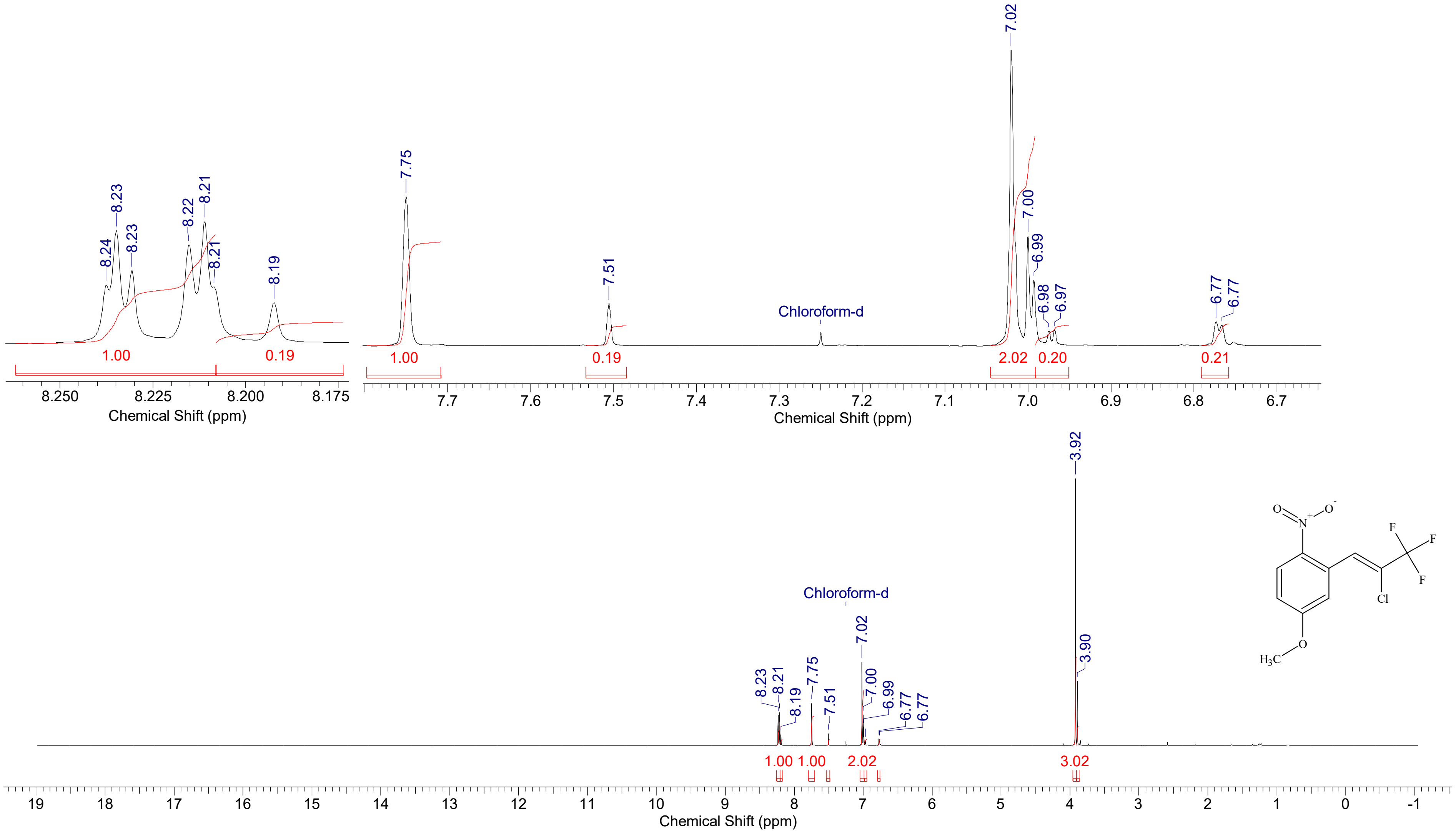

^{1}H NMR spectrum of **2d** (400.1 MHz, $CDCl_3$)

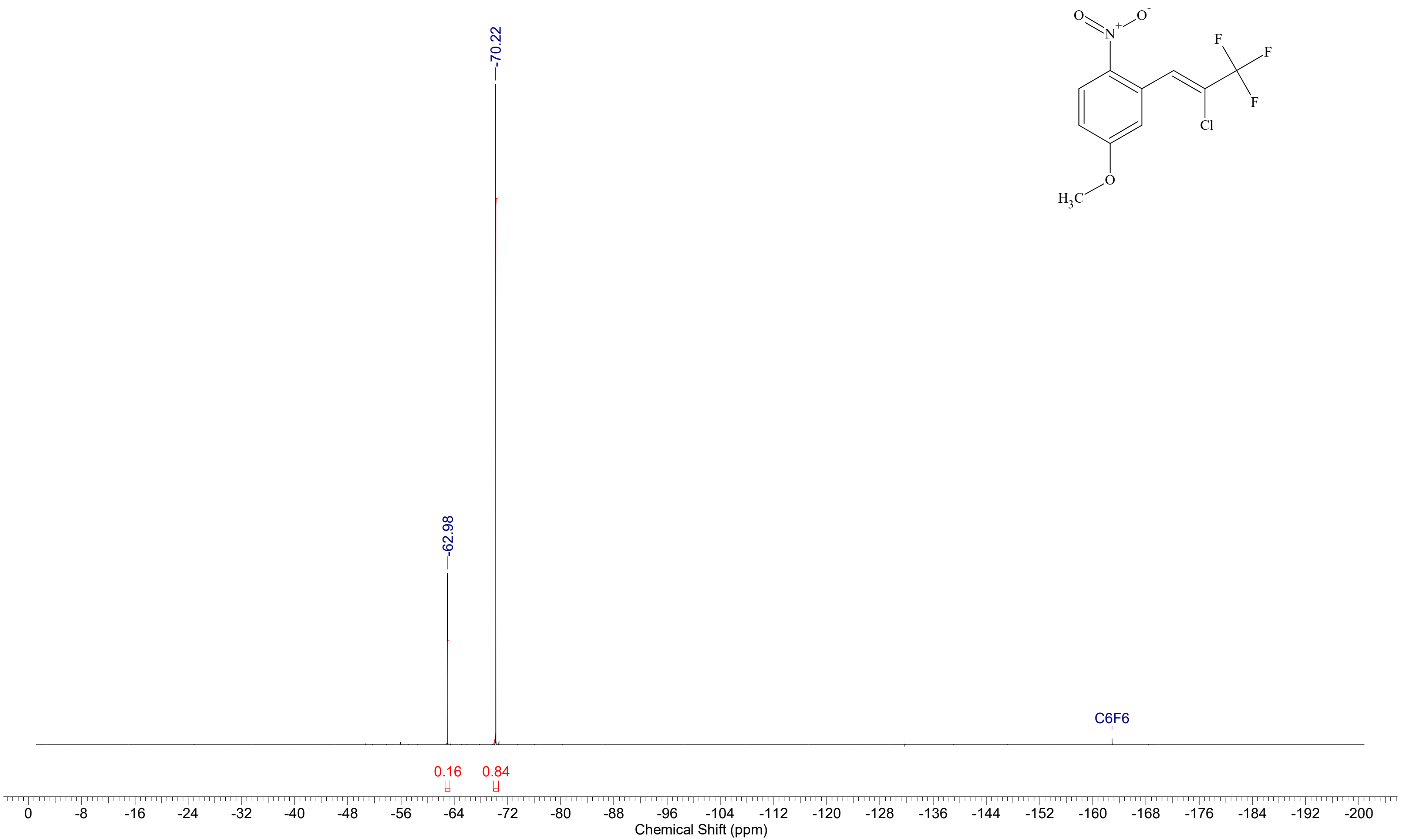

^{19}F NMR spectrum of **2d** (376.5 MHz, $CDCl_3$)

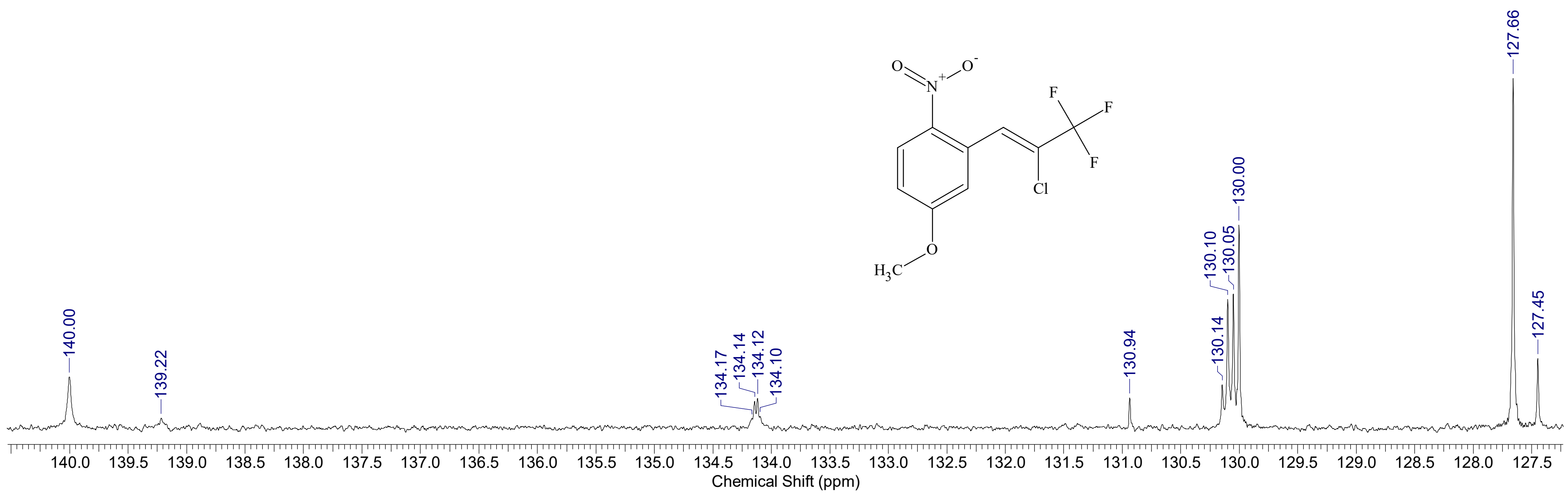

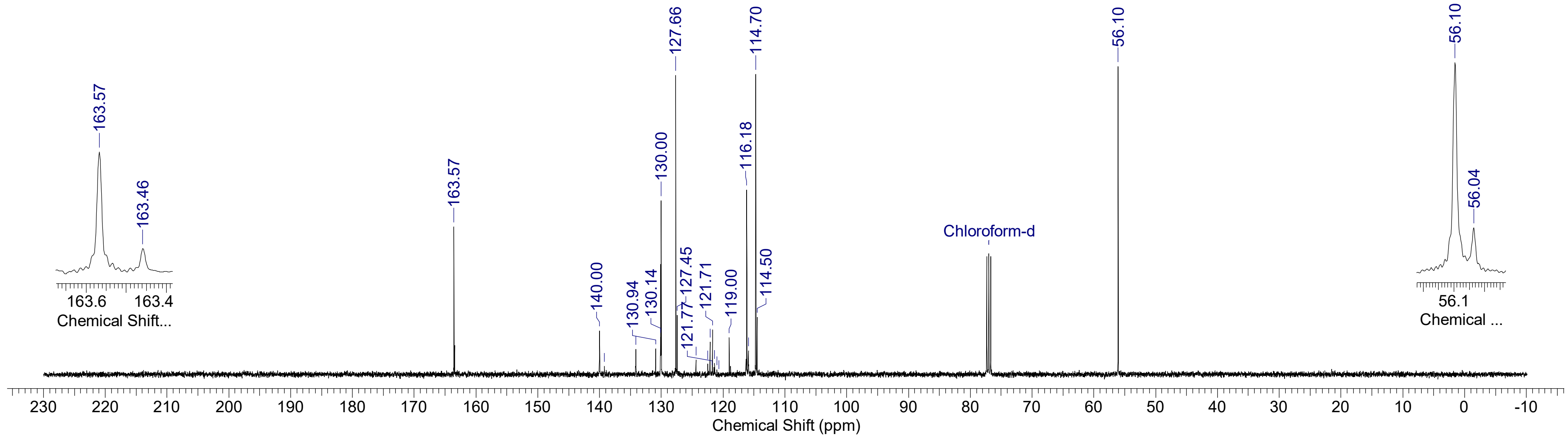

$^{13}C\{^{1}H\}$ NMR spectrum of **2d** (100.6 MHz, $CDCl_3$).

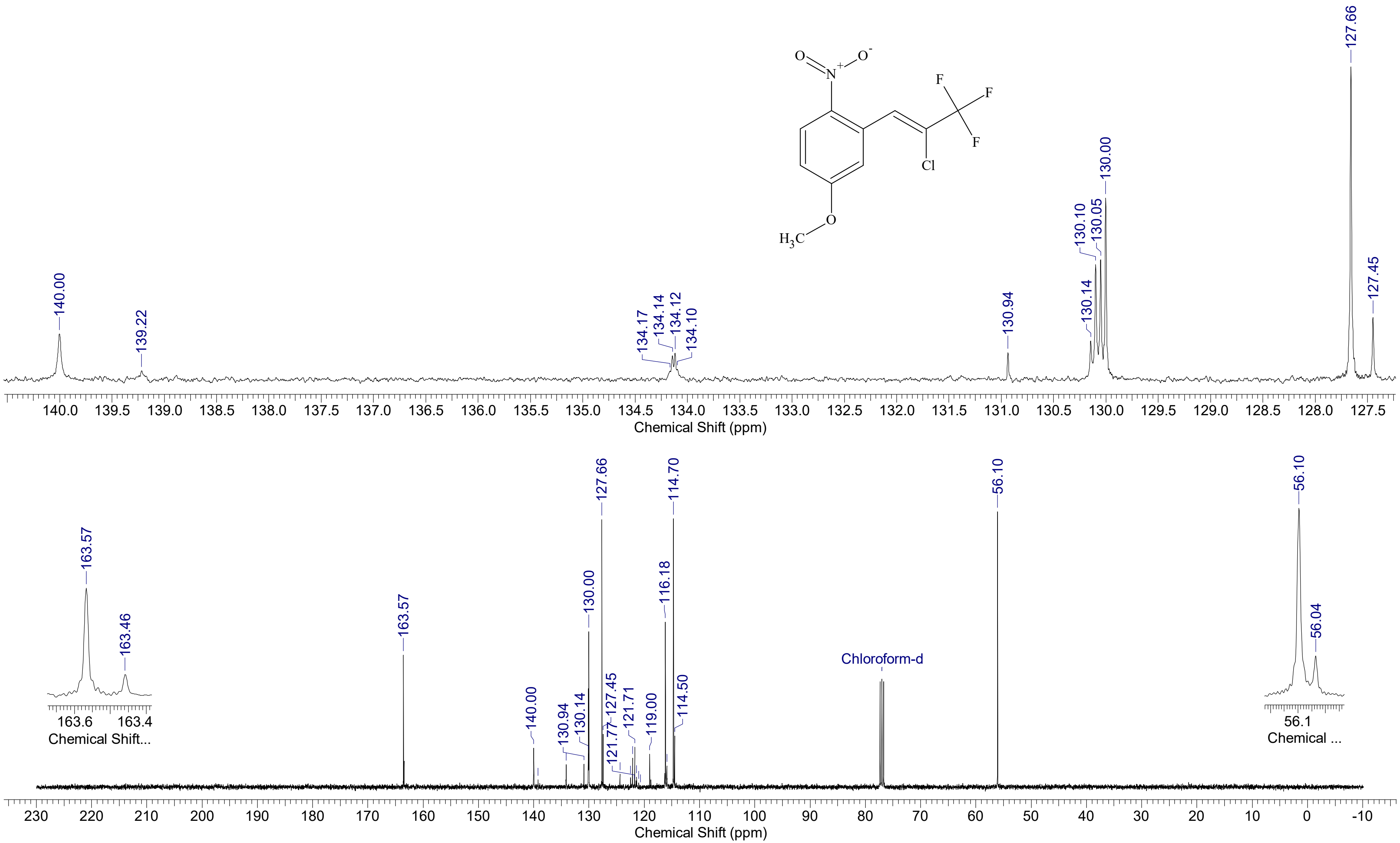

$^{13}C\{^{1}H\}$ NMR spectrum of **2d** (100.6 MHz, $CDCl_3$).

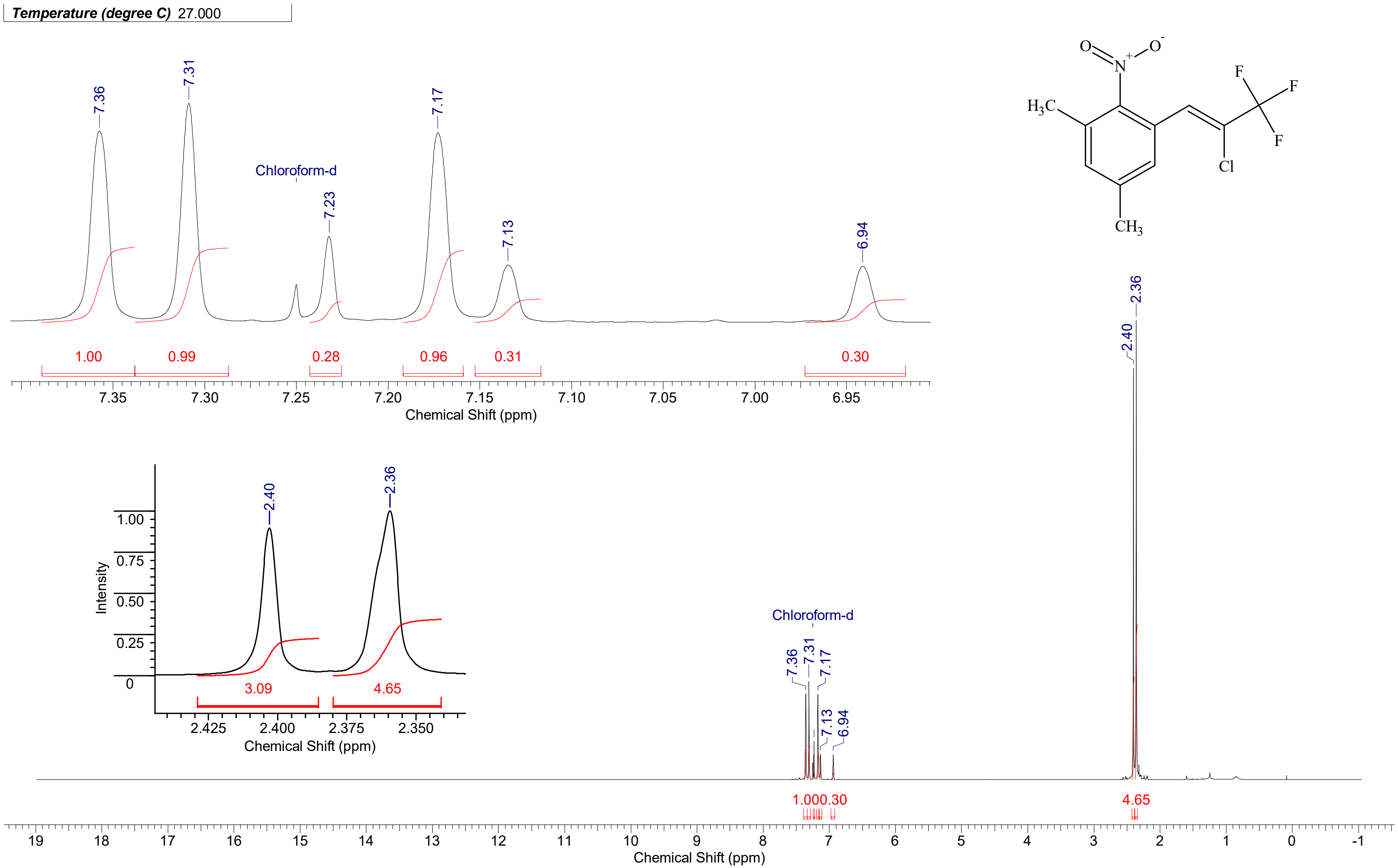

^{1}H NMR spectrum of **2e** (400.1 MHz, $CDCl_3$)

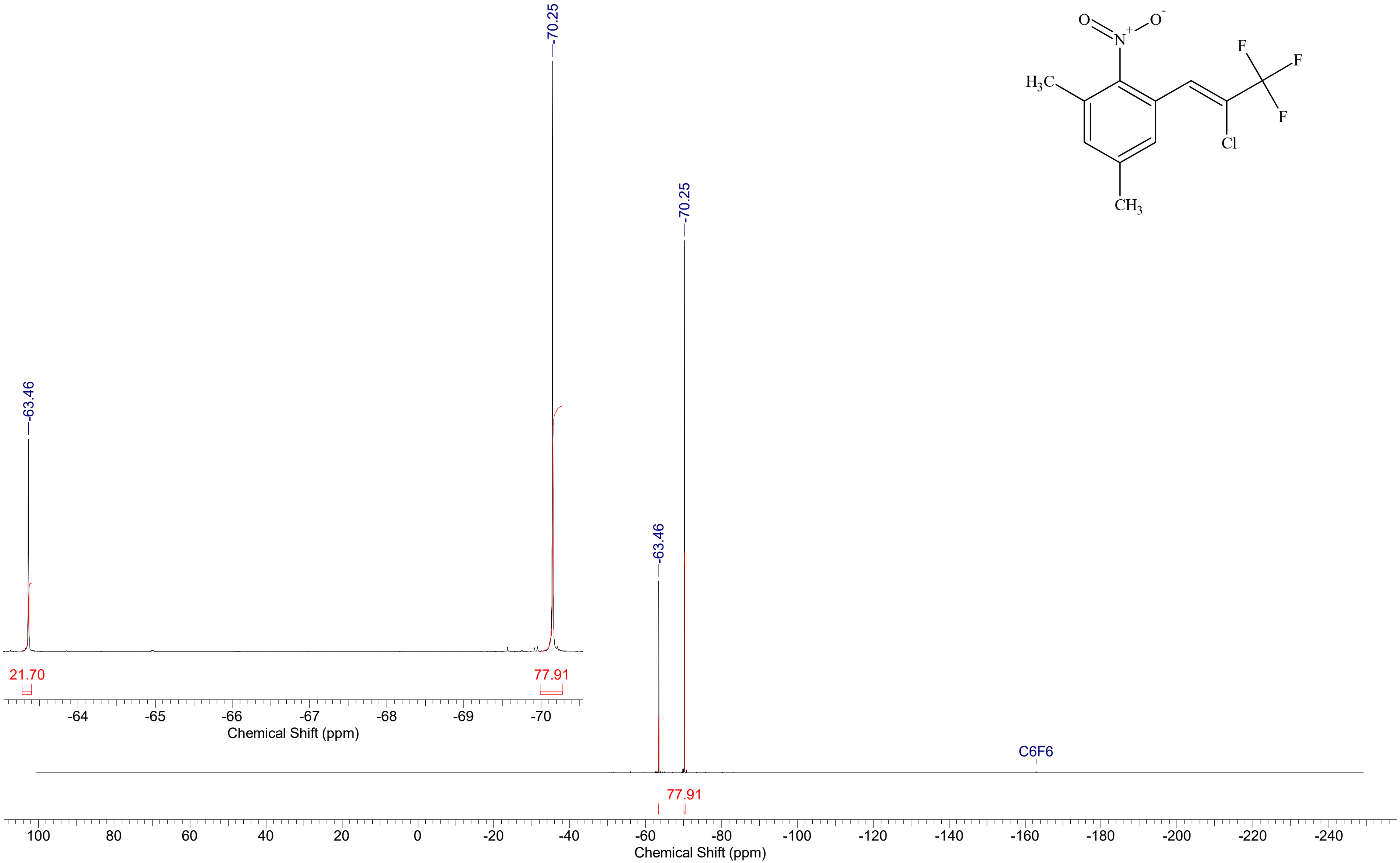

^{19}F NMR spectrum of **2e** (376.5 MHz, $CDCl_3$)

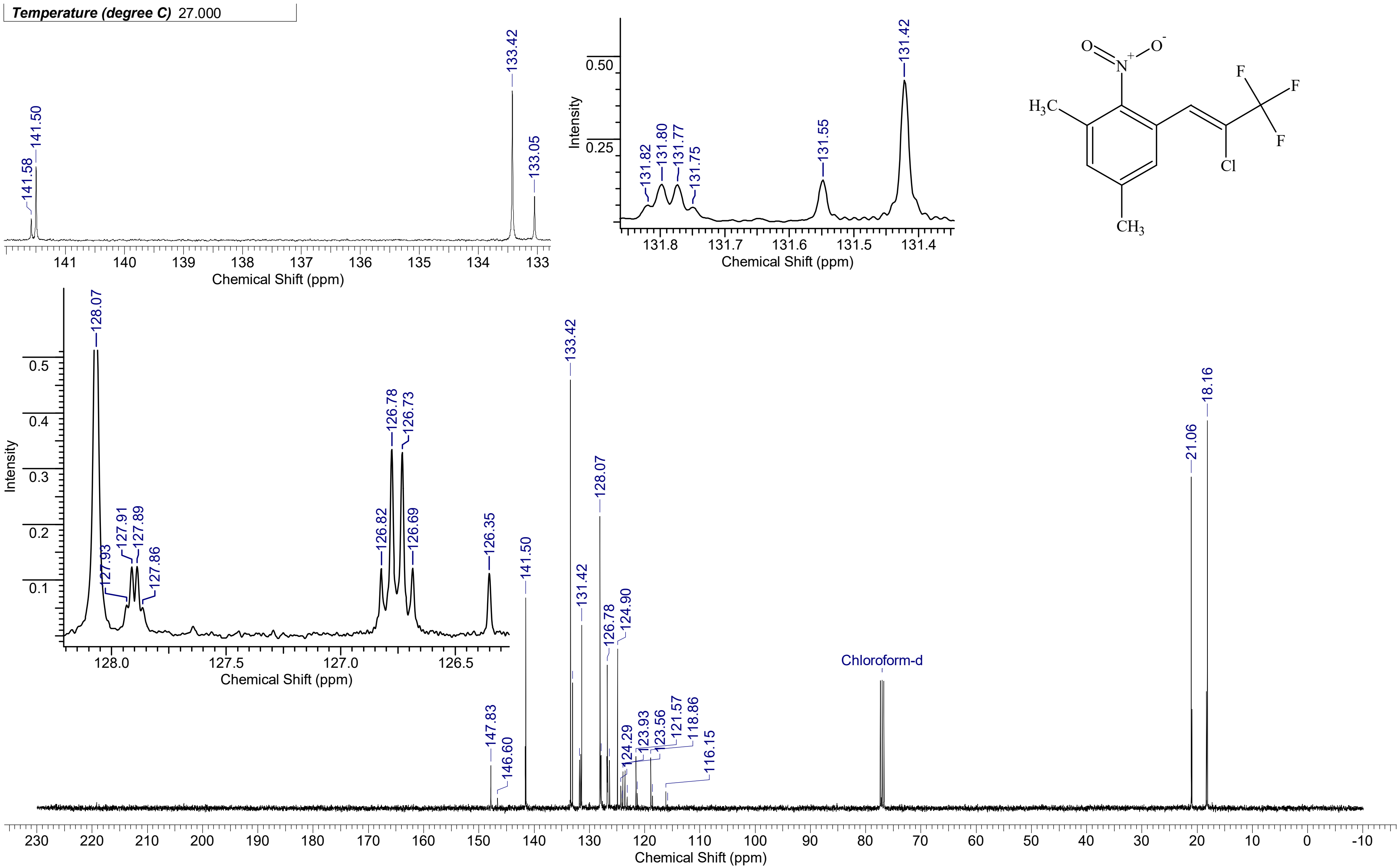

$^{13}C\{^{1}H\}$ NMR spectrum of **2e** (100.6 MHz, $CDCl_3$)

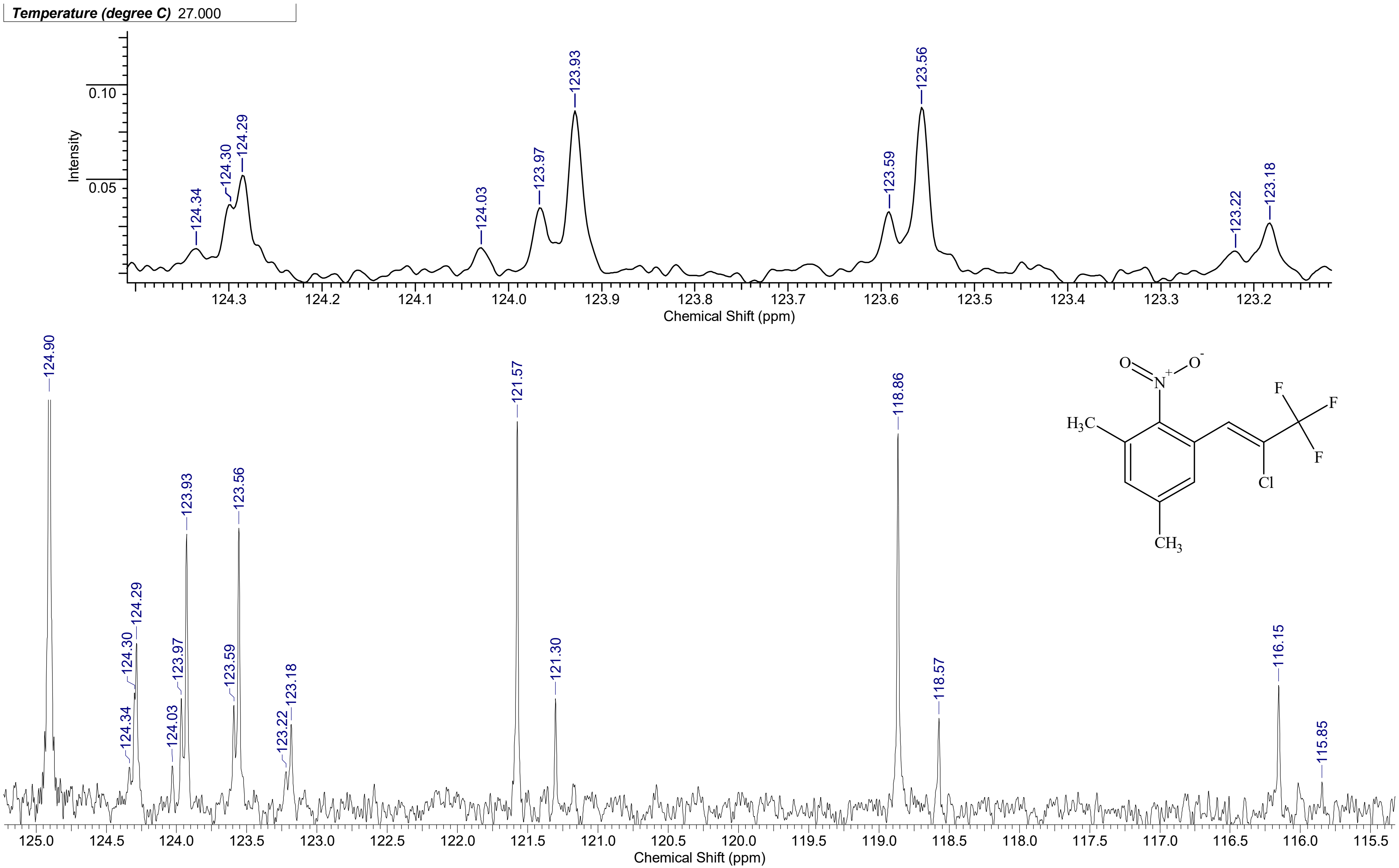

$^{13}C\{^{1}H\}$ NMR spectrum of **2e** (100.6 MHz, $CDCl_3$)

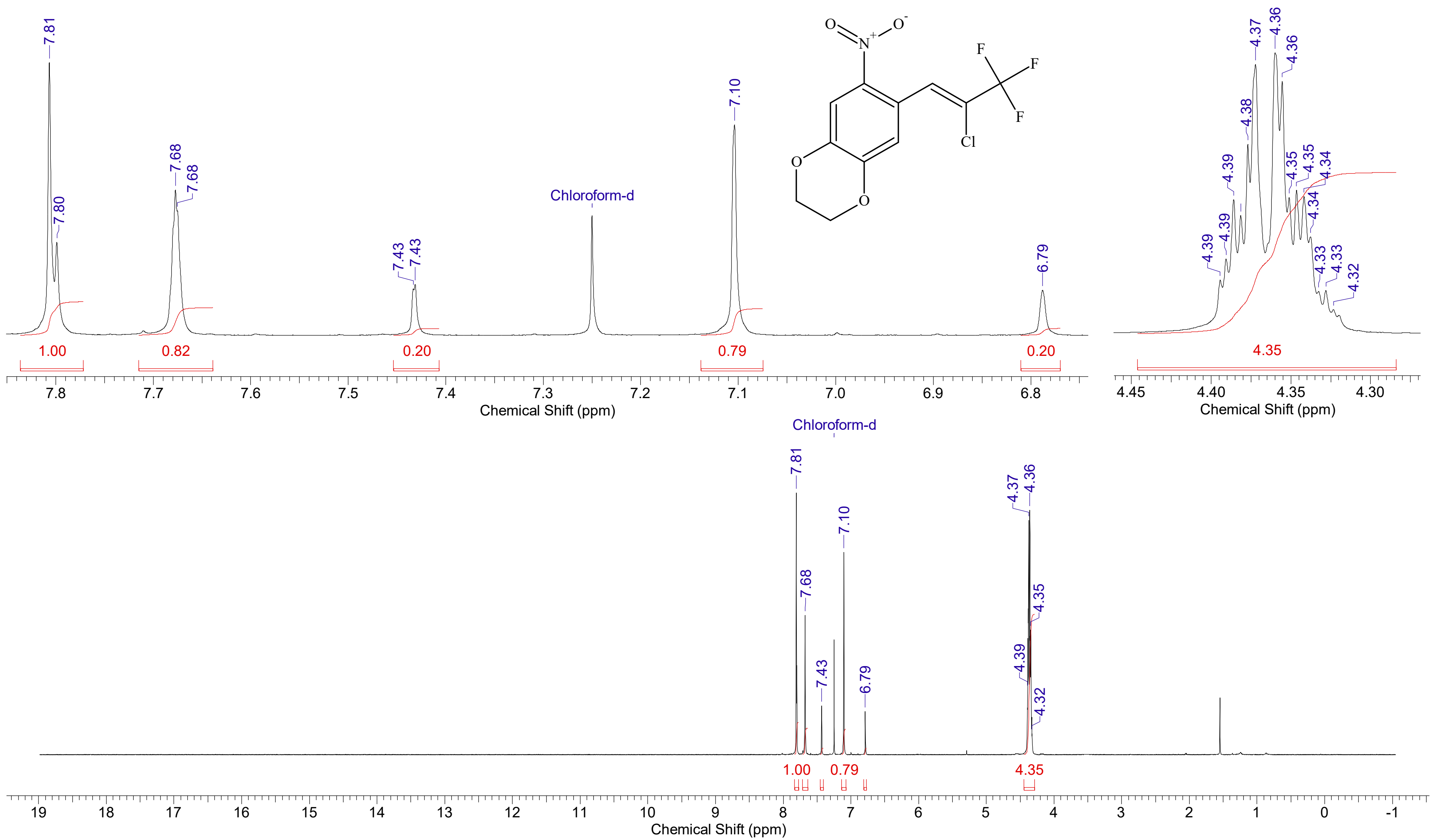

^{1}H NMR spectrum of **2f** (400.1 MHz, $CDCl_3$)

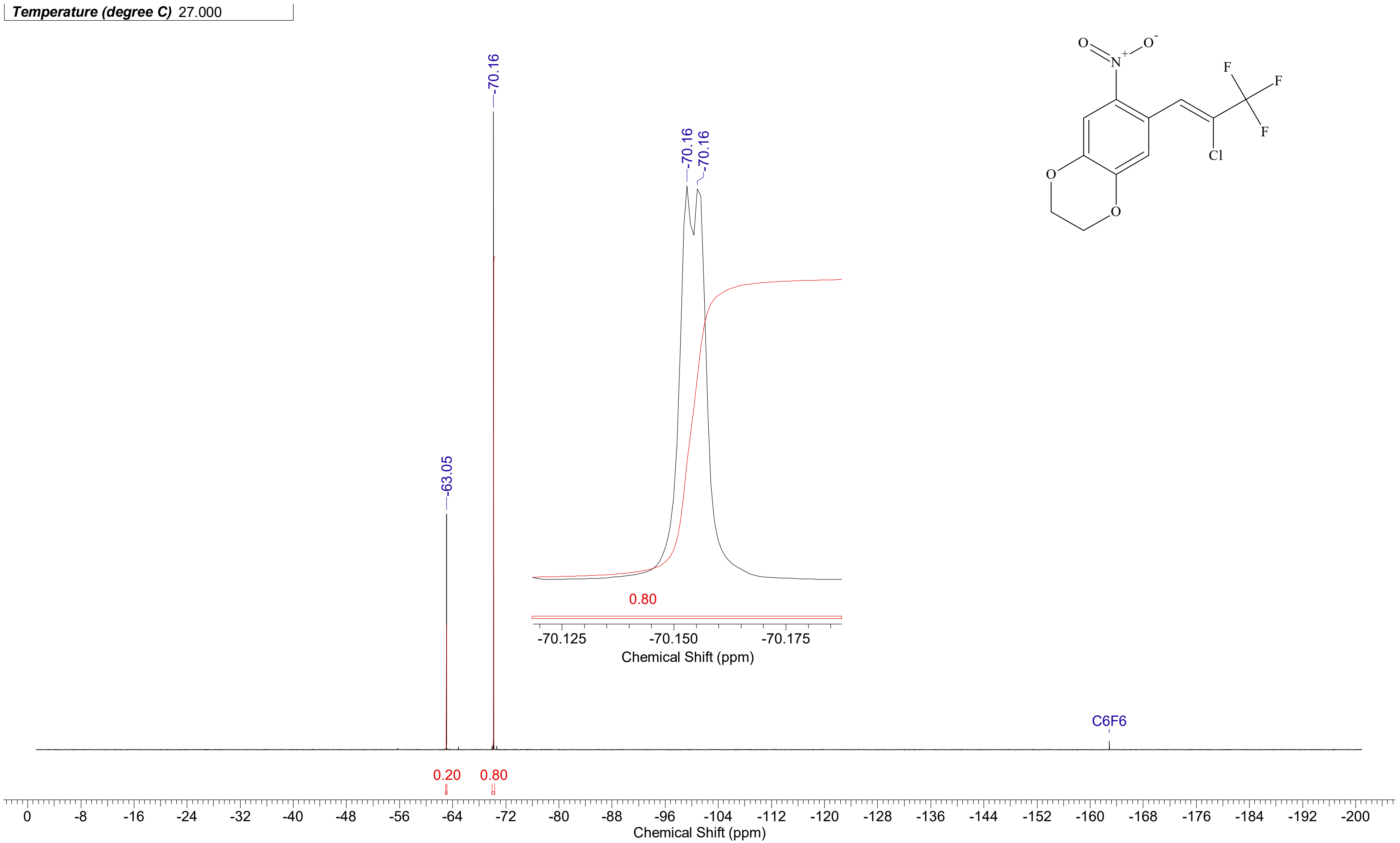

^{19}F NMR spectrum of **2f** (376.5 MHz, $CDCl_3$)

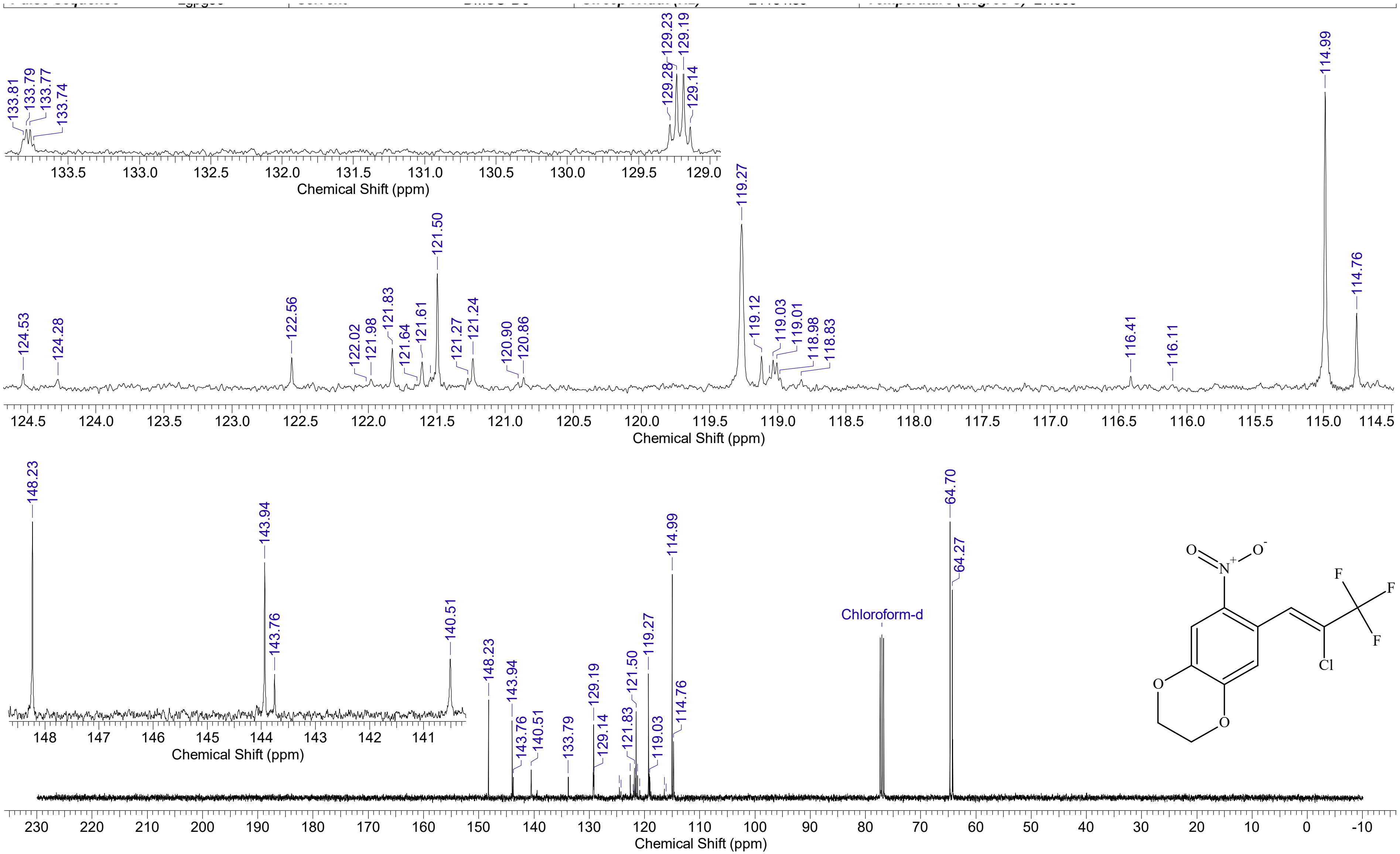

$^{13}C\{^1H\}$ NMR spectrum of **2f** (100.6 MHz, $CDCl_3$)

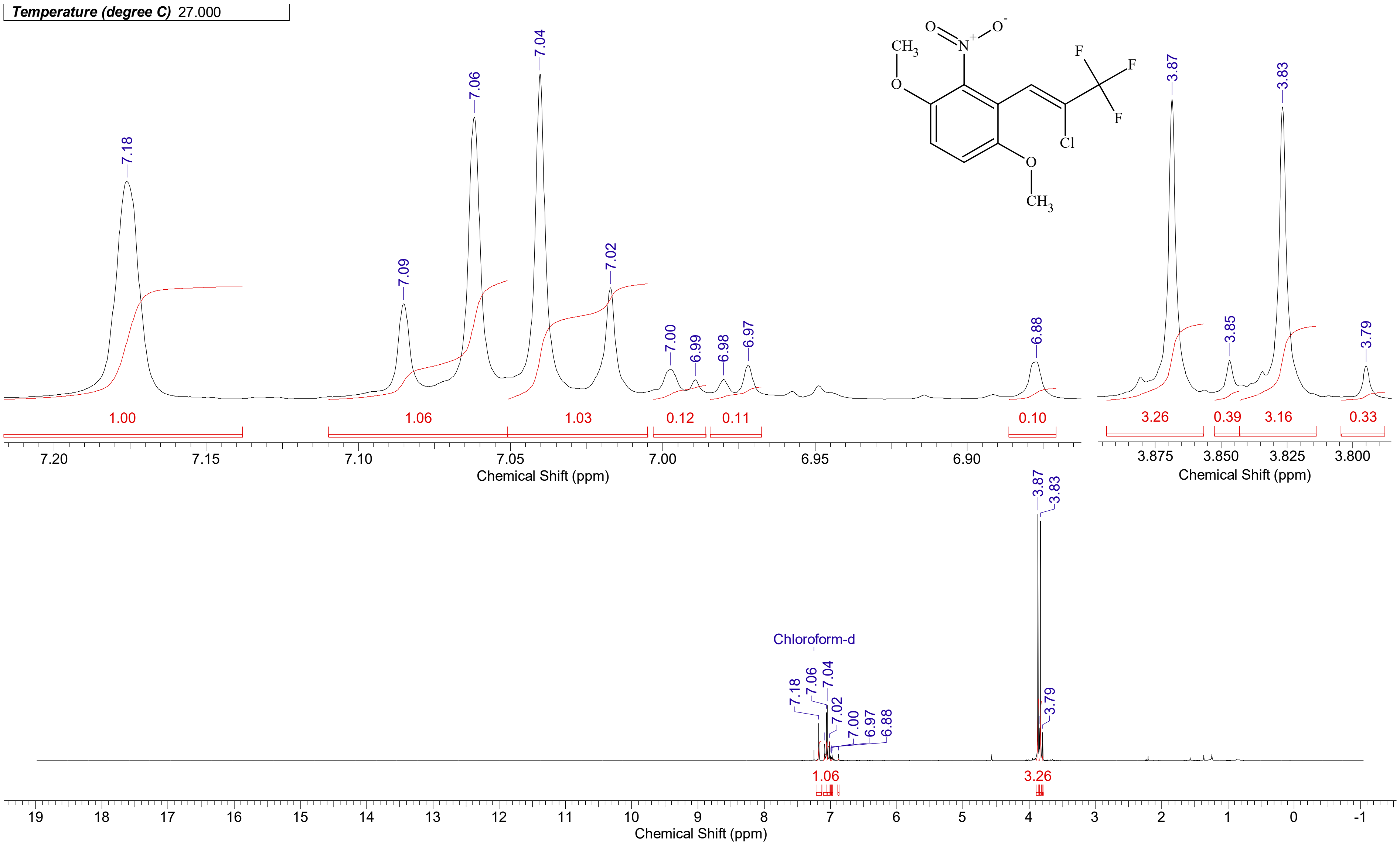

^{1}H NMR spectrum of **2g** (400.1 MHz, $CDCl_3$)

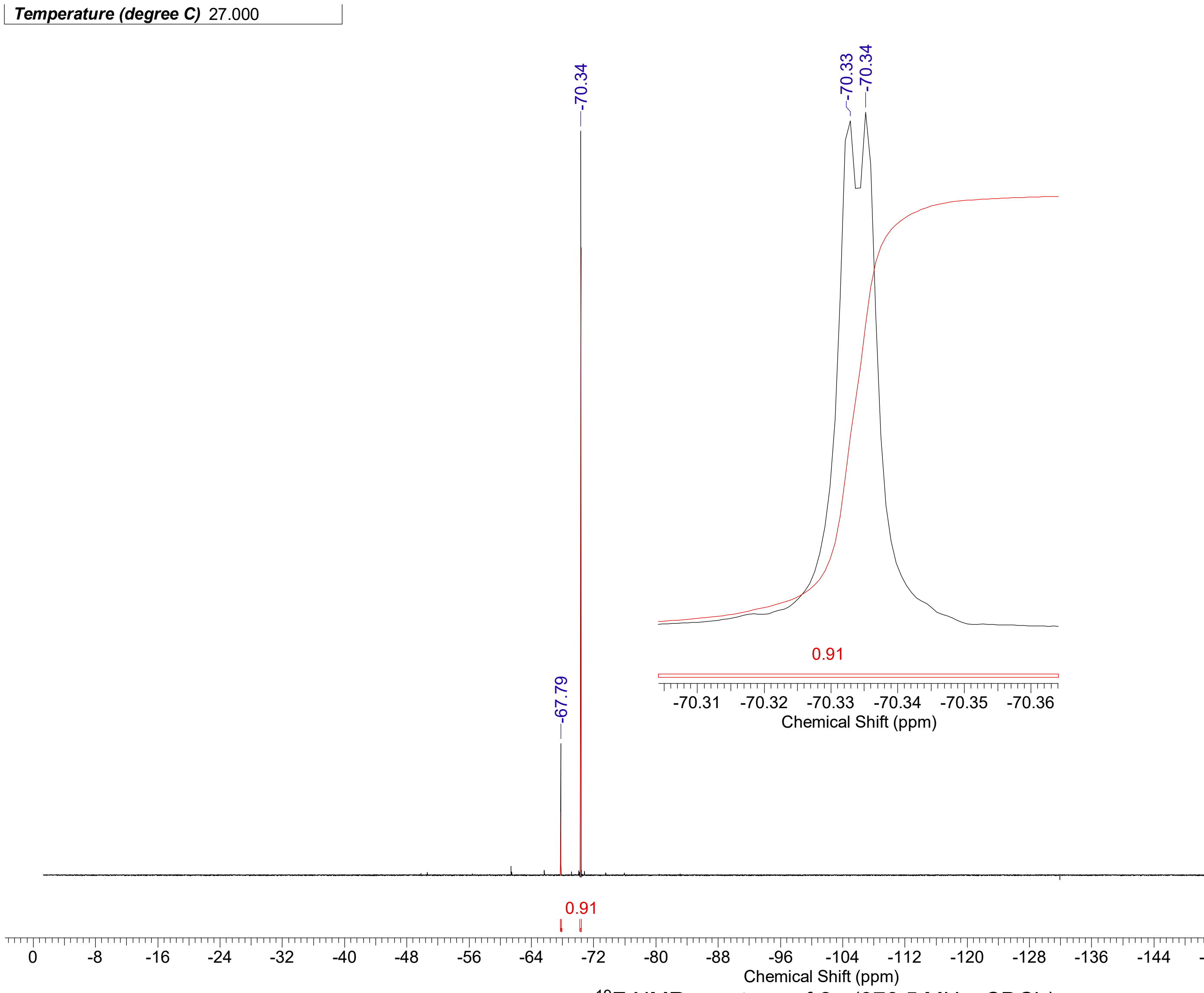

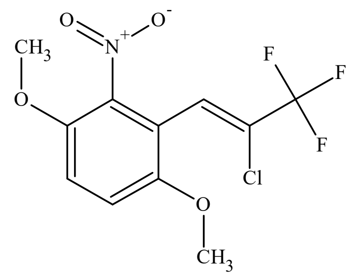

^{19}F NMR spectrum of **2g** (376.5 MHz, $CDCl_3$)

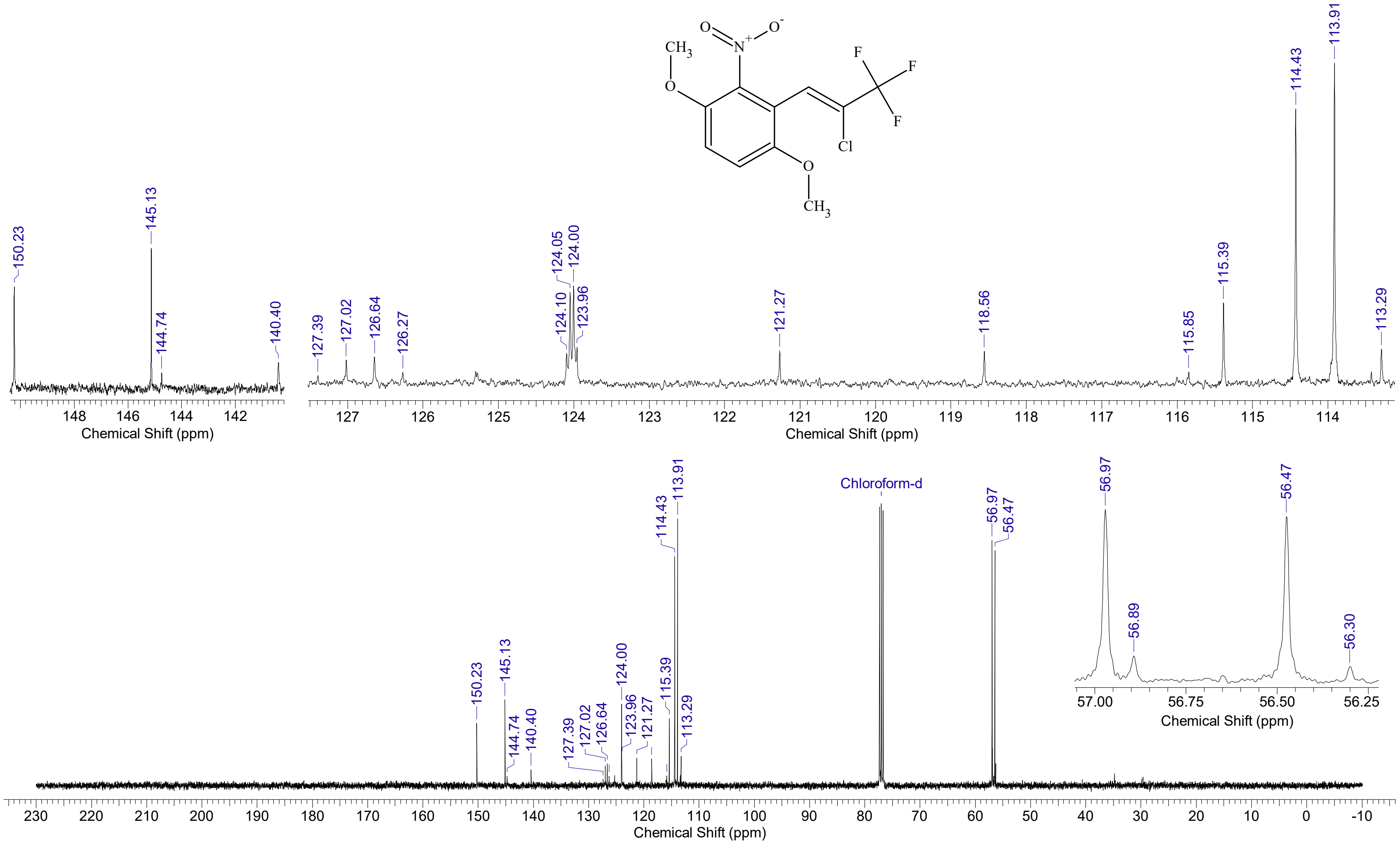

$^{13}C\{^1H\}$ NMR spectrum of **2g** (100.6 MHz, $CDCl_3$)

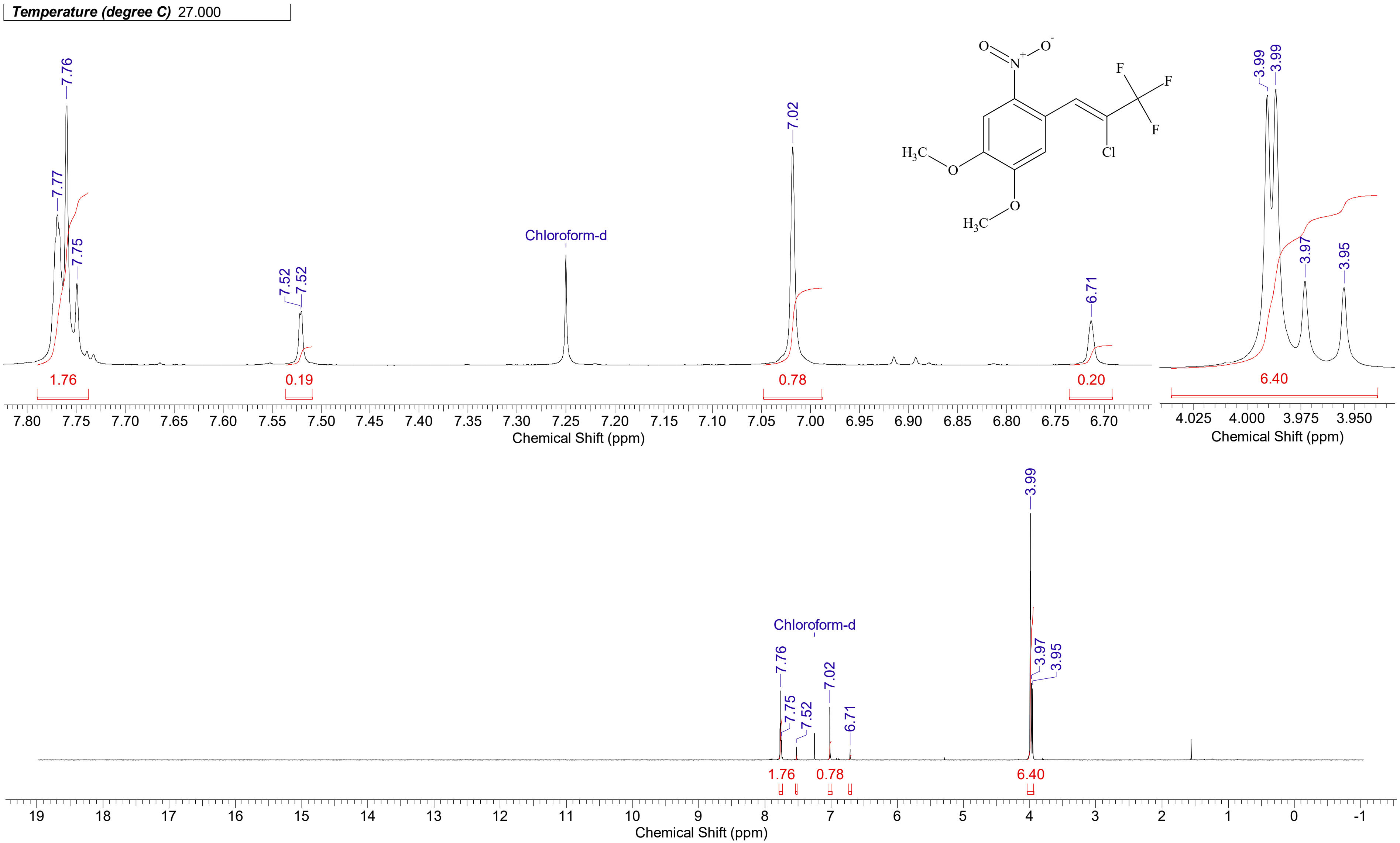

^{1}H NMR spectrum of **2h** (400.1 MHz, $CDCl_3$)

[illegible]

Temperature (degree C)	27.000

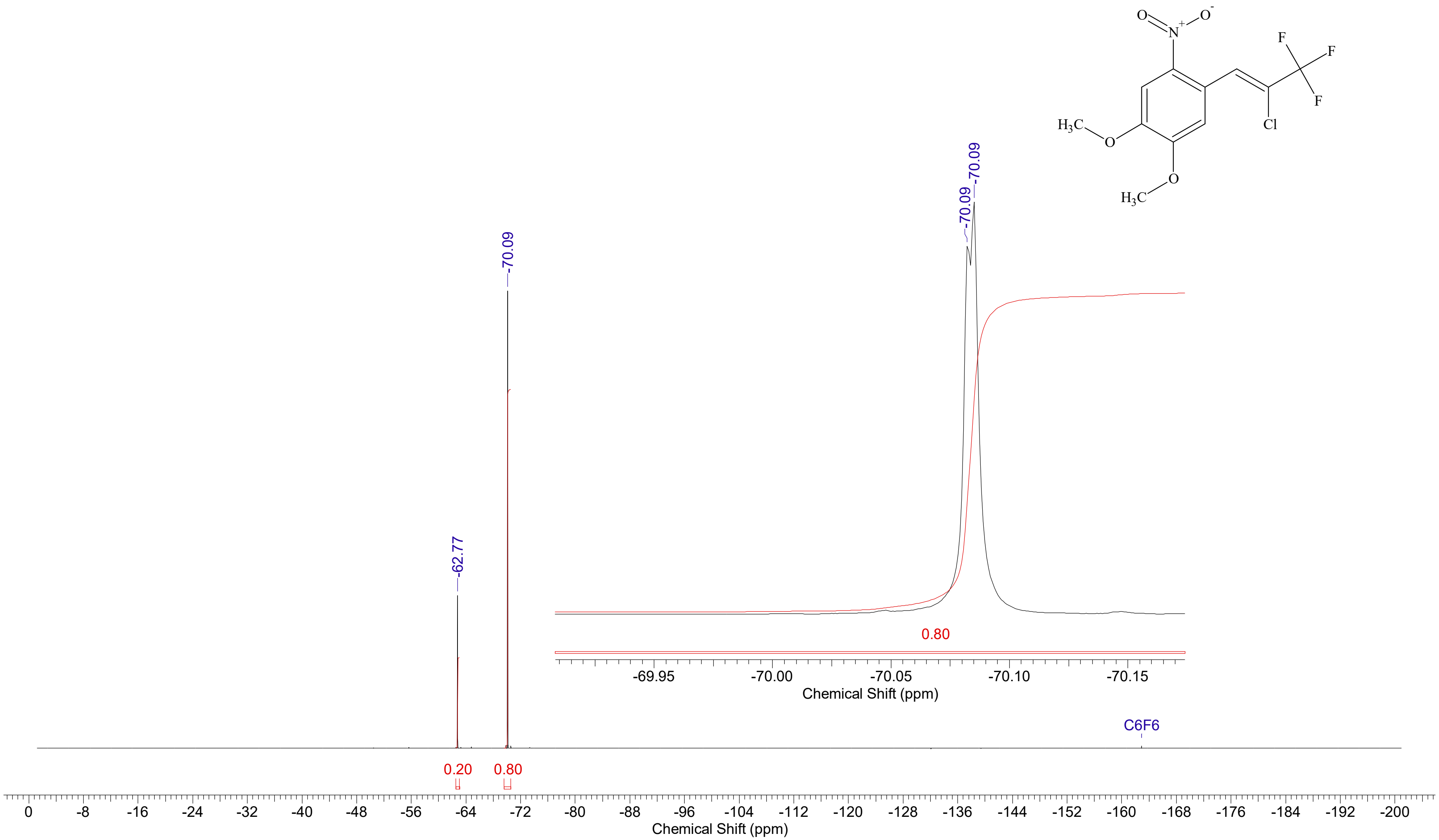

^{19}F NMR spectrum of **2h** (376.5 MHz, $CDCl_3$)

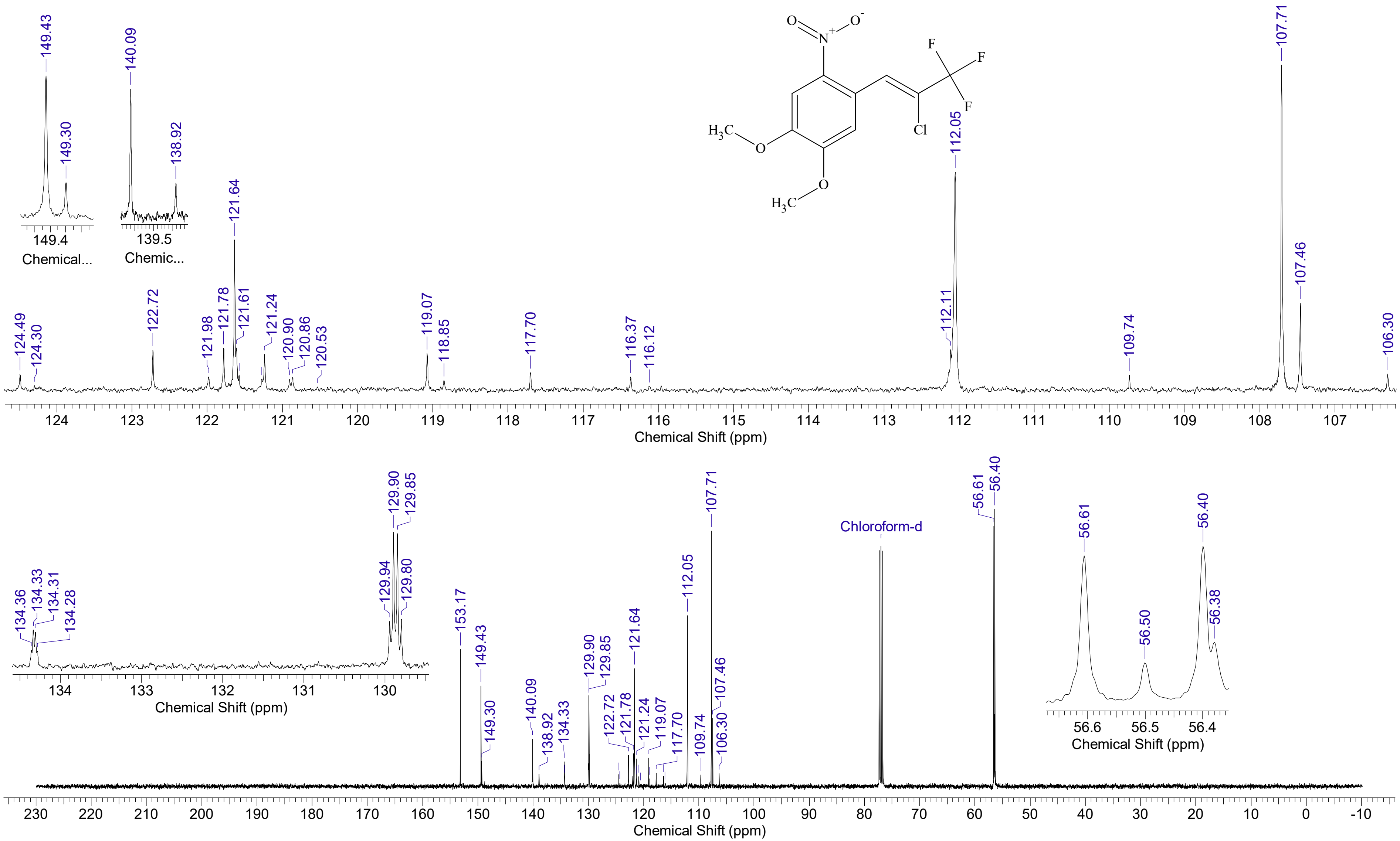

$^{13}C\{^{1}H\}$ NMR spectrum of **2h** (100.6 MHz, $CDCl_3$)

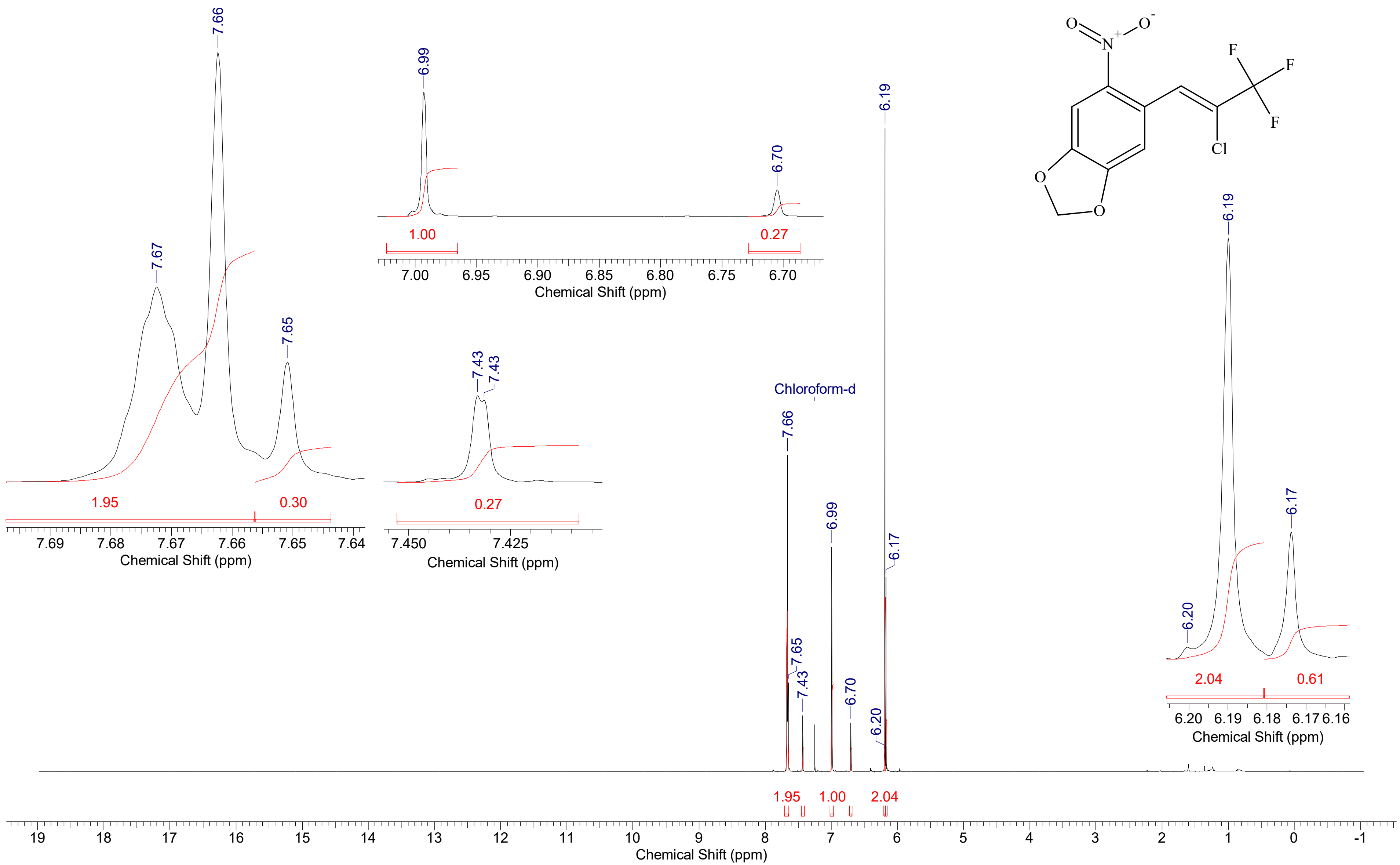

^{1}H NMR spectrum of **2i** (400.1 MHz, $CDCl_3$)

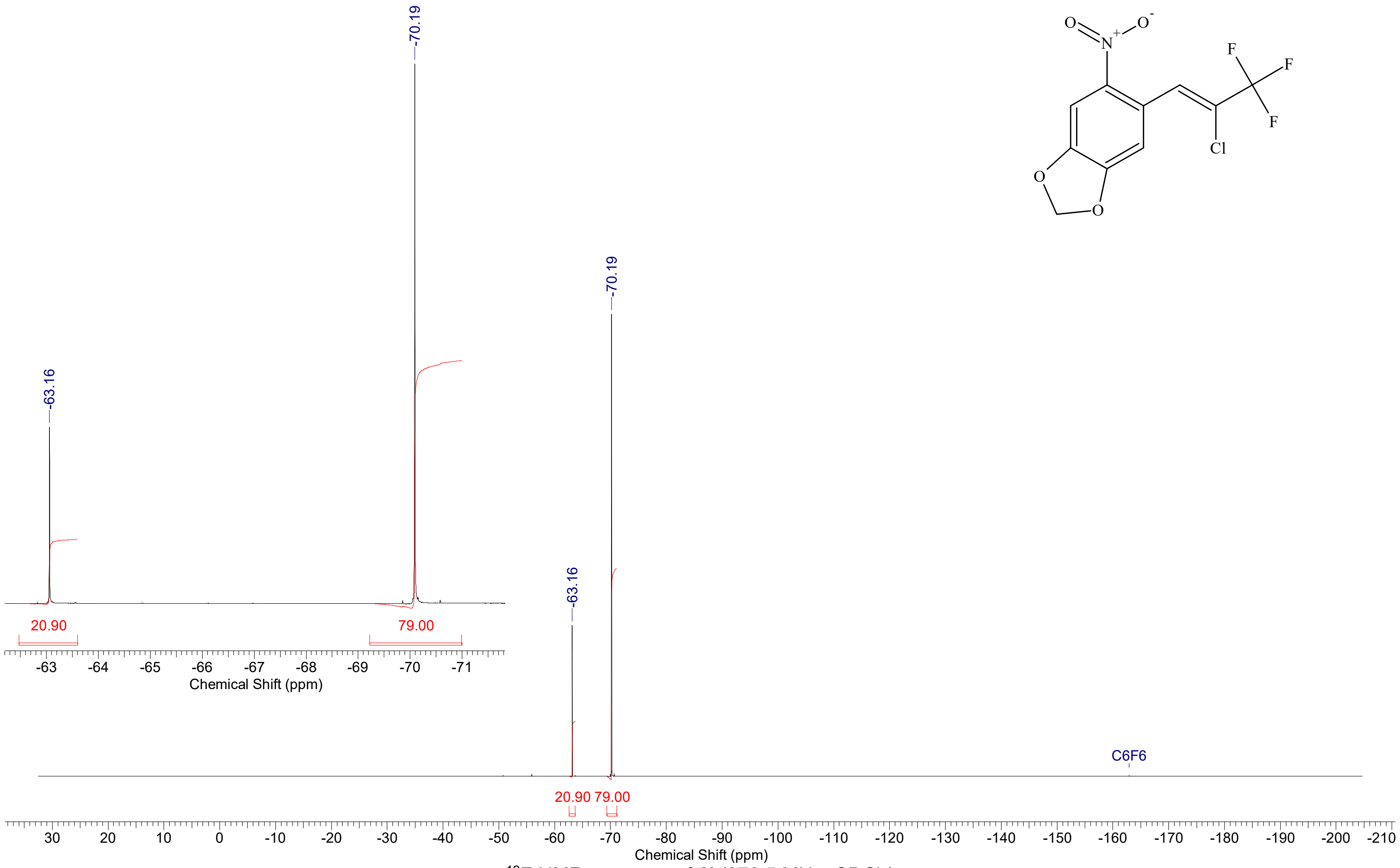

^{19}F NMR spectrum of **2i** (376.5 MHz, $CDCl_3$)

Temperature (degree C) 27.000

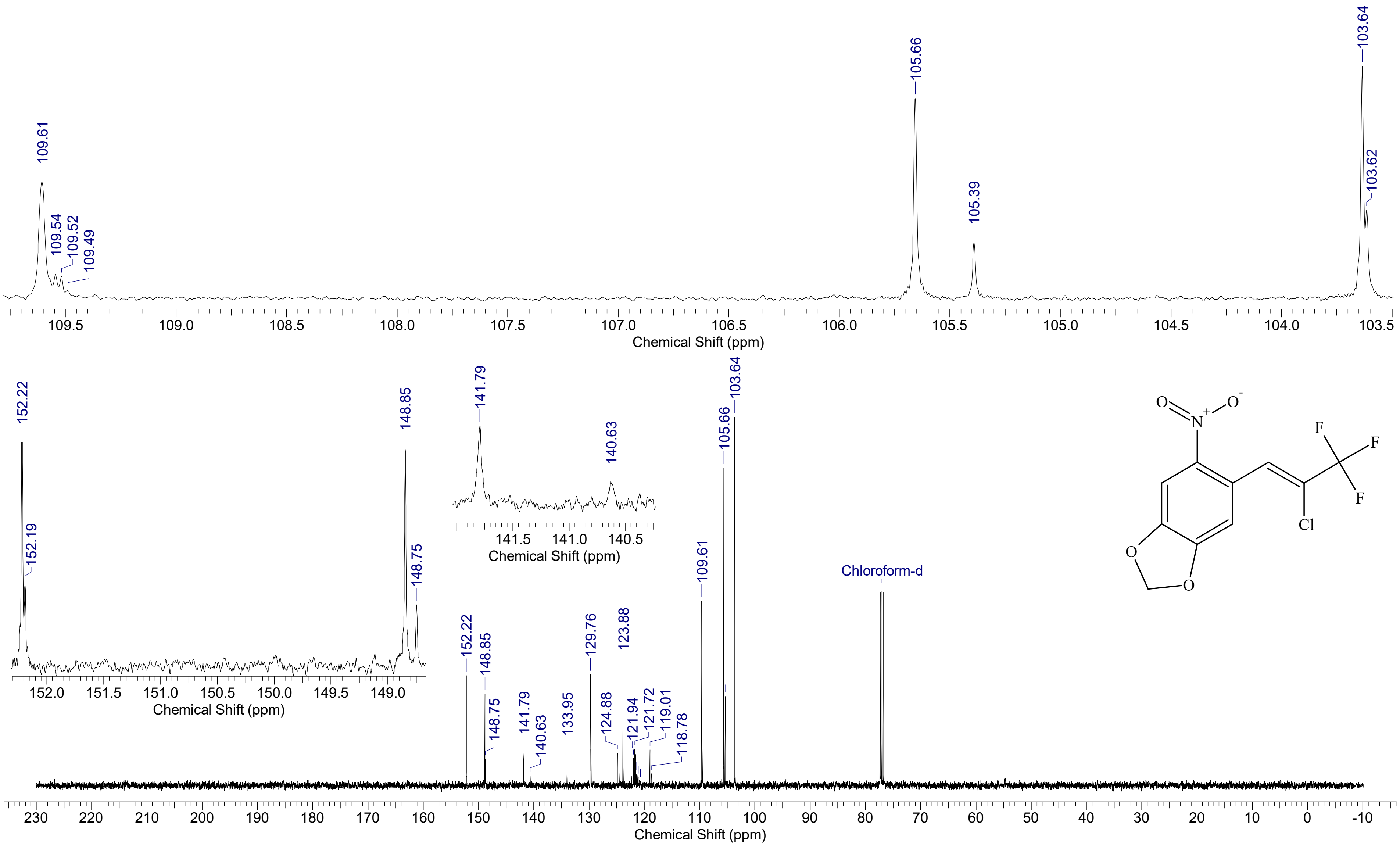

$^{13}C\{^{1}H\}$ NMR spectrum of **2i** (100.6 MHz, $CDCl_3$)

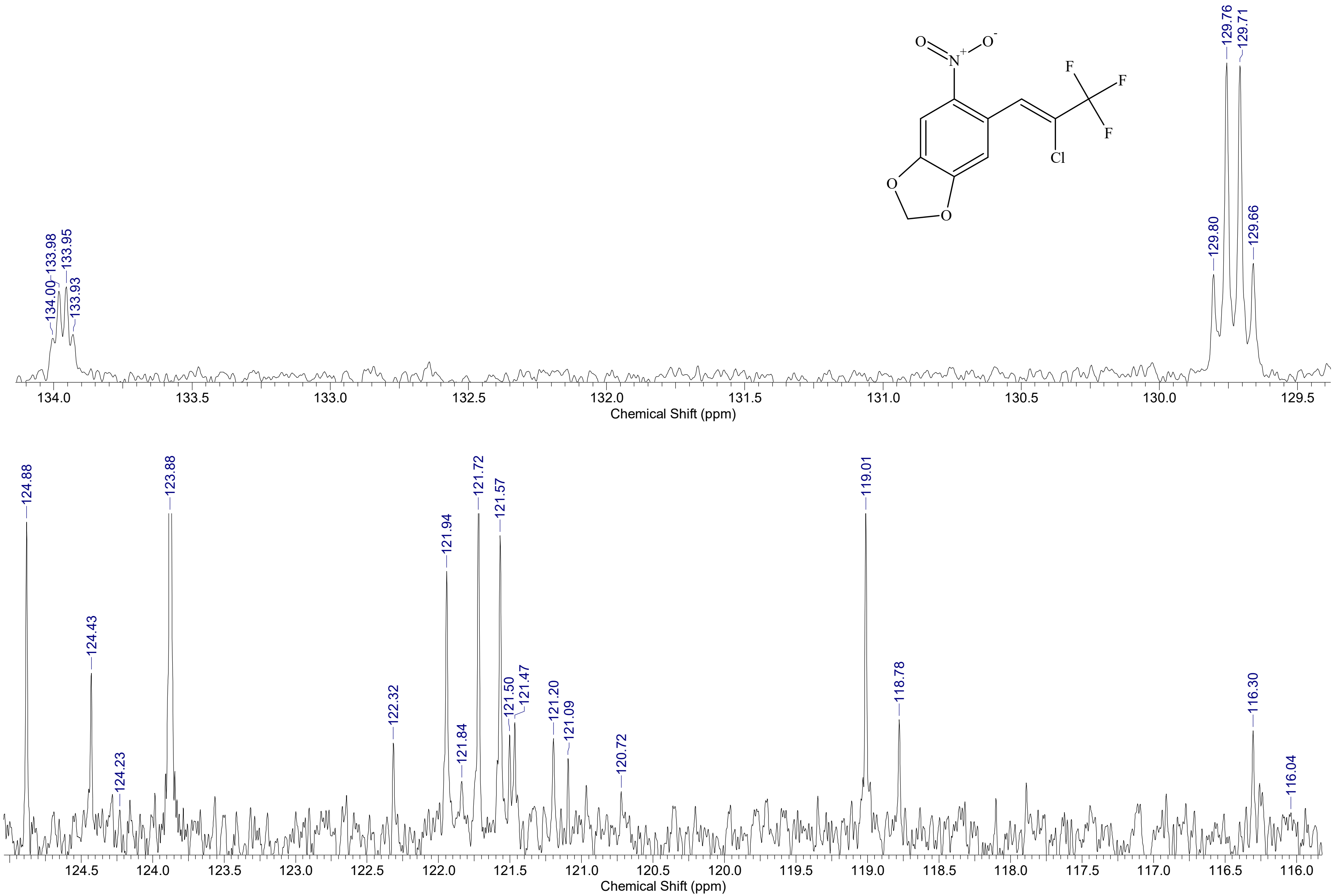

$^{13}C\{^{1}H\}$ NMR spectrum of **2i** (100.6 MHz, $CDCl_3$)

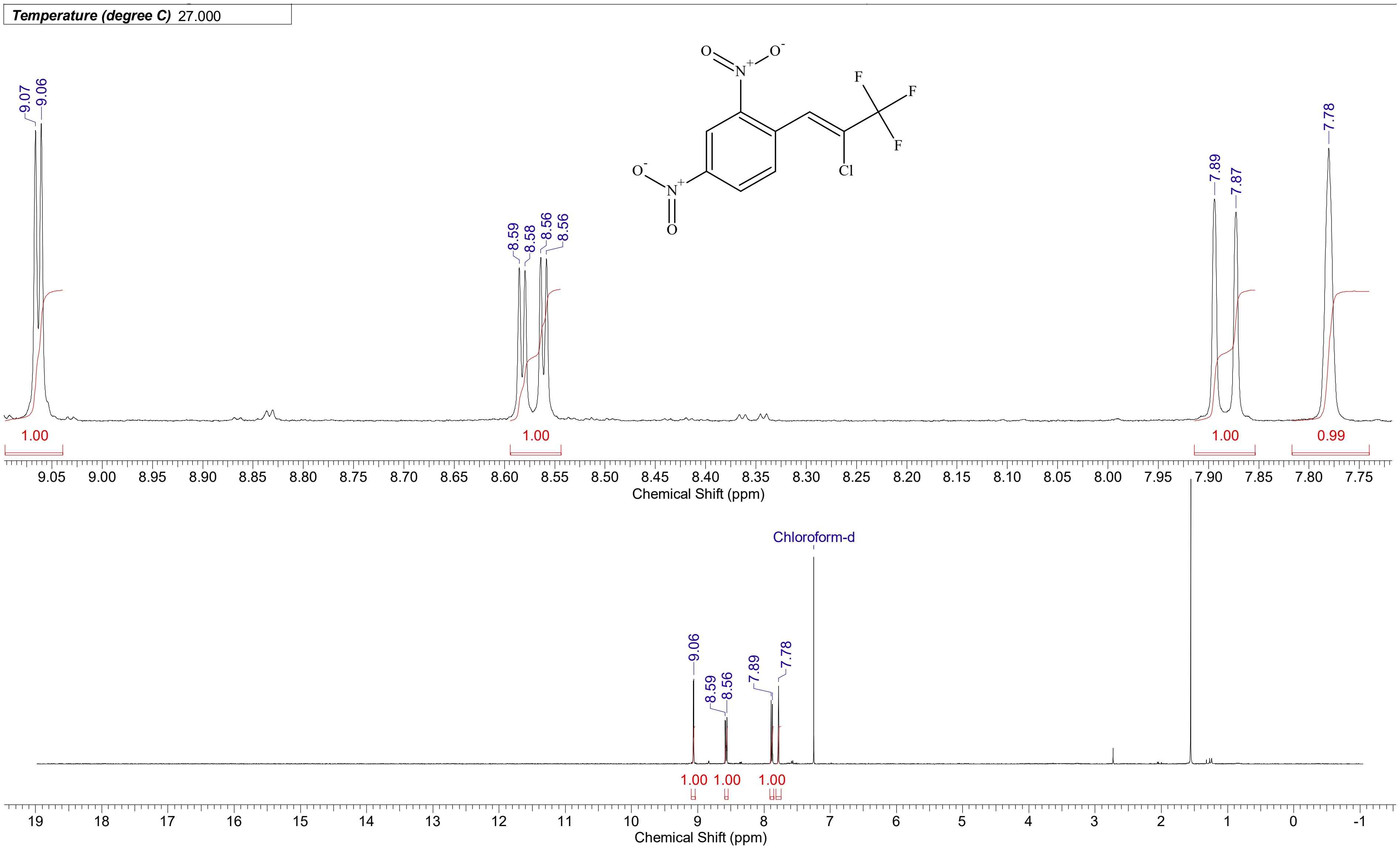

^{1}H NMR spectrum of **2j** (400.1 MHz, $CDCl_3$)

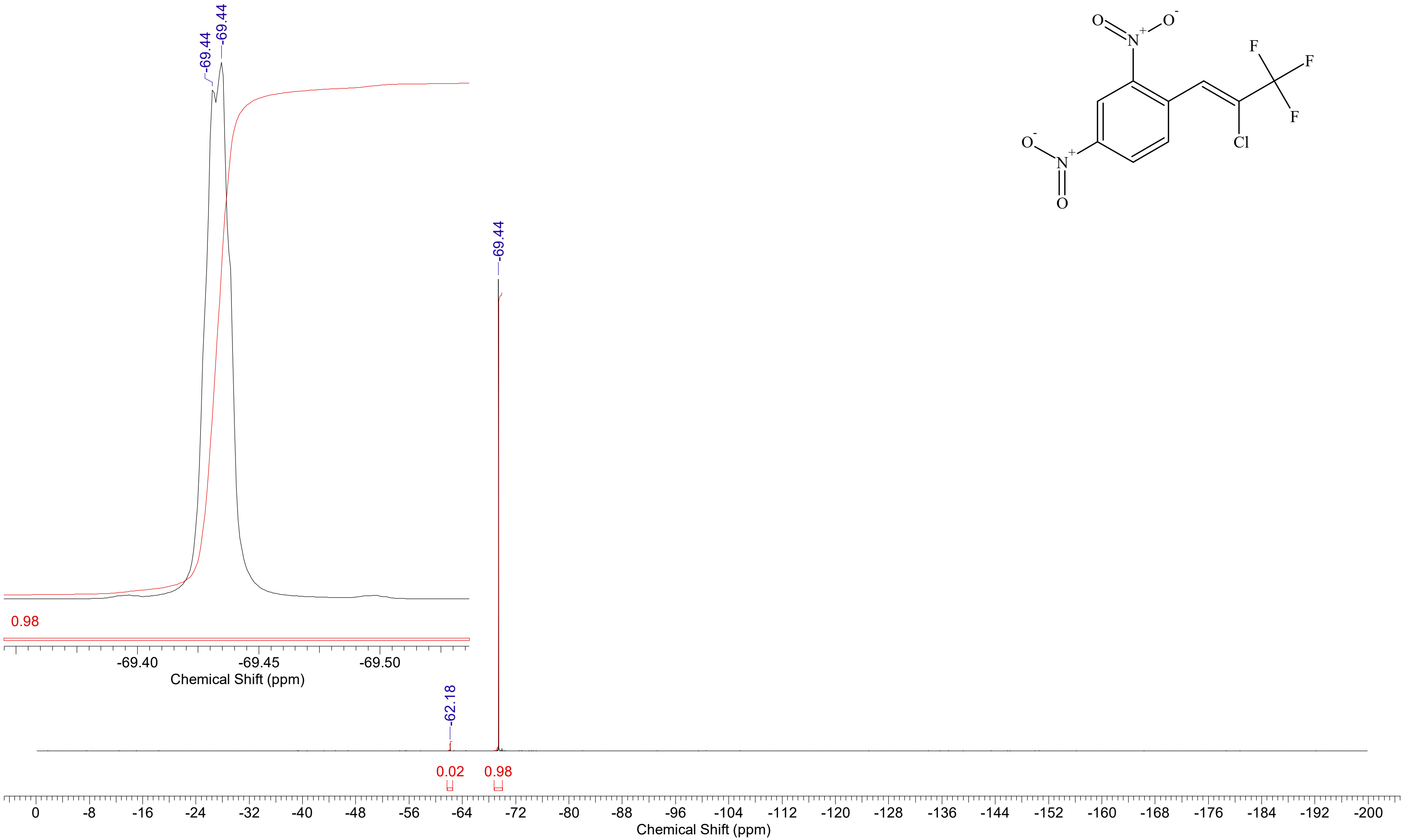

^{19}F NMR spectrum of **2j** (376.5 MHz $CDCl_3$)

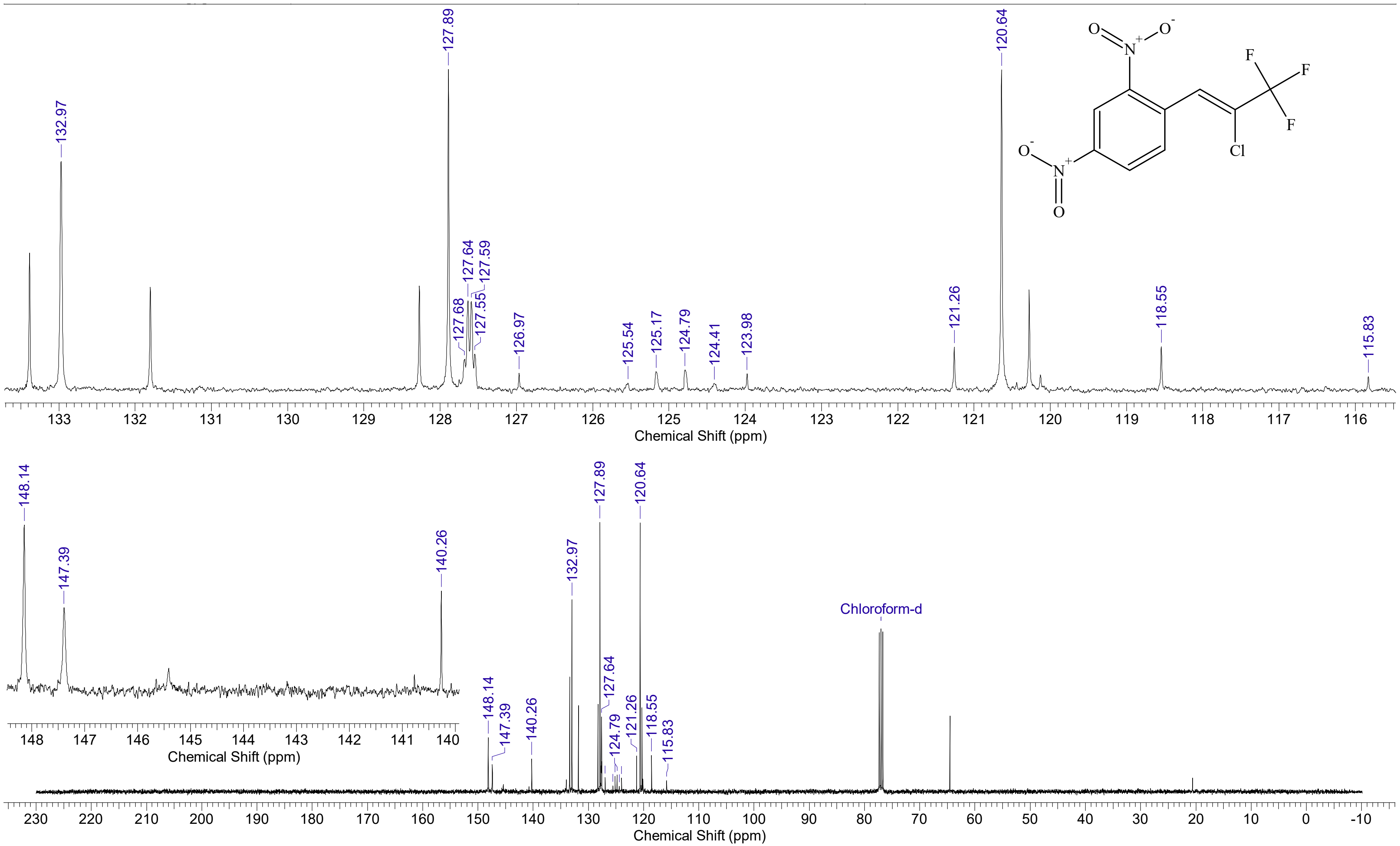

$^{13}C\{^{1}H\}$ NMR spectrum of **2j** (100.6 MHz, $CDCl_3$)

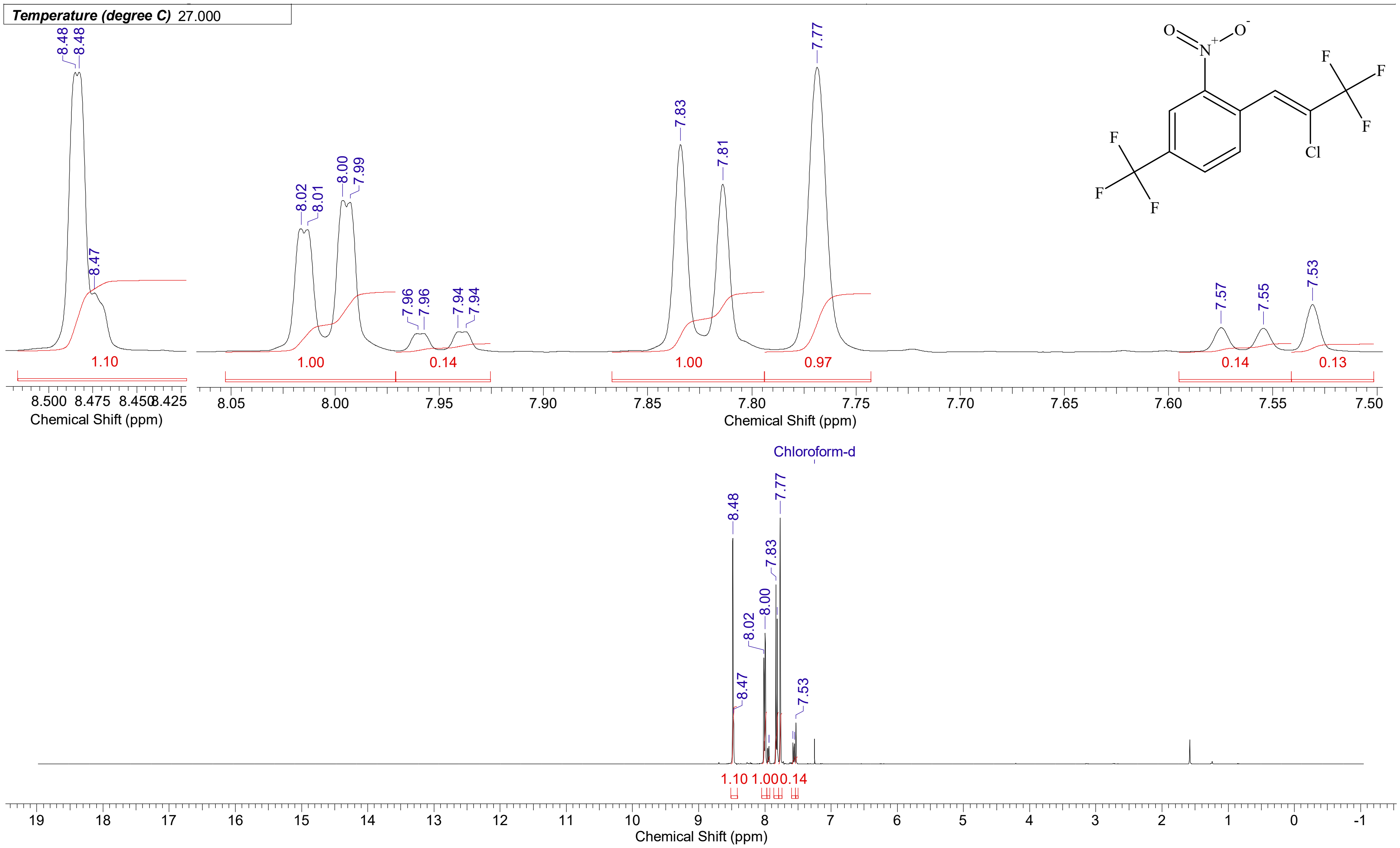

^{1}H NMR spectrum of **2k** (400.1 MHz, $CDCl_3$)

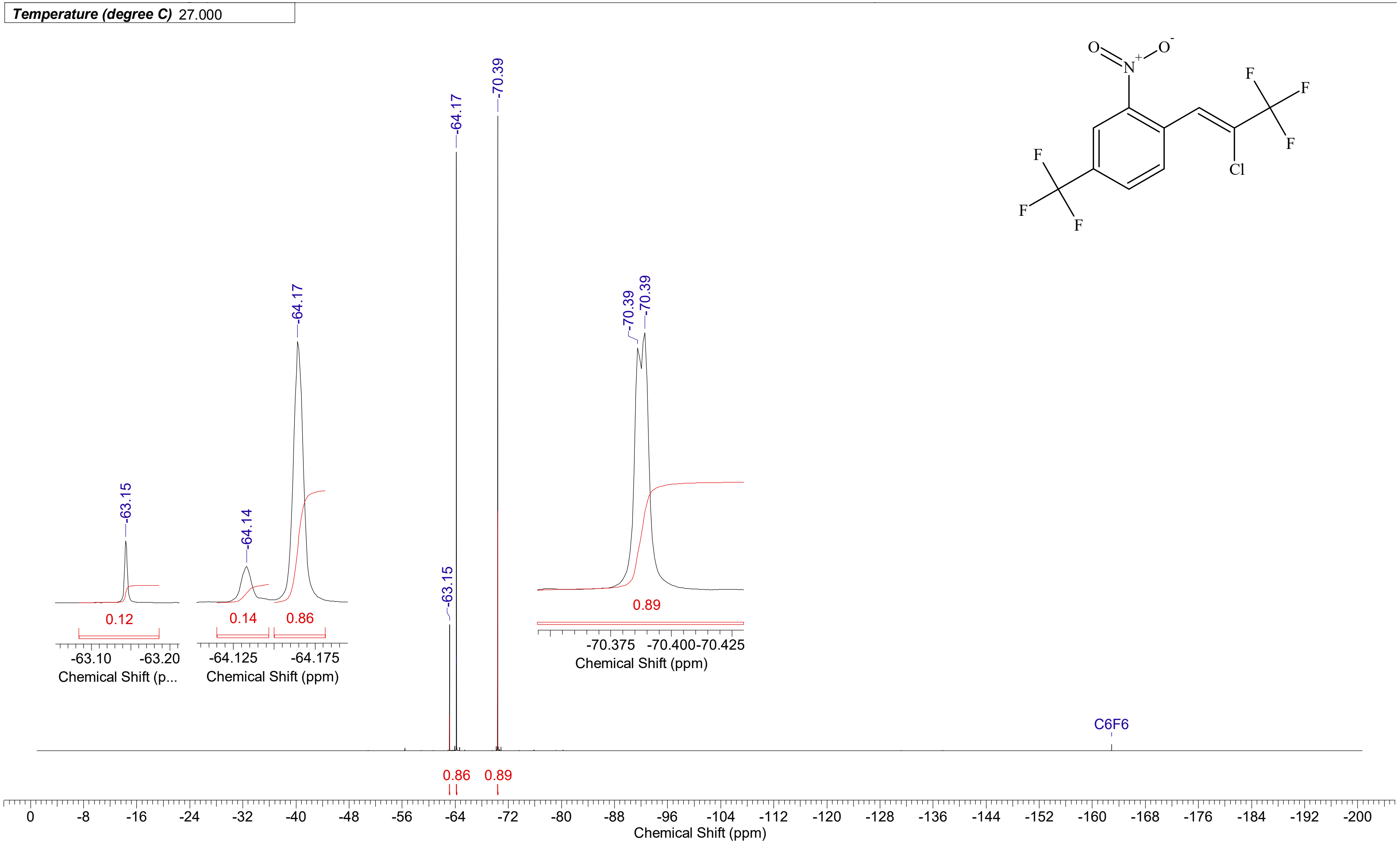

^{19}F NMR spectrum of **2k** (376.5 MHz, $CDCl_3$)

Temperature (degree C) 27.000

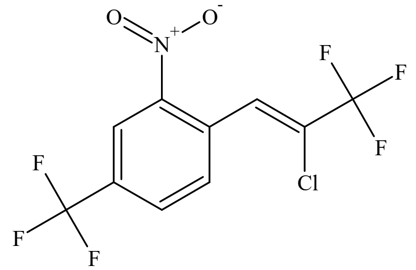

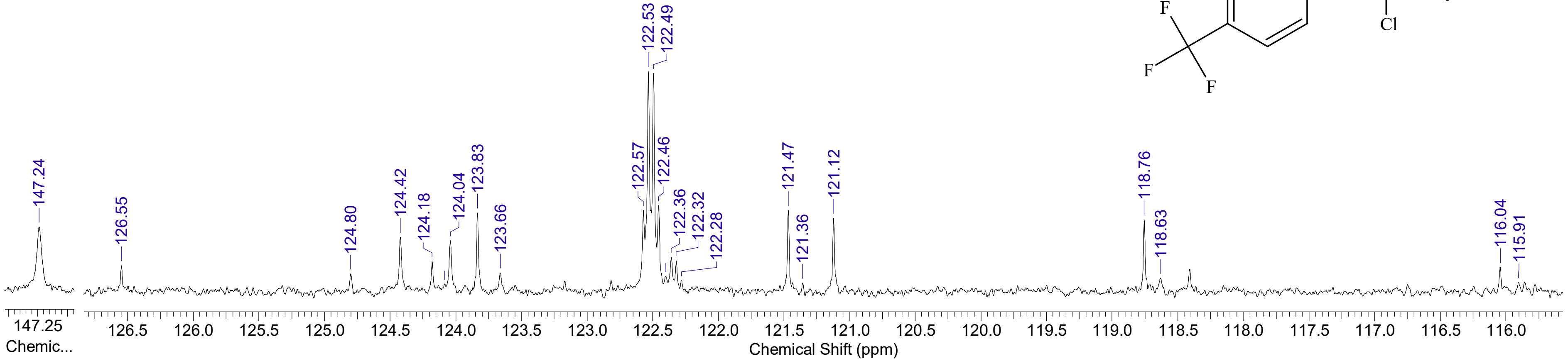

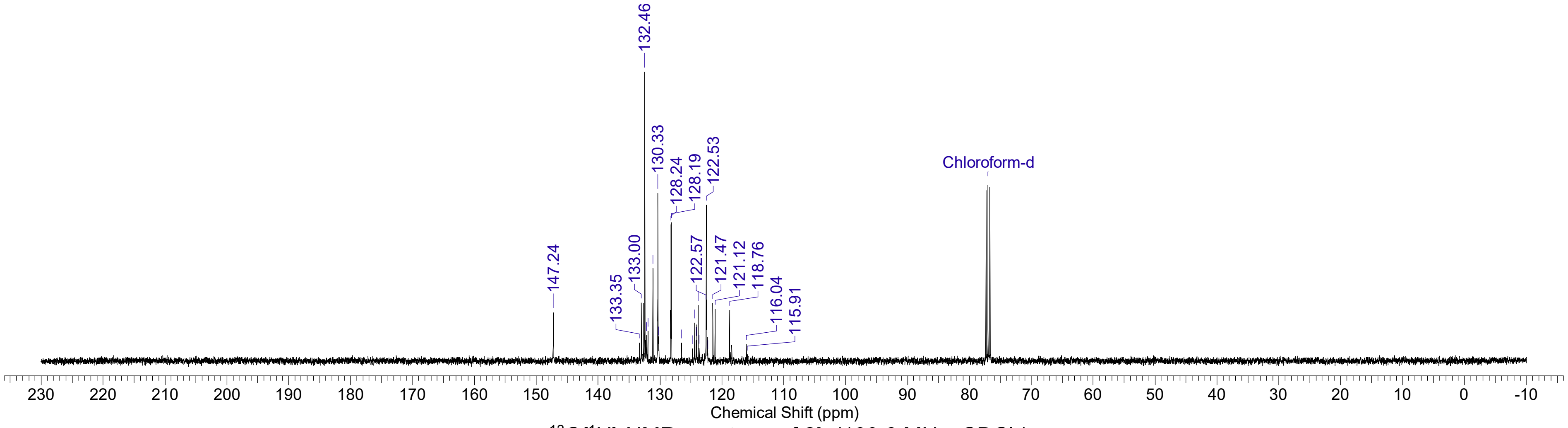

$^{13}C\{^{1}H\}$ NMR spectrum of **2k** (100.6 MHz, $CDCl_3$)

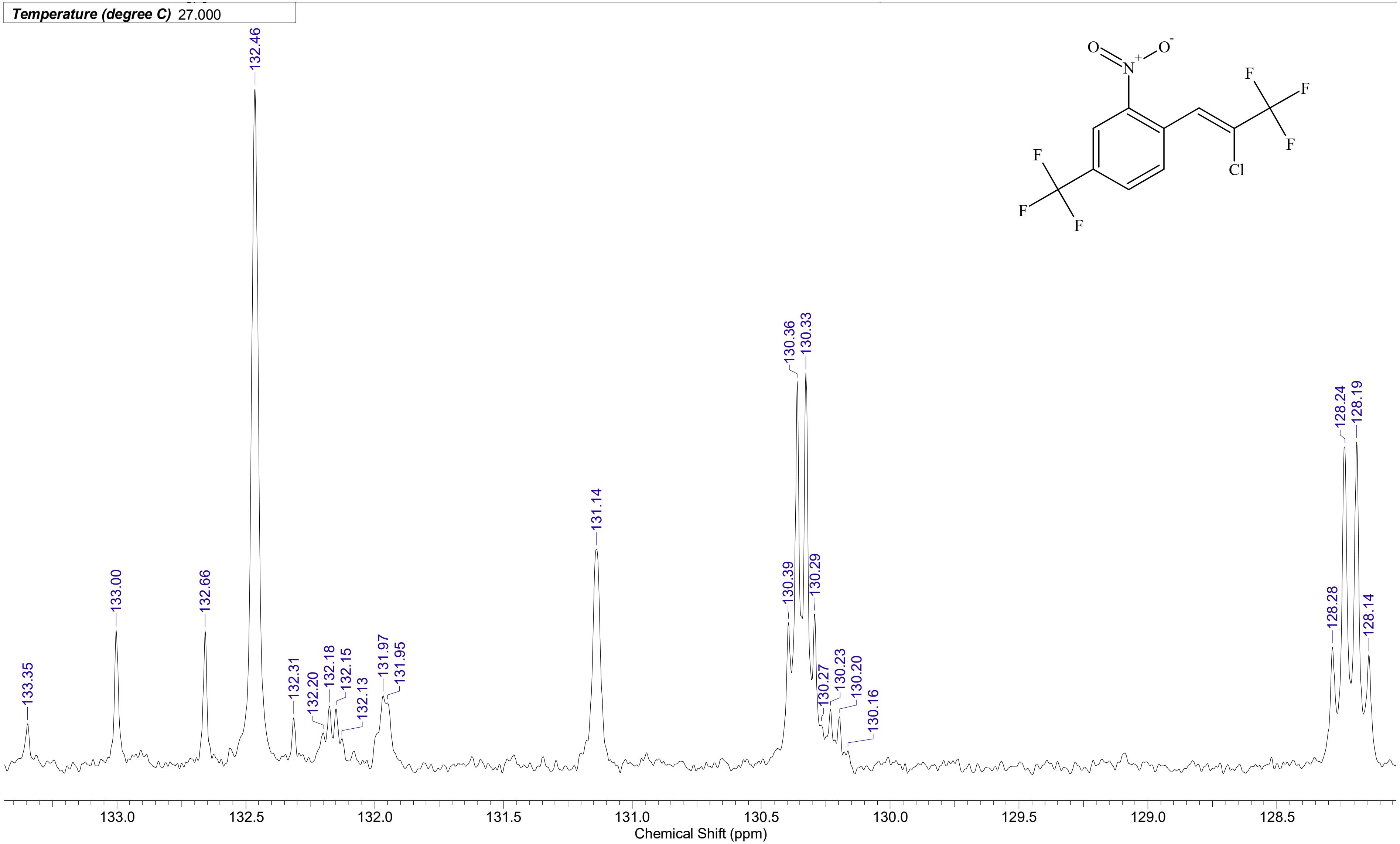

$^{13}C\{^{1}H\}$ NMR spectrum of **2k** (100.6 MHz, $CDCl_3$)

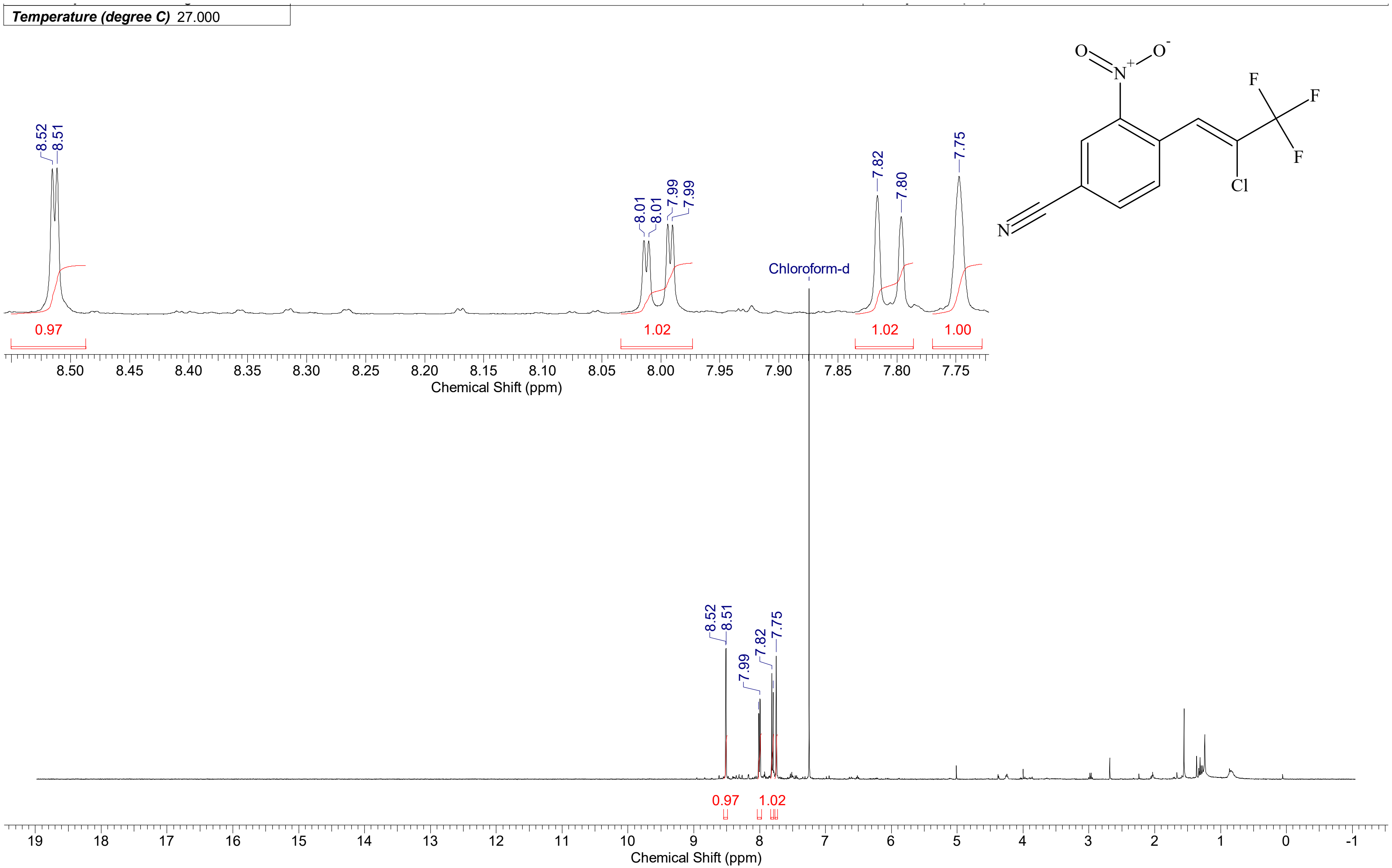

^{1}H NMR spectrum of **2l** (400.1 MHz, $CDCl_3$)

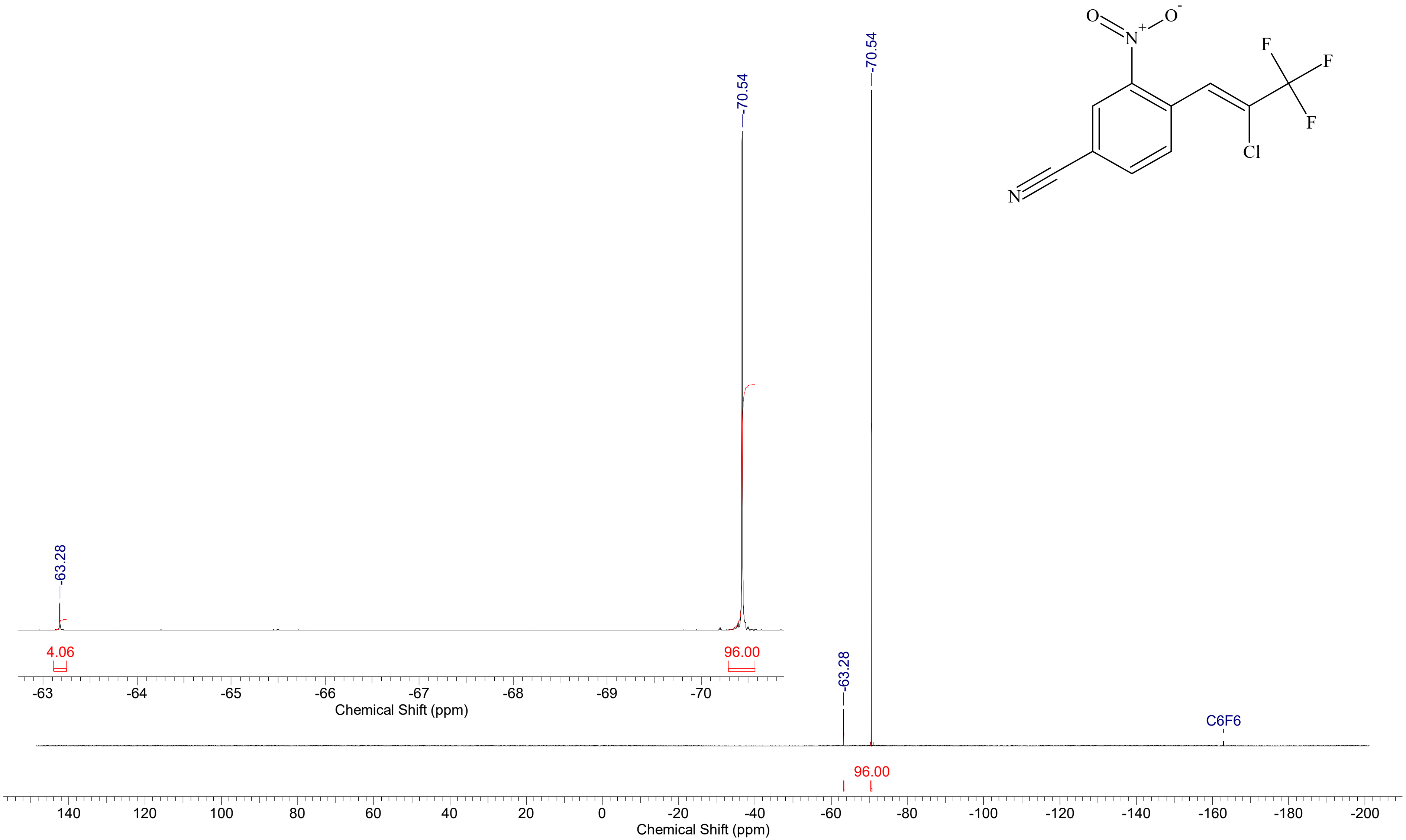

^{19}F NMR spectrum of **2l** (376.5 MHz, $CDCl_3$)

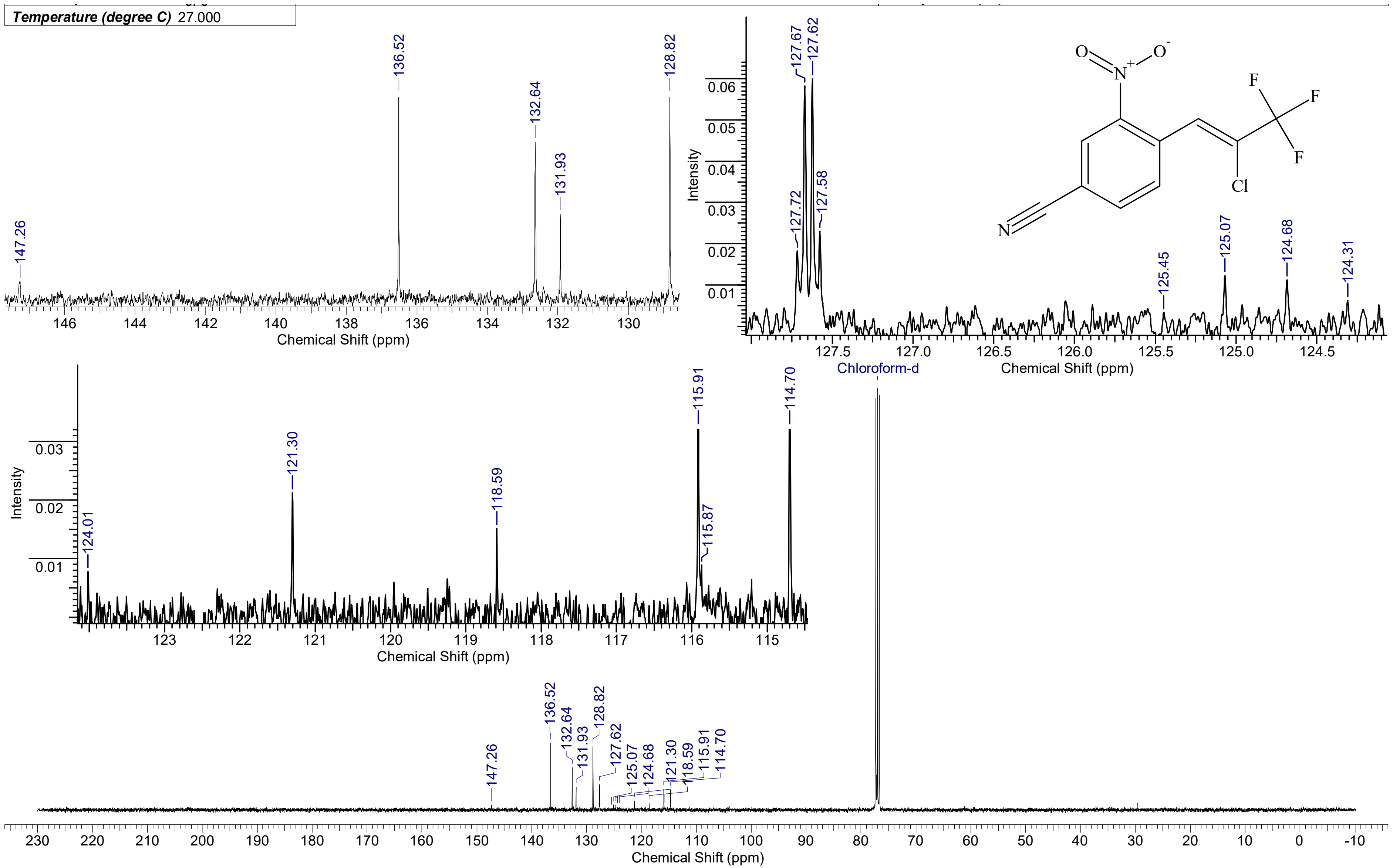

$^{13}C\{^{1}H\}$ NMR spectrum of **2l** (100.6 MHz, $CDCl_3$)

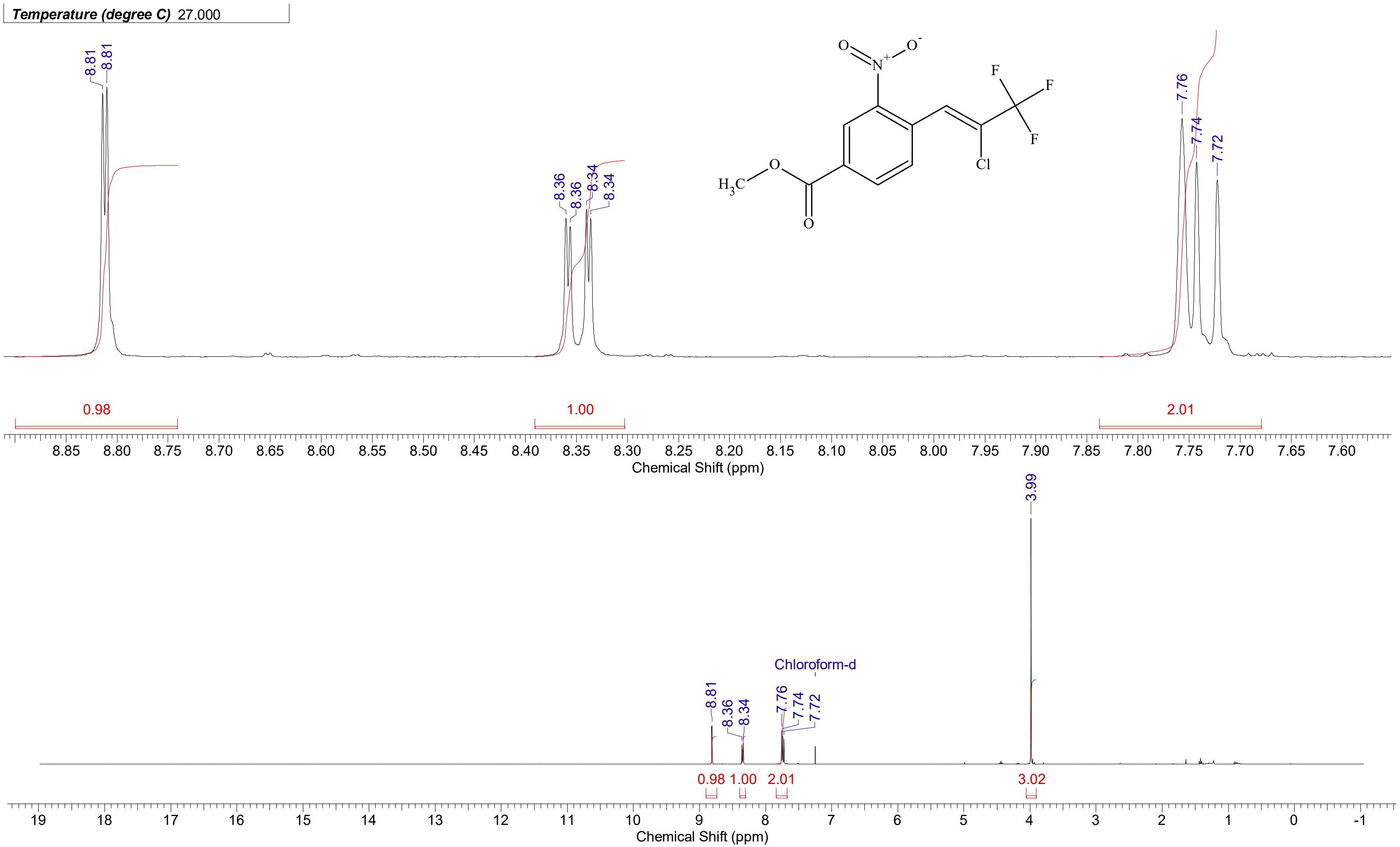

^{1}H NMR spectrum of **2m** (400.1 MHz, $CDCl_3$)

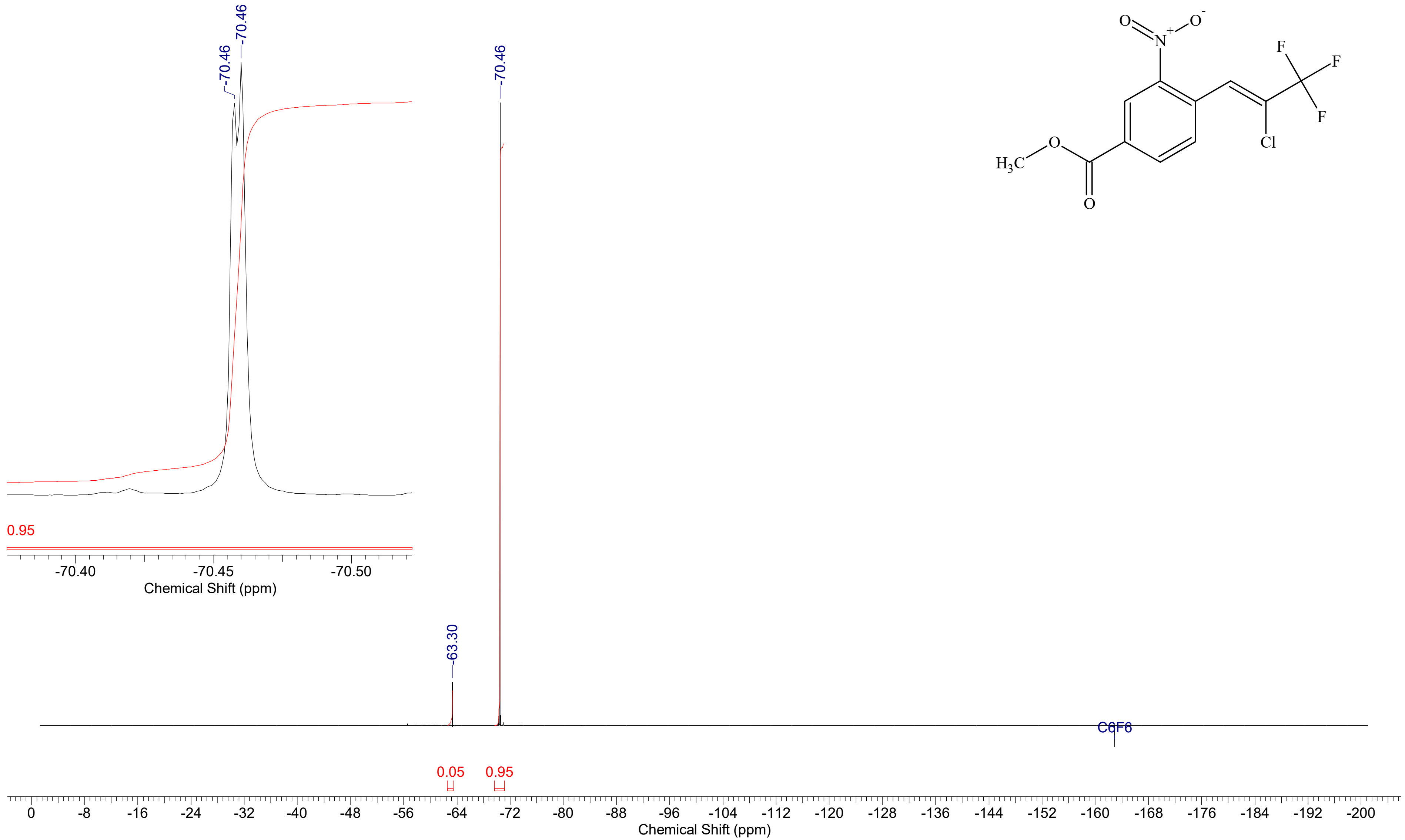

^{19}F NMR spectrum of **2m** (376.5 MHz, $CDCl_3$)

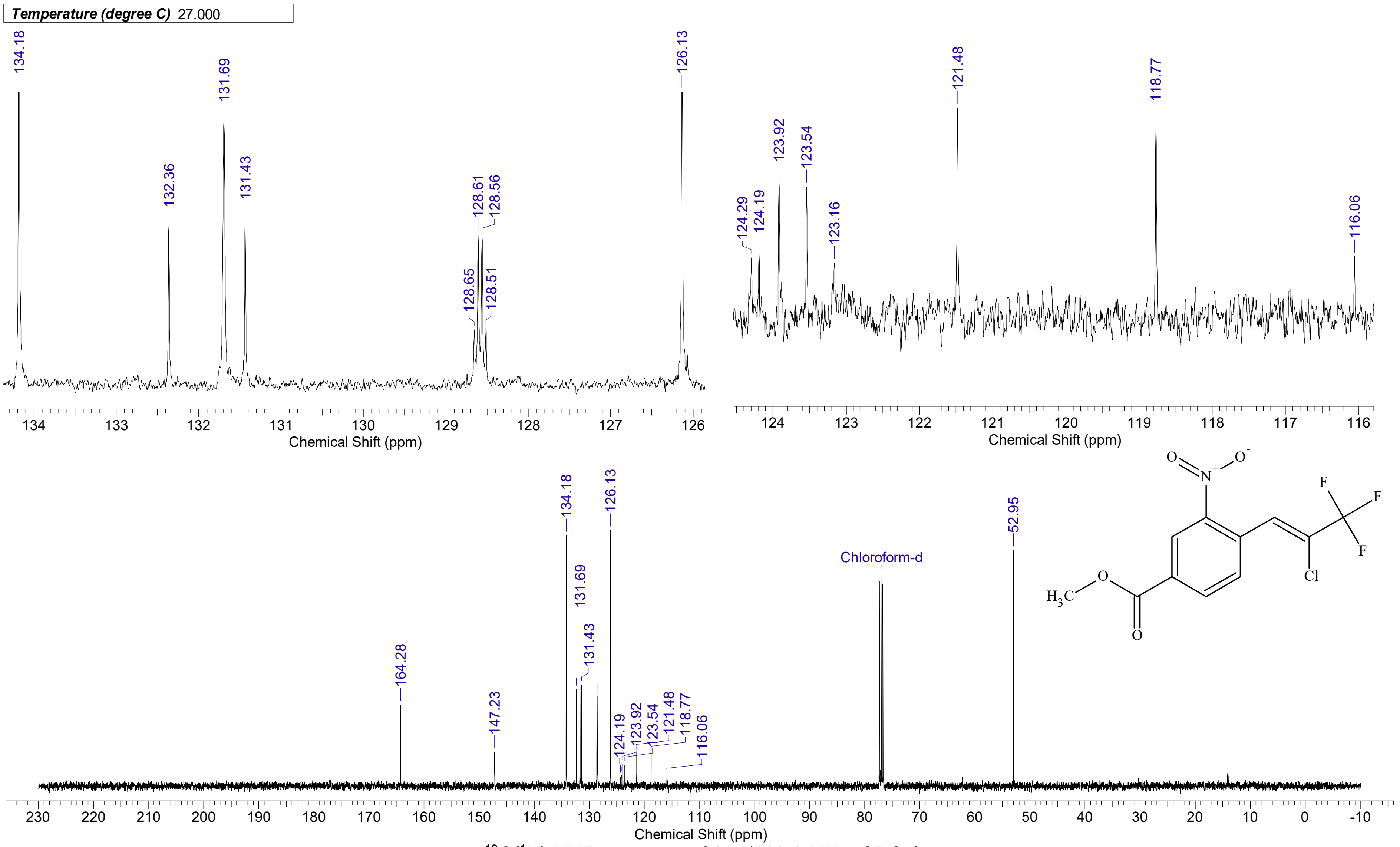

$^{13}C\{^{1}H\}$ NMR spectrum of **2m** (100.6 MHz, $CDCl_3$)

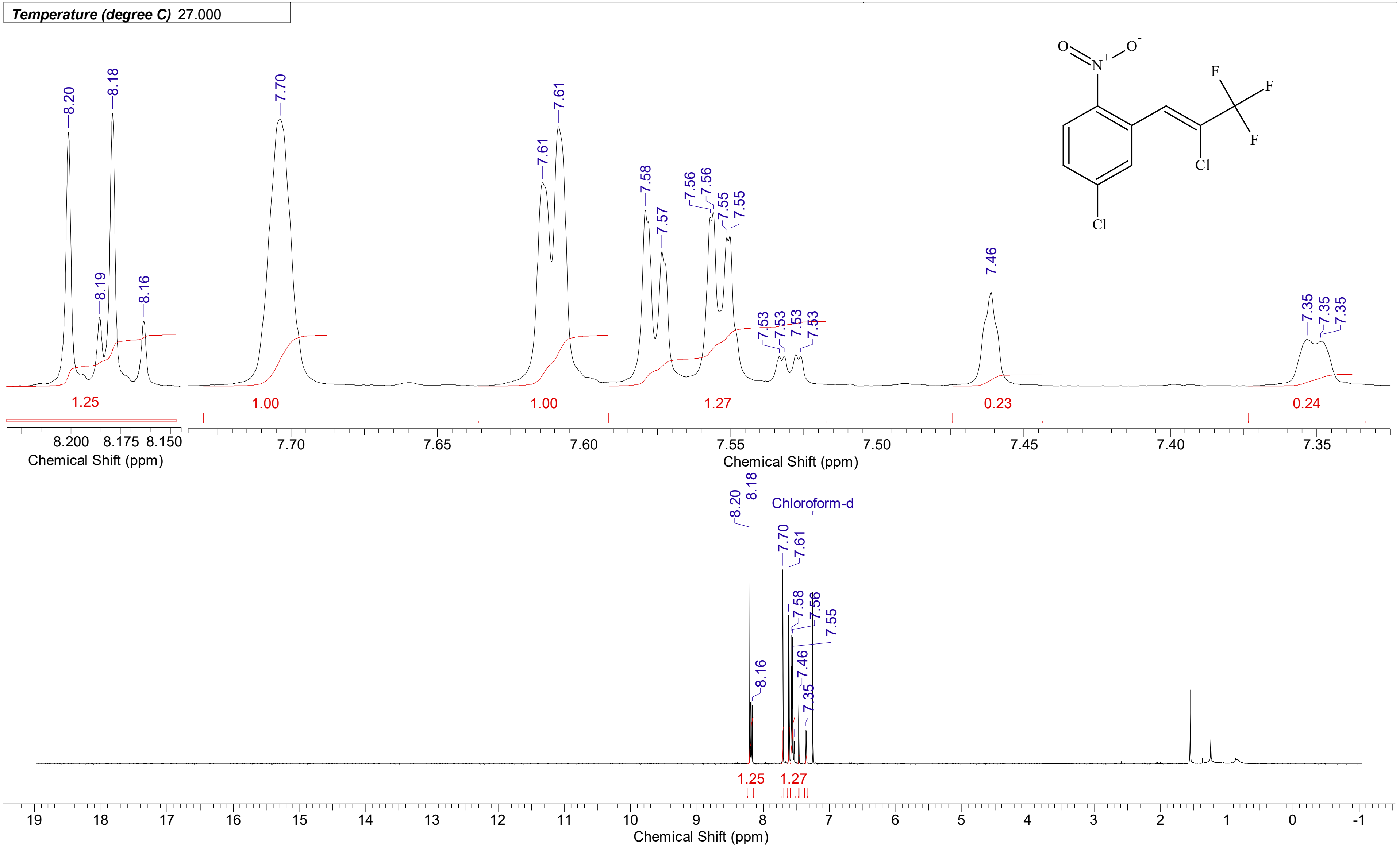

^{1}H NMR spectrum of **2n** (400.1 MHz, $CDCl_3$).

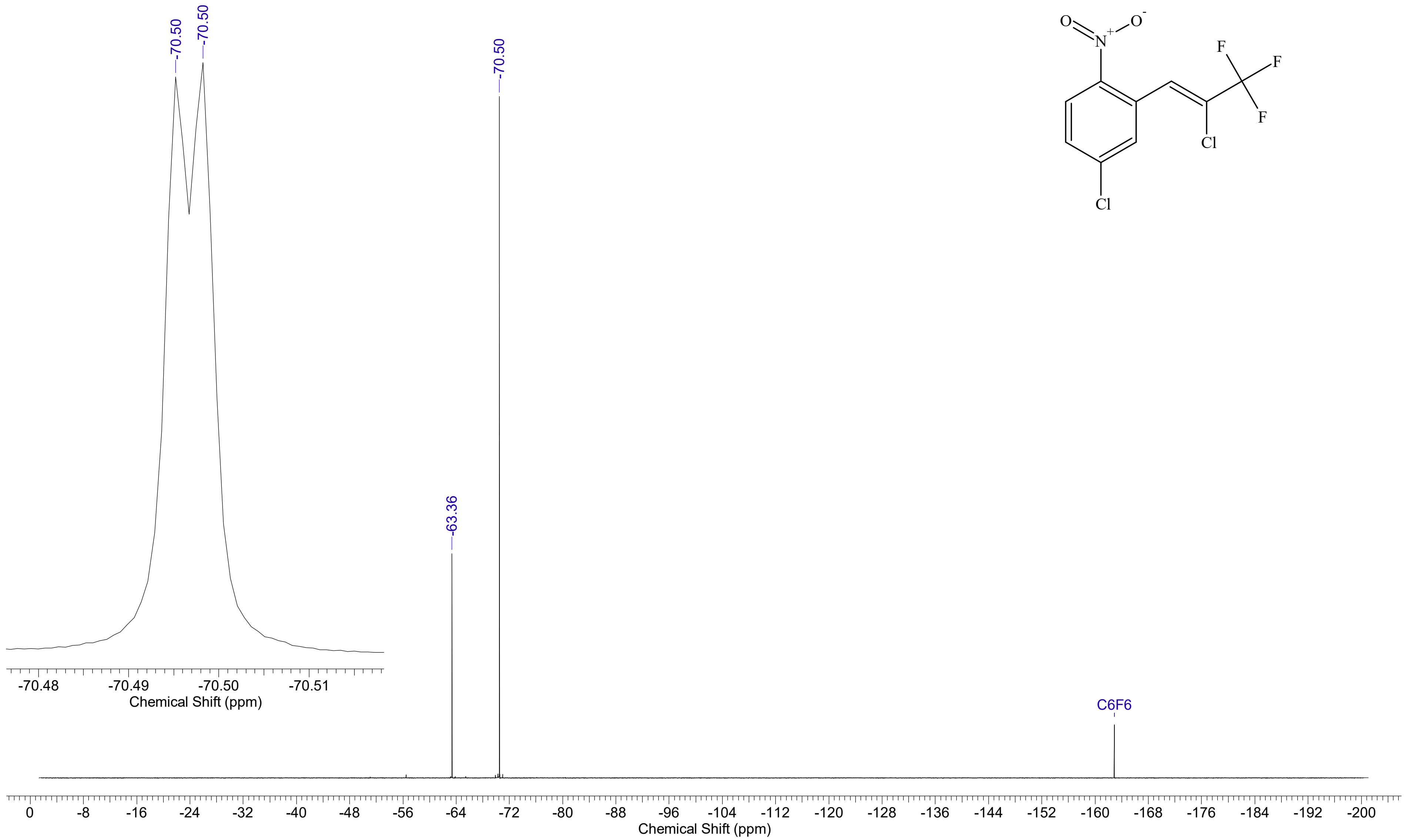

^{19}F NMR spectrum of **2n** (376.5 MHz, $CDCl_3$)

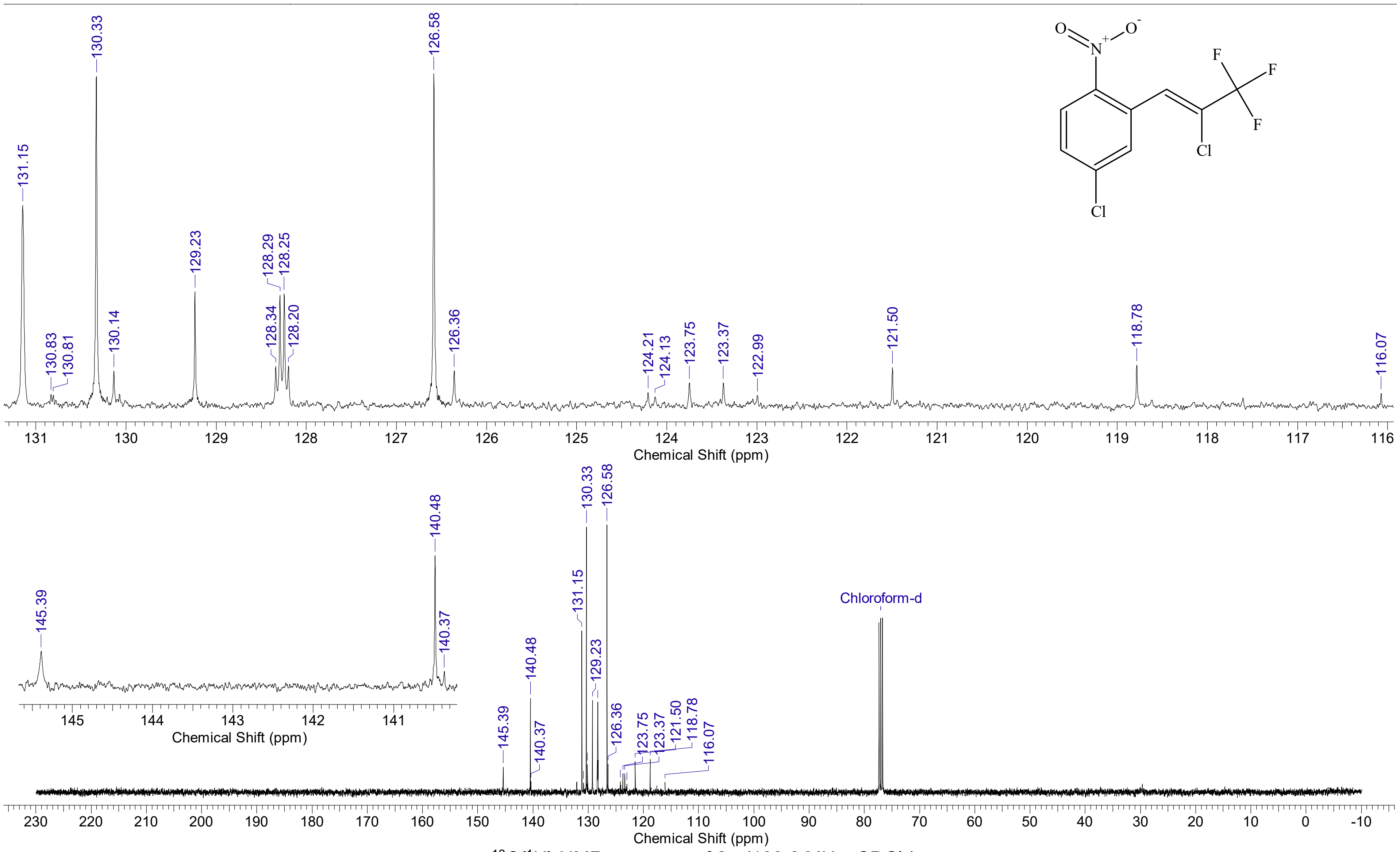

$^{13}C\{^1H\}$ NMR spectrum of **2n** (100.6 MHz, $CDCl_3$)

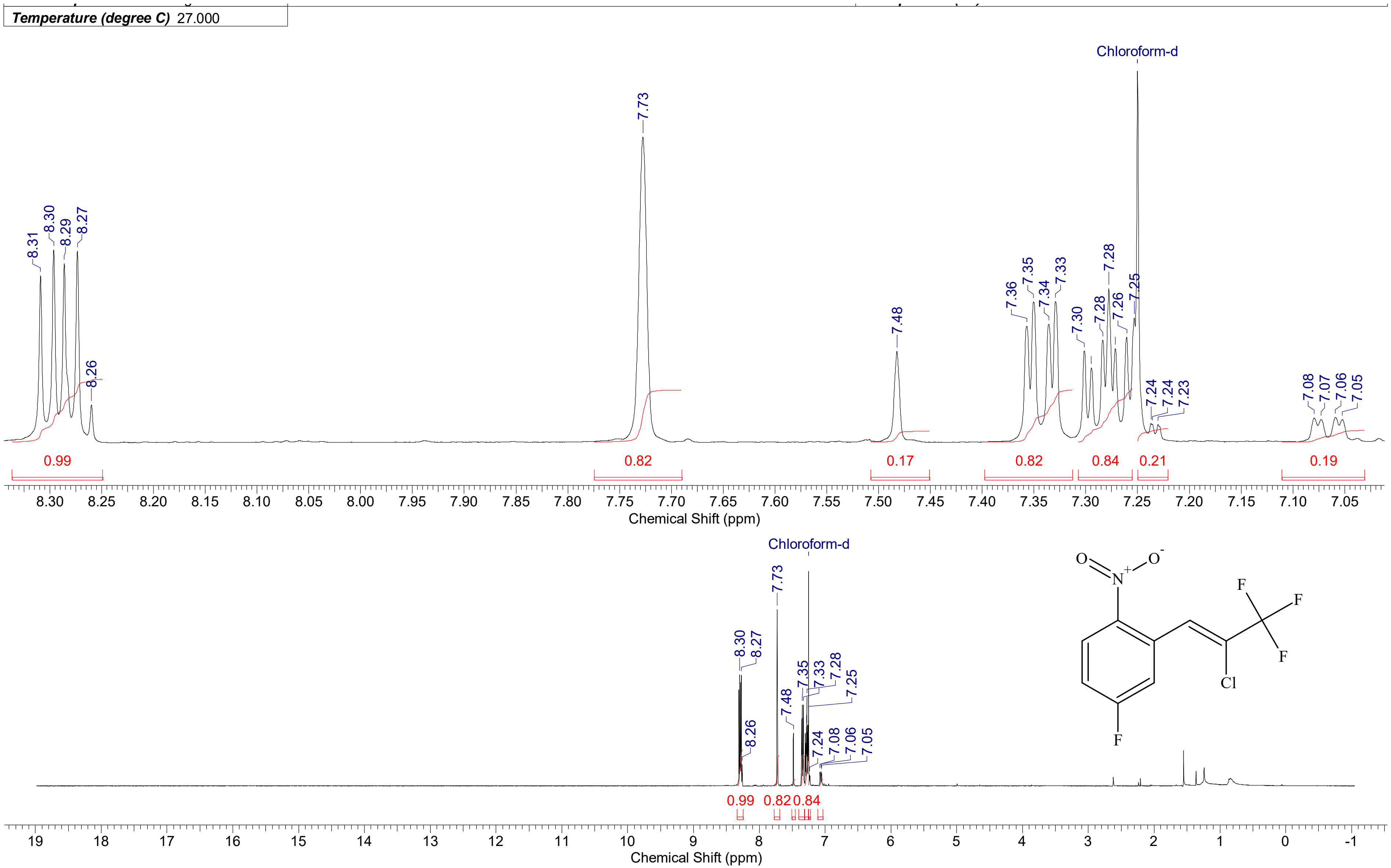

^{1}H NMR spectrum of **2o** (400.1 MHz, $CDCl_3$)

Acquisition Time (sec)	2.0000	***Date***	Dec 16 2019	***File Name***	C:\DOCS\OUTPUT_301\F19\2019.12.16\SZA-169_20191216_01\FLUORINE_01
Frequency (MHz)	376.31	***Nucleus***	19F	***Number of Transients***	16 ***Original Points Count*** 178571
Points Count	262144	***Pulse Sequence***	s2pul	***Solvent***	CHLOROFORM-D
Sweep Width (Hz)	89285.71	***Temperature (degree C)***	30.000		

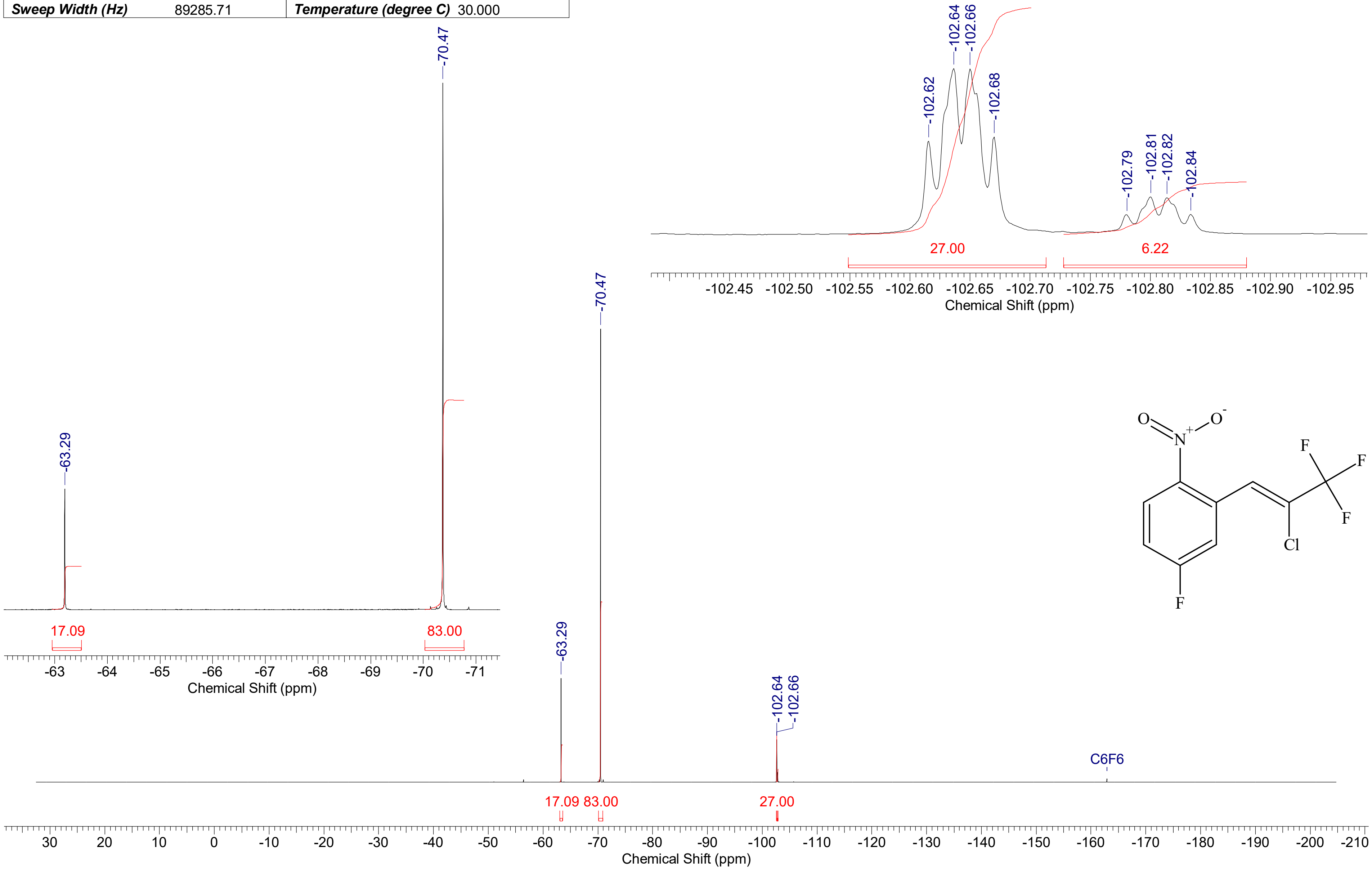

^{19}F NMR spectrum of **2o** (376.5 MHz, $CDCl_3$)

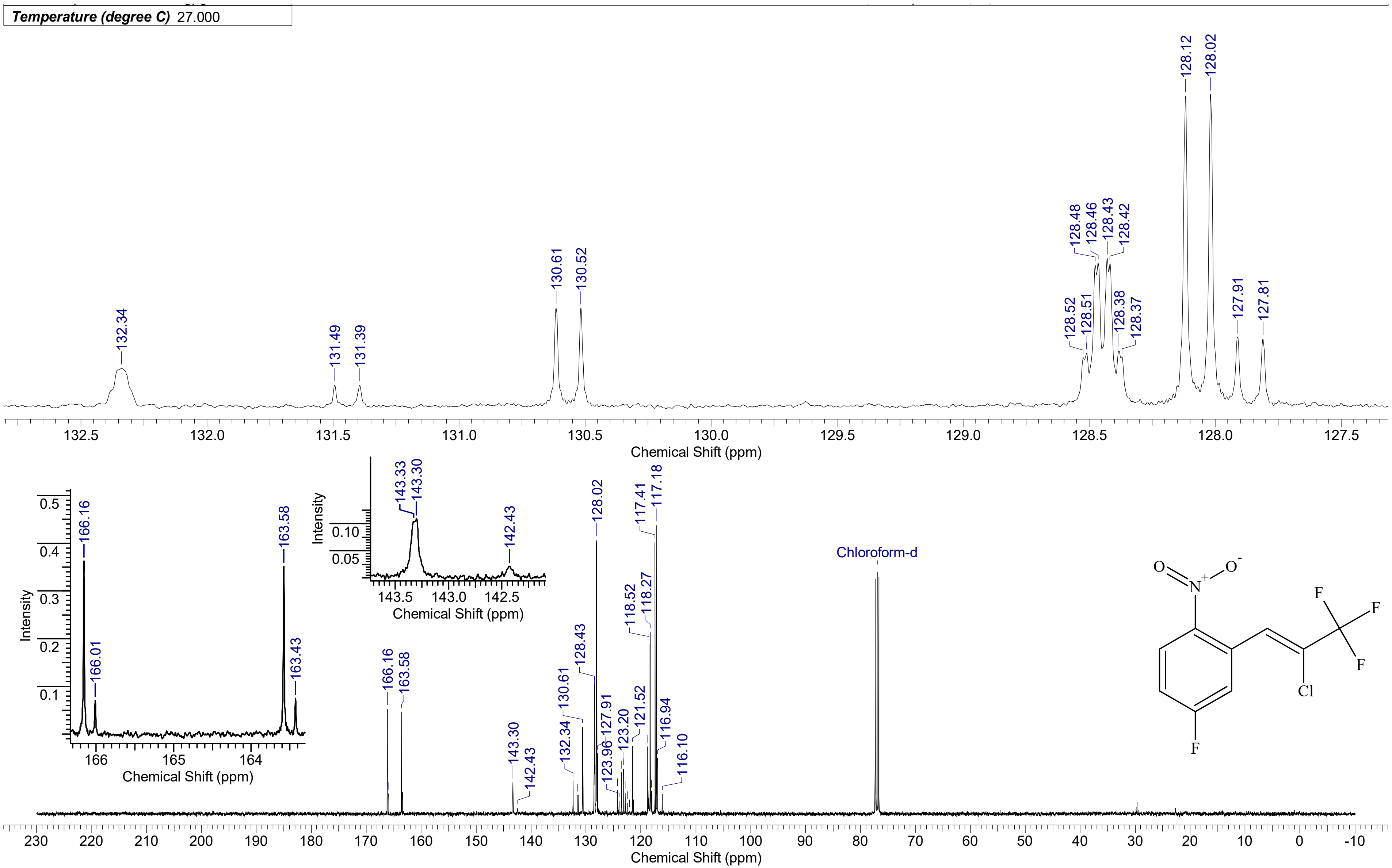

$^{13}C\{^{1}H\}$ NMR spectrum of **2o** (100.6 MHz, $CDCl_3$)

Temperature (degree C)	27.000

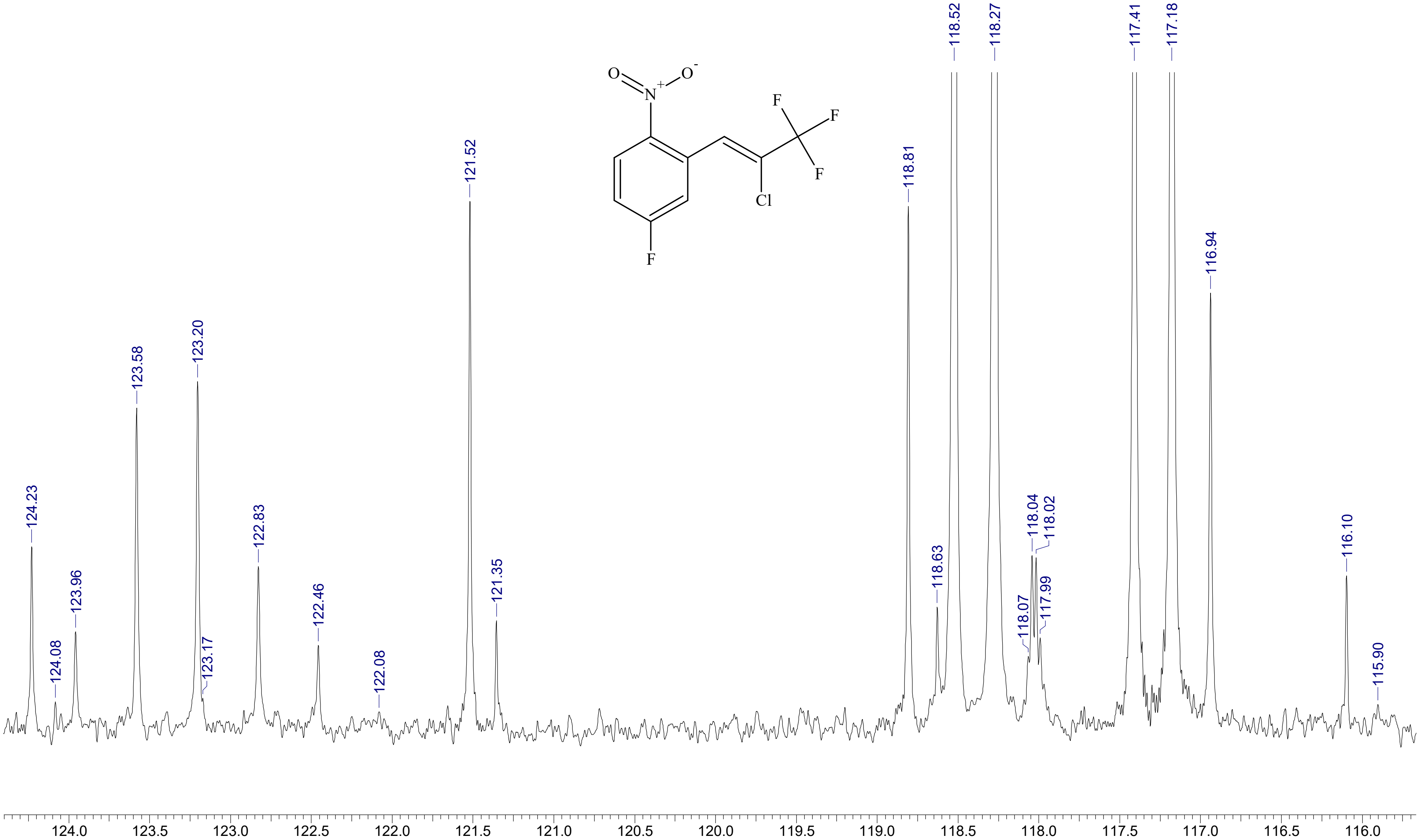

$^{13}C\{^{1}H\}$ NMR spectrum of **2o** (100.6 MHz, $CDCl_3$)

Temperature (degree C) 27.000

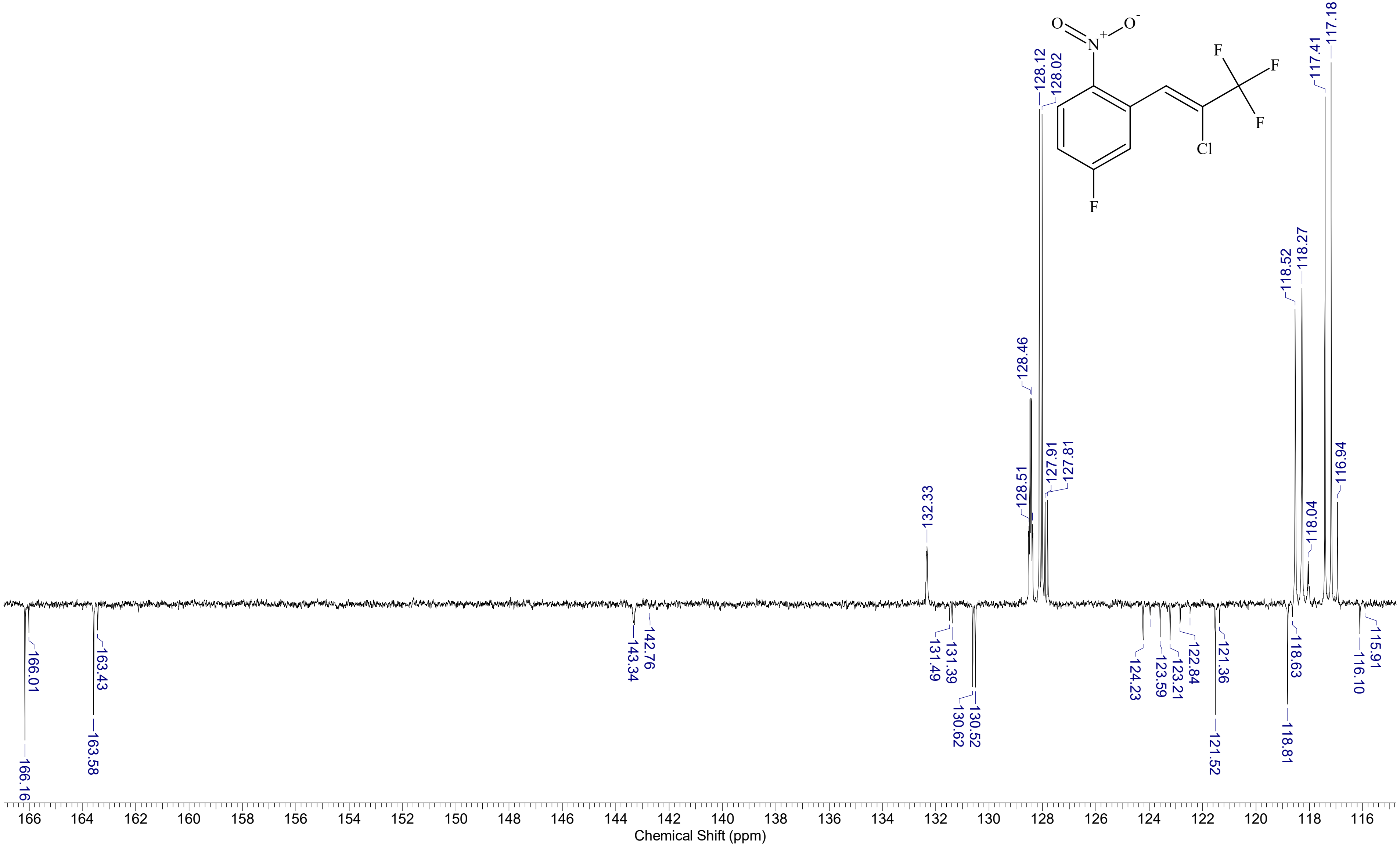

$^{13}C\{^{1}H\}$ APT NMR spectrum of **2o** (100.6 MHz, $CDCl_3$)

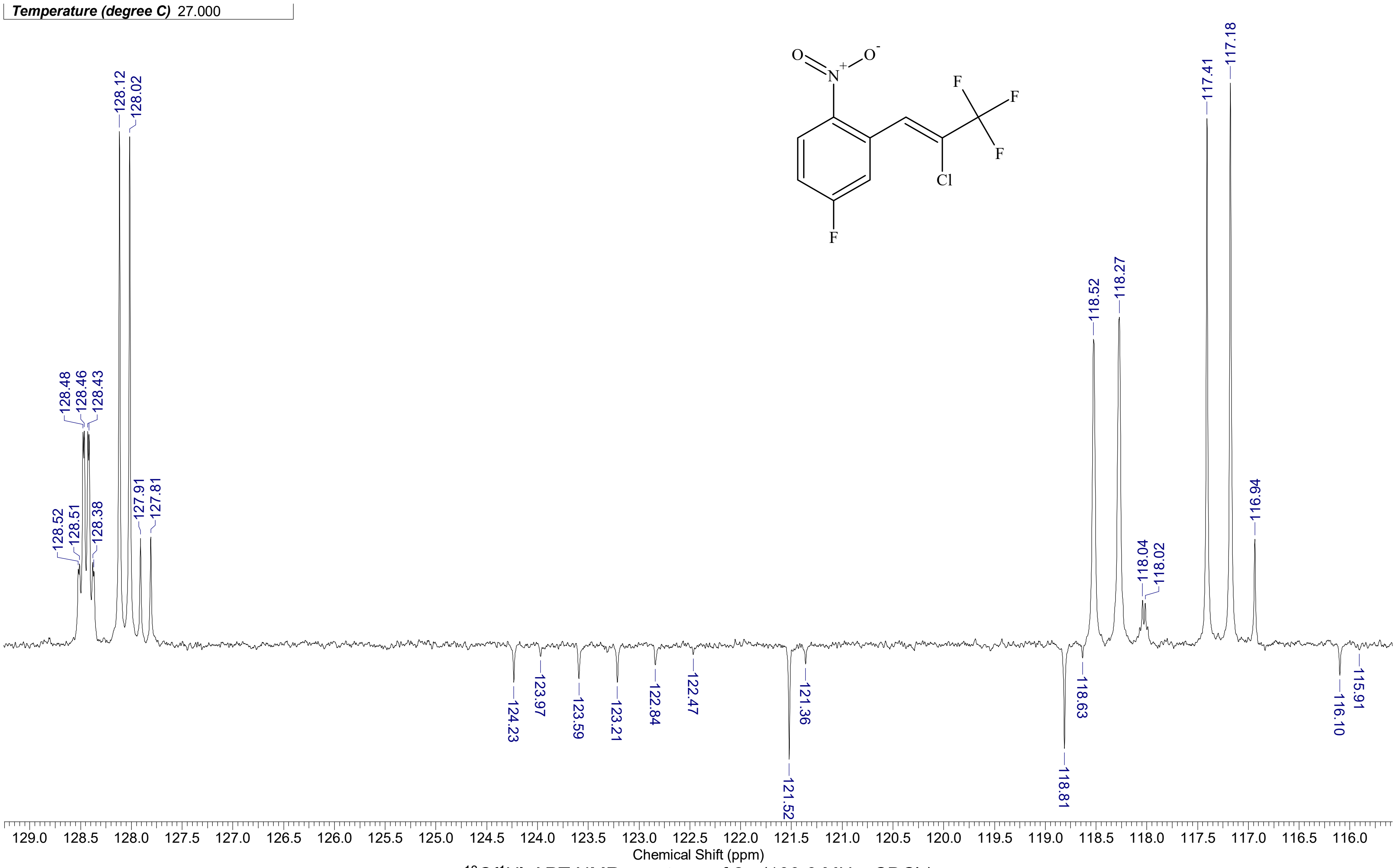

$^{13}C\{^{1}H\}$ APT NMR spectrum of **2o** (100.6 MHz, $CDCl_3$)

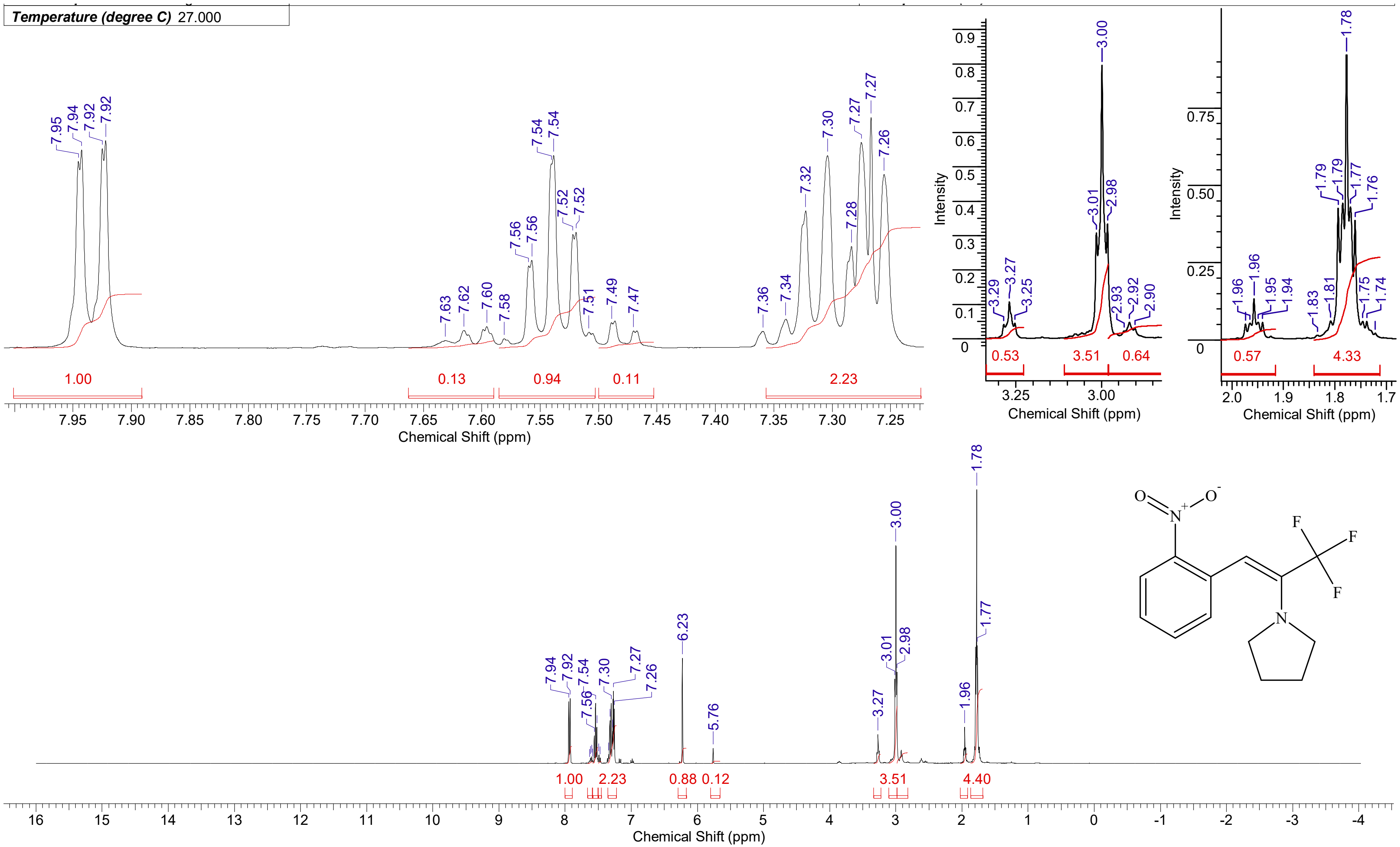

^{1}H NMR spectrum of **3a** (400.1 MHz, $CDCl_3$)

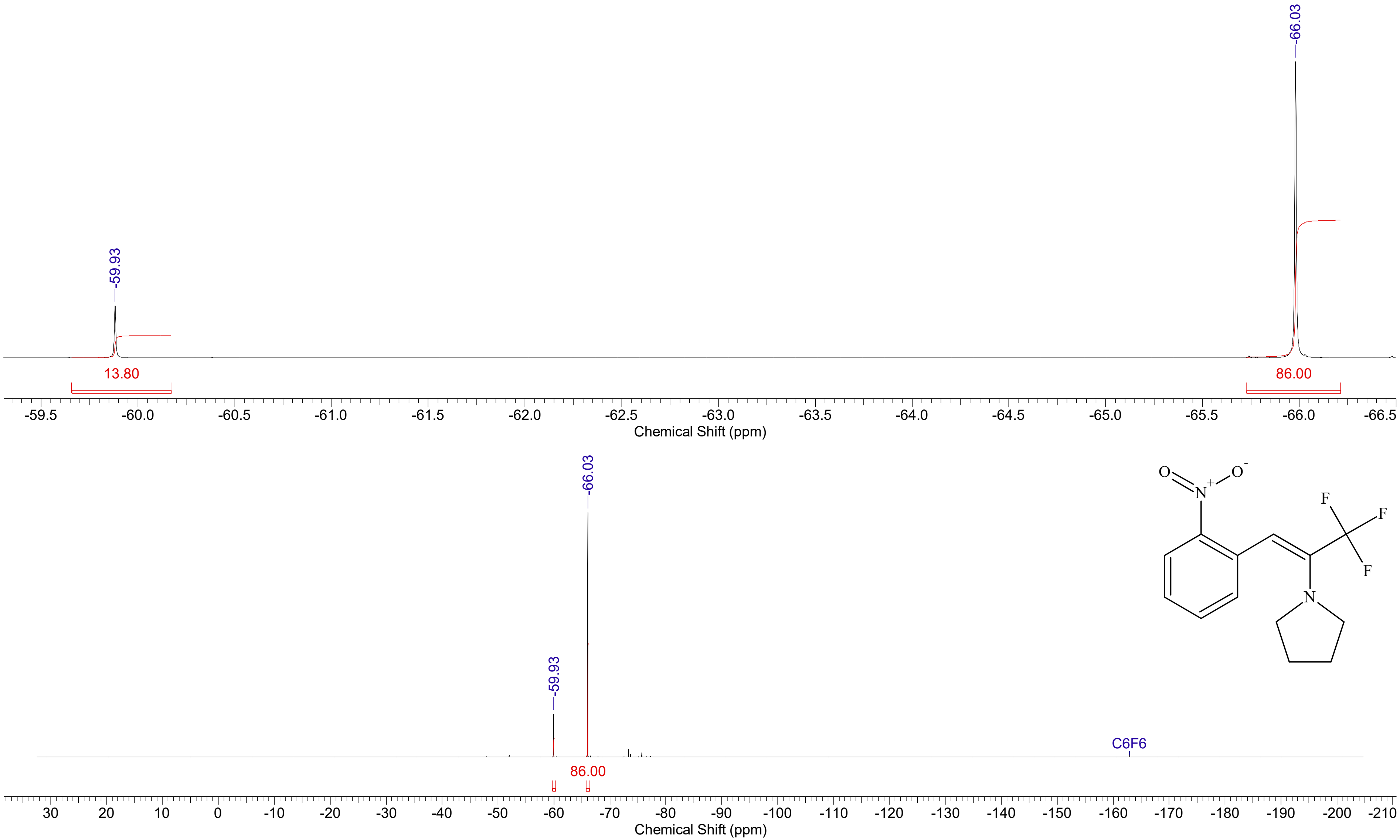

^{19}F NMR spectrum of **3a** (376.5 MHz, $CDCl_3$)

Nucleus	1H	Number of Transients	4	Original Points Count	32768	Points Count	131072
Pulse Sequence	zg30	Solvent	CHLOROFORM-D			Sweep Width (Hz)	8012.82
Temperature (degree C)	27.000						

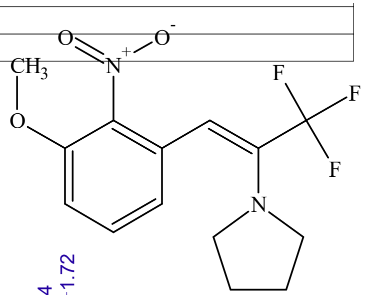

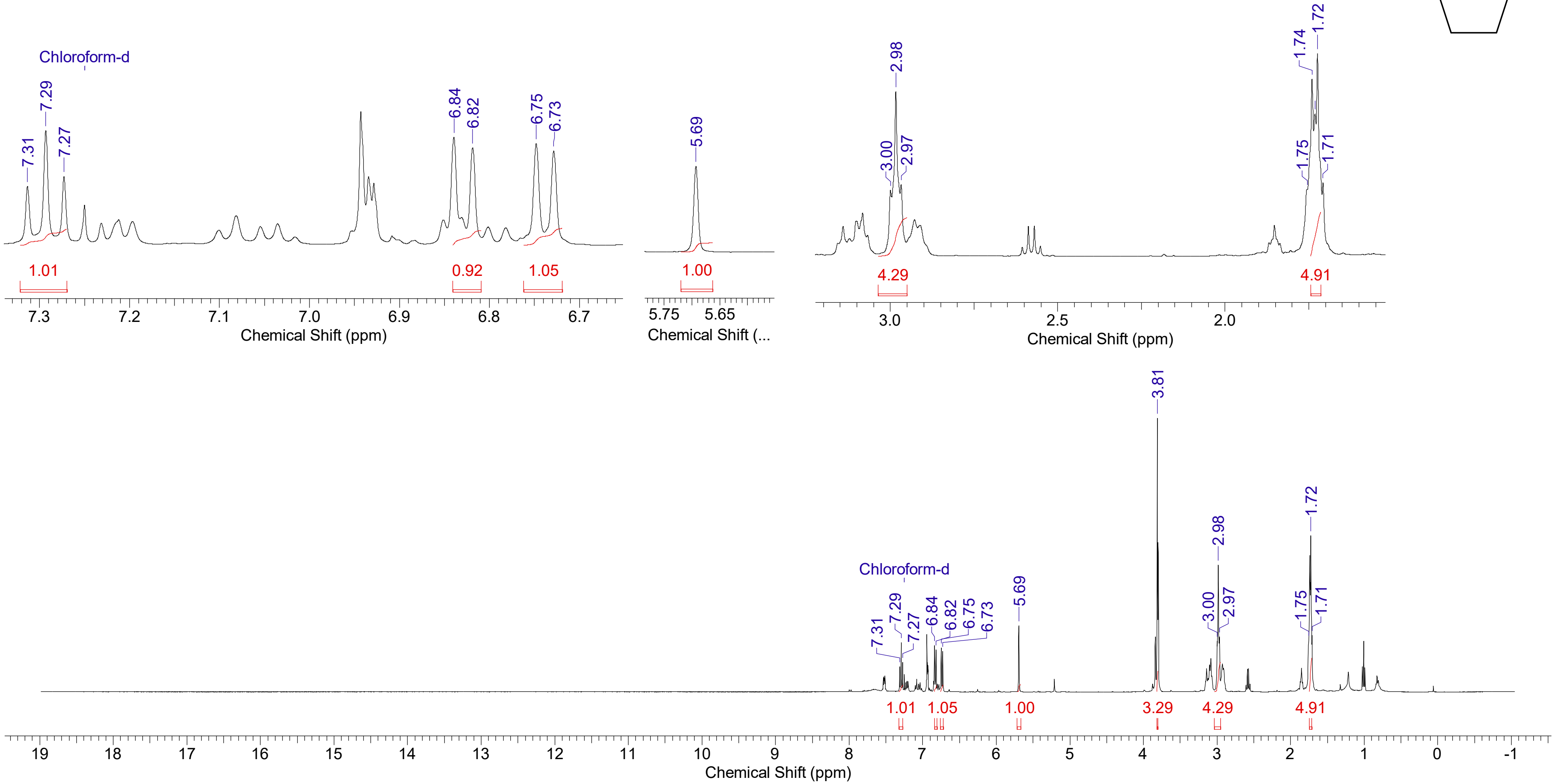

^{1}H NMR spectrum of **3c** (400.1 MHz, $CDCl_3$)

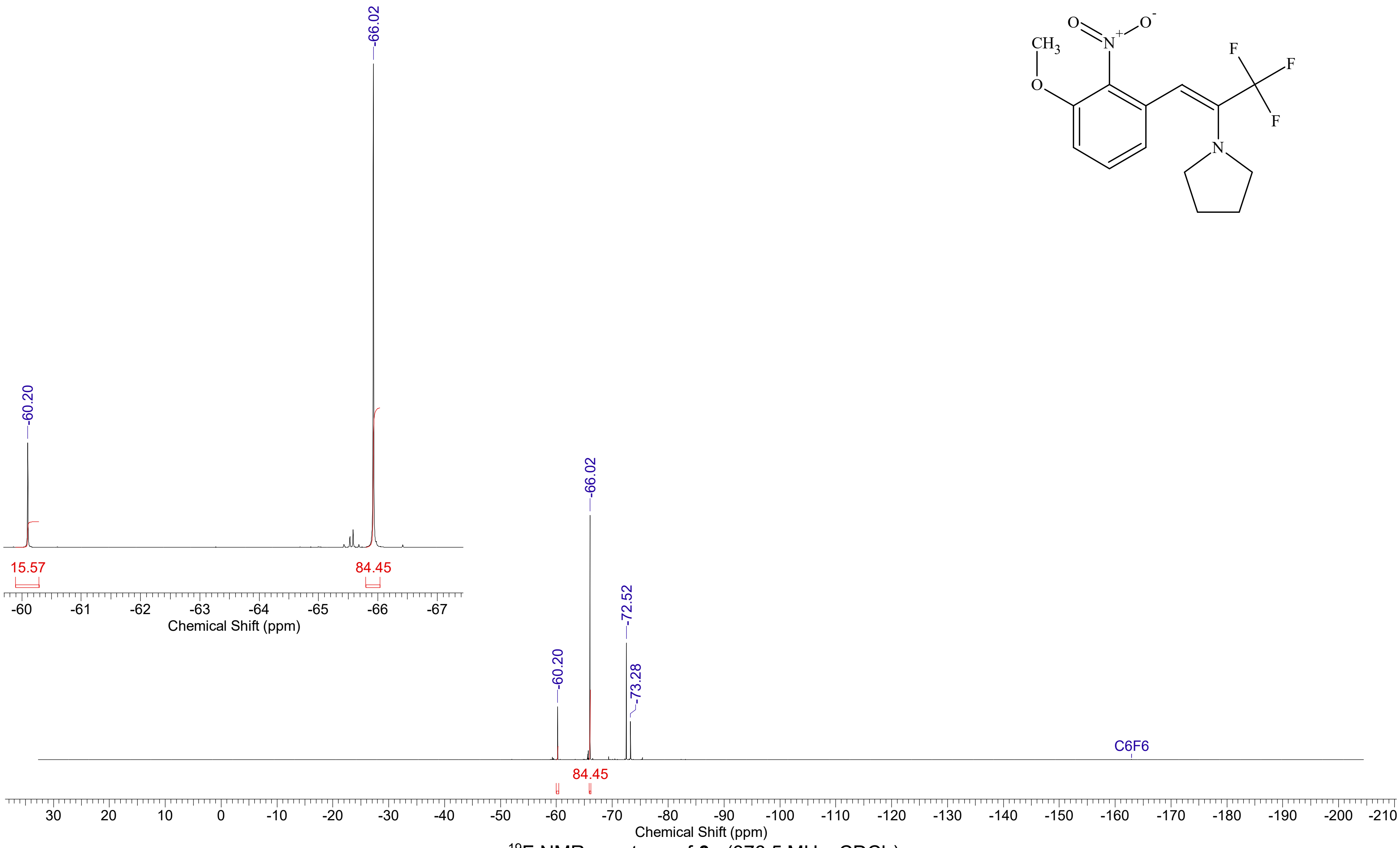

^{19}F NMR spectrum of **3c** (376.5 MHz, $CDCl_3$)

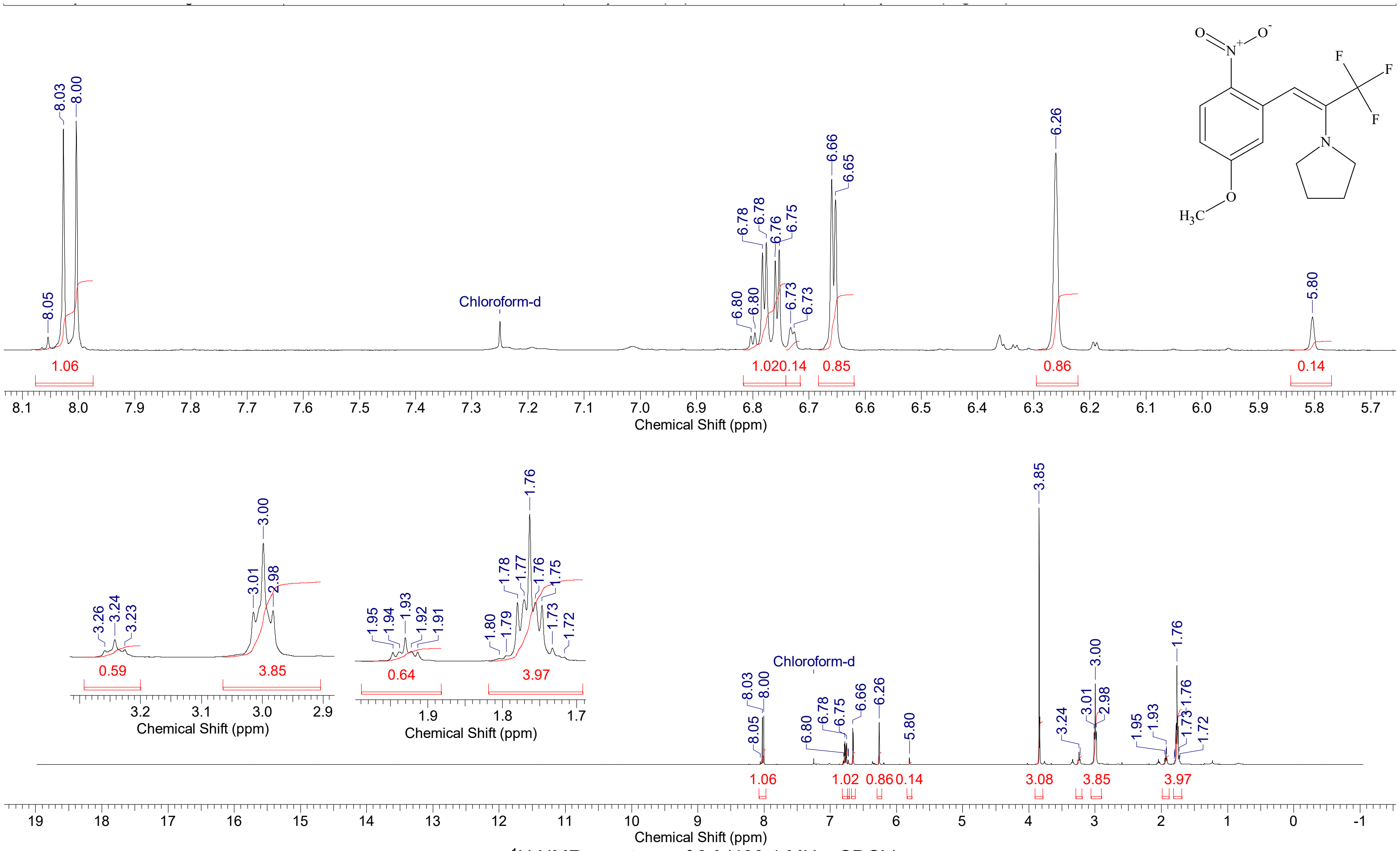

^{1}H NMR spectrum of **3d** (400.1 MHz, $CDCl_3$)

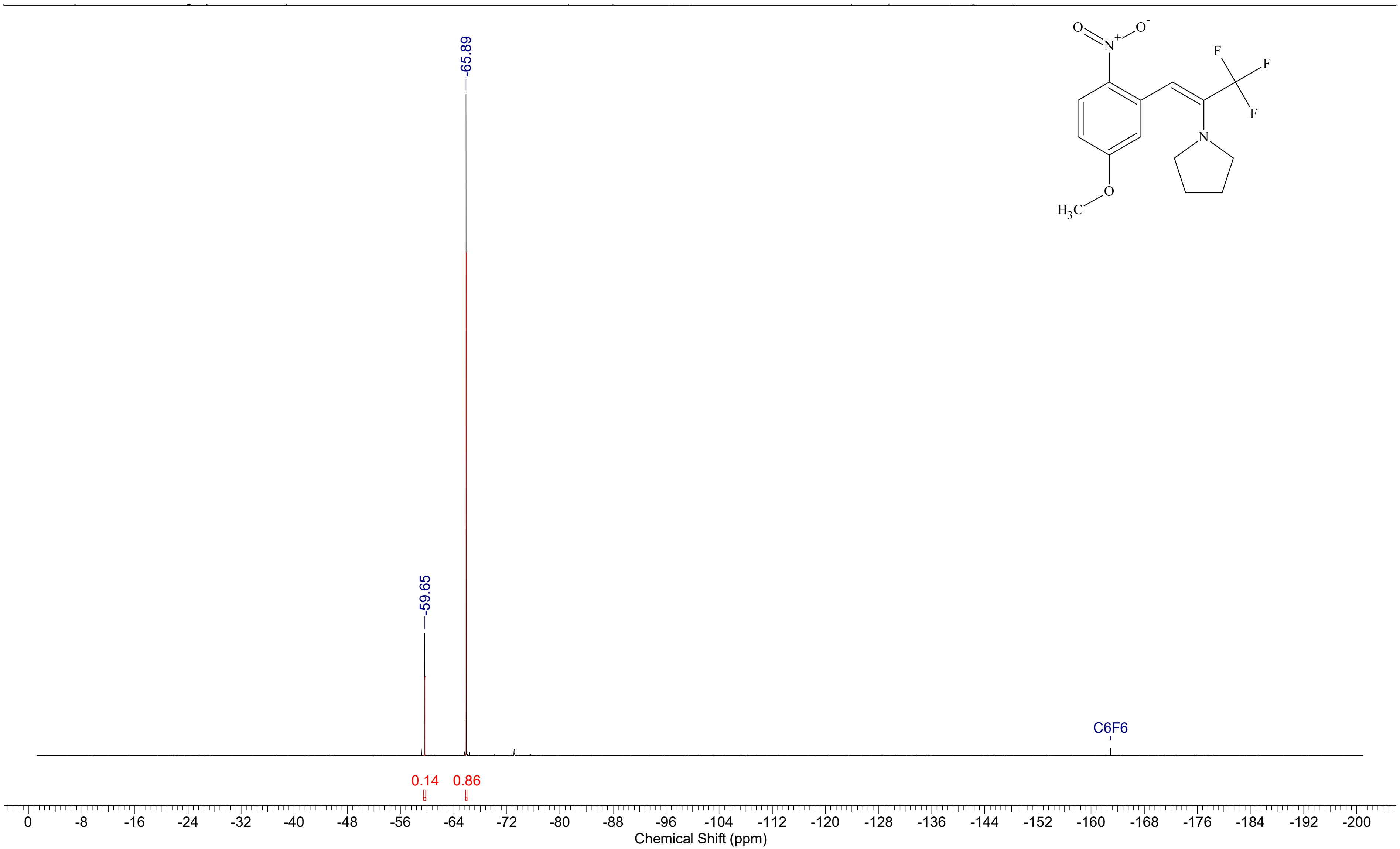

^{19}F NMR spectrum of **3d** (376.5 MHz, $CDCl_3$)

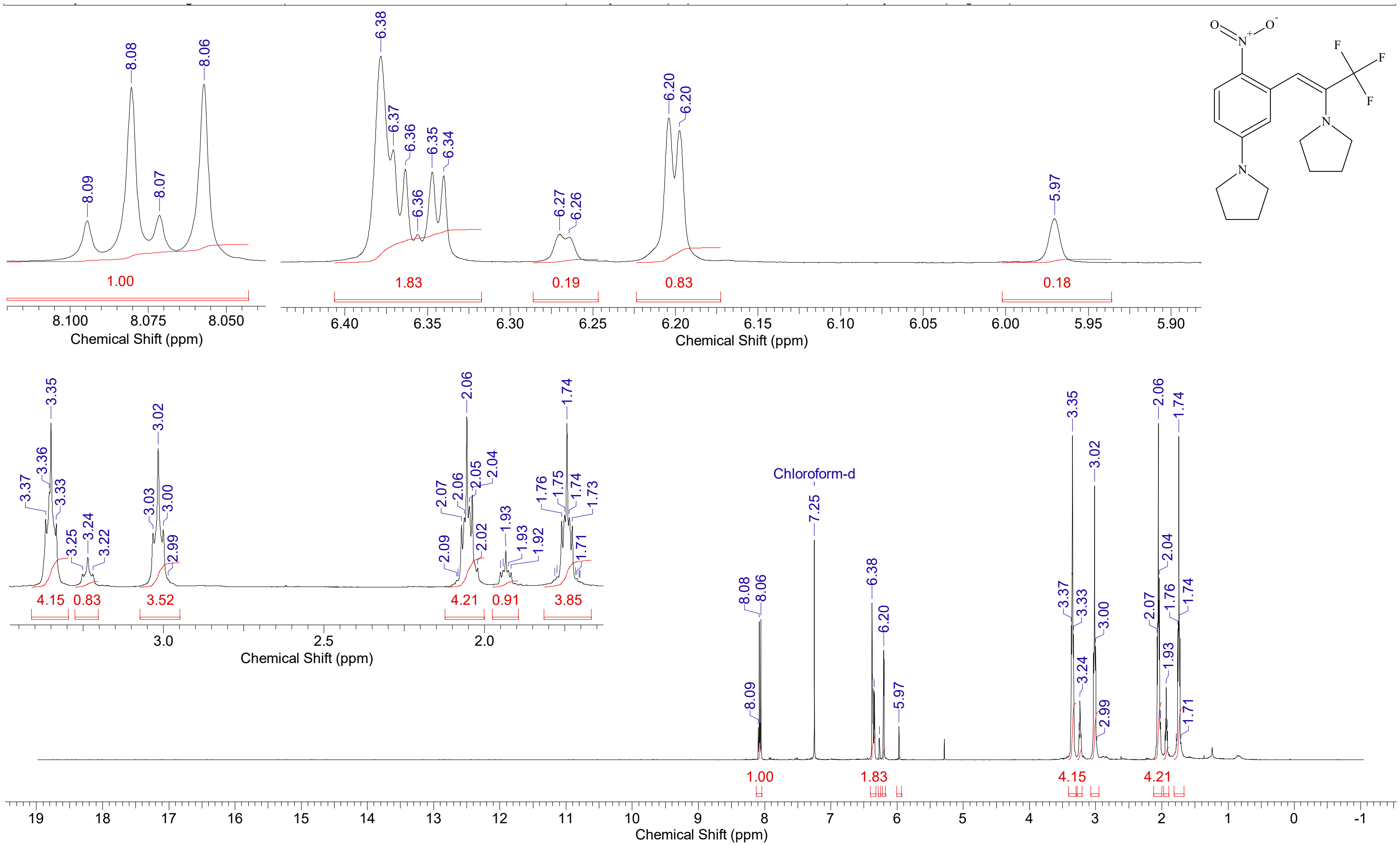

^{1}H NMR spectrum of **3n** (400.1 MHz, $CDCl_3$)

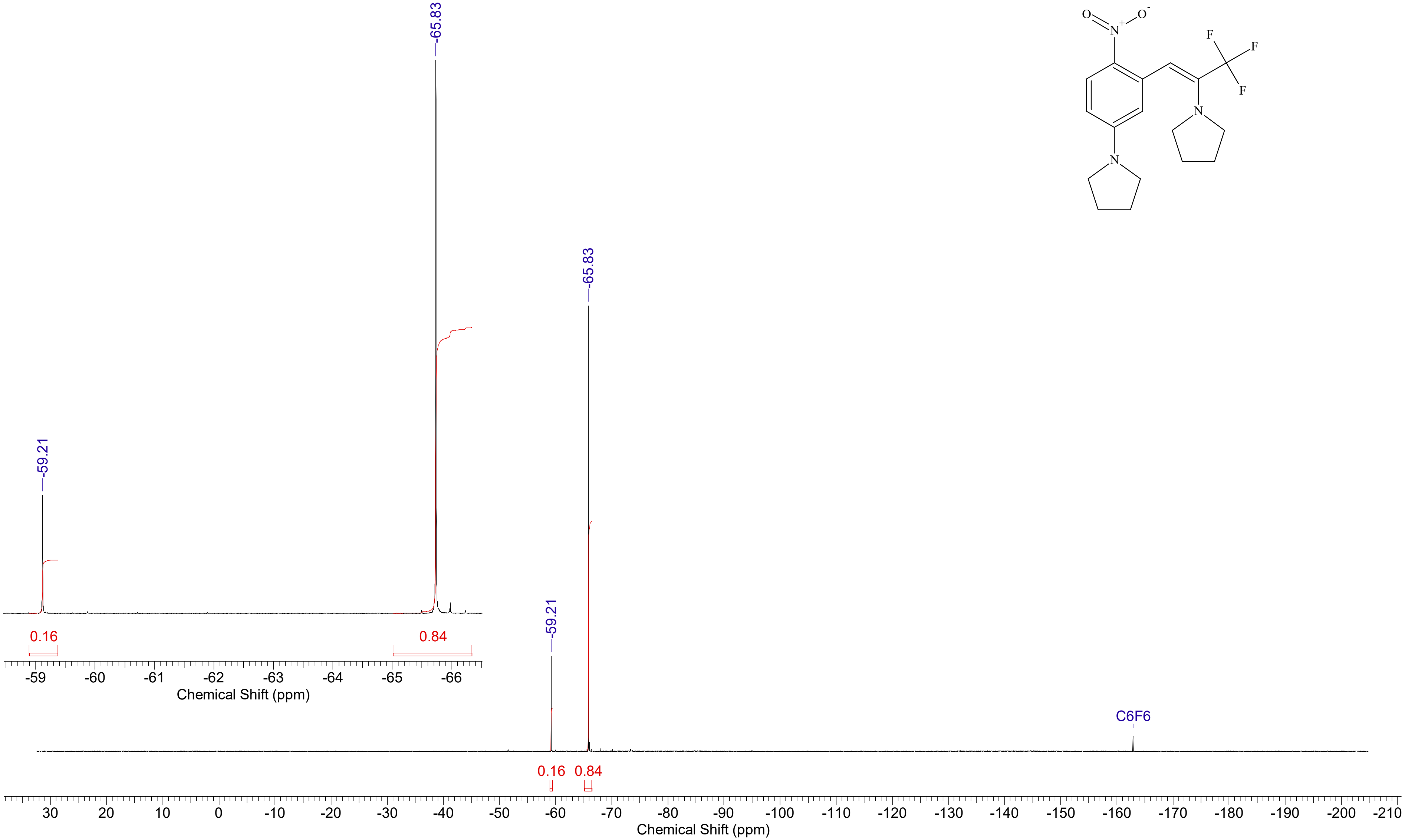

^{19}F NMR spectrum of **3n** (376.5 MHz, $CDCl_3$)

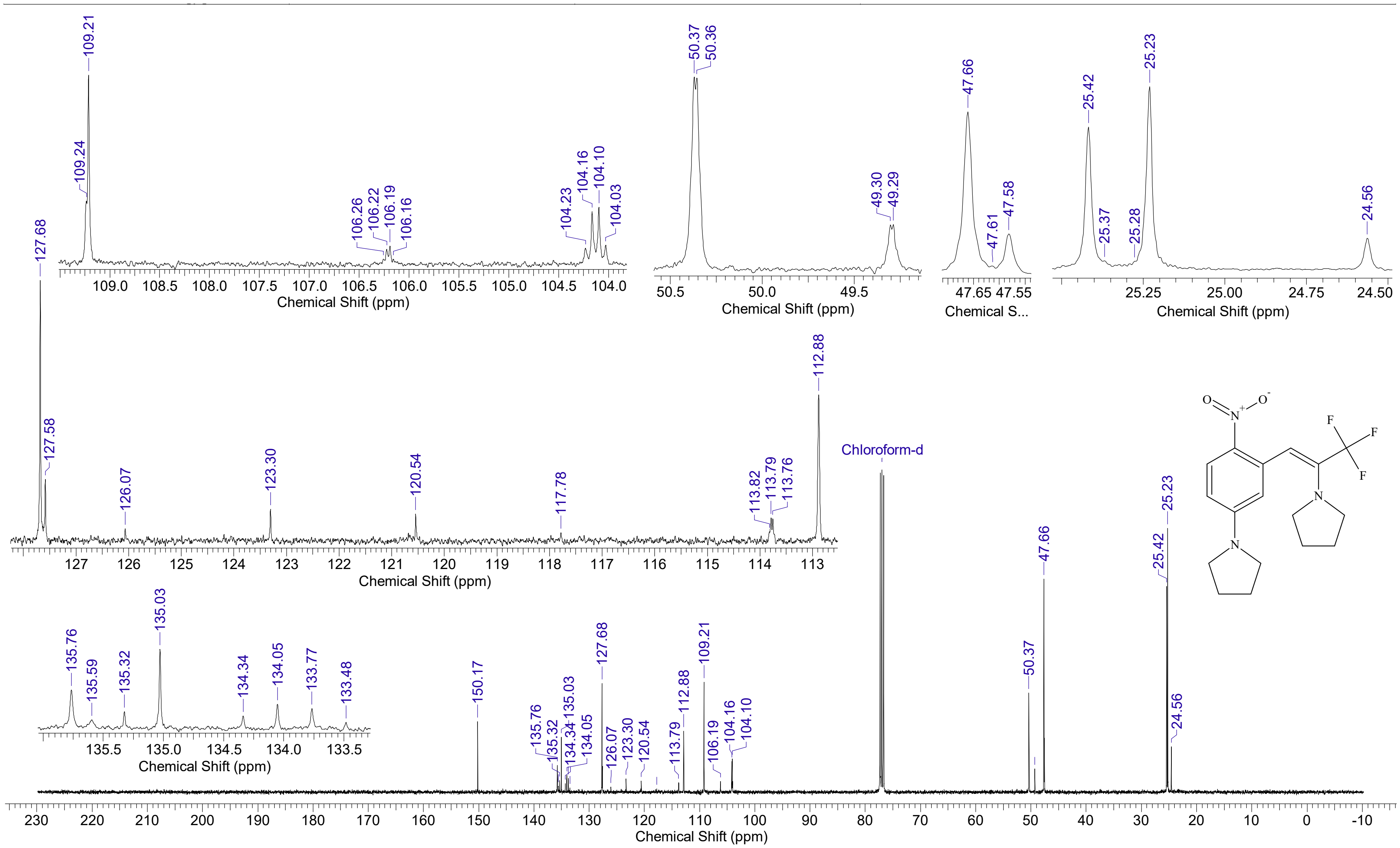

$^{13}C\{^1H\}$ NMR spectrum of **3n** (100.6 MHz, $CDCl_3$)

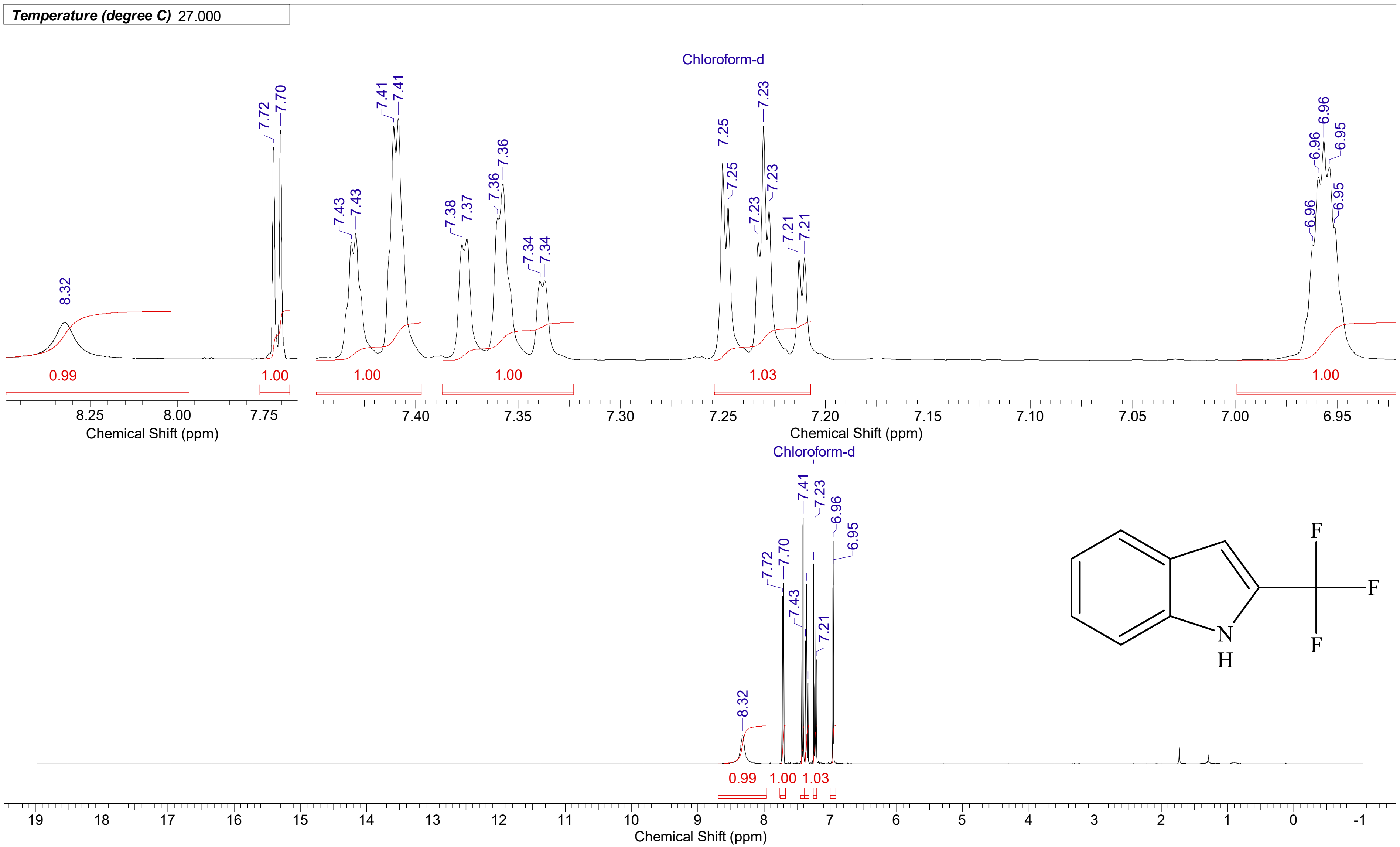

^{1}H NMR spectrum of **4a** (400.1 MHz, $CDCl_3$)

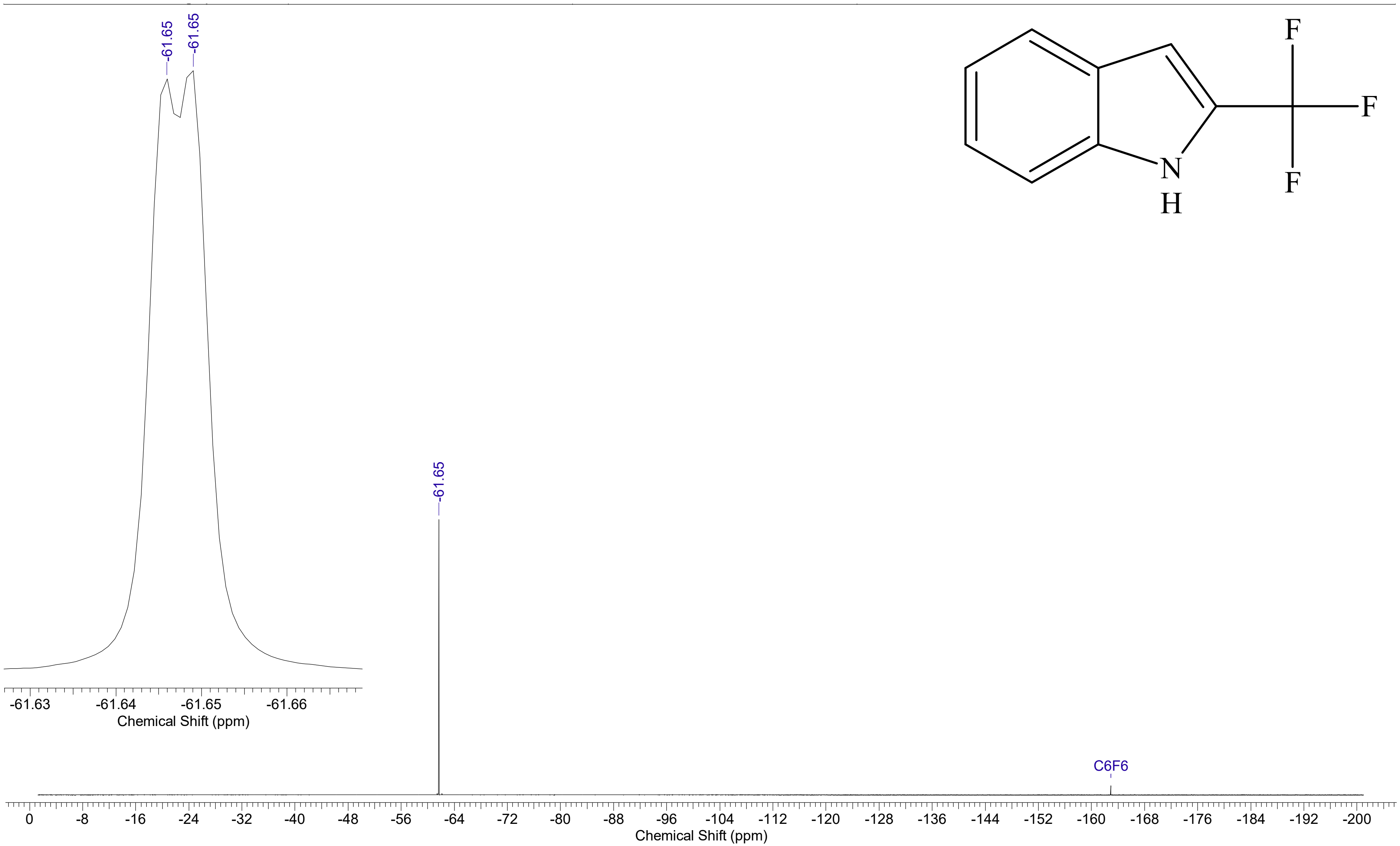

^{19}F NMR spectrum of **4a** (376.5 MHz, $CDCl_3$)

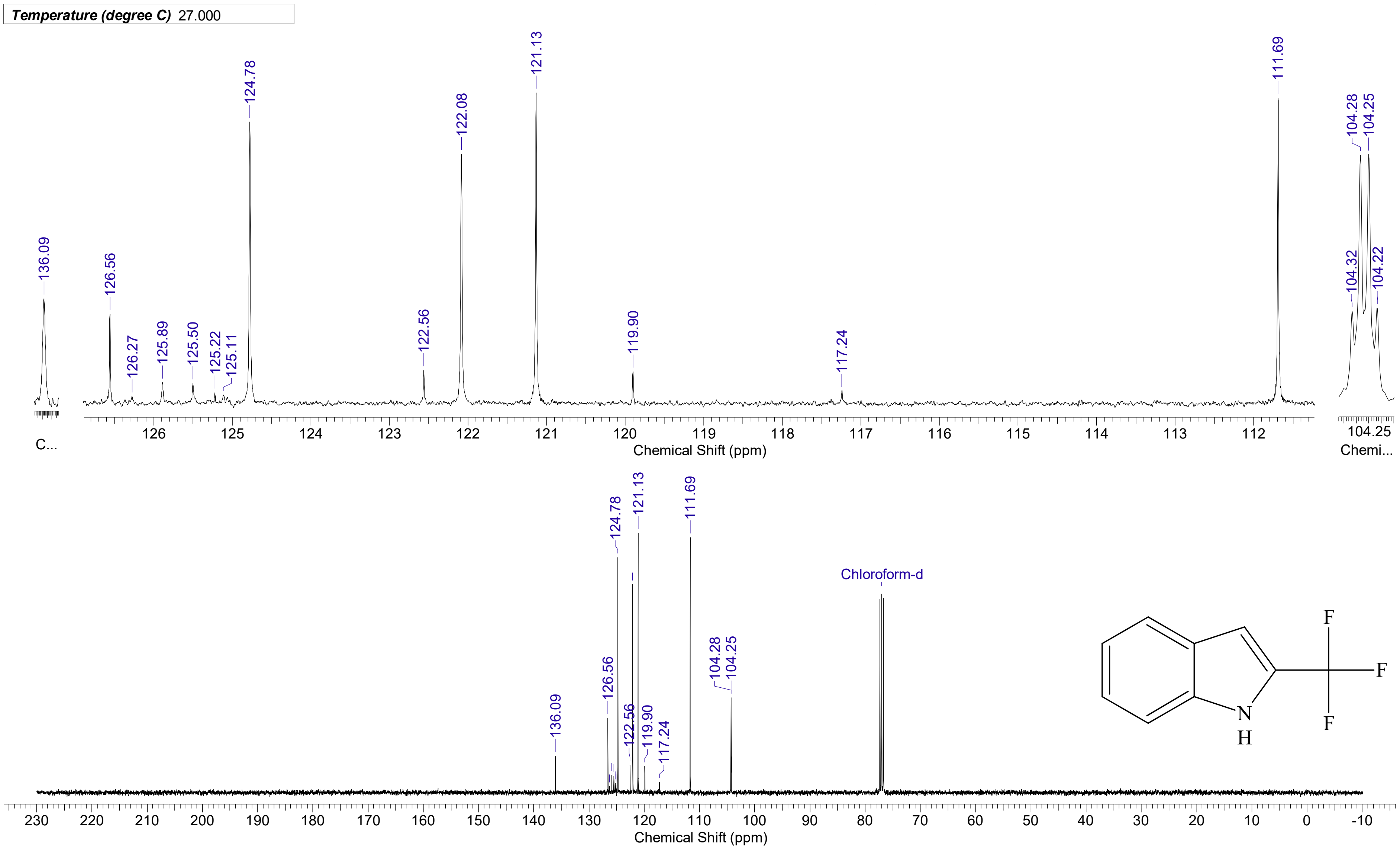

$^{13}C\{^{1}H\}$ NMR spectrum of **4a** (100.6 MHz, $CDCl_3$)

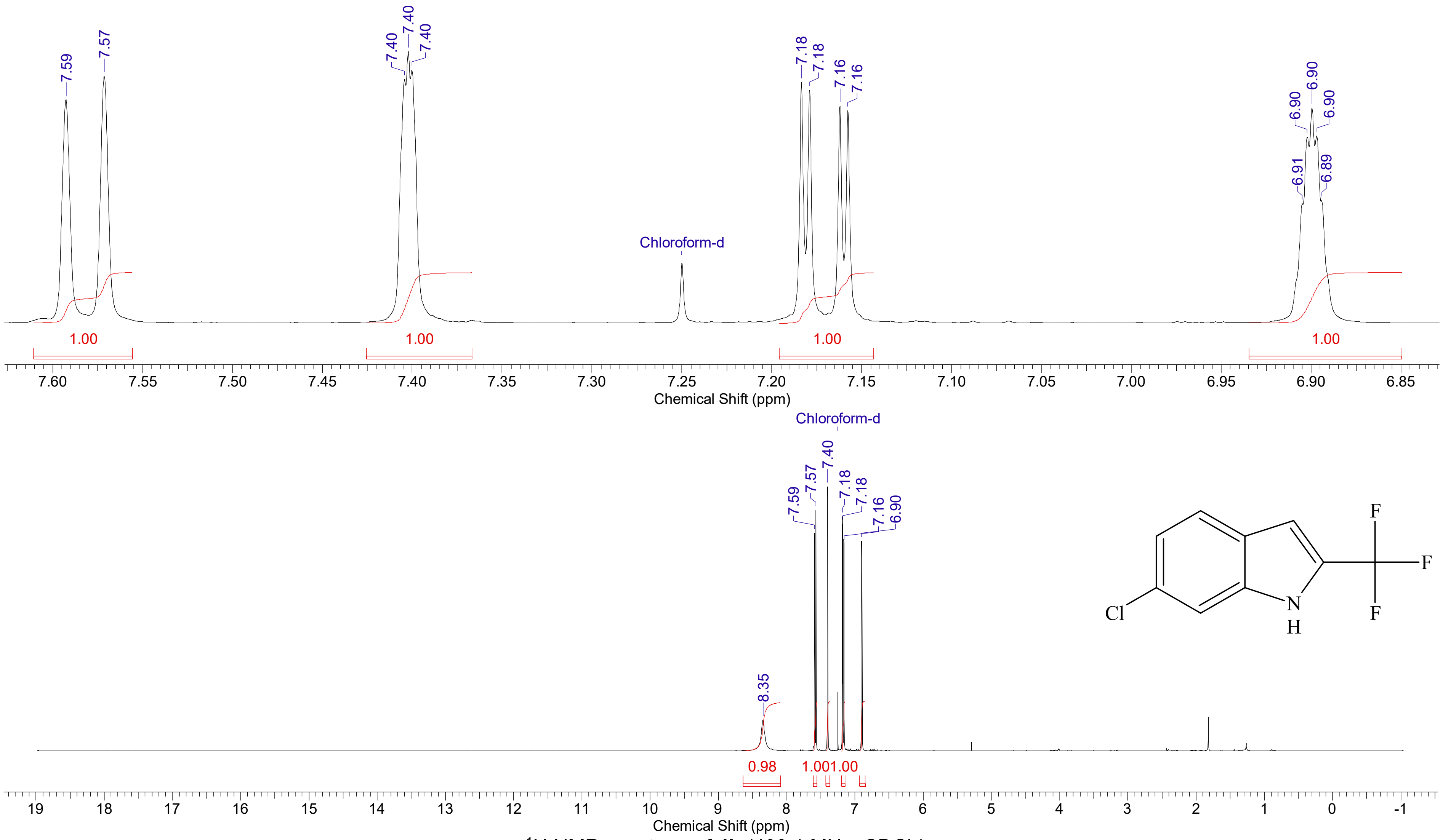

^{1}H NMR spectrum of **4b** (400.1 MHz, $CDCl_3$)

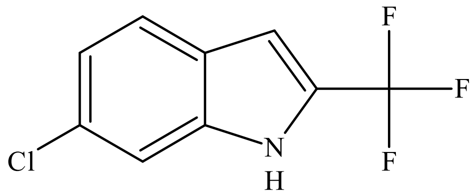

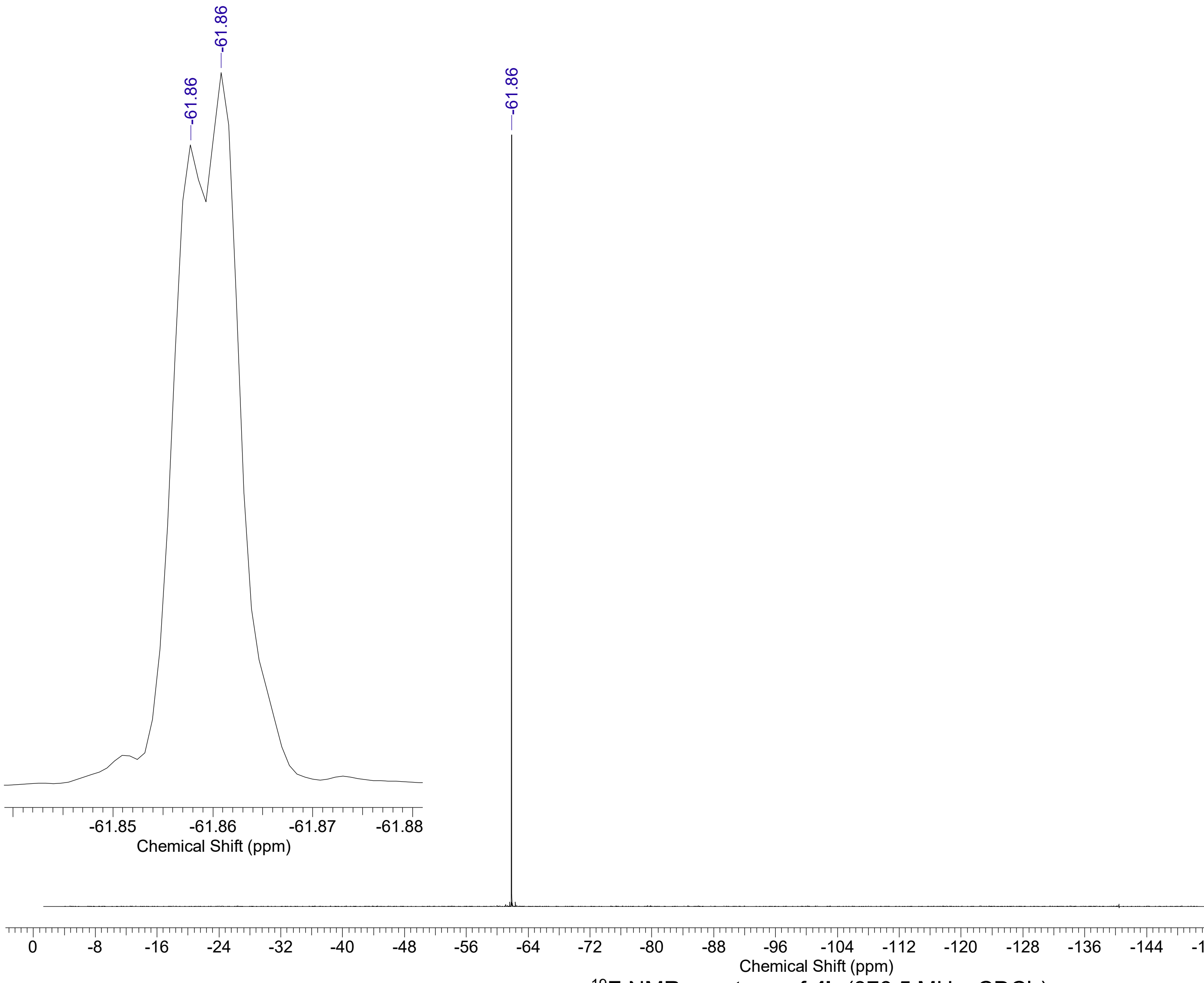

^{19}F NMR spectrum of **4b** (376.5 MHz, $CDCl_3$)

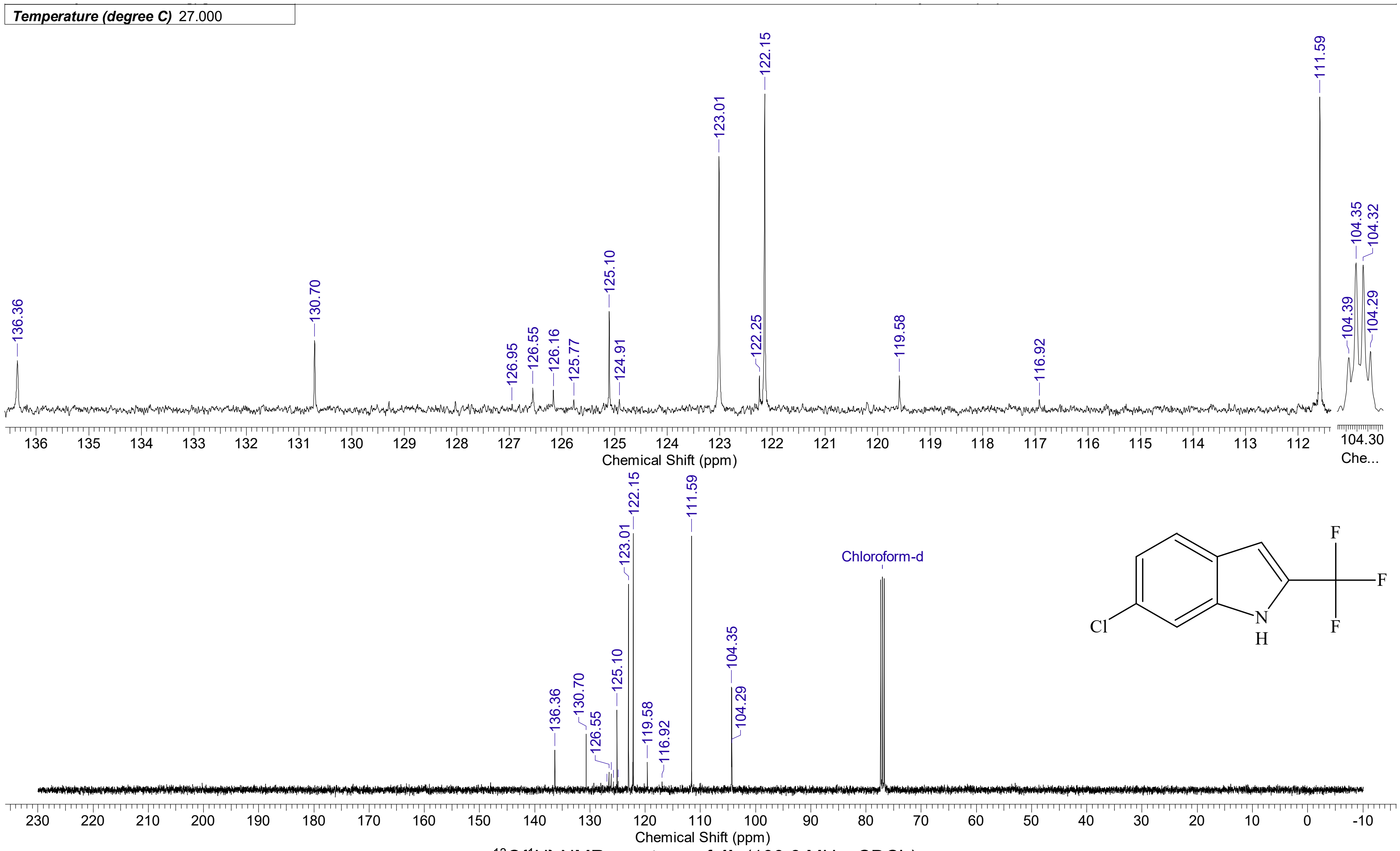

$^{13}C\{^{1}H\}$ NMR spectrum of **4b** (100.6 MHz, $CDCl_3$)

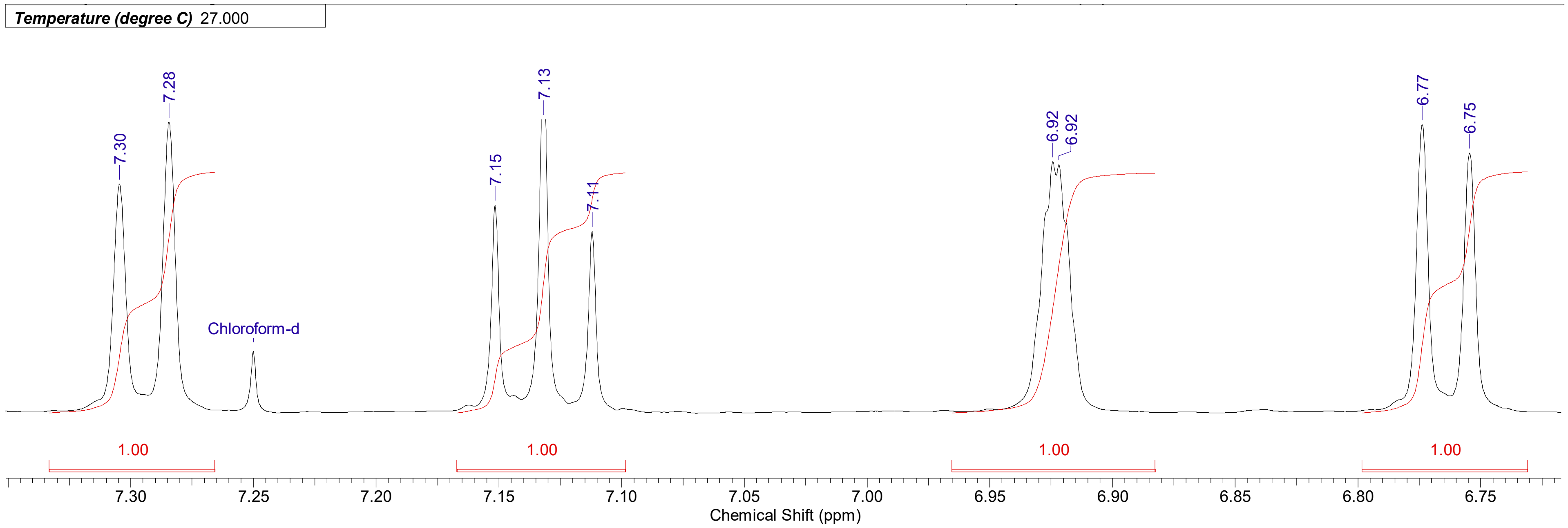

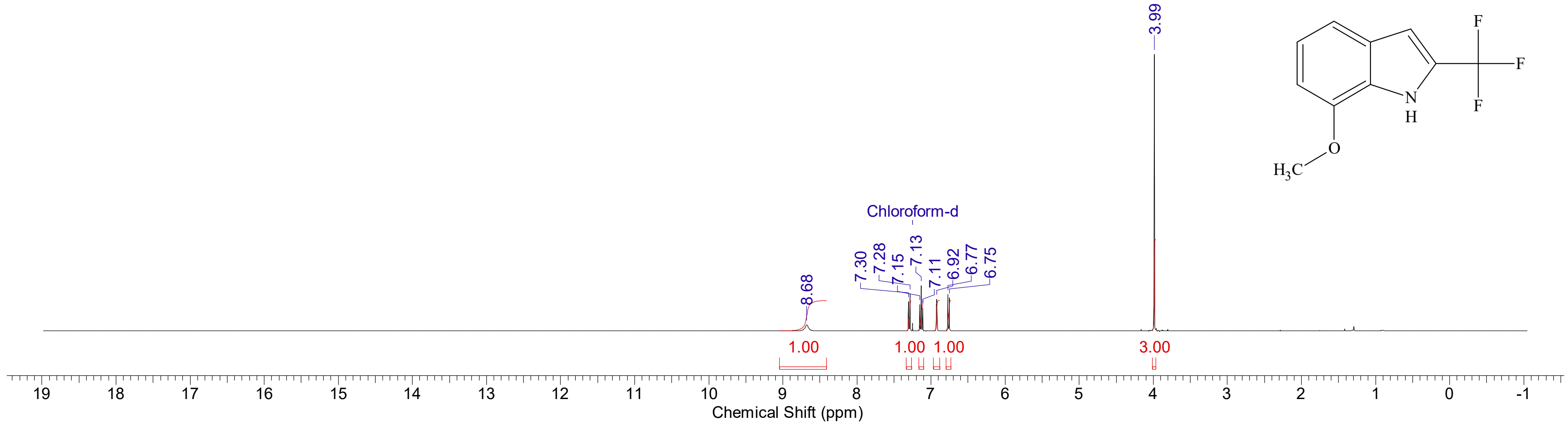

^{1}H NMR spectrum of **4c** (400.1 MHz, $CDCl_3$)

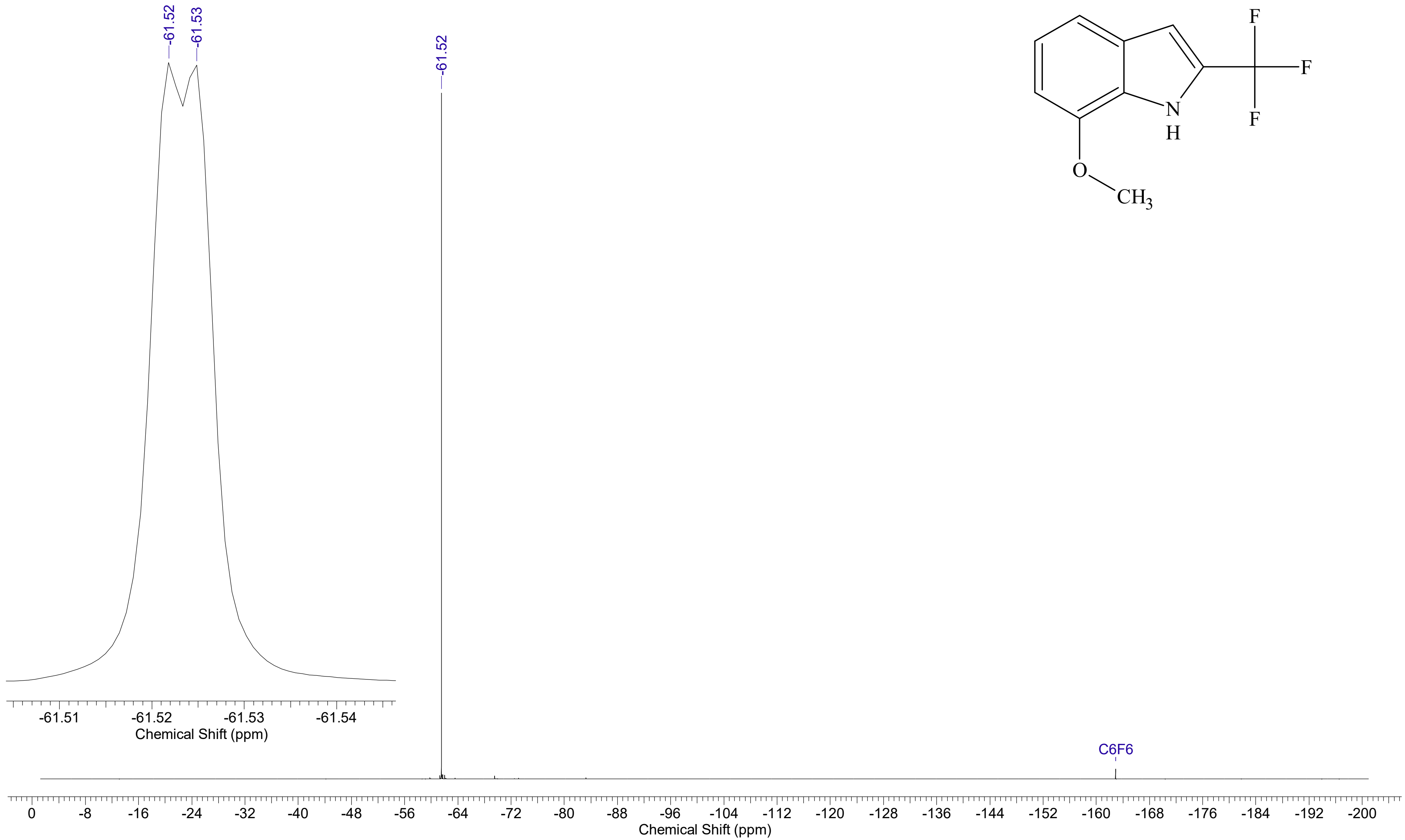

^{19}F NMR spectrum of **4c** (376.5 MHz, $CDCl_3$)

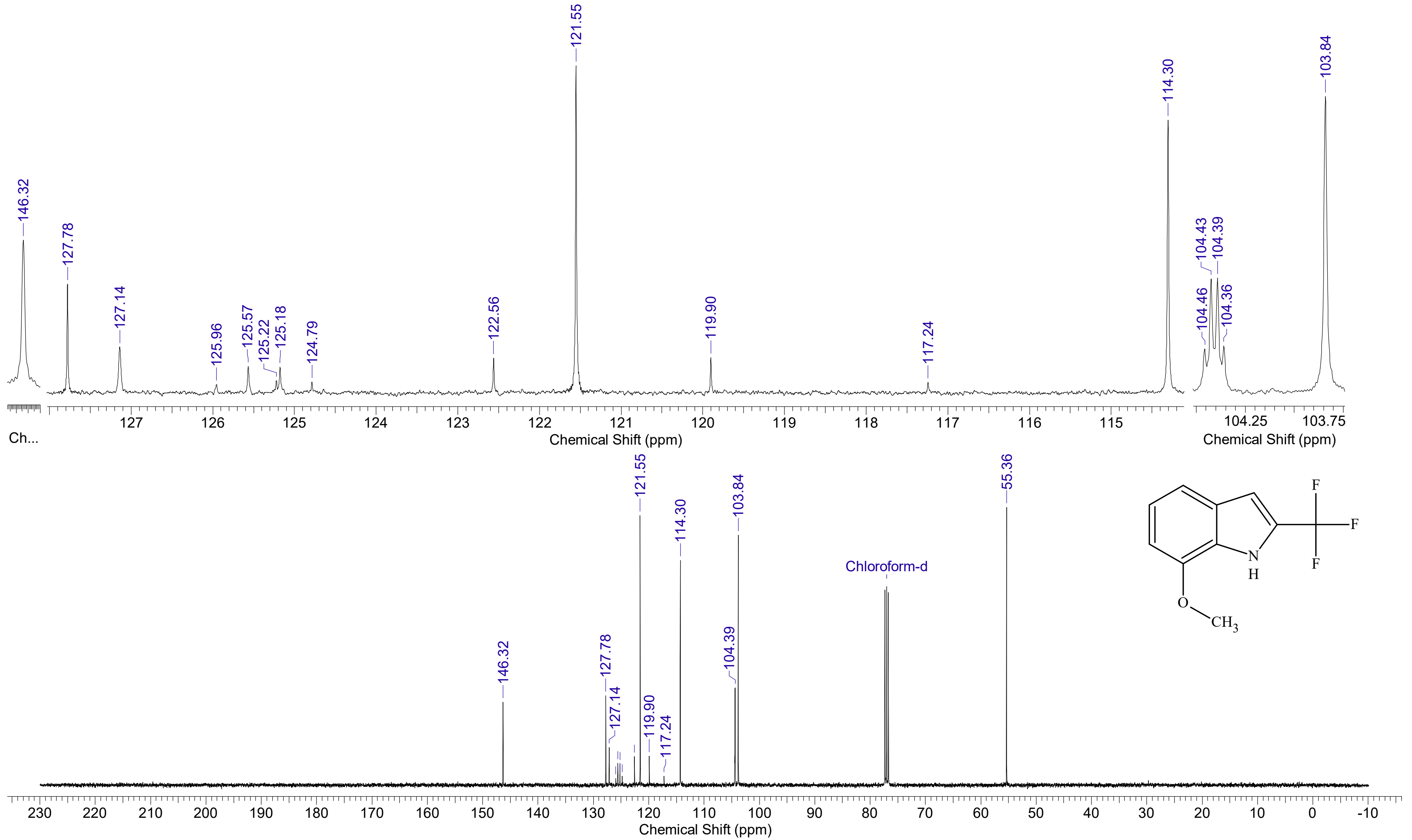

$^{13}C\{^{1}H\}$ NMR spectrum of **4c** (100.6 MHz, $CDCl_3$)

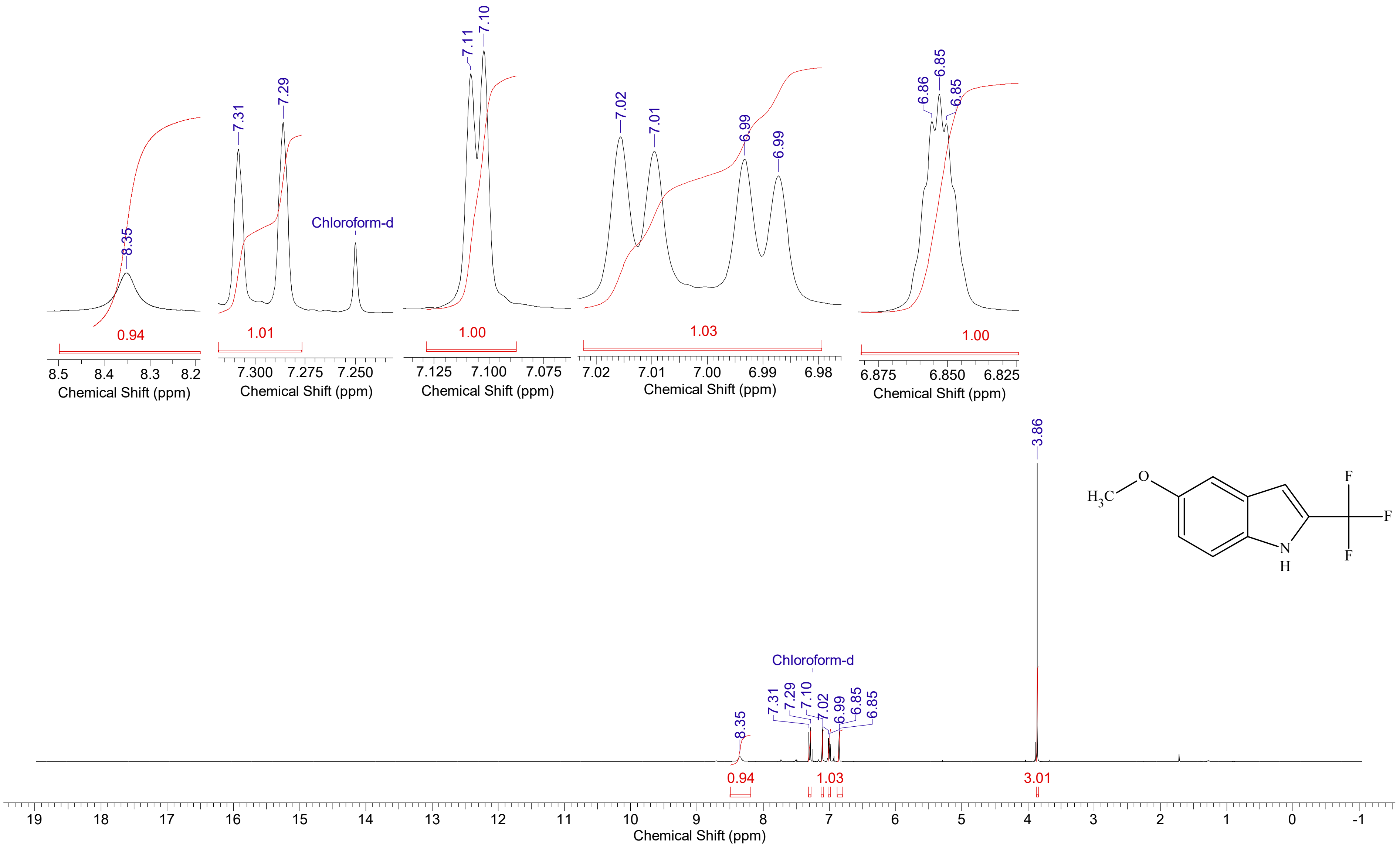

^{1}H NMR spectrum of **4d** (400.1 MHz, $CDCl_3$)

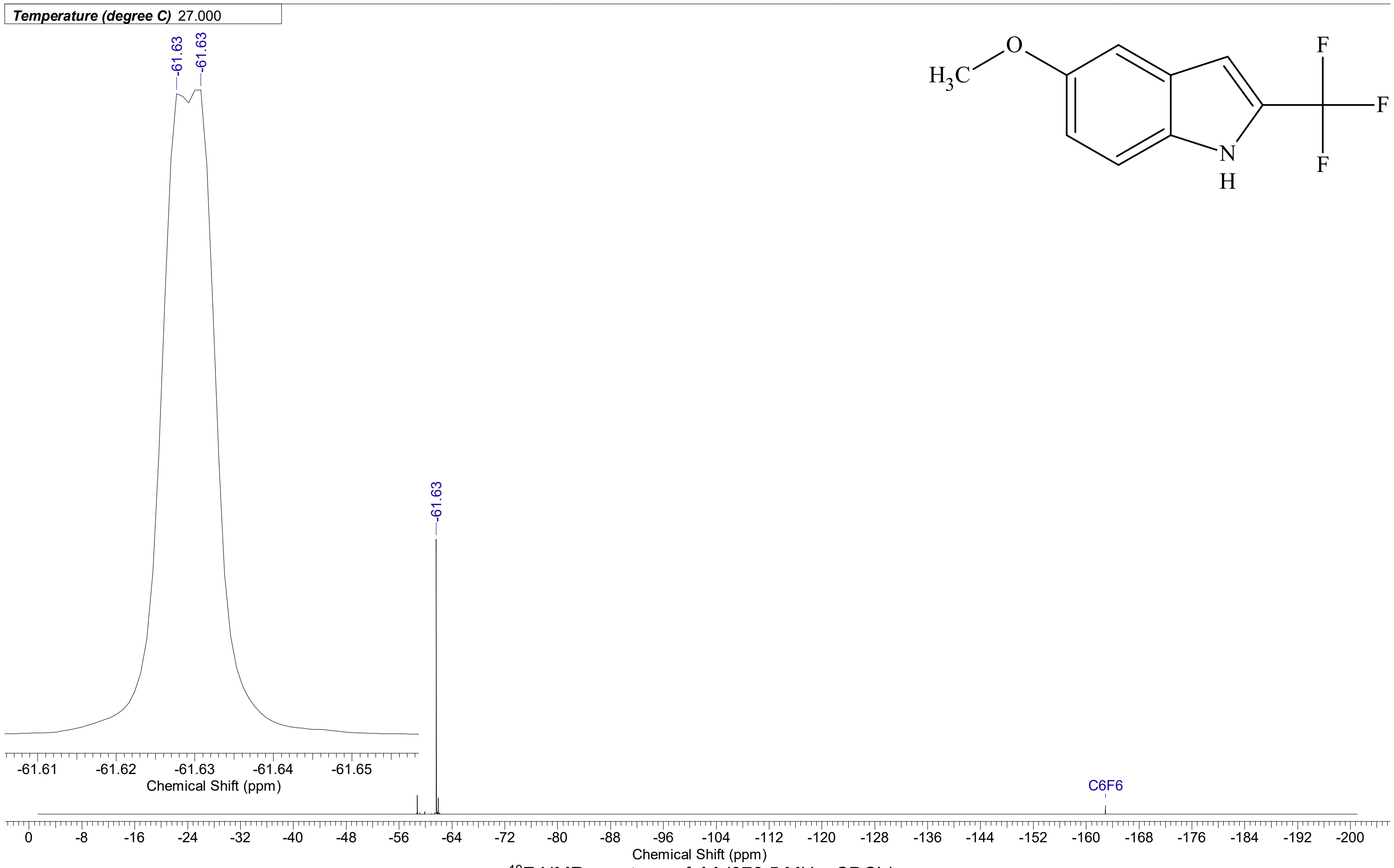

^{19}F NMR spectrum of **4d** (376.5 MHz, $CDCl_3$)

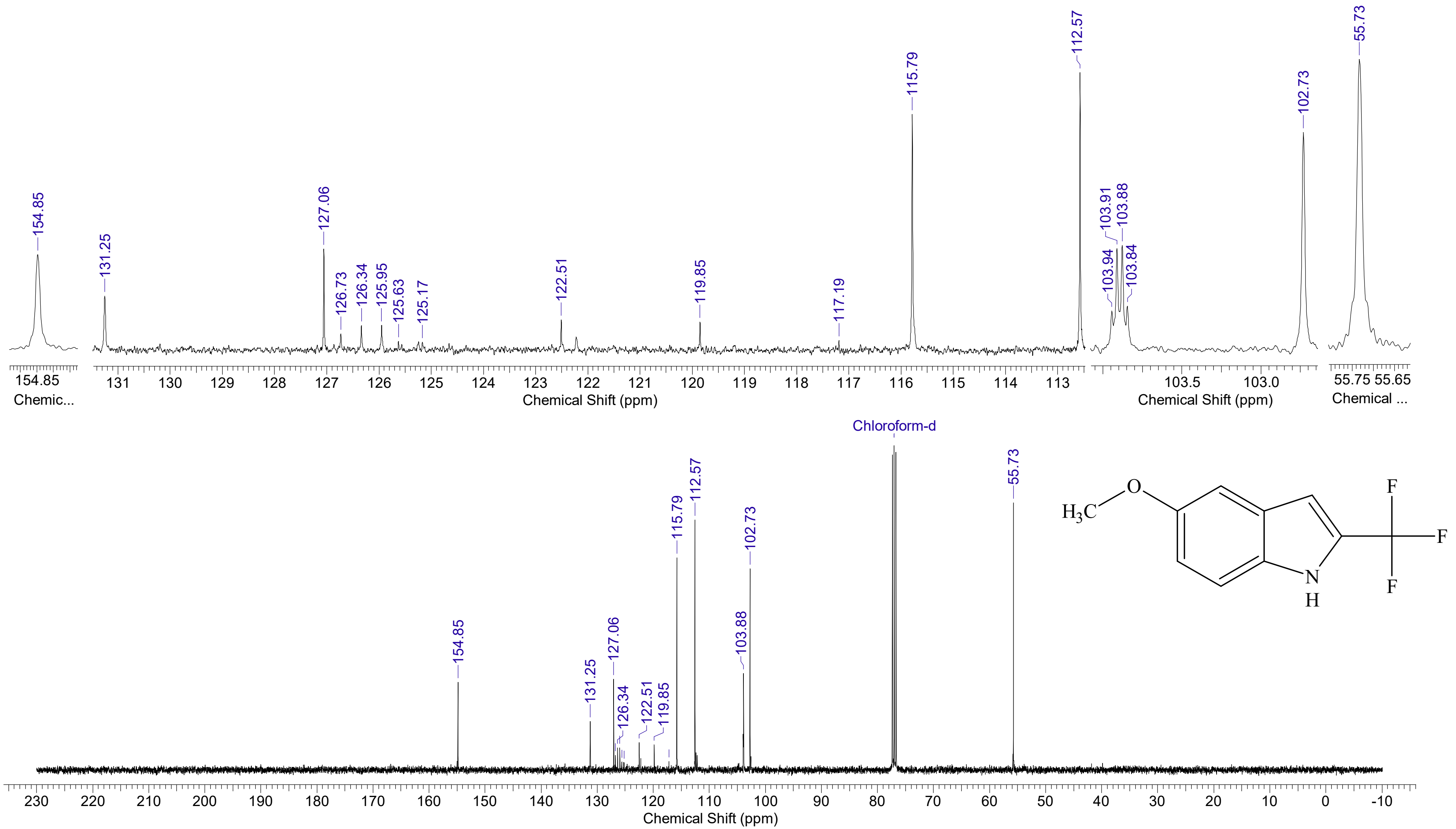

$^{13}C\{^1H\}$ NMR spectrum of **4d** (100.6 MHz, $CDCl_3$).

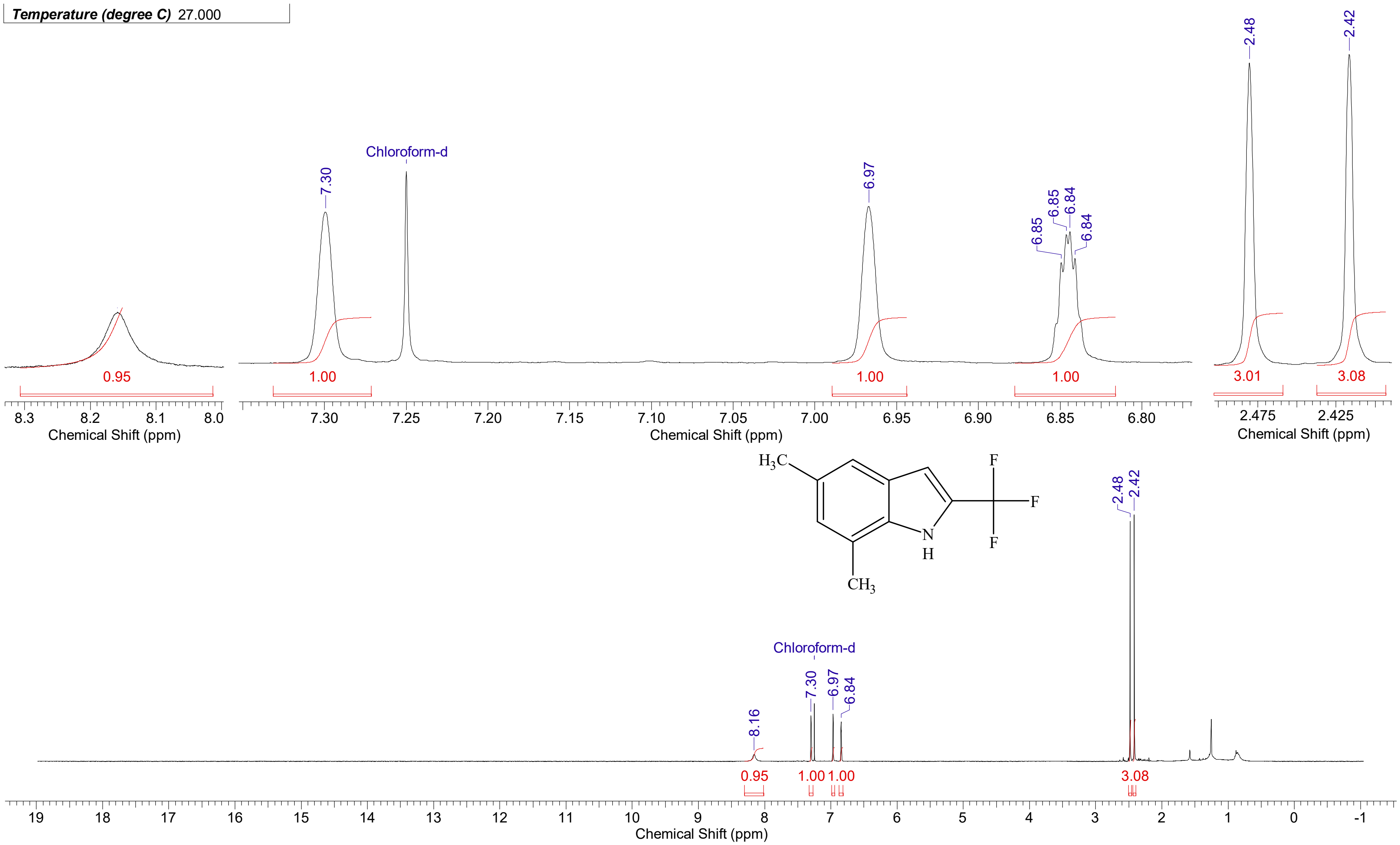

^{1}H NMR spectrum of **4e** (400.1 MHz, $CDCl_3$)

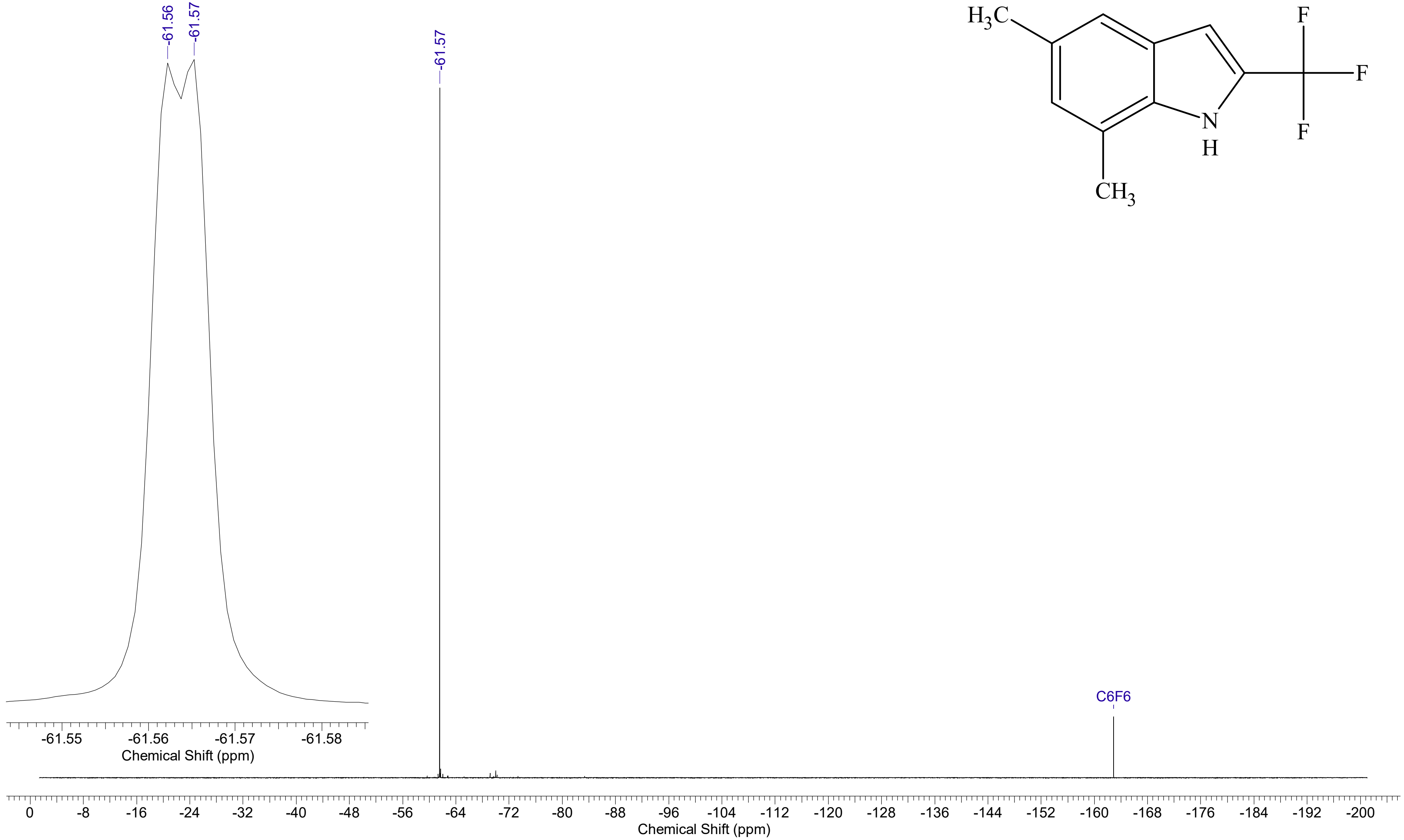

^{19}F NMR spectrum of **4e** (376.5 MHz, $CDCl_3$)

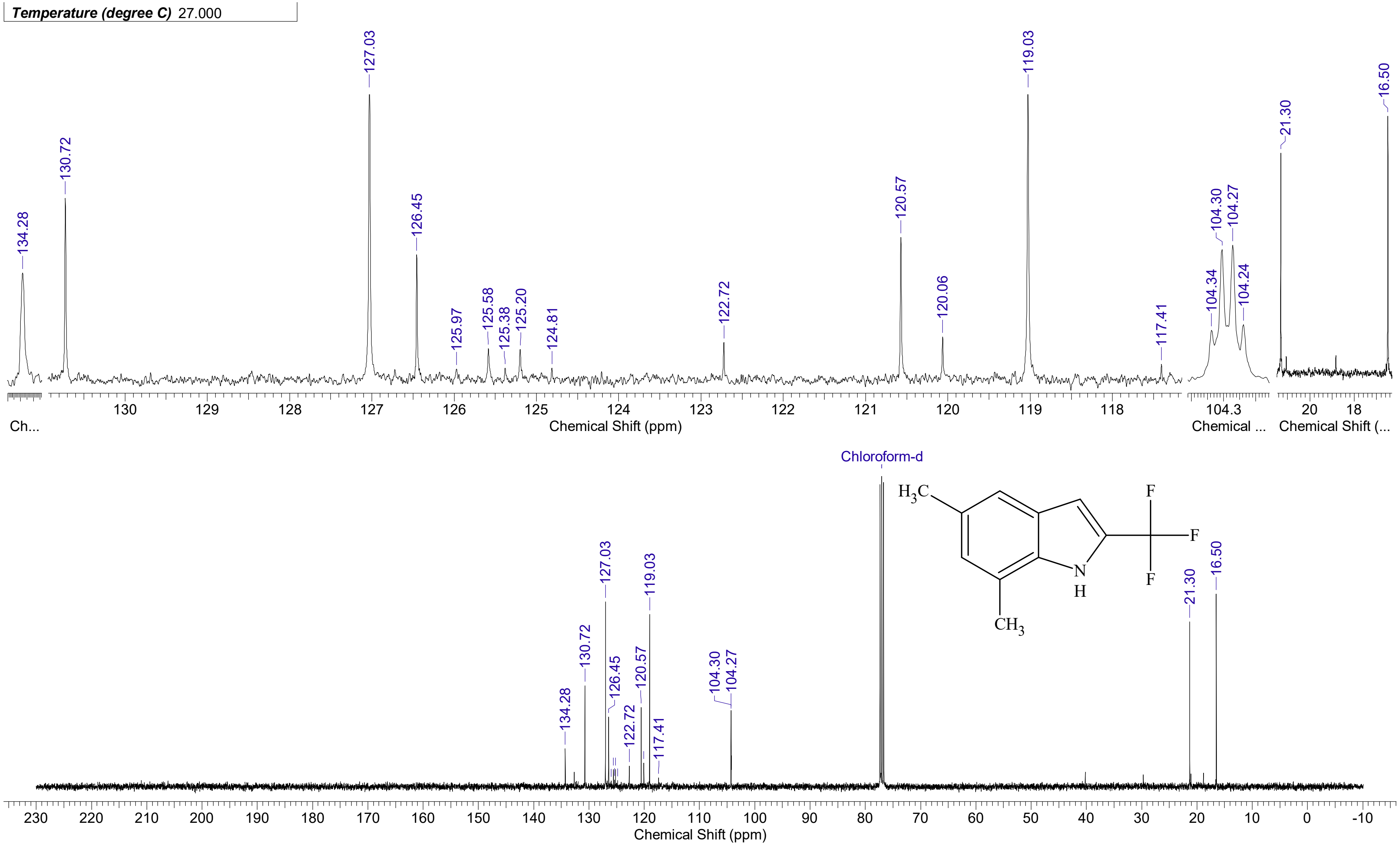

$^{13}C\{^{1}H\}$ NMR spectrum of **4e** (100.6 MHz, $CDCl_3$)

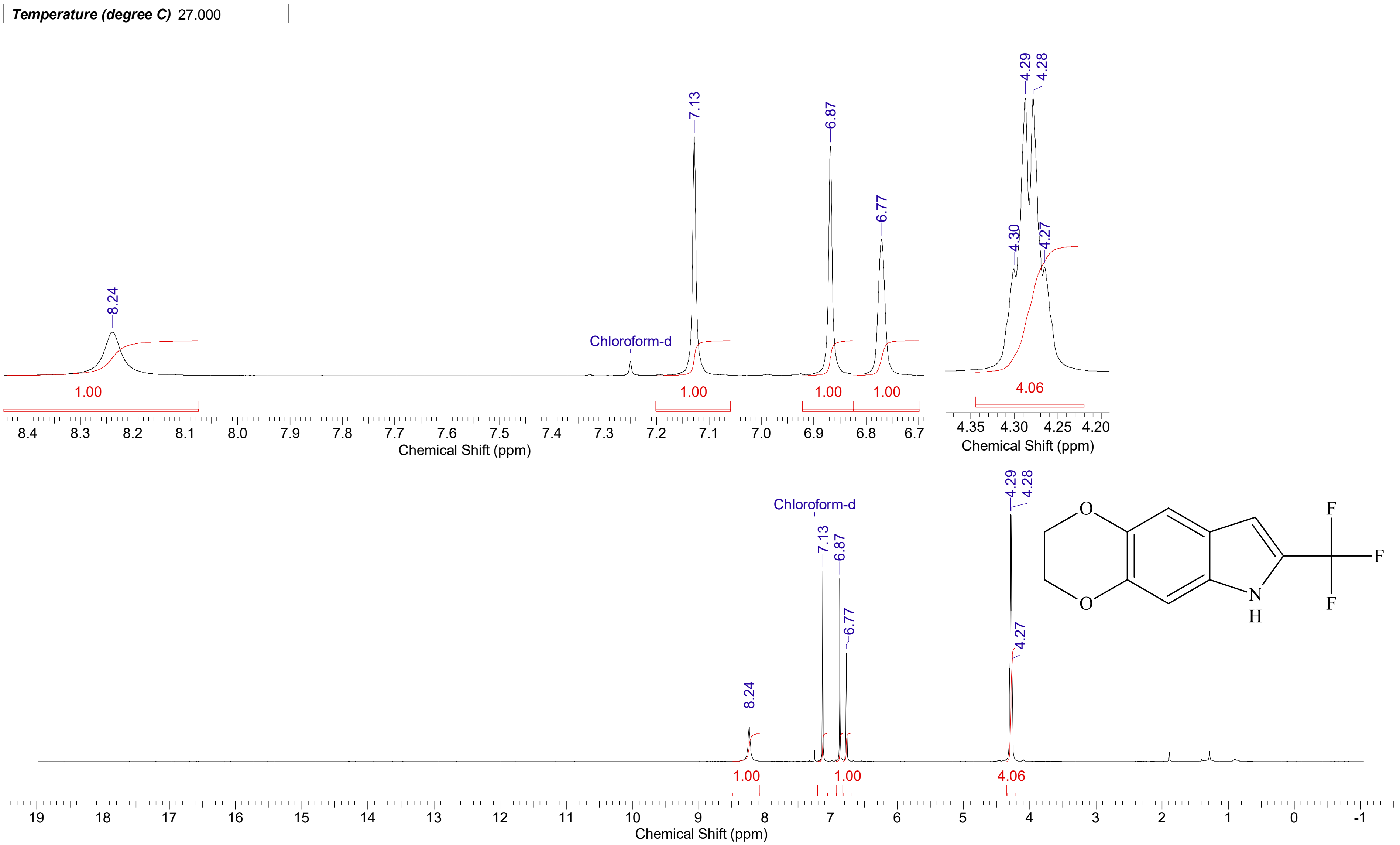

^{1}H NMR spectrum of **4f** (400.1 MHz, $CDCl_3$)

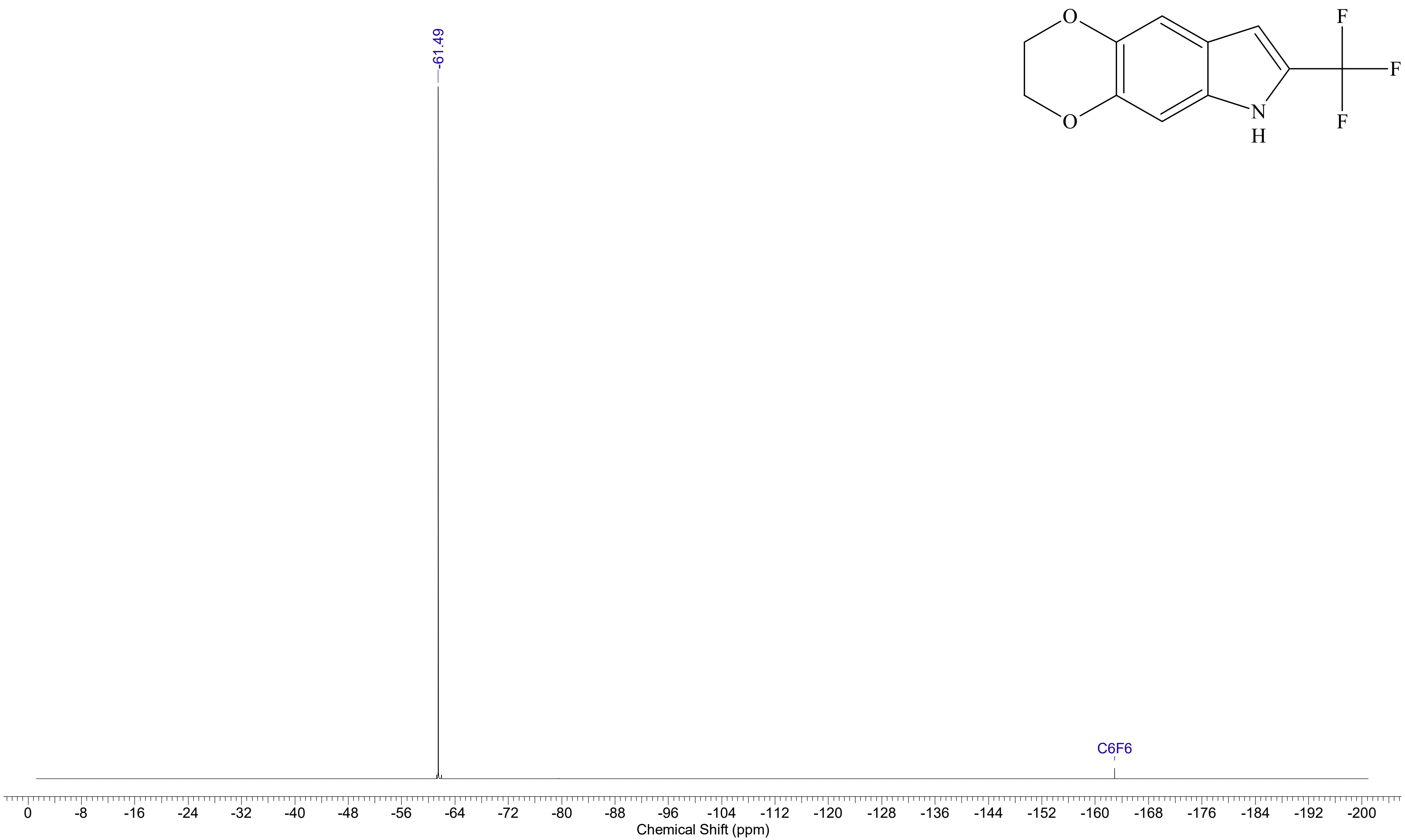

^{19}F NMR spectrum of **4f** (376.5 MHz, $CDCl_3$)

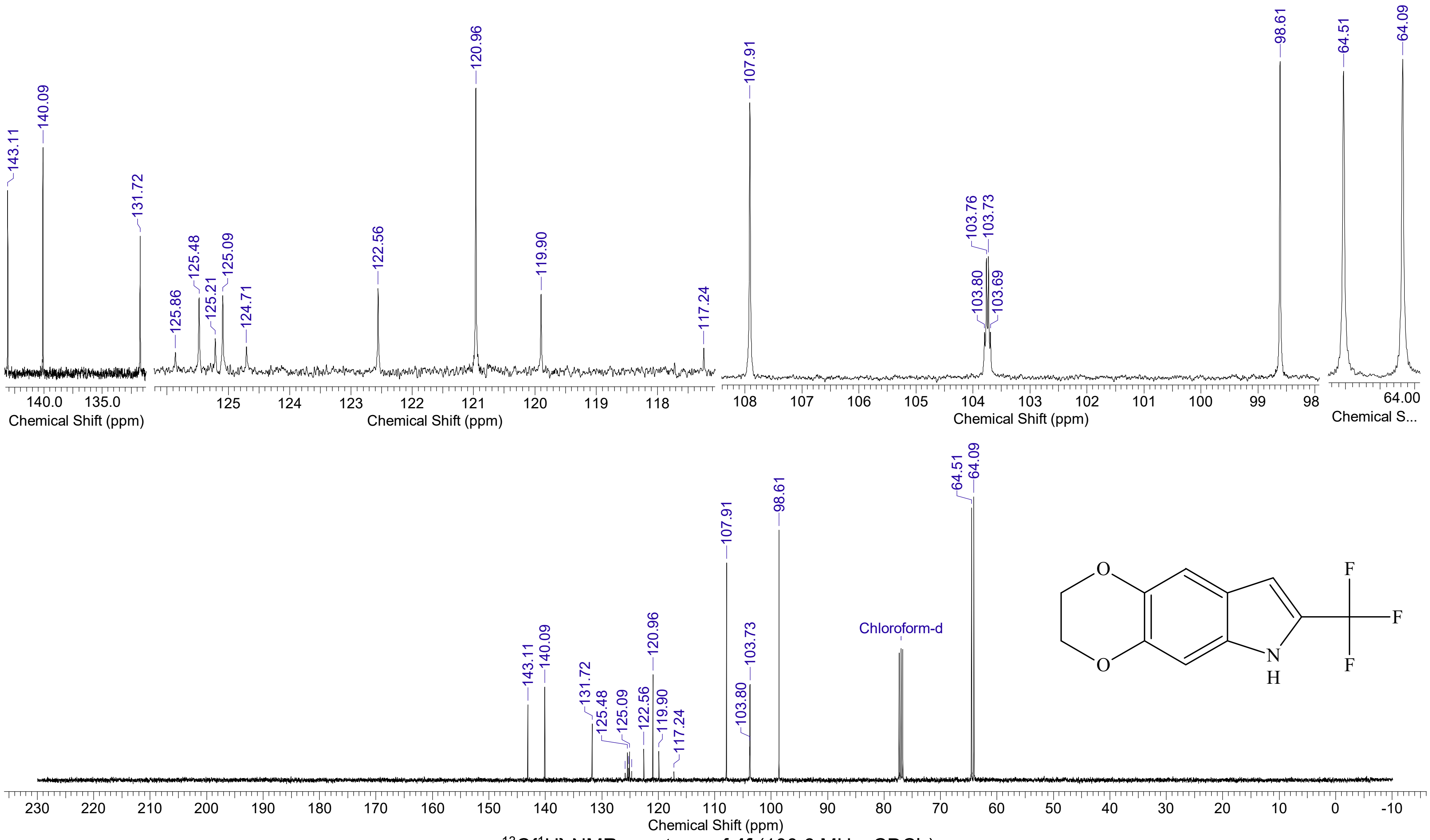

$^{13}C\{^{1}H\}$ NMR spectrum of **4f** (100.6 MHz, $CDCl_3$)

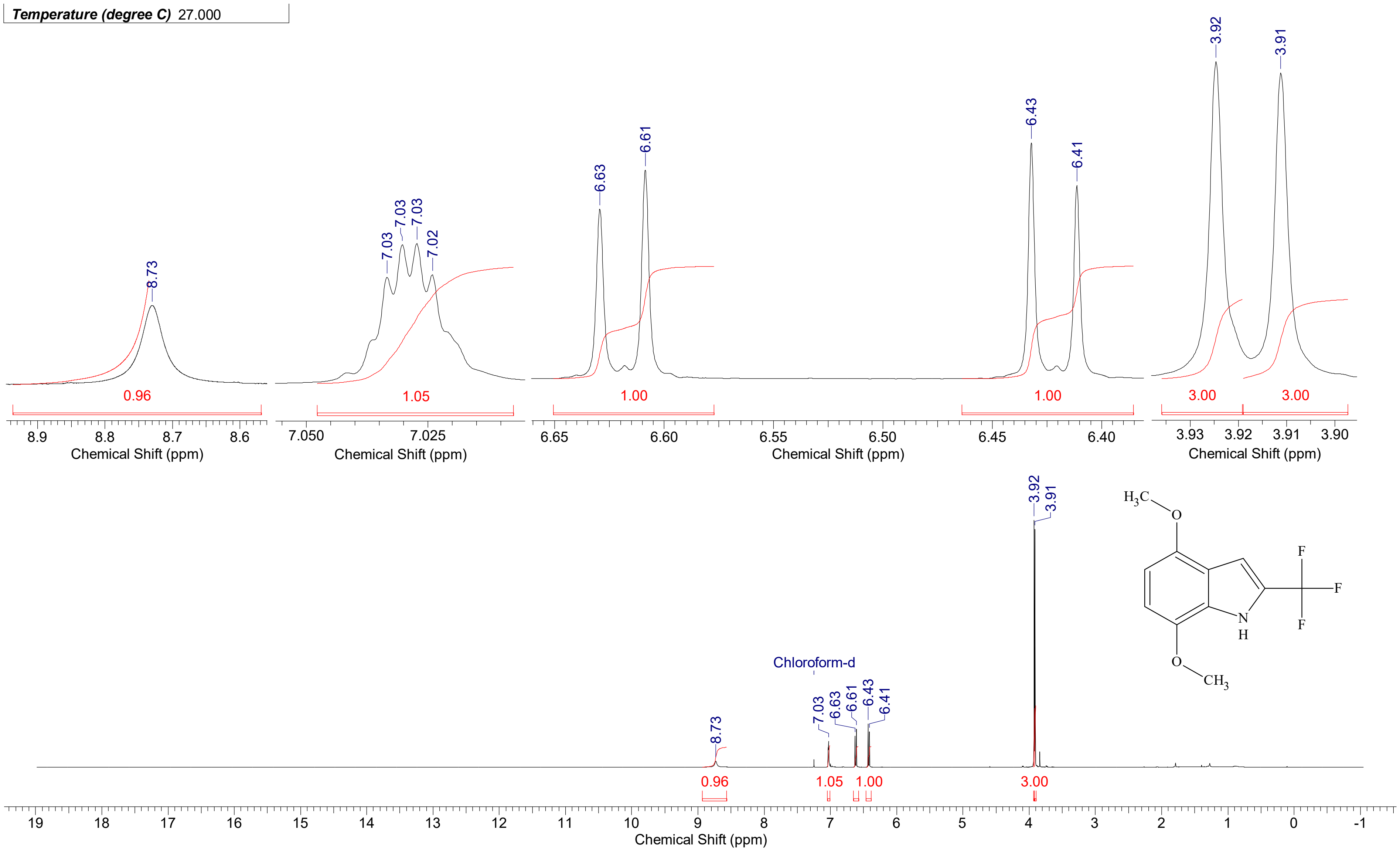

^{1}H NMR spectrum of **4g** (400.1 MHz, $CDCl_3$)

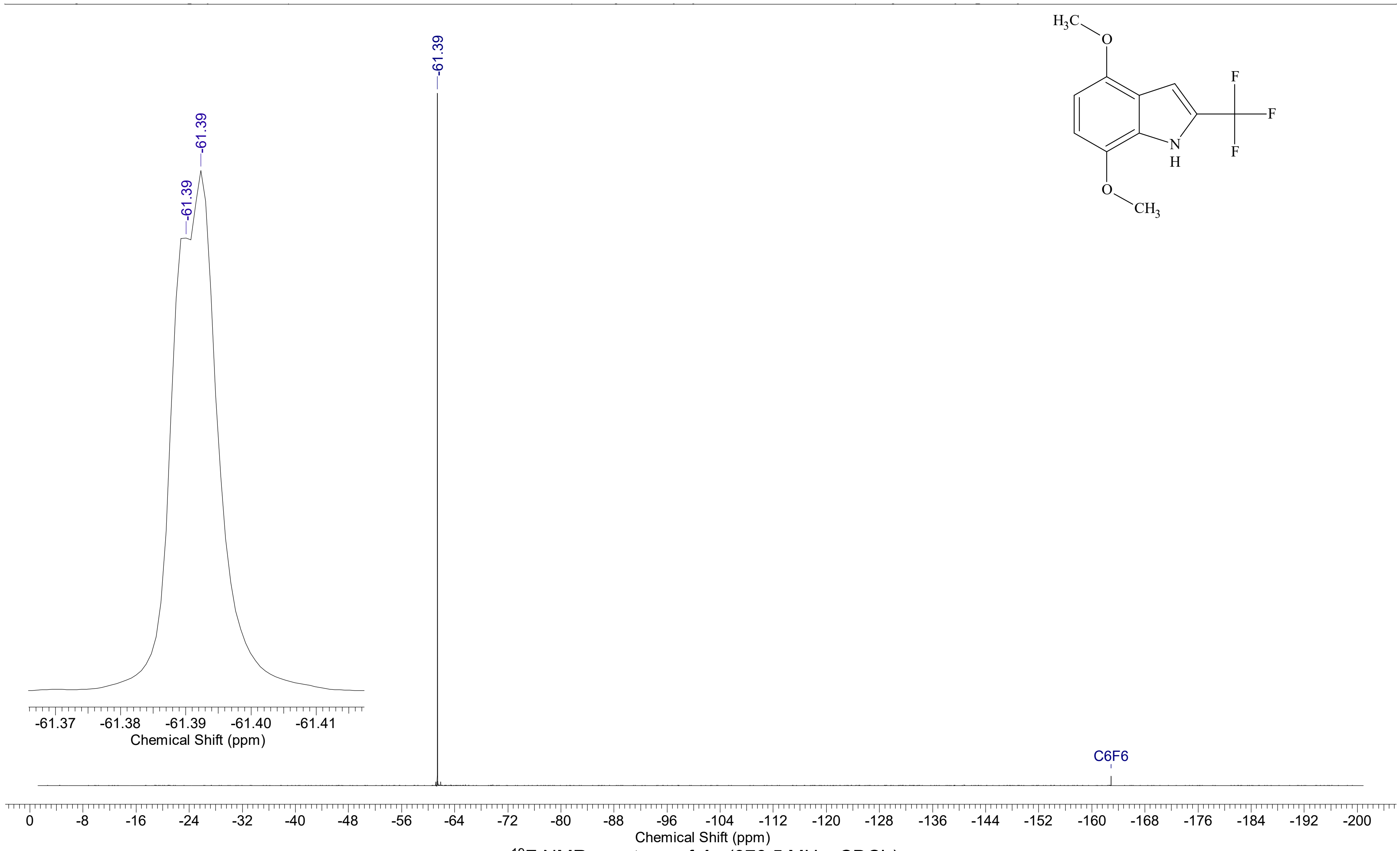

^{19}F NMR spectrum of **4g** (376.5 MHz, $CDCl_3$)

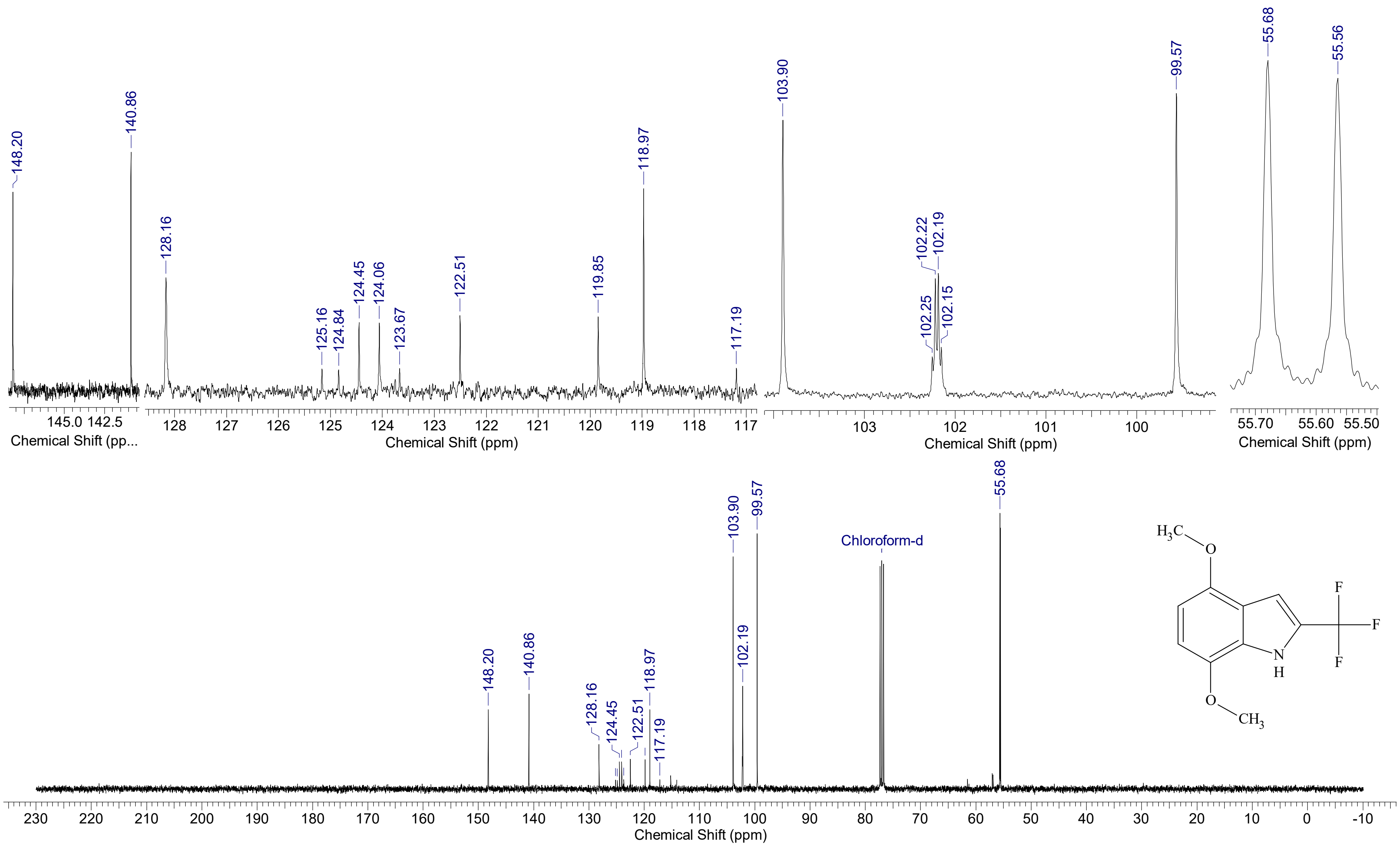

$^{13}C\{^1H\}$ NMR spectrum of **4g** (100.6 MHz, $CDCl_3$)

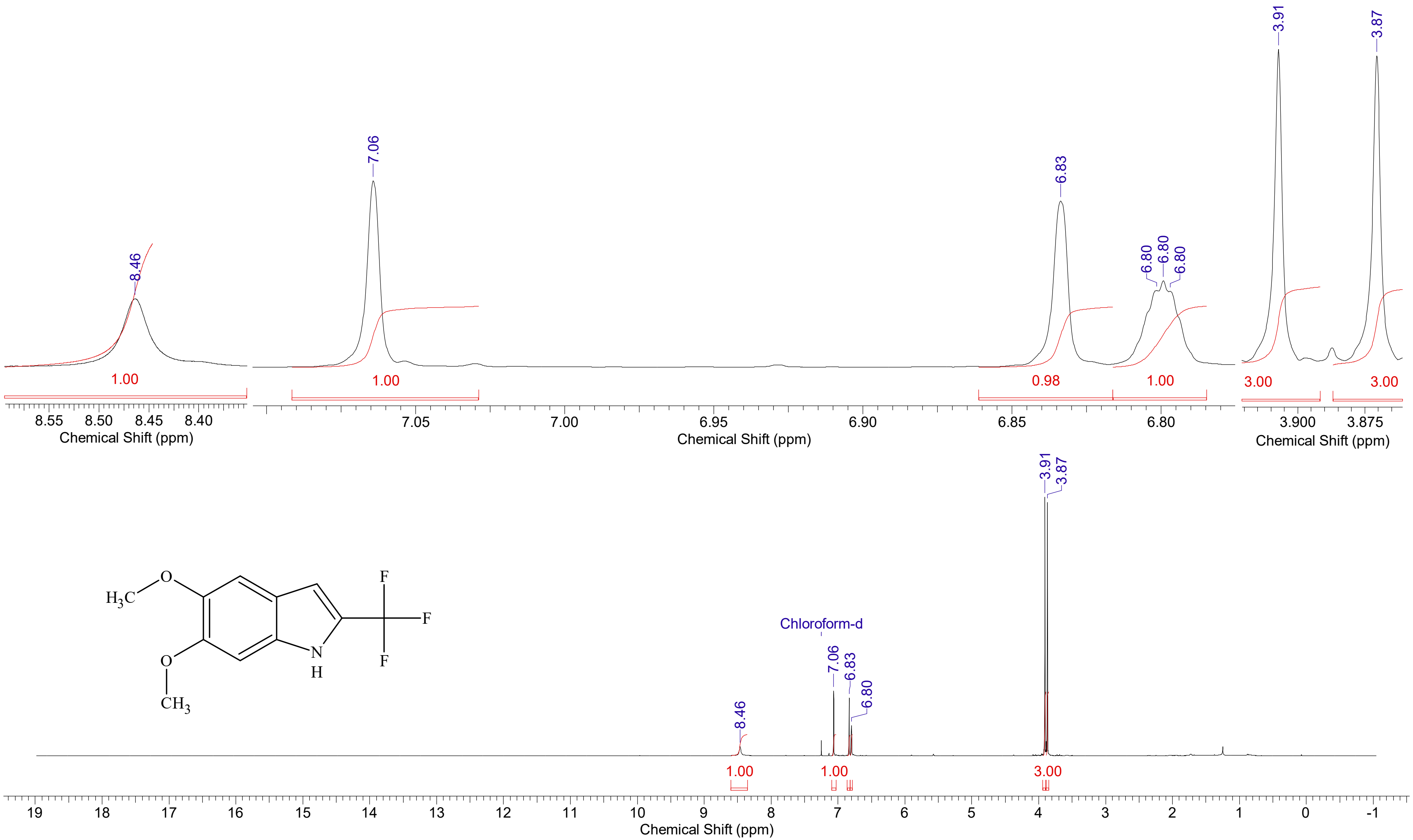

^{1}H NMR spectrum of **4h** (400.1 MHz, $CDCl_3$)

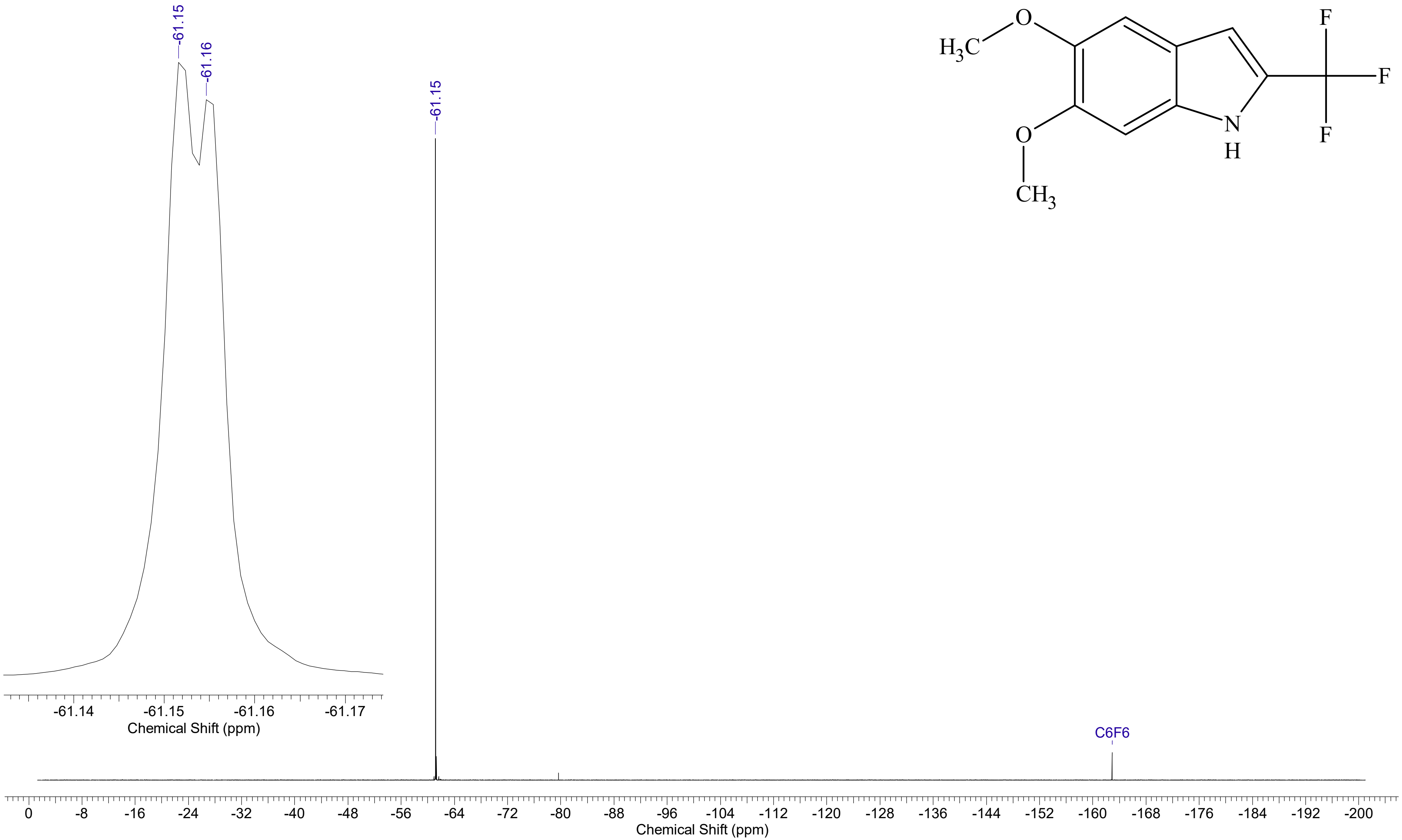

^{19}F NMR spectrum of **4h** (376.5 MHz, $CDCl_3$)

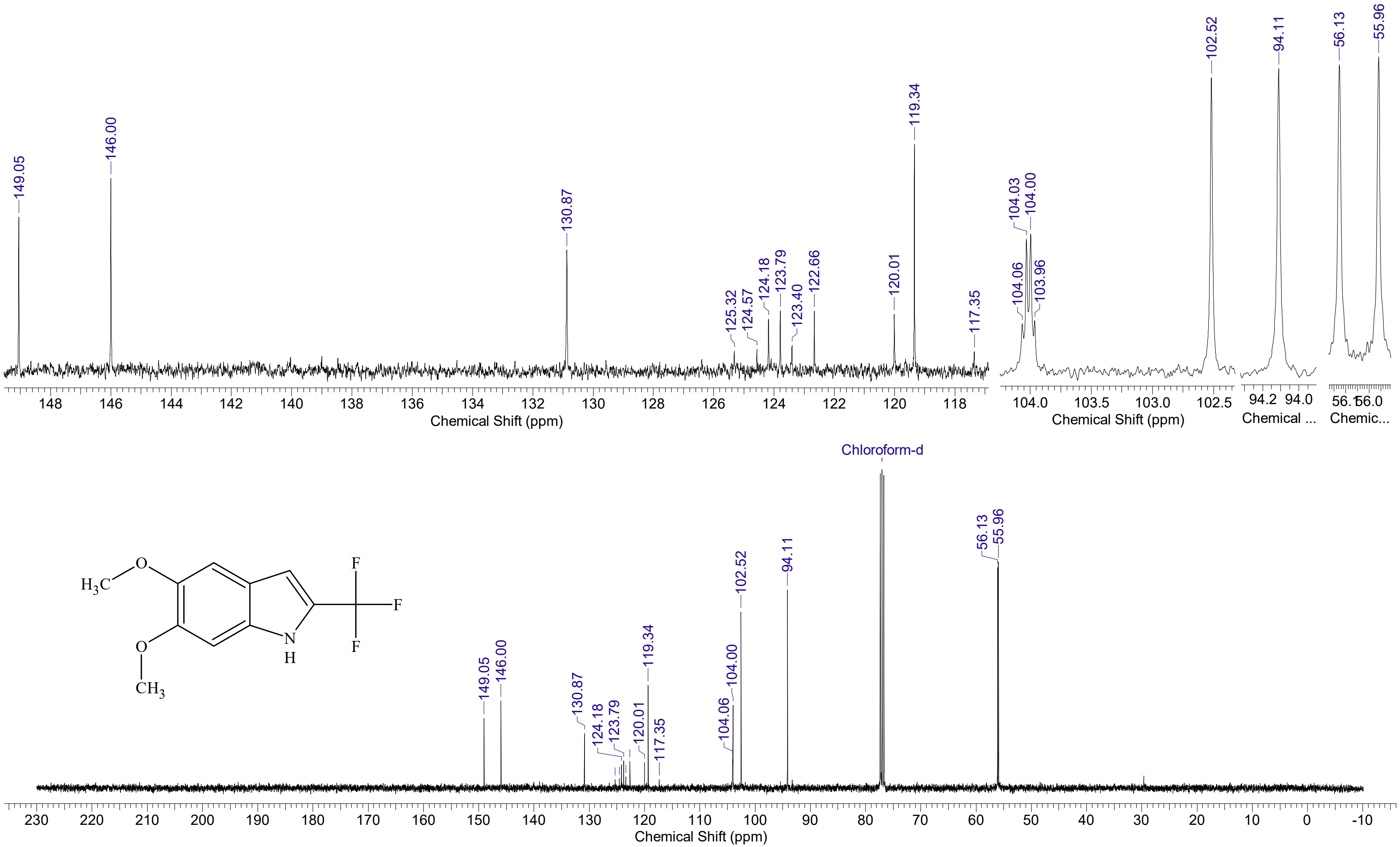

$^{13}C\{^{1}H\}$ NMR spectrum of **4h** (100.6 MHz, $CDCl_3$)

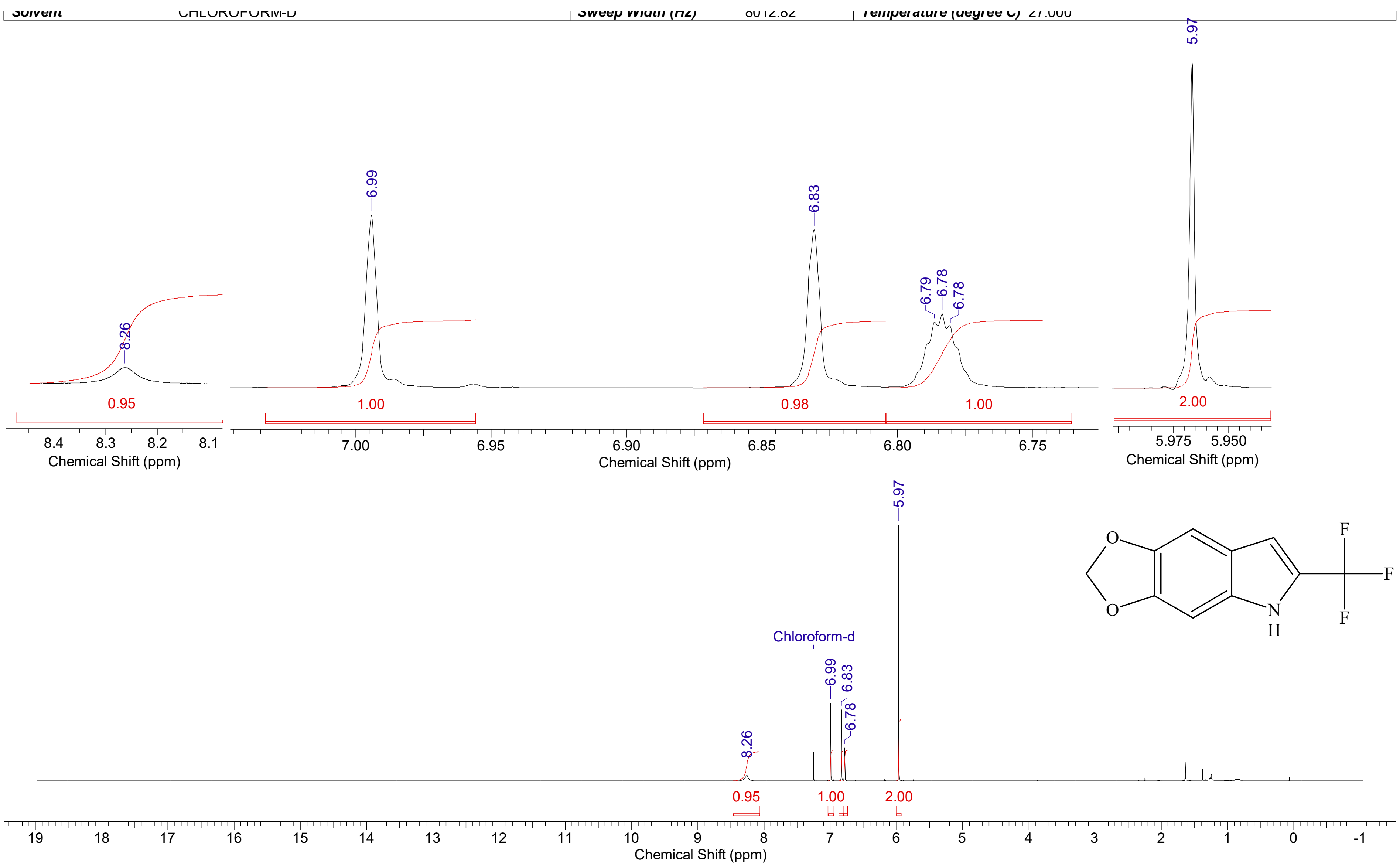

^{1}H NMR spectrum of **4i** (400.1 MHz, $CDCl_3$)

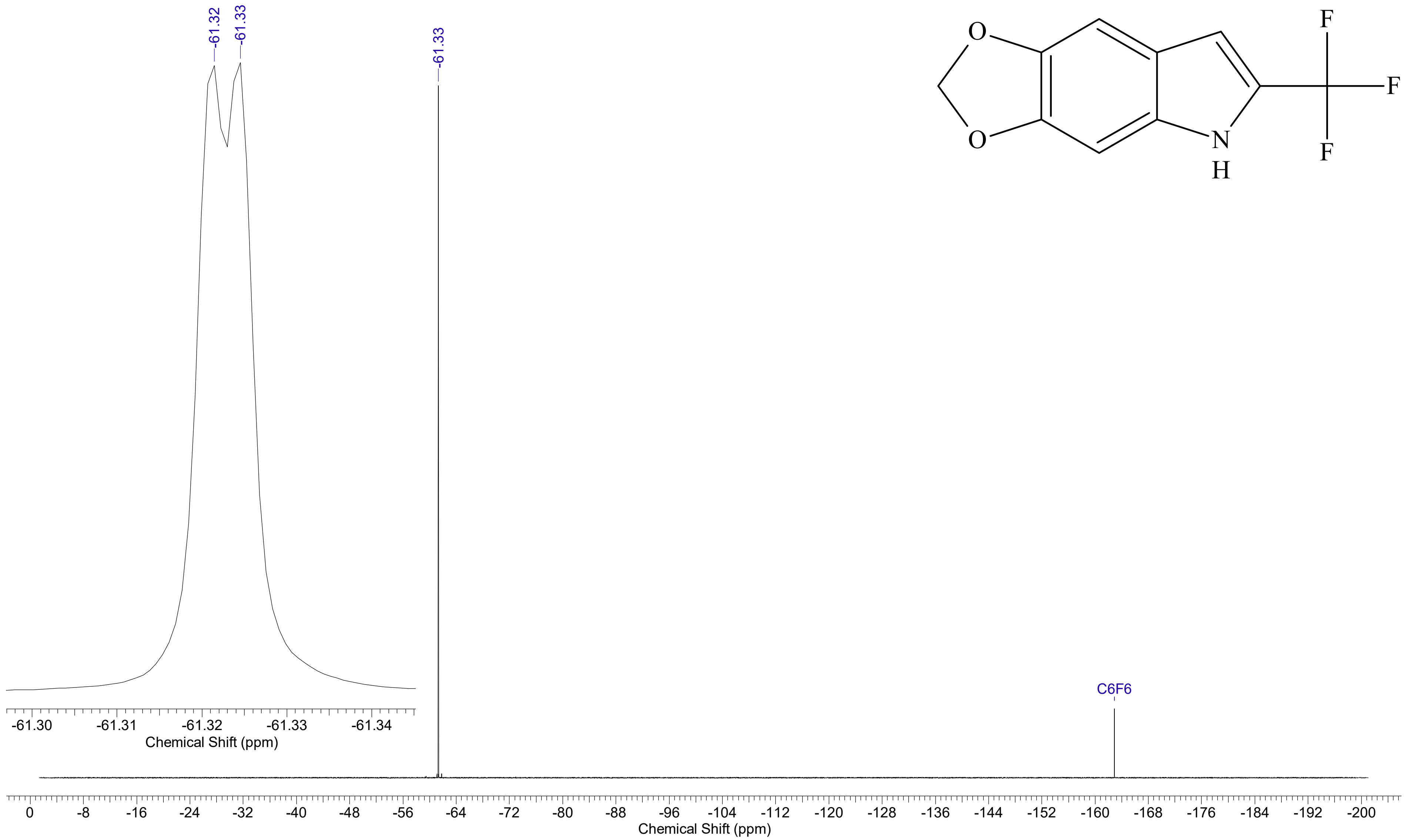

^{19}F NMR spectrum of **4i** (376.5 MHz, $CDCl_3$)

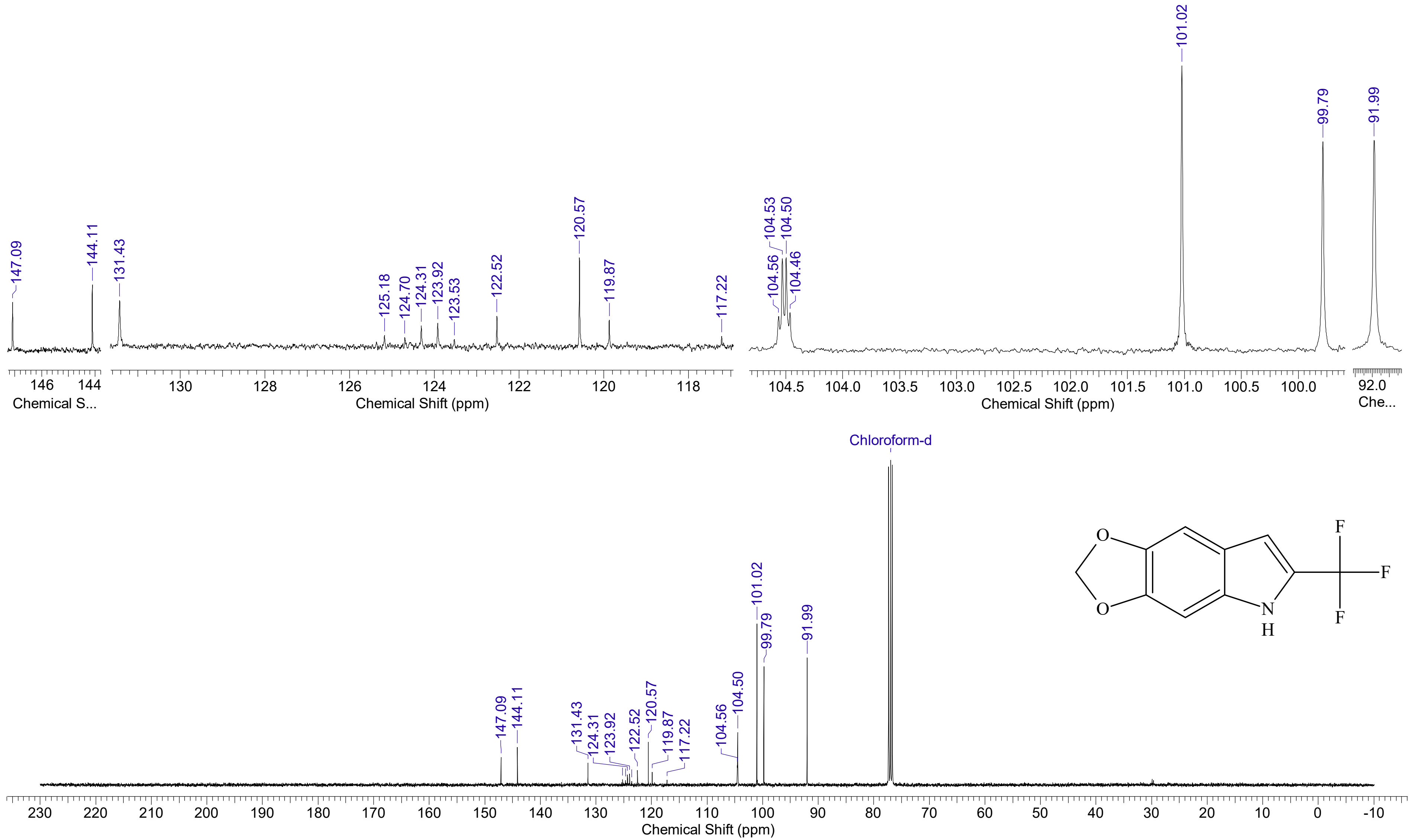

$^{13}C\{^1H\}$ NMR spectrum of **4i** (100.6 MHz, $CDCl_3$)

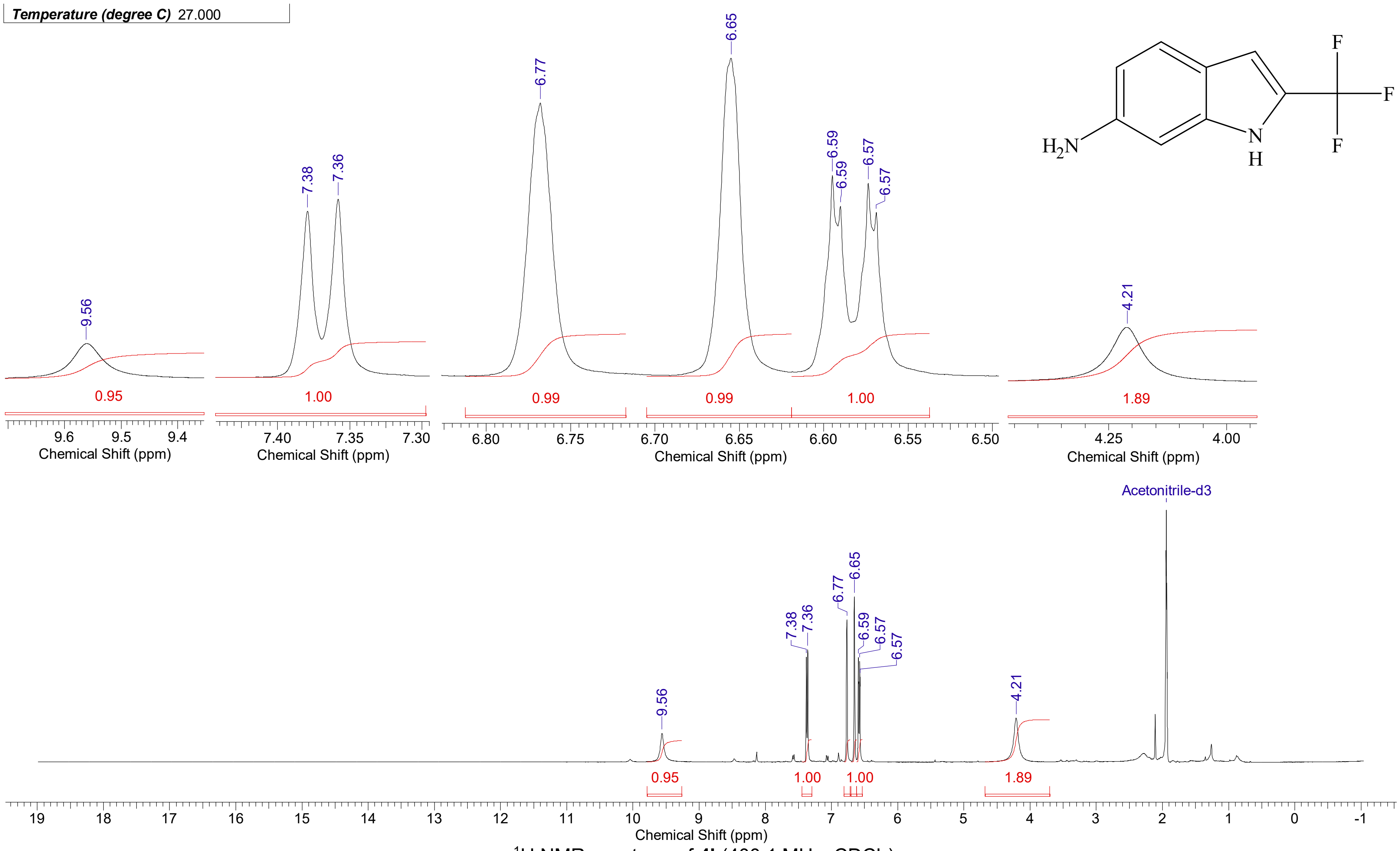

^{1}H NMR spectrum of **4j** (400.1 MHz, $CDCl_3$)

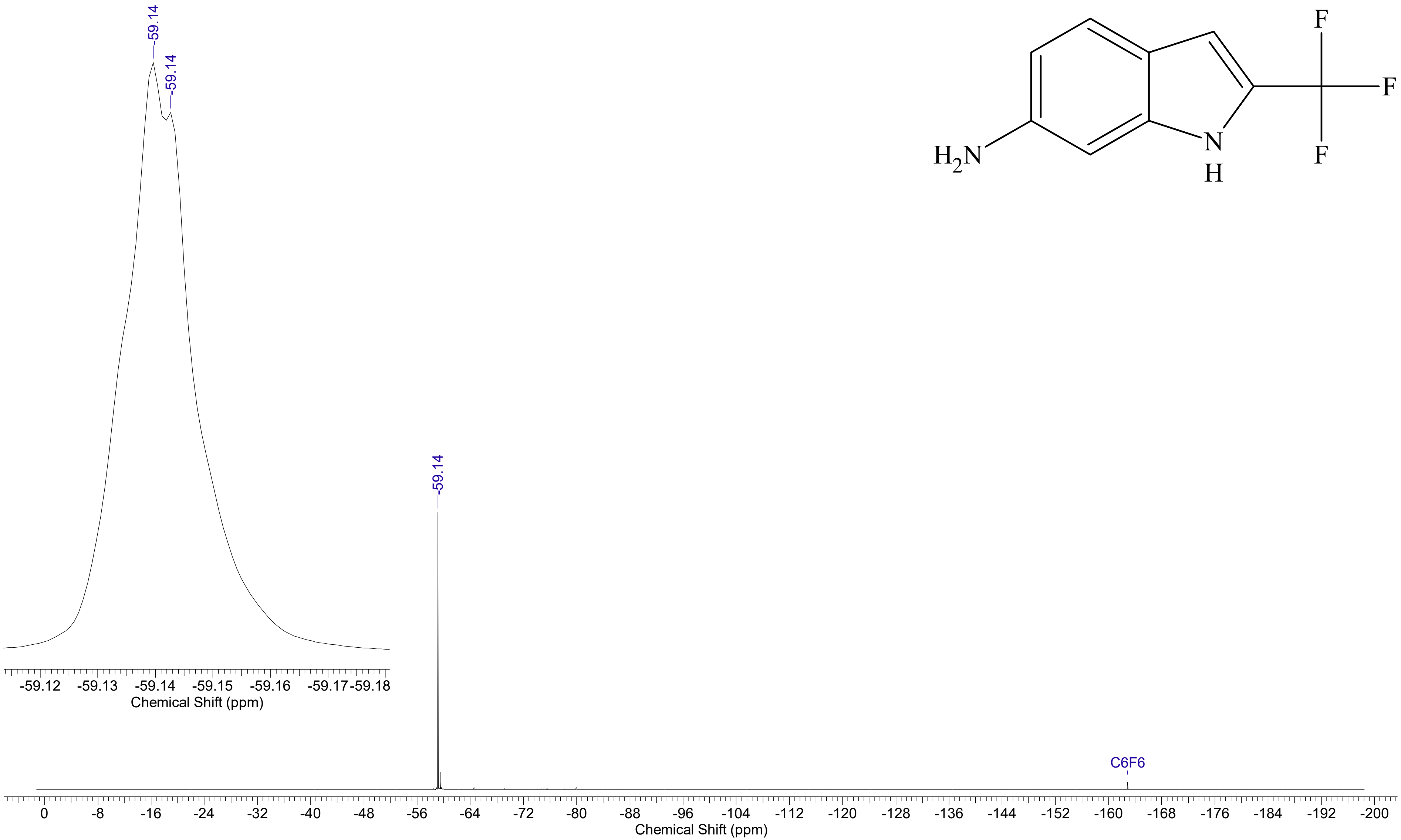

^{19}F NMR spectrum of **4j** (376.5 MHz $CDCl_3$)

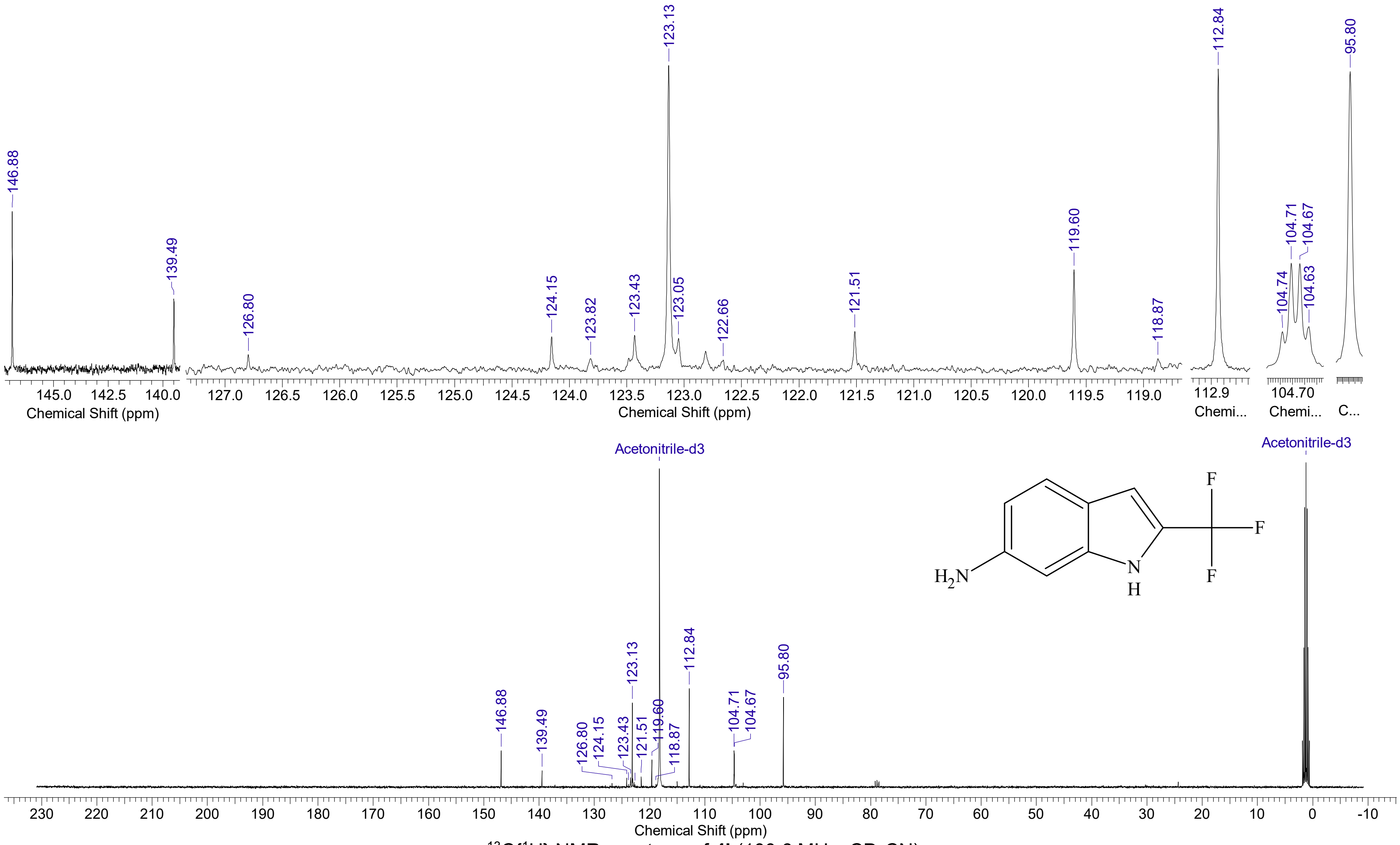

$^{13}C\{^{1}H\}$ NMR spectrum of **4j** (100.6 MHz, CD_3CN)

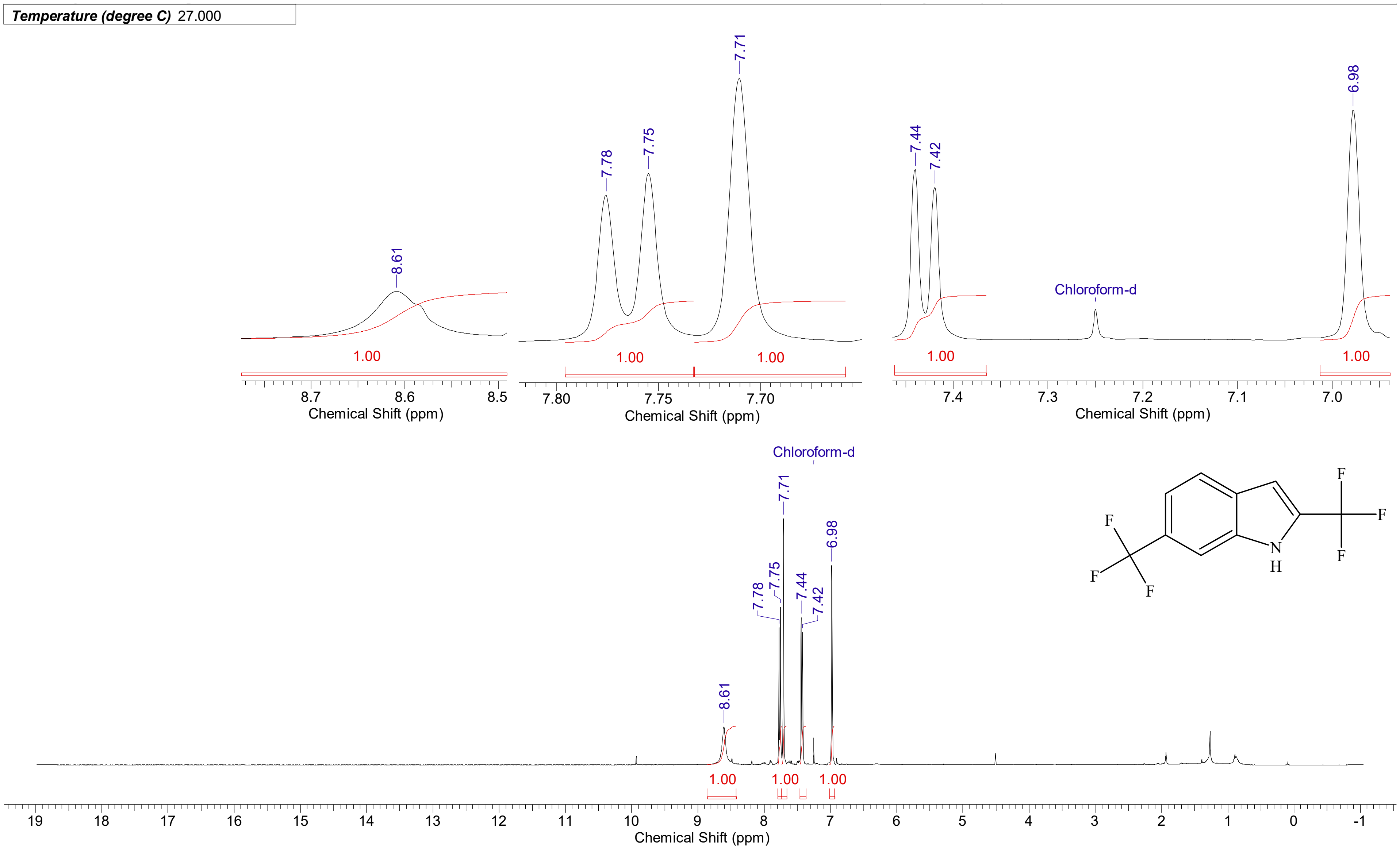

^{1}H NMR spectrum of **4k** (400.1 MHz, $CDCl_3$)

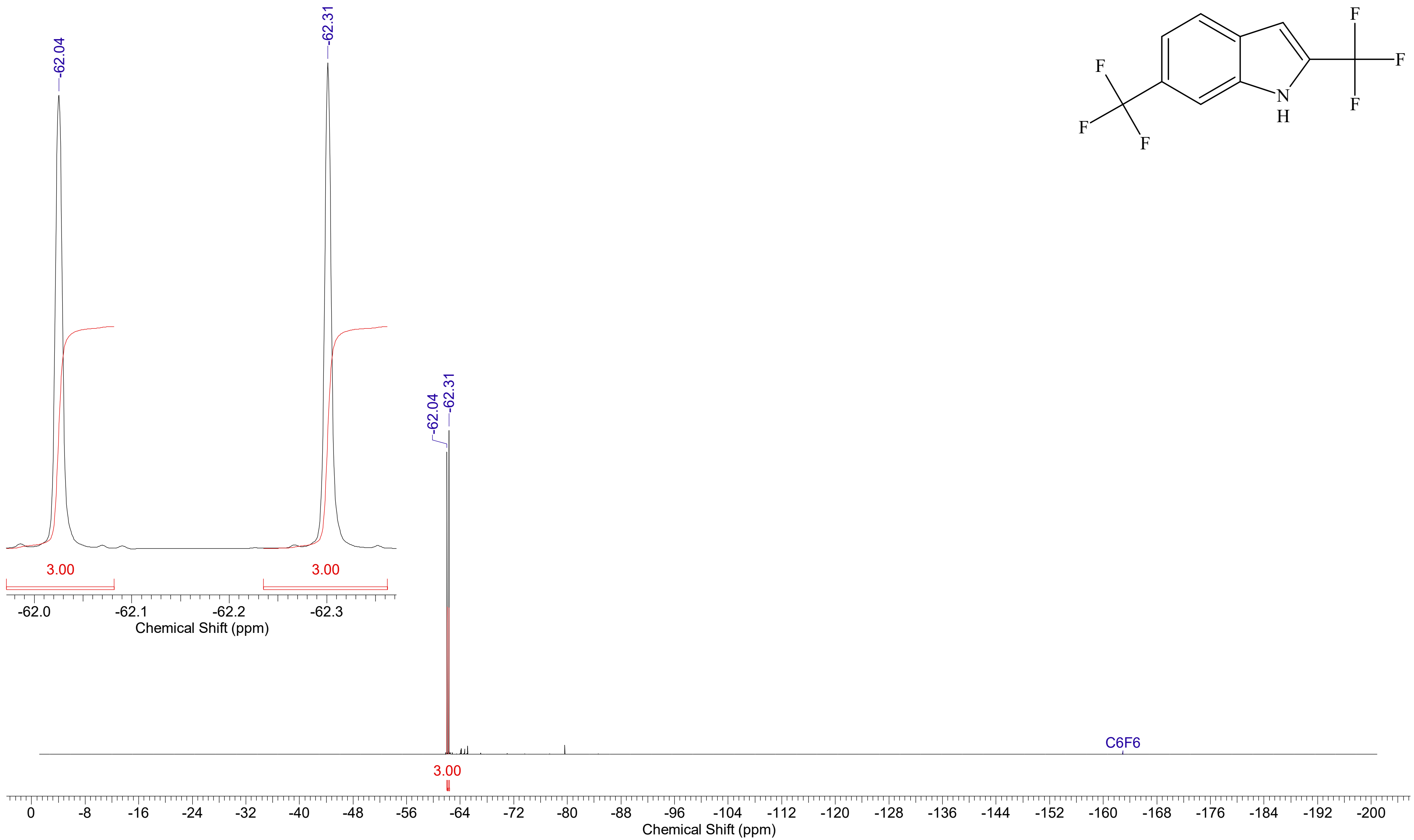

^{19}F NMR spectrum of **4k** (376.5 MHz, $CDCl_3$)

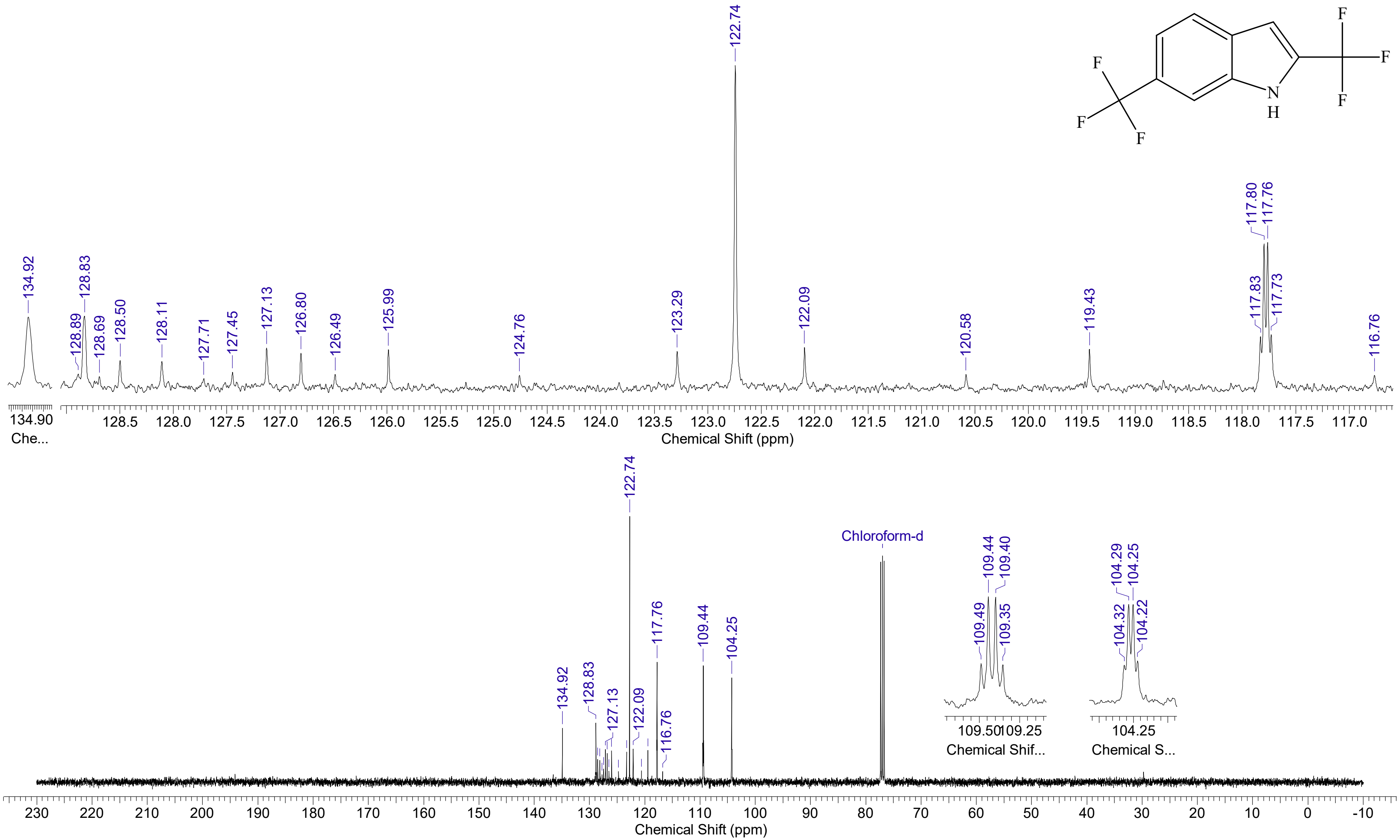

$^{13}C\{^{1}H\}$ NMR spectrum of **4k** (100.6 MHz, $CDCl_3$)

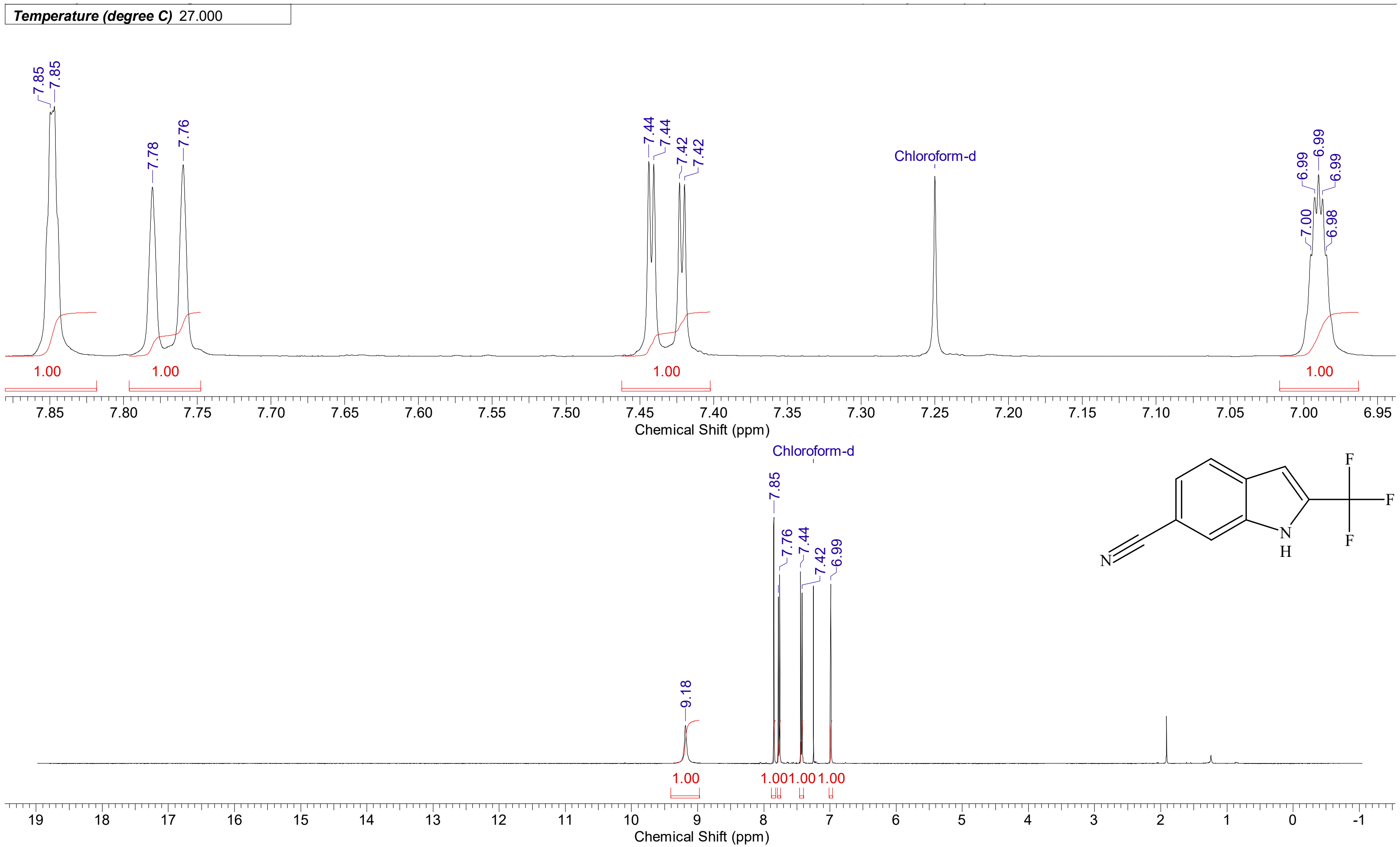

^{1}H NMR spectrum of **4l** (400.1 MHz, $CDCl_3$)

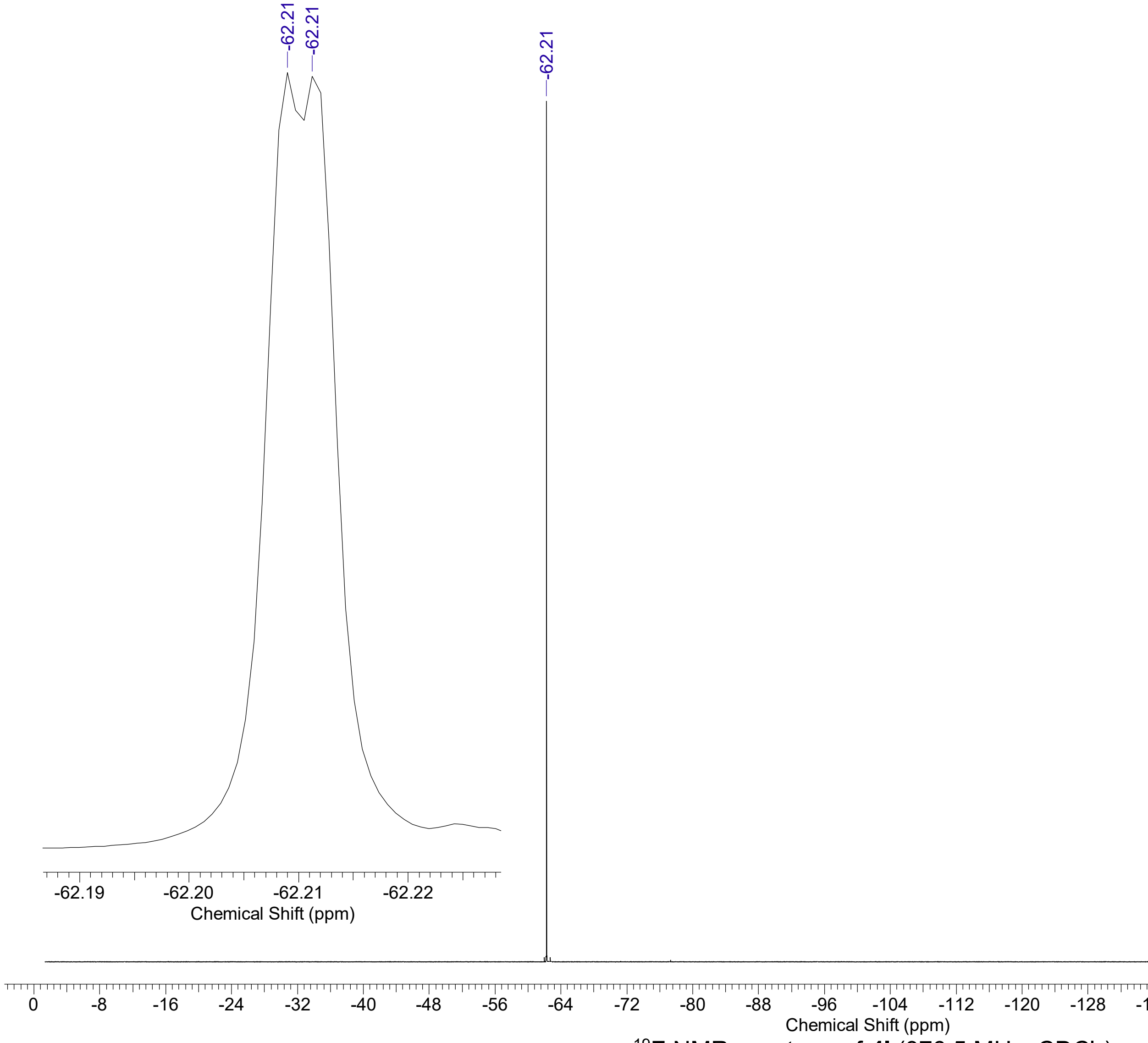

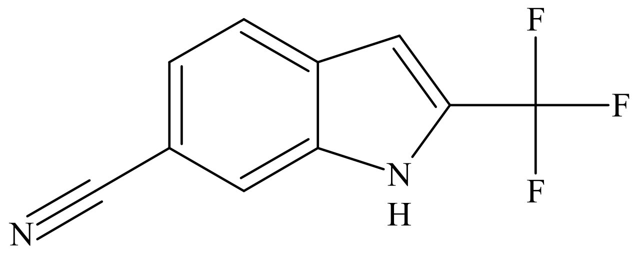

^{19}F NMR spectrum of **4l** (376.5 MHz, $CDCl_3$)

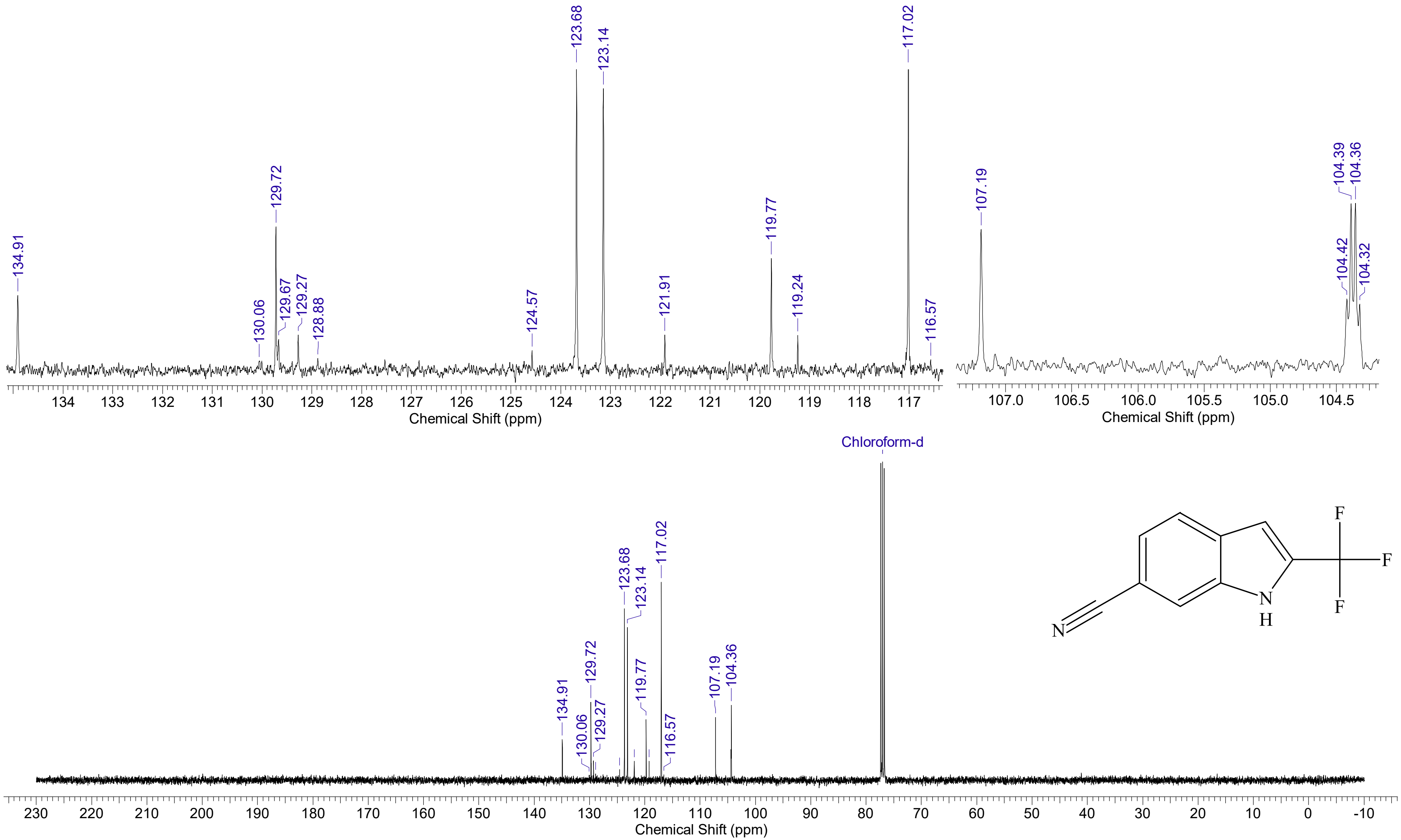

$^{13}C\{^{1}H\}$ NMR spectrum of **4l** (100.6 MHz, $CDCl_3$)

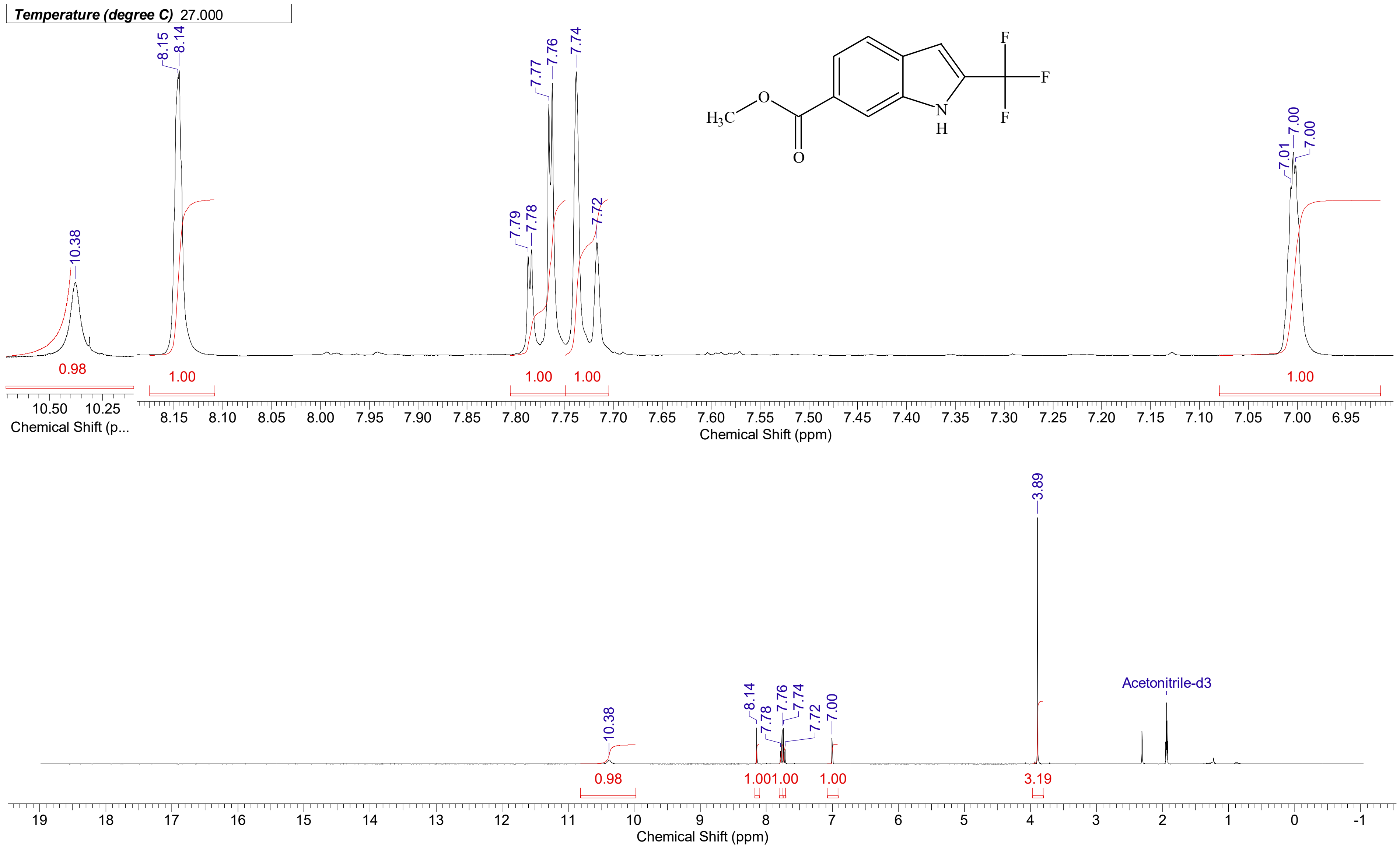

^{1}H NMR spectrum of **4m** (400.1 MHz, $CDCl_3$)

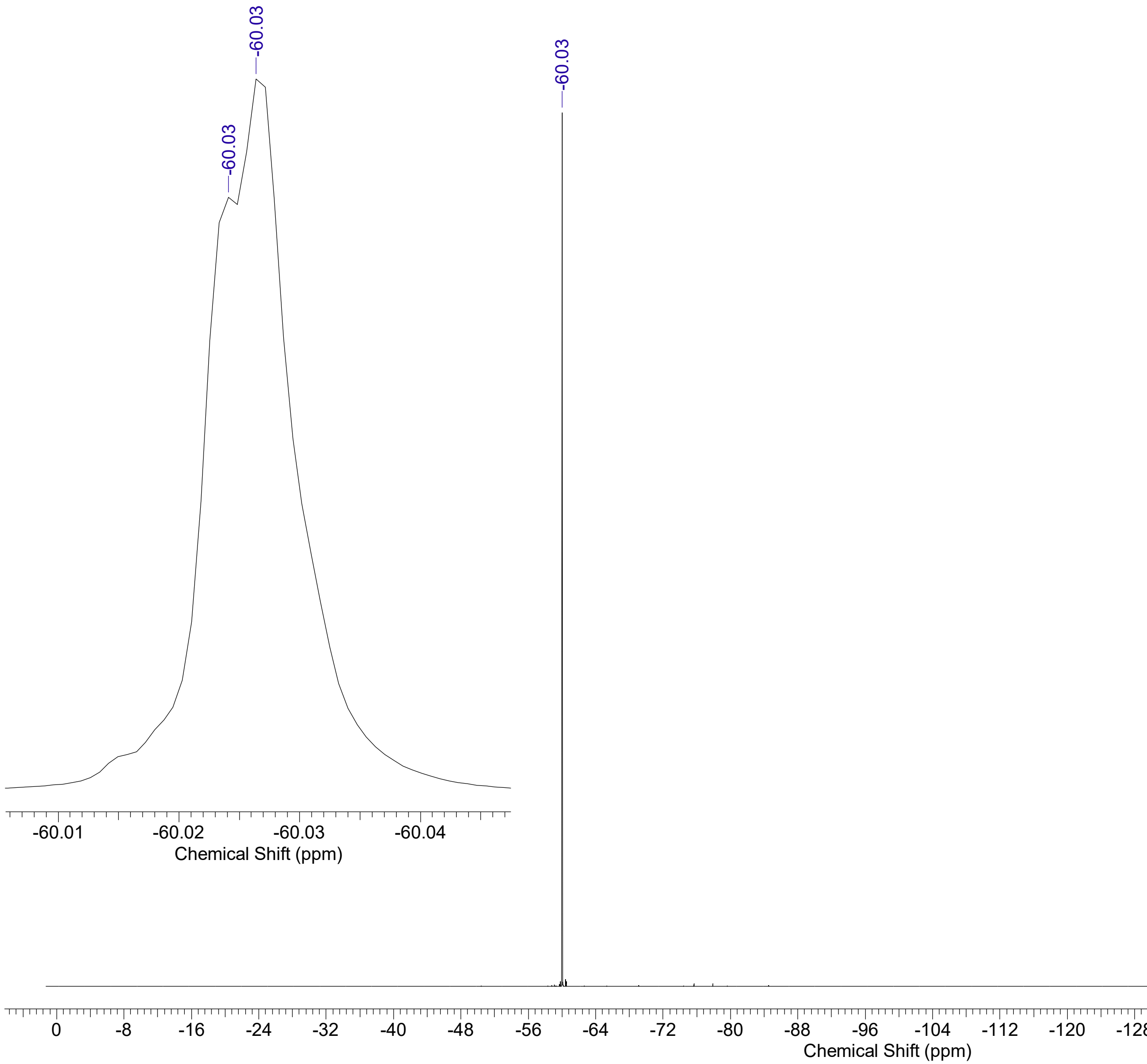

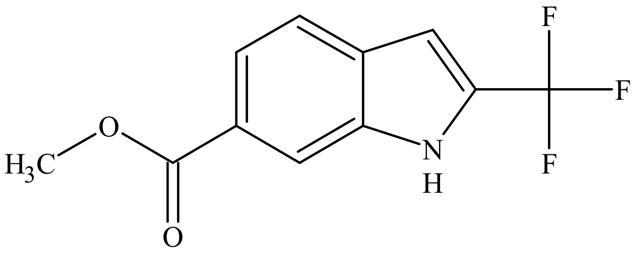

^{19}F NMR spectrum of **4m** (376.5 MHz, $CDCl_3$)

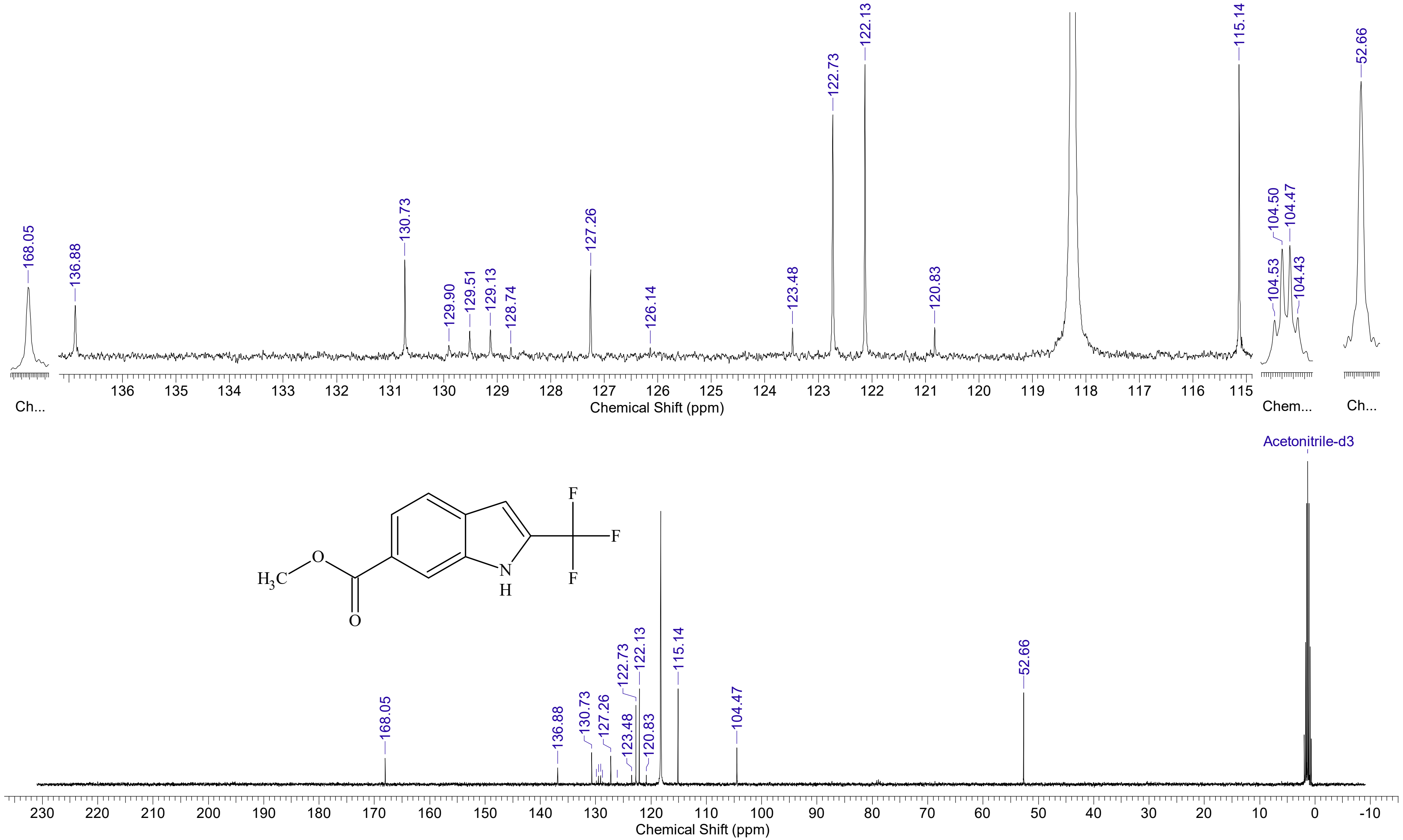

$^{13}C\{^1H\}$ NMR spectrum of **4m** (100.6 MHz, $CDCl_3$)

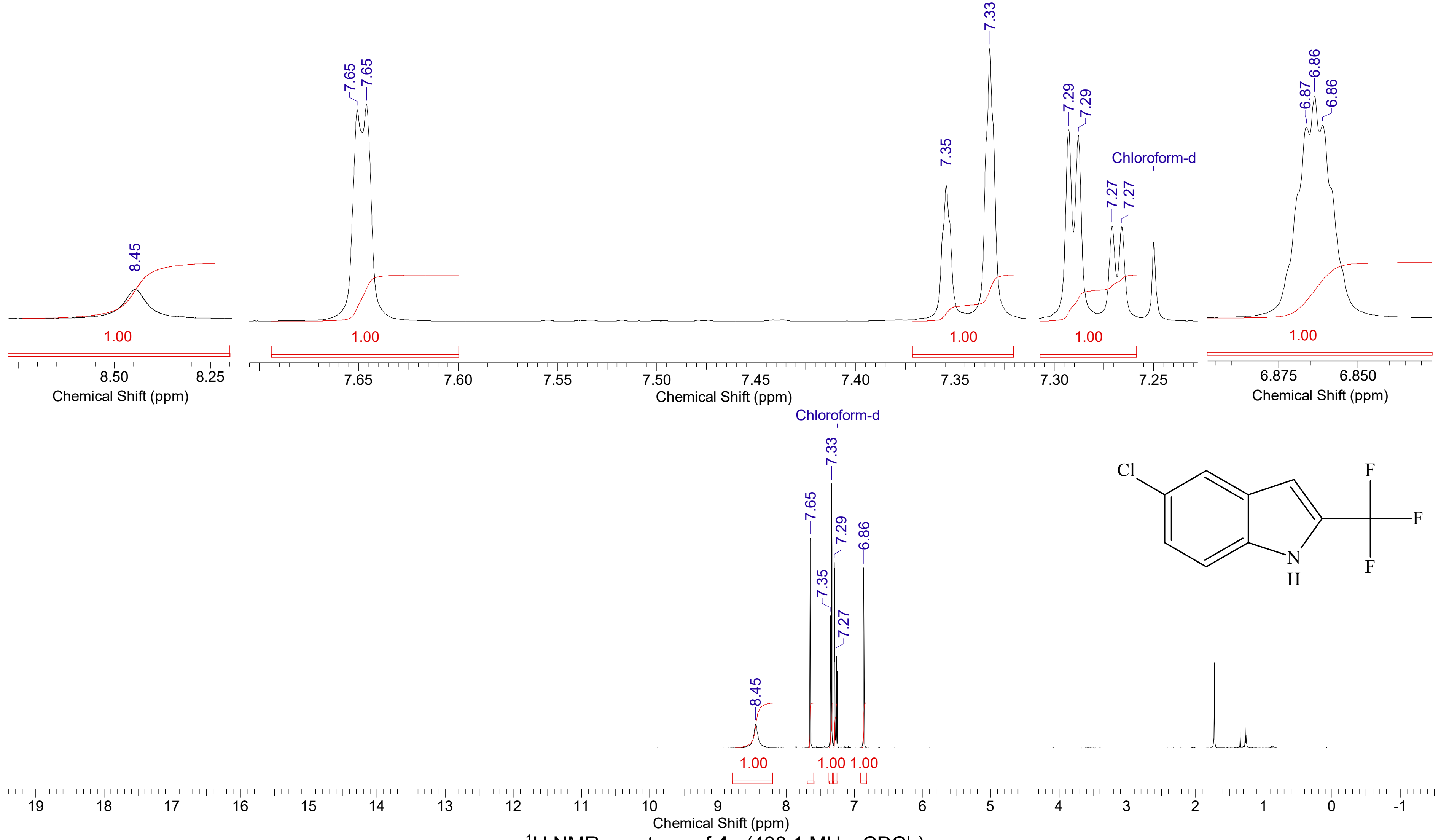

^{1}H NMR spectrum of **4n** (400.1 MHz, $CDCl_3$)

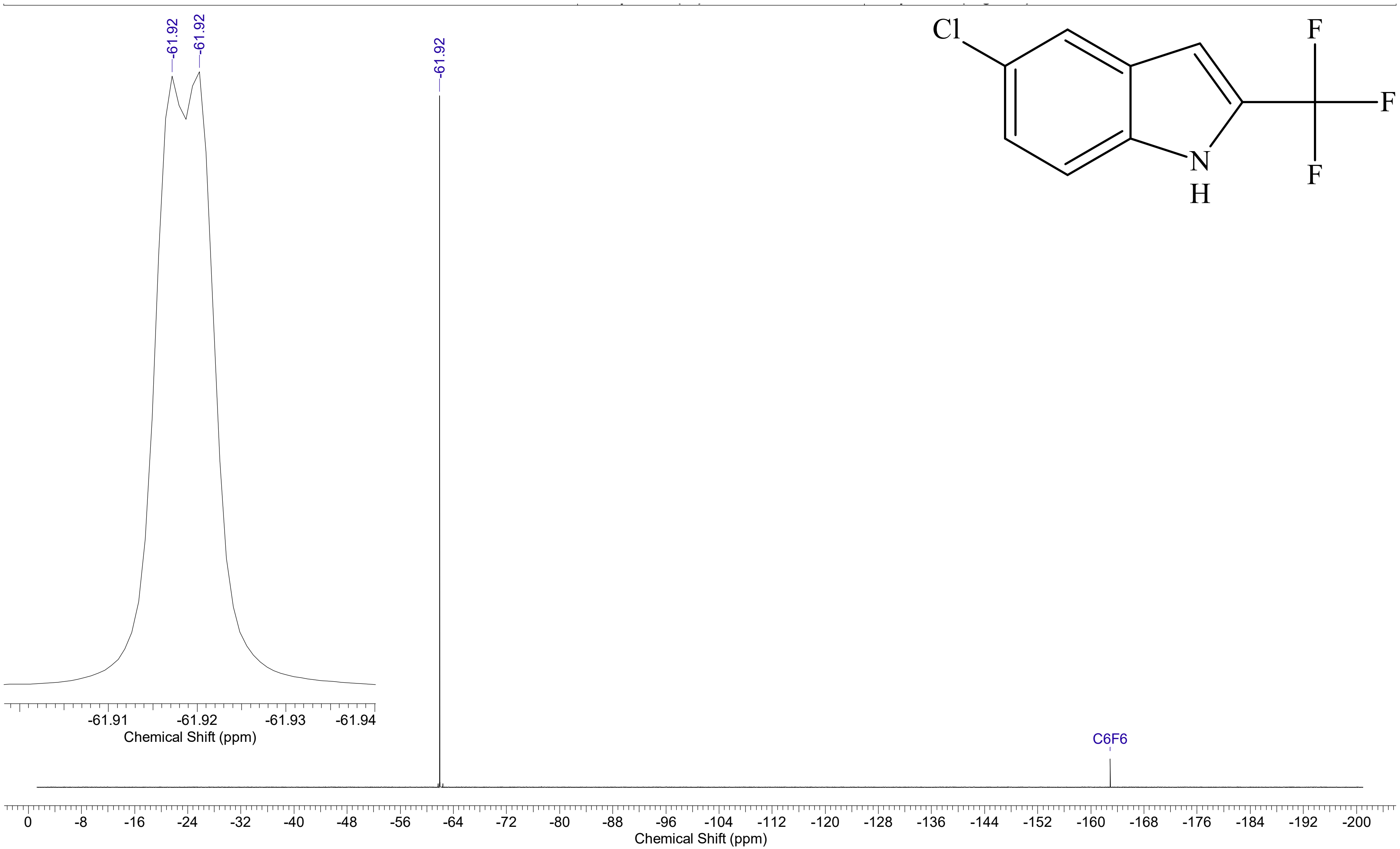

^{19}F NMR spectrum of **4n** (376.5 MHz, $CDCl_3$)

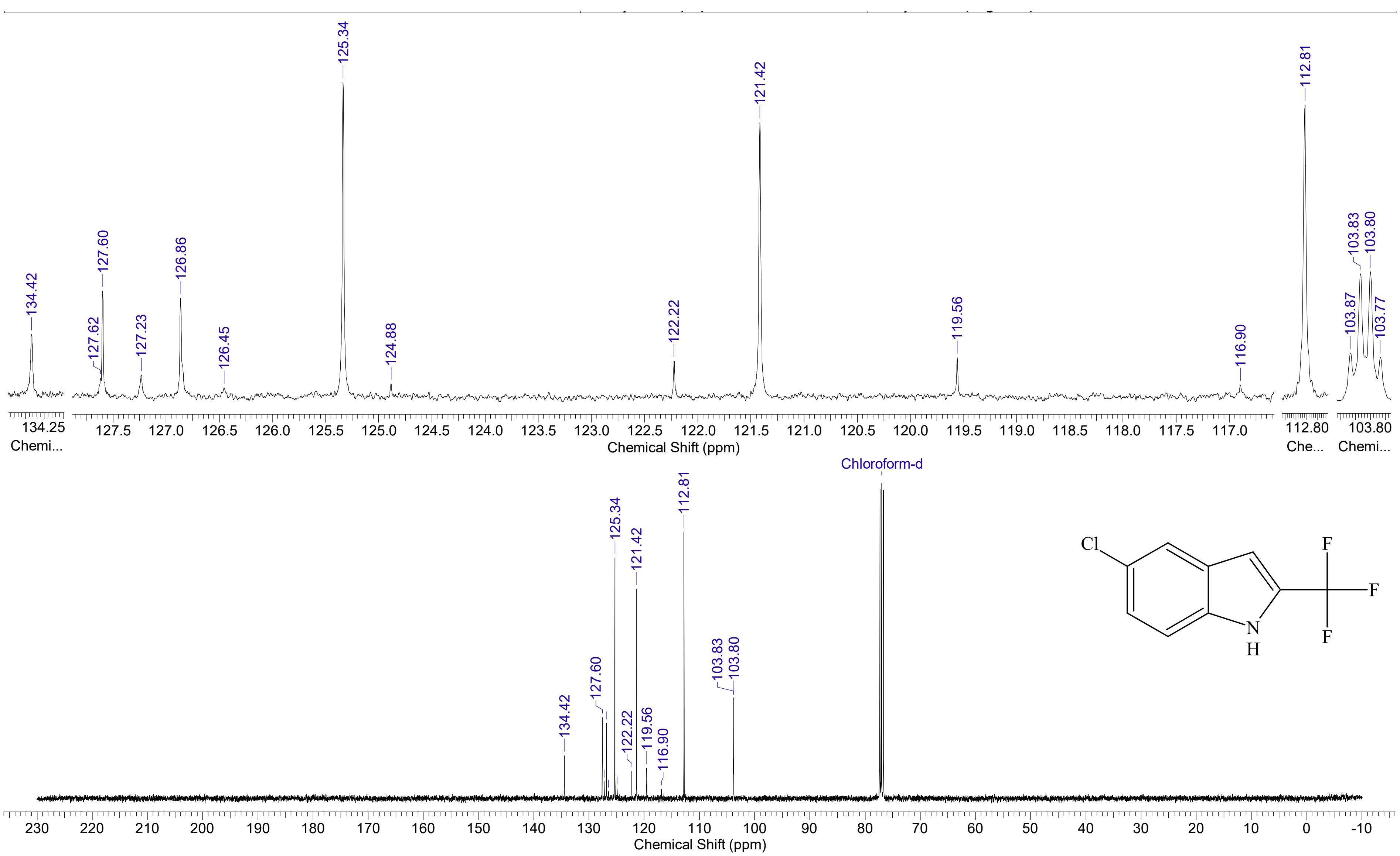

$^{13}C\{^{1}H\}$ NMR spectrum of **4n** (100.6 MHz, $CDCl_3$)

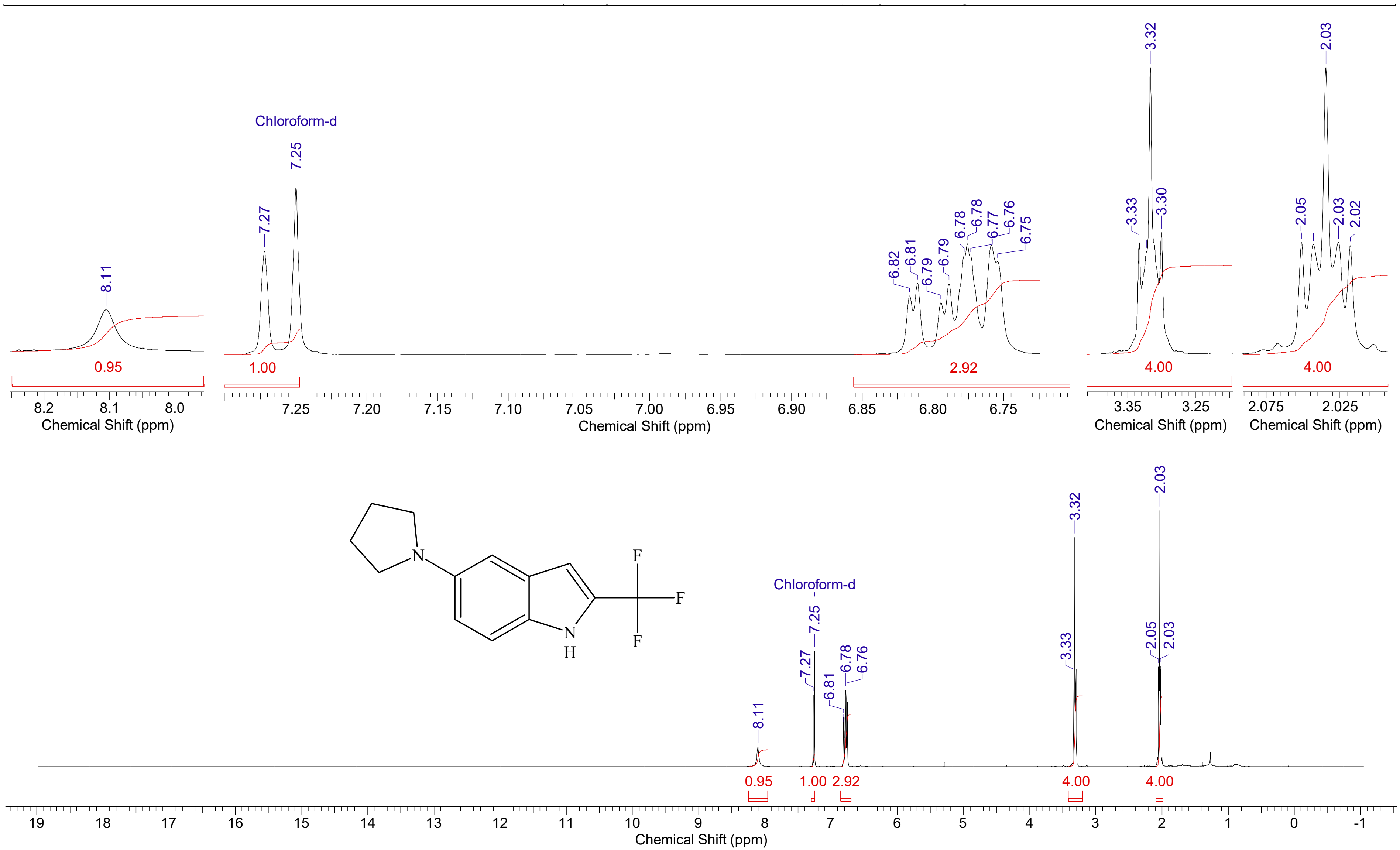

^{1}H NMR spectrum of **10a** (400.1 MHz, $CDCl_3$).

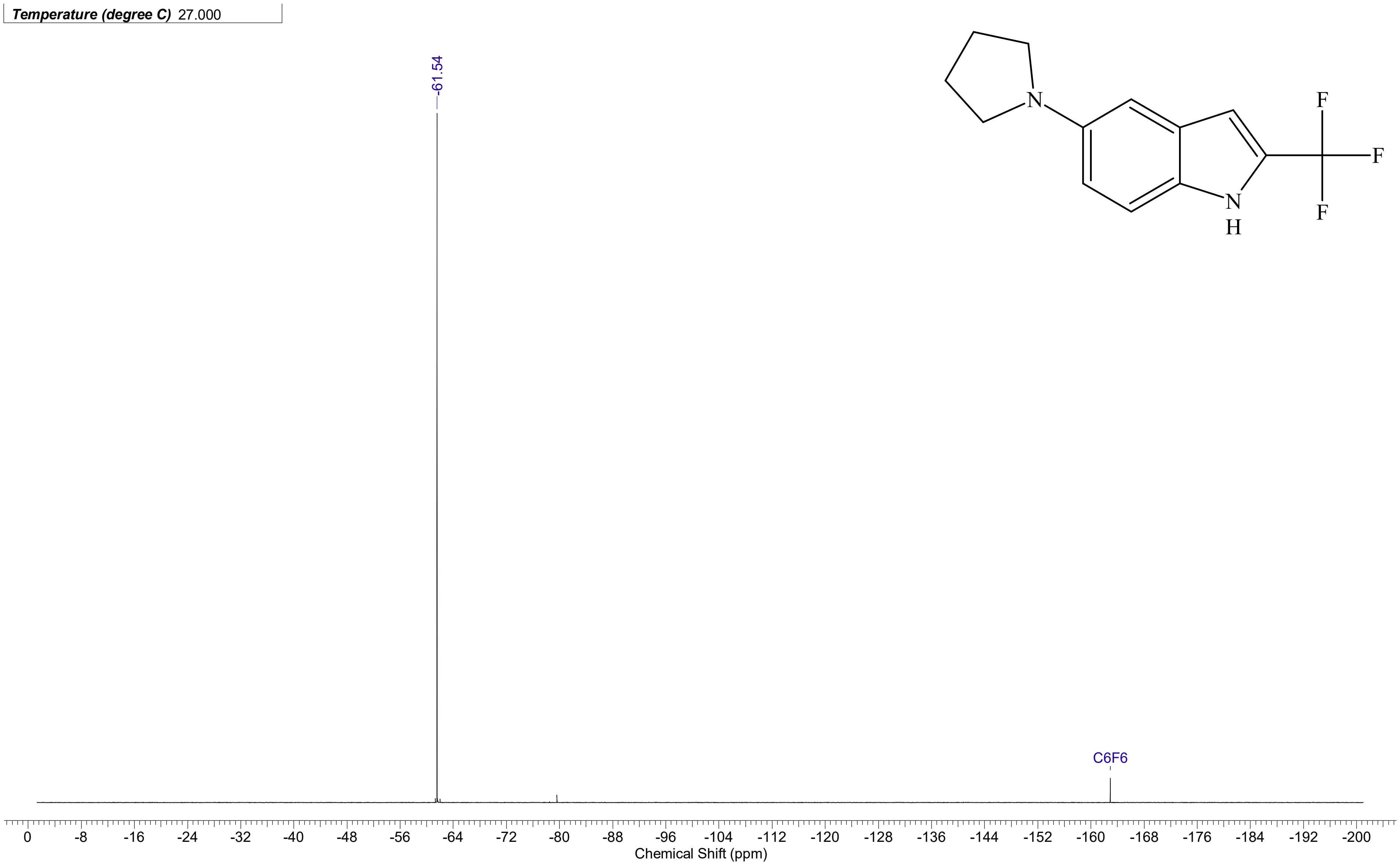

^{19}F NMR spectrum of **10a** (376.5 MHz, $CDCl_3$)

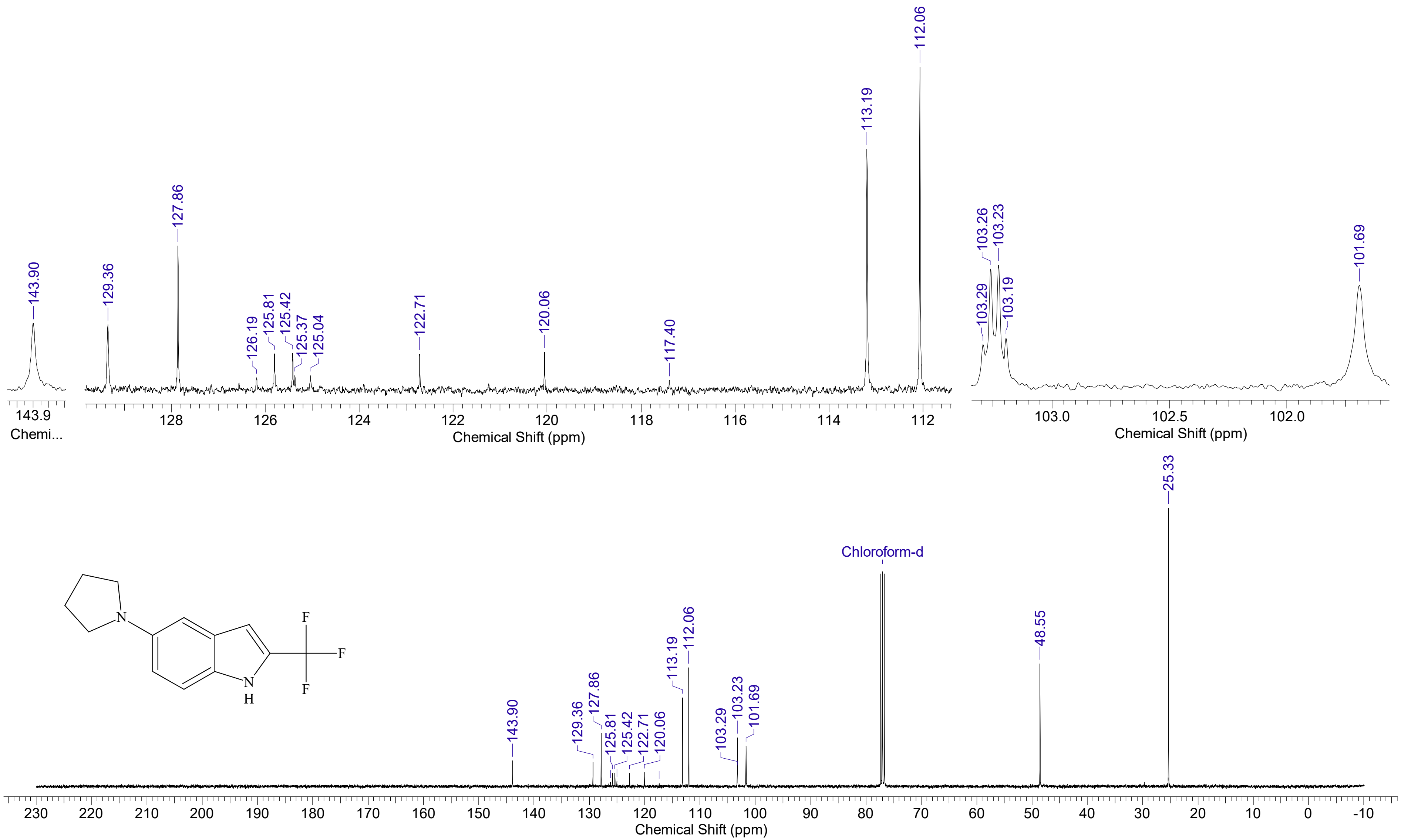

$^{13}C\{^1H\}$ NMR spectrum of **10a** (100.6 MHz, $CDCl_3$)

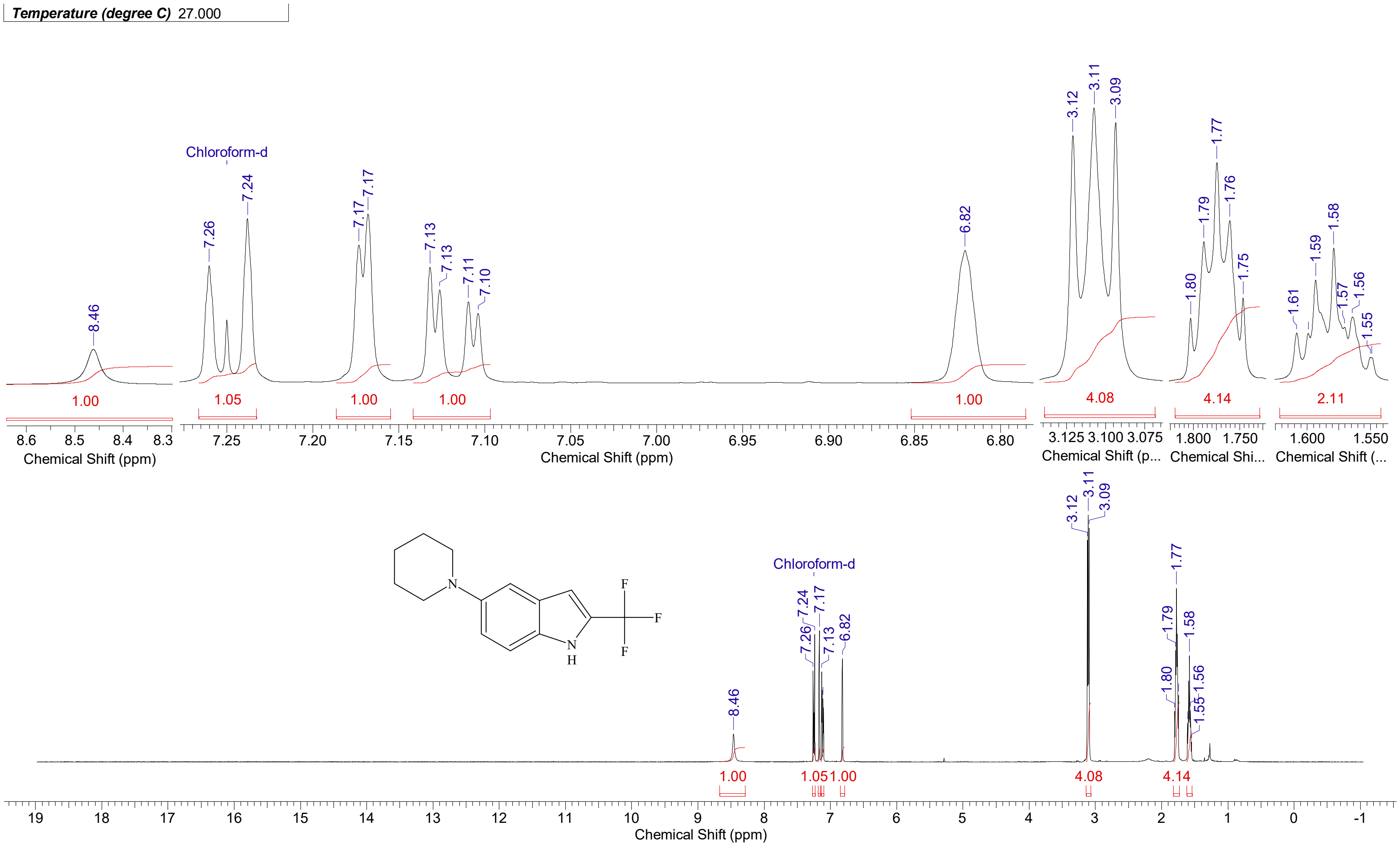

^{1}H NMR spectrum of **10b** (400.1 MHz, $CDCl_3$)

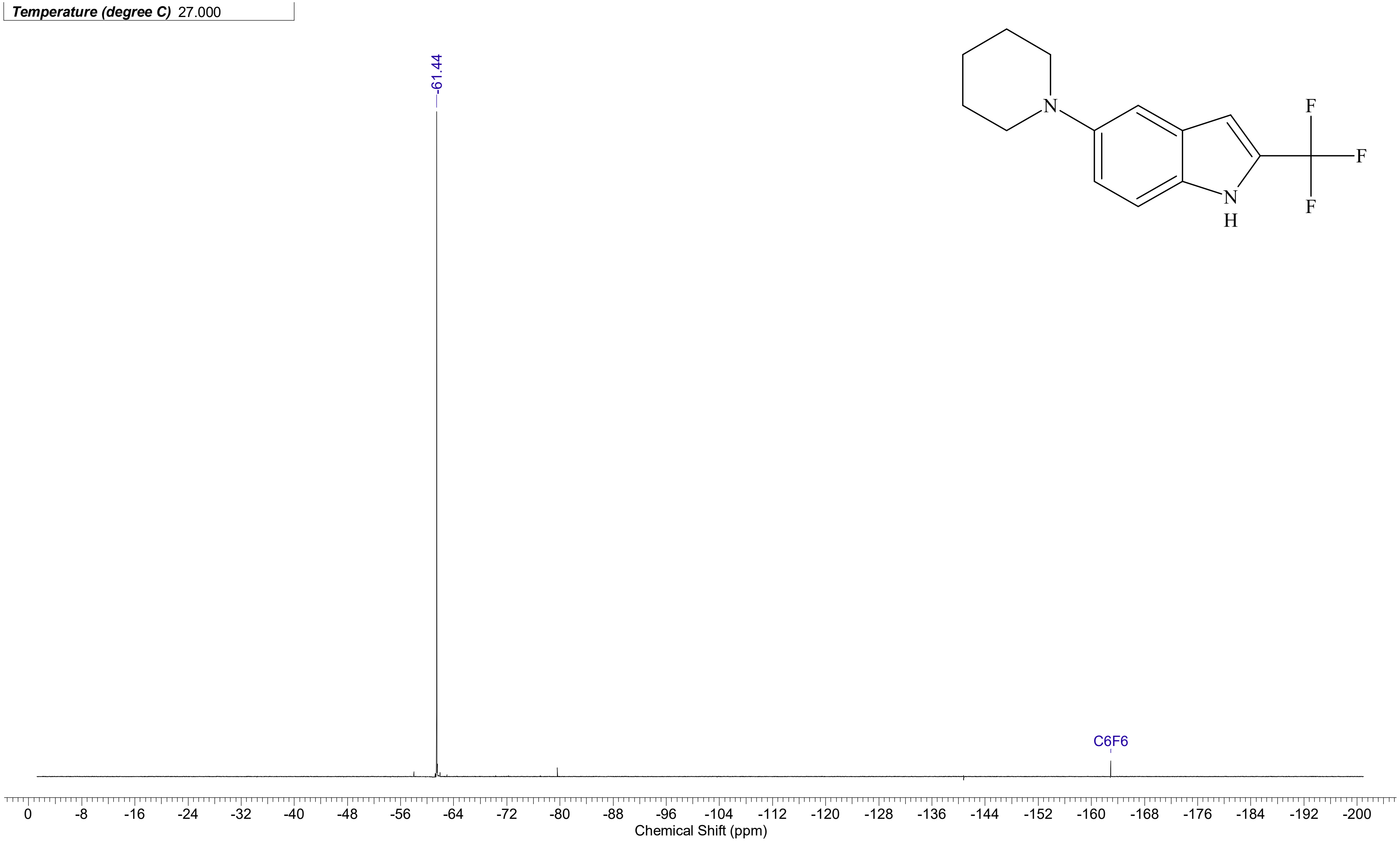

^{19}F NMR spectrum of **10b** (376.5 MHz, $CDCl_3$)

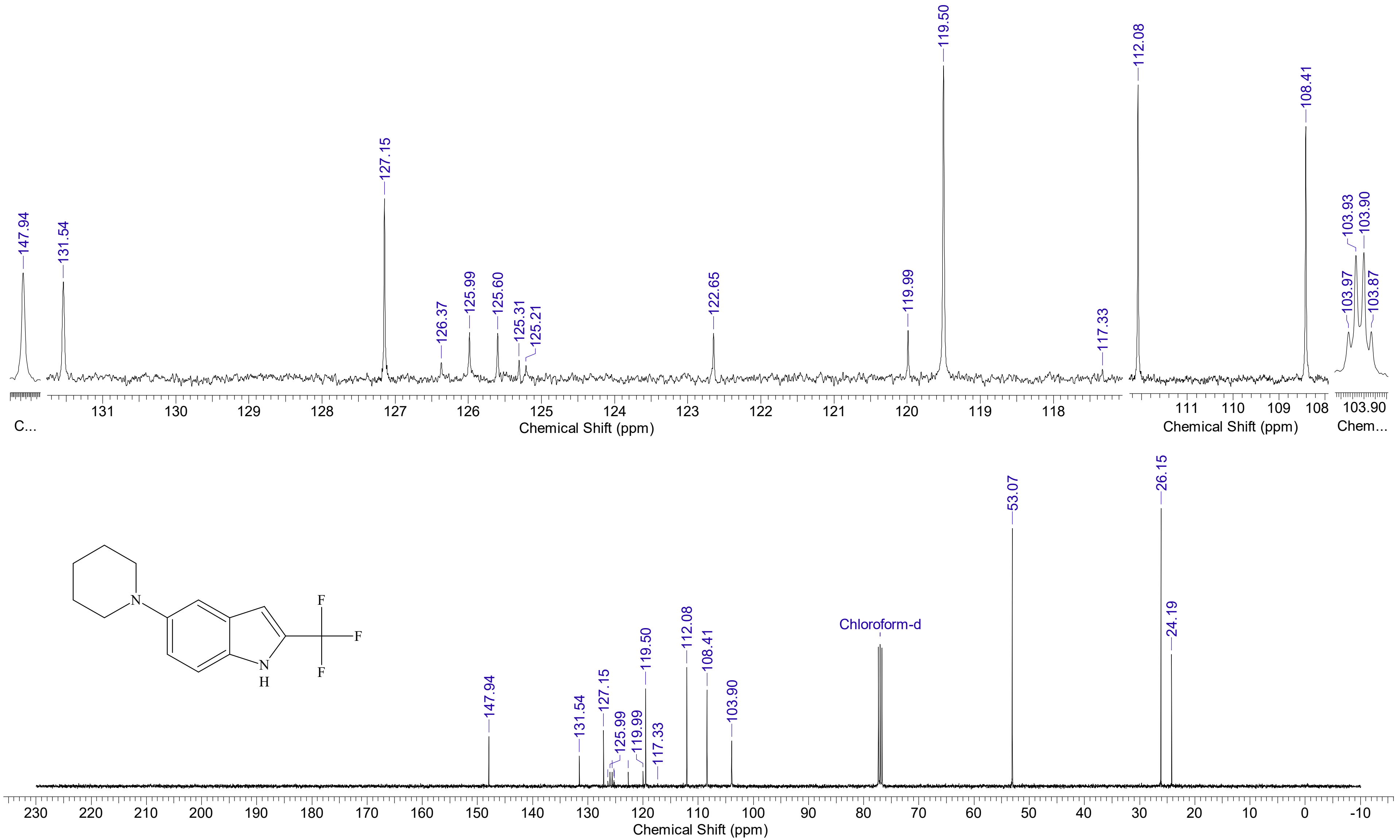

$^{13}C\{^{1}H\}$ NMR spectrum of **10b** (100.6 MHz, $CDCl_3$)

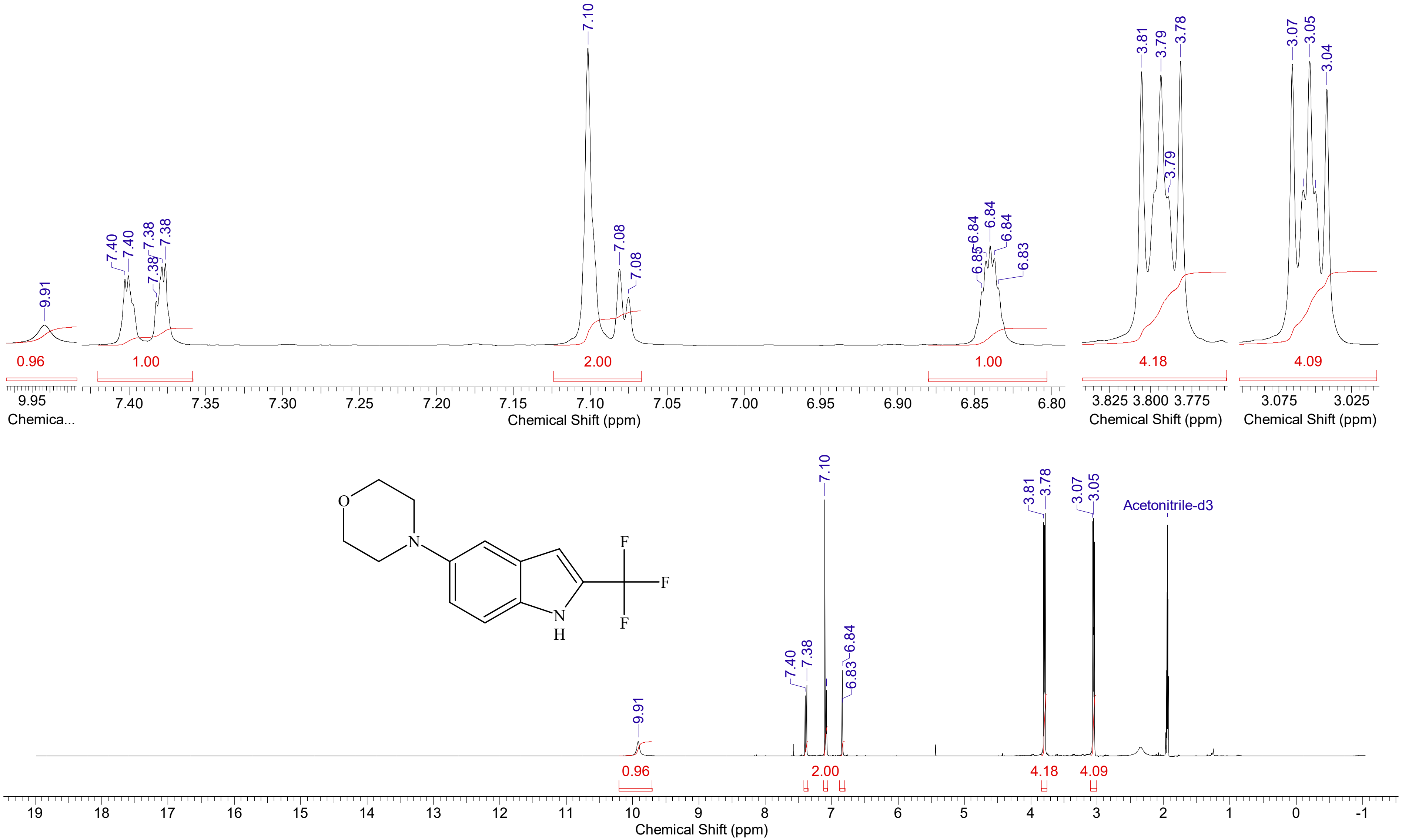

^{1}H NMR spectrum of **10c** (400.1 MHz, $CDCl_3$)

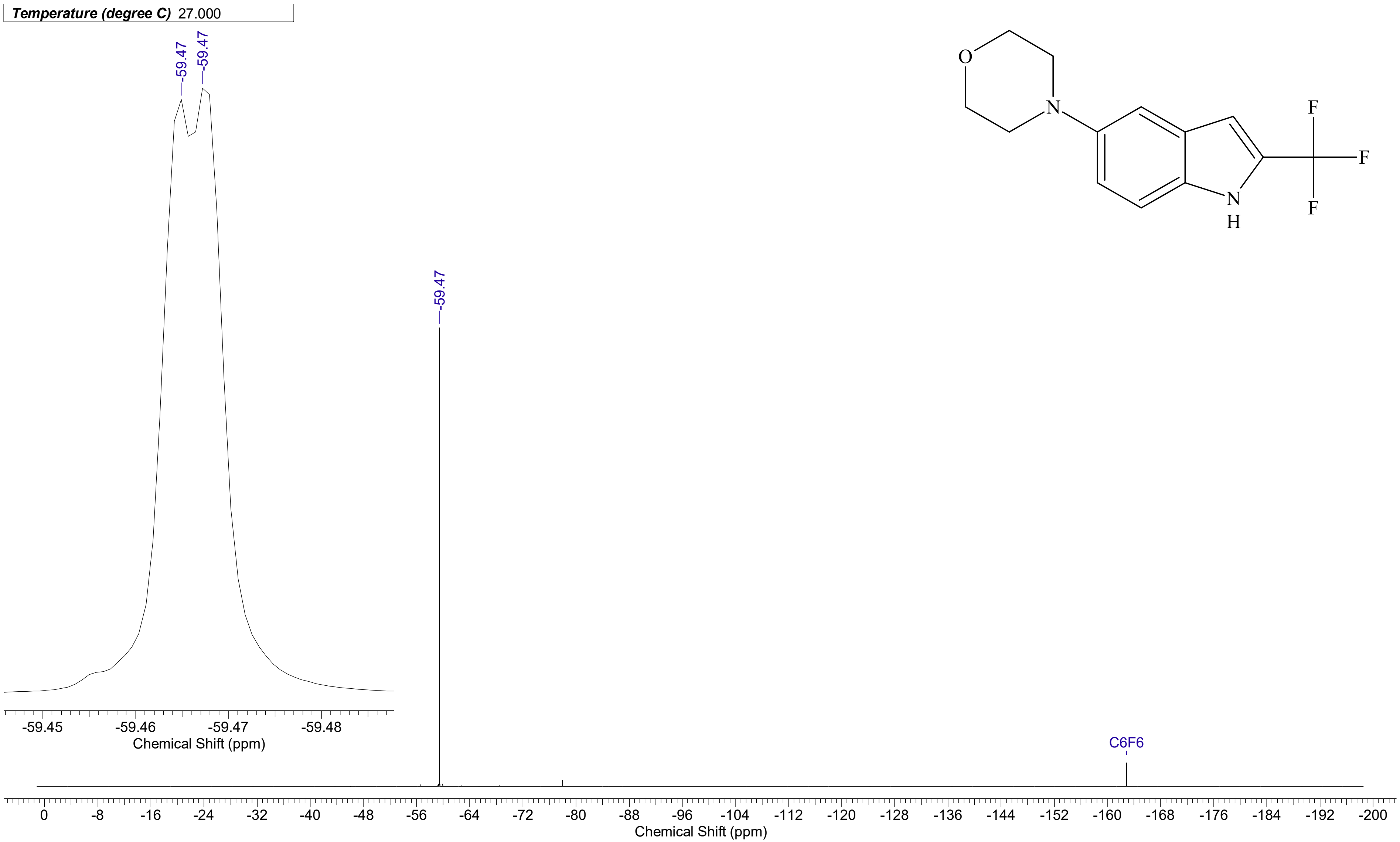

^{19}F NMR spectrum of **10c** (376.5 MHz, $CDCl_3$)

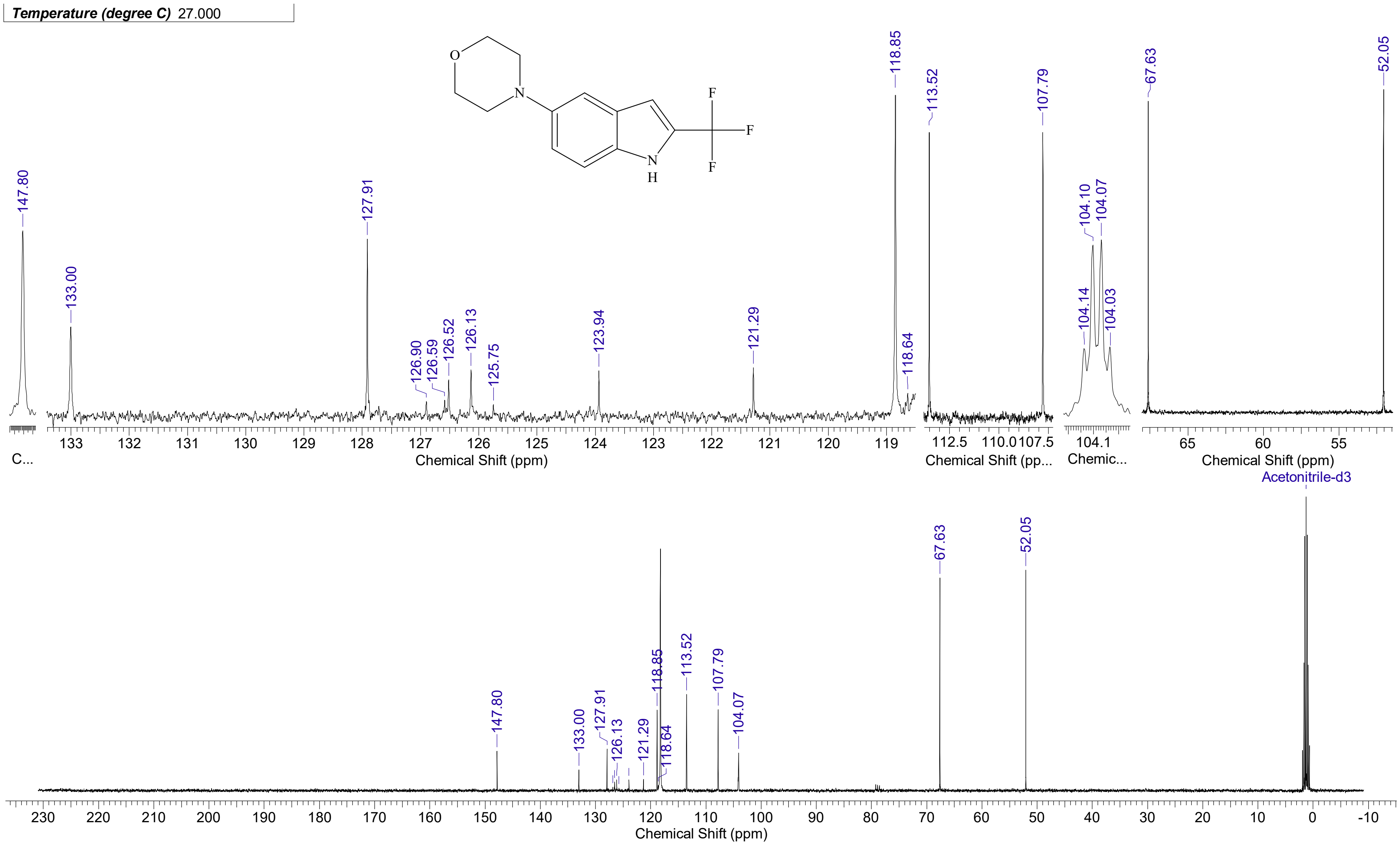

$^{13}C\{^{1}H\}$ NMR spectrum of **10c** (100.6 MHz, $CDCl_3$)

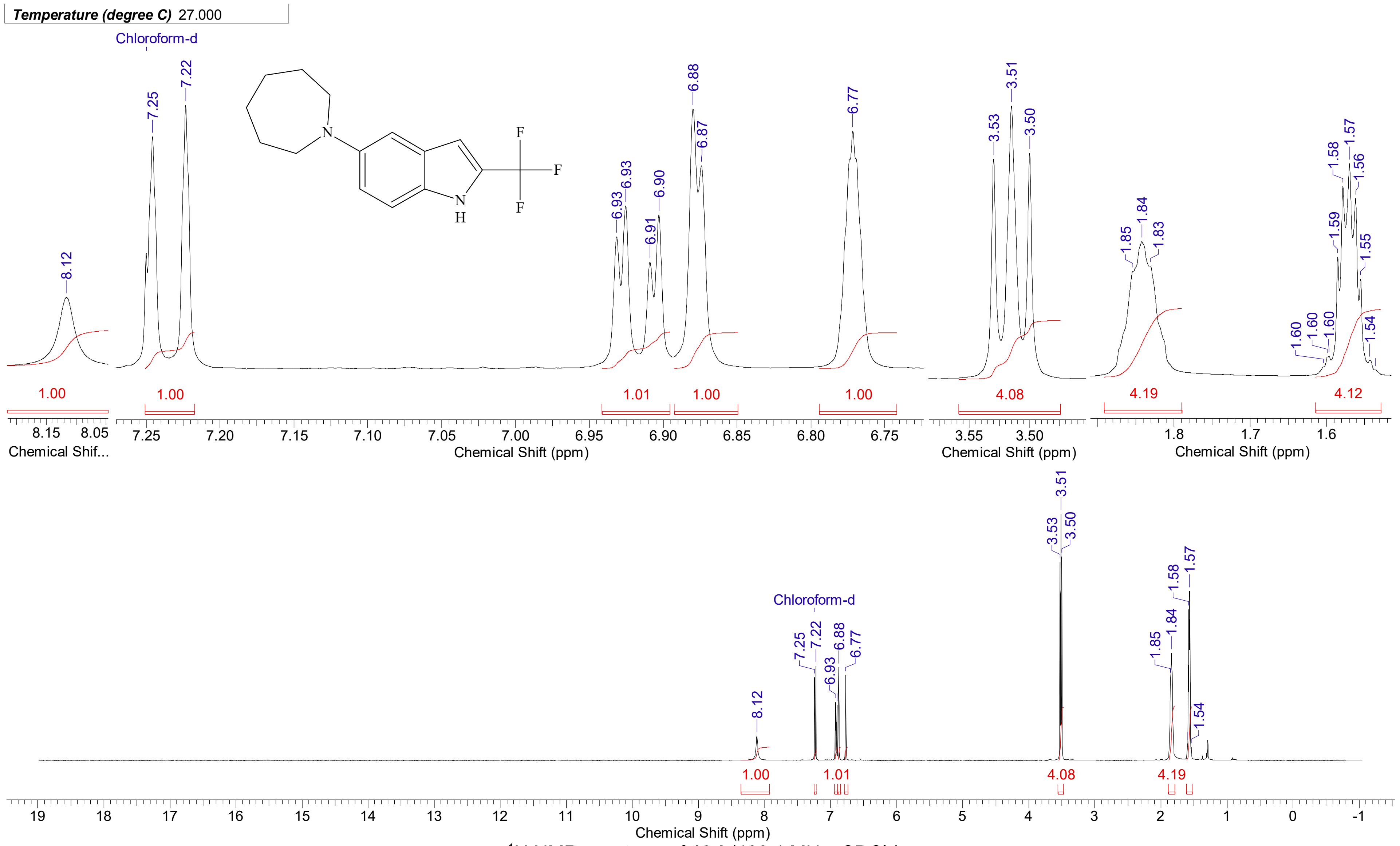

^{1}H NMR spectrum of **10d** (400.1 MHz, $CDCl_3$)

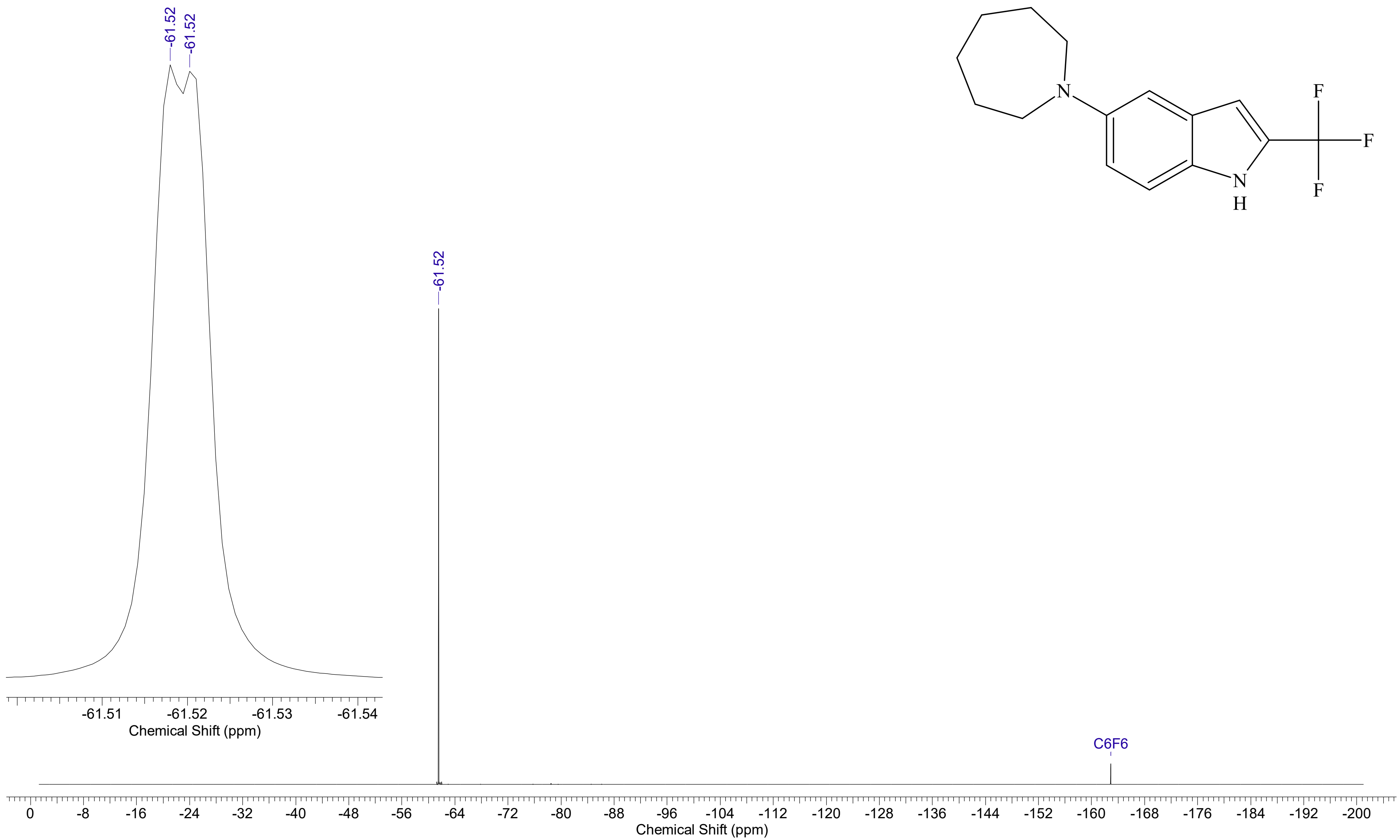

^{19}F NMR spectrum of **10d** (376.5 MHz, $CDCl_3$)

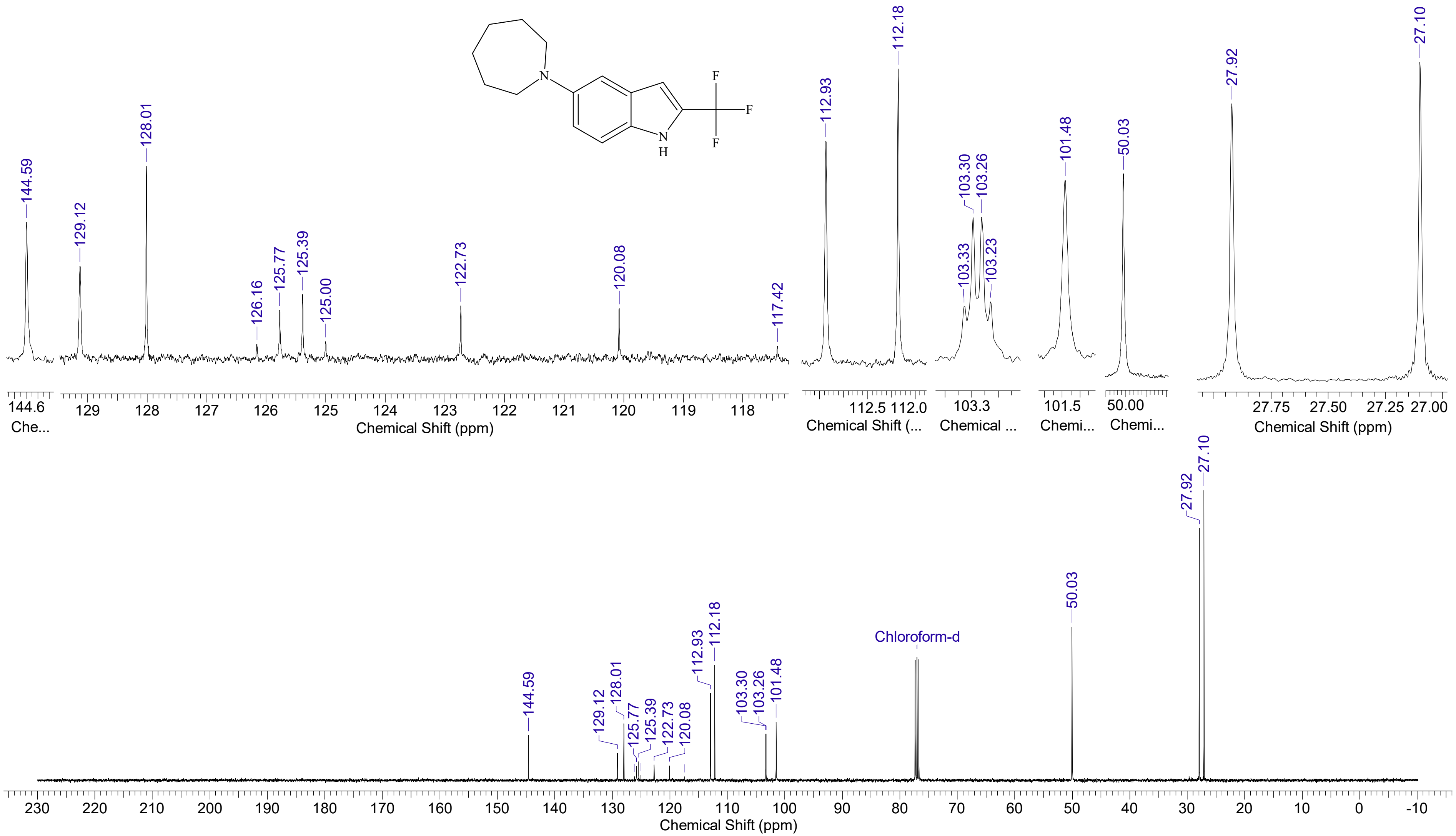

$^{13}C\{^{1}H\}$ NMR spectrum of **10d** (100.6 MHz, $CDCl_3$)

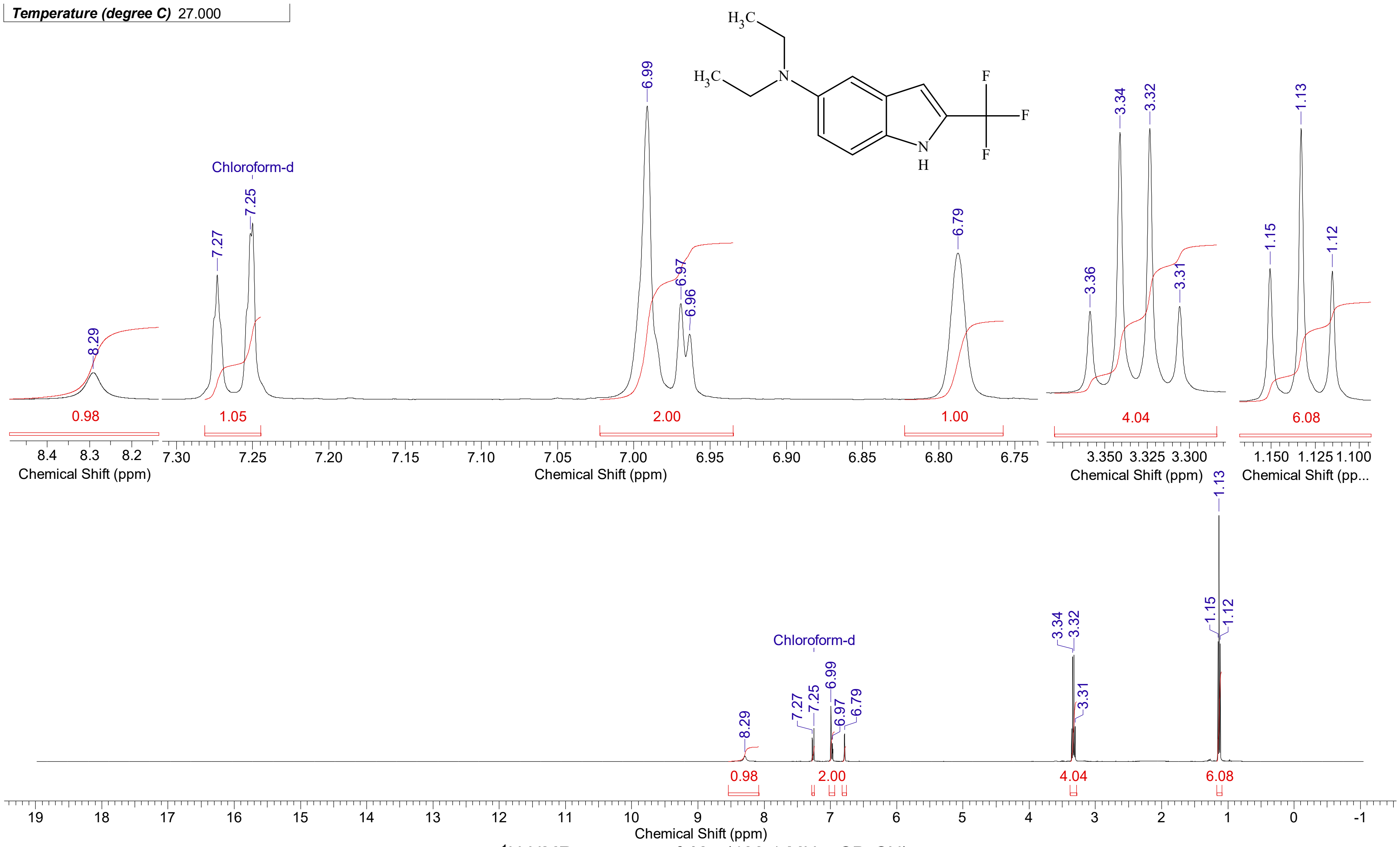

^{1}H NMR spectrum of **10e** (400.1 MHz, CD_3CN)

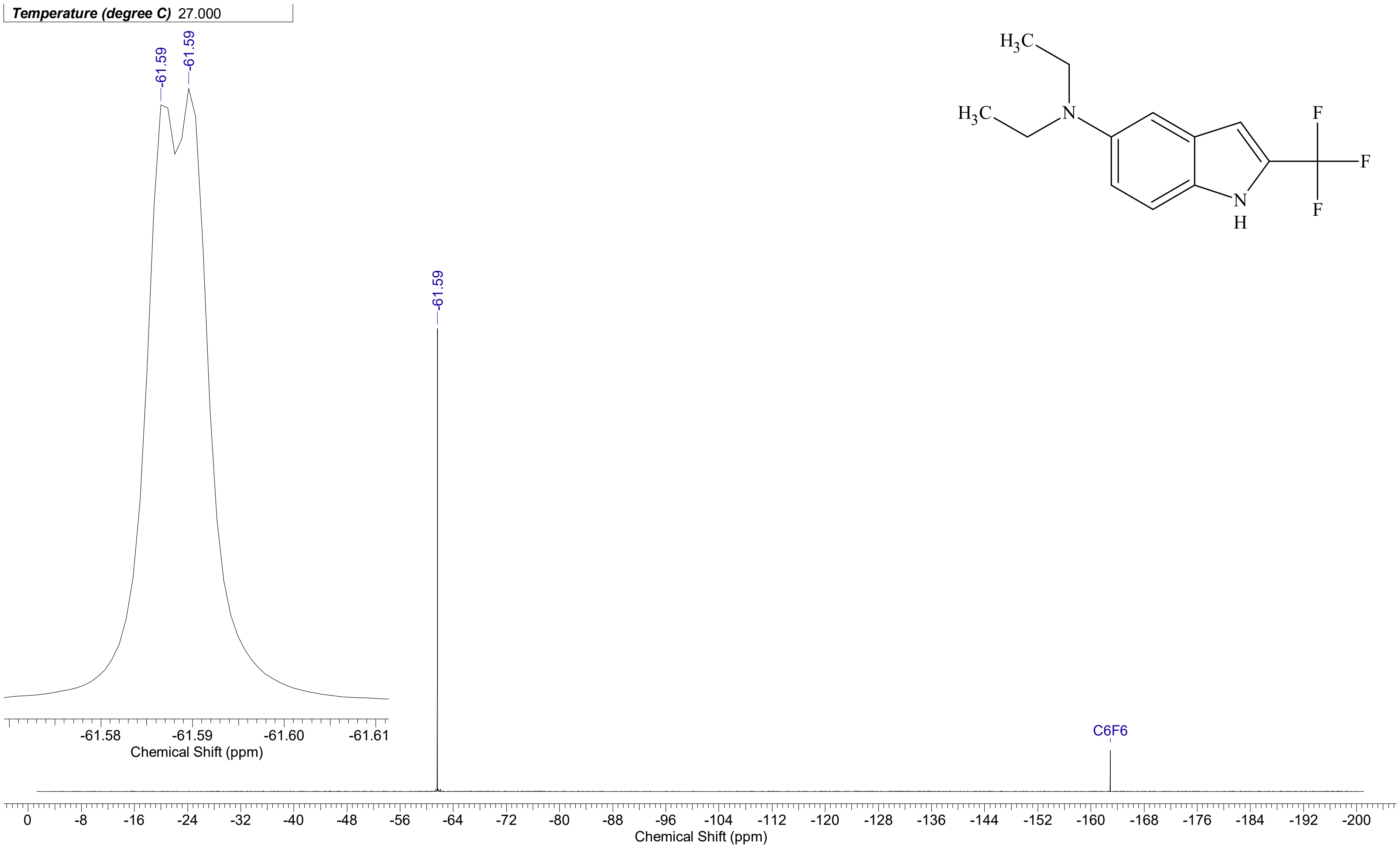

^{19}F NMR spectrum of **10e** (376.5 MHz, CD_3CN)

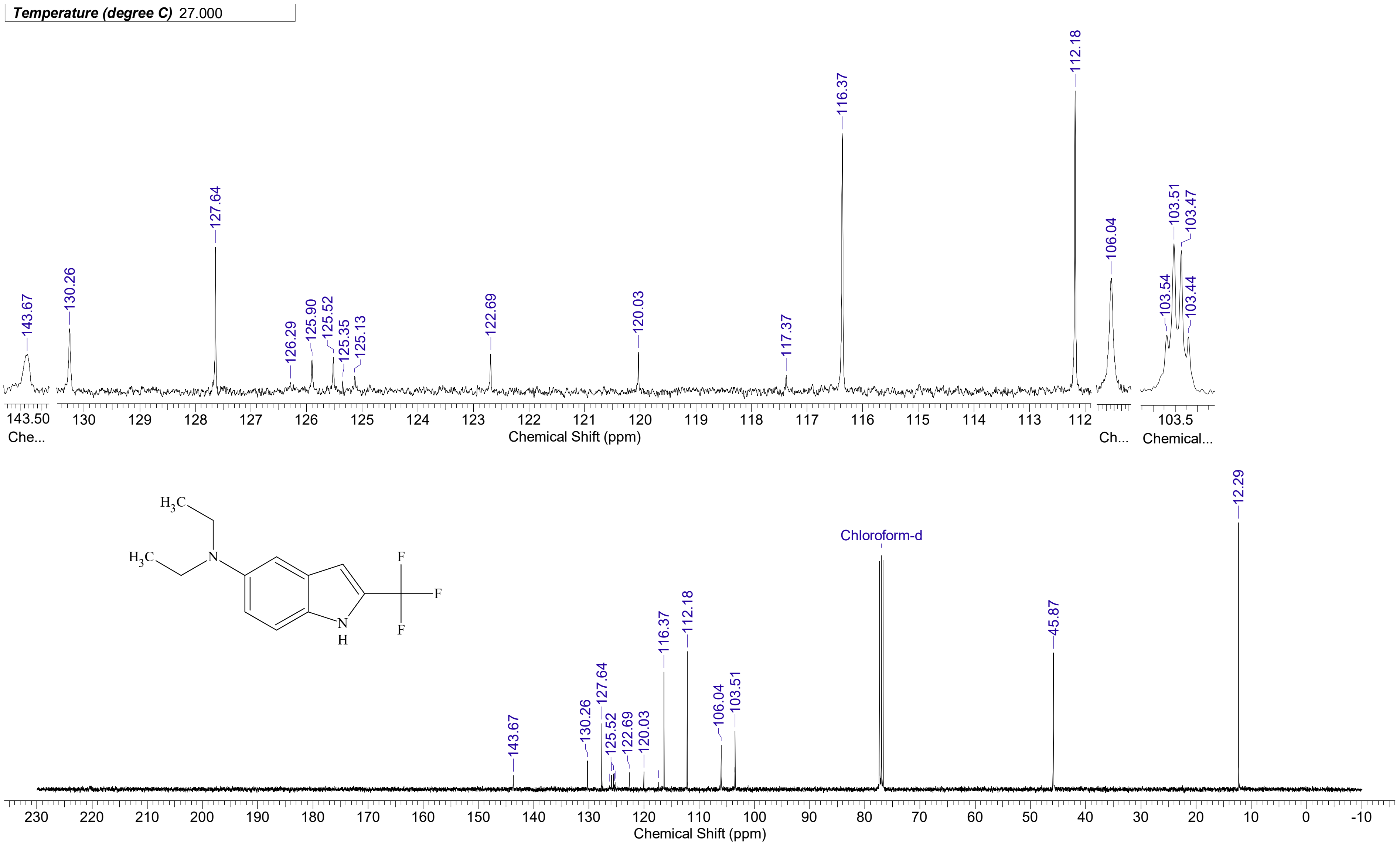

$^{13}C\{^{1}H\}$ NMR spectrum of **10e** (100.6 MHz, CD_3CN)

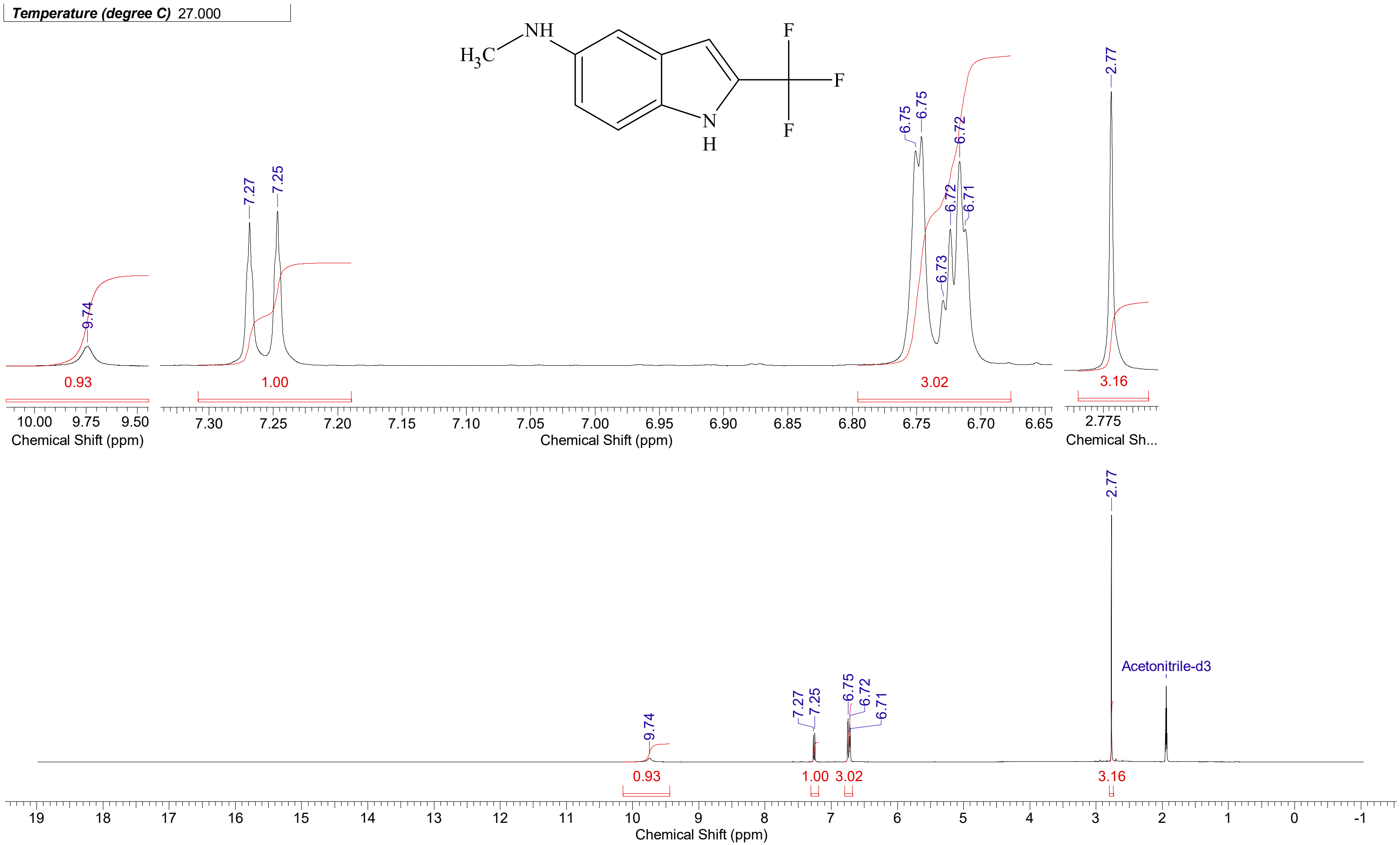

^{1}H NMR spectrum of **10f** (400.1 MHz, $CDCl_3$)

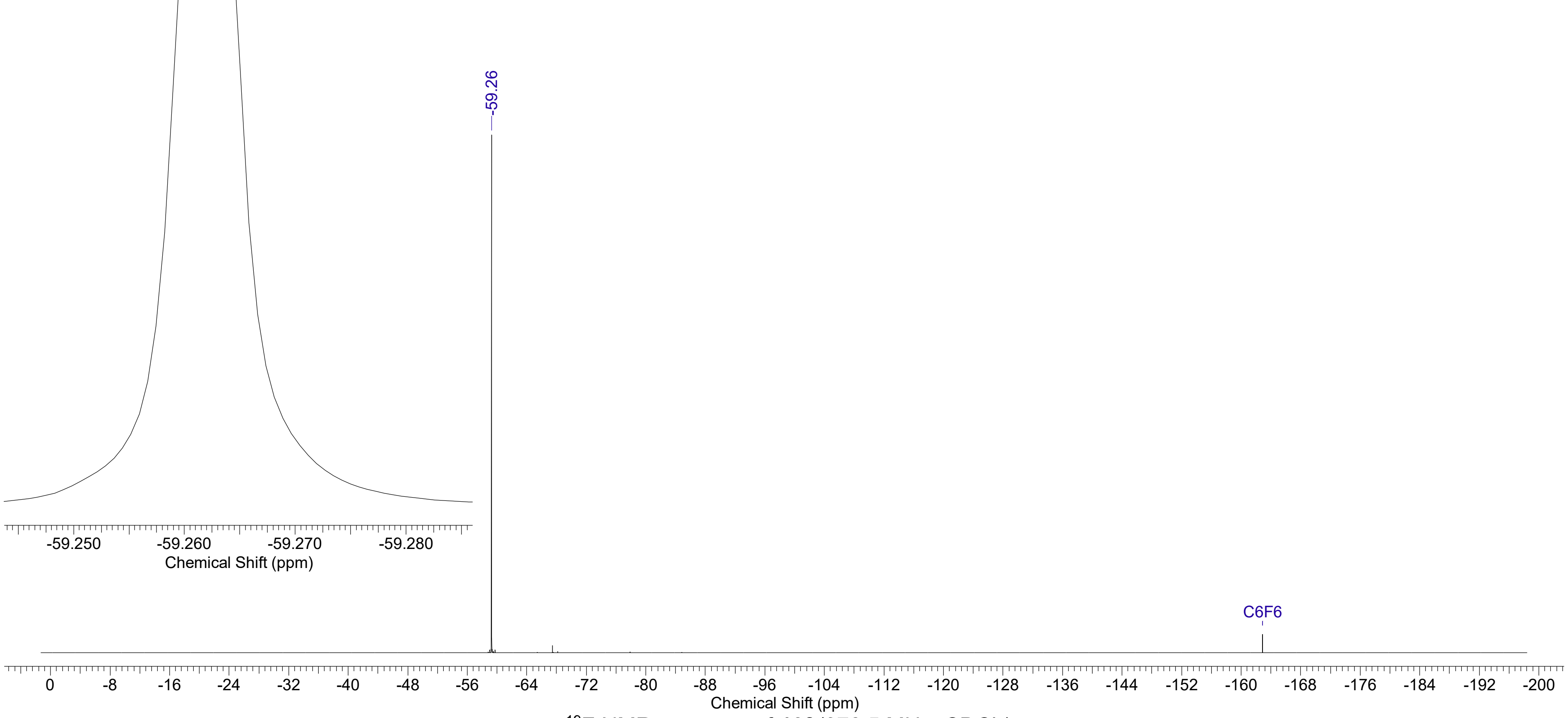

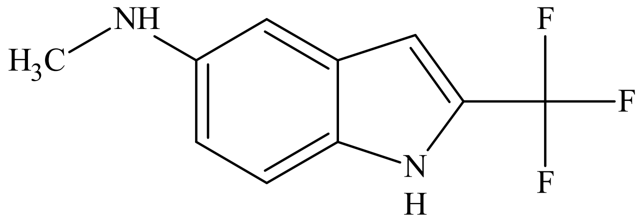

^{19}F NMR spectrum of **10f** (376.5 MHz, $CDCl_3$)

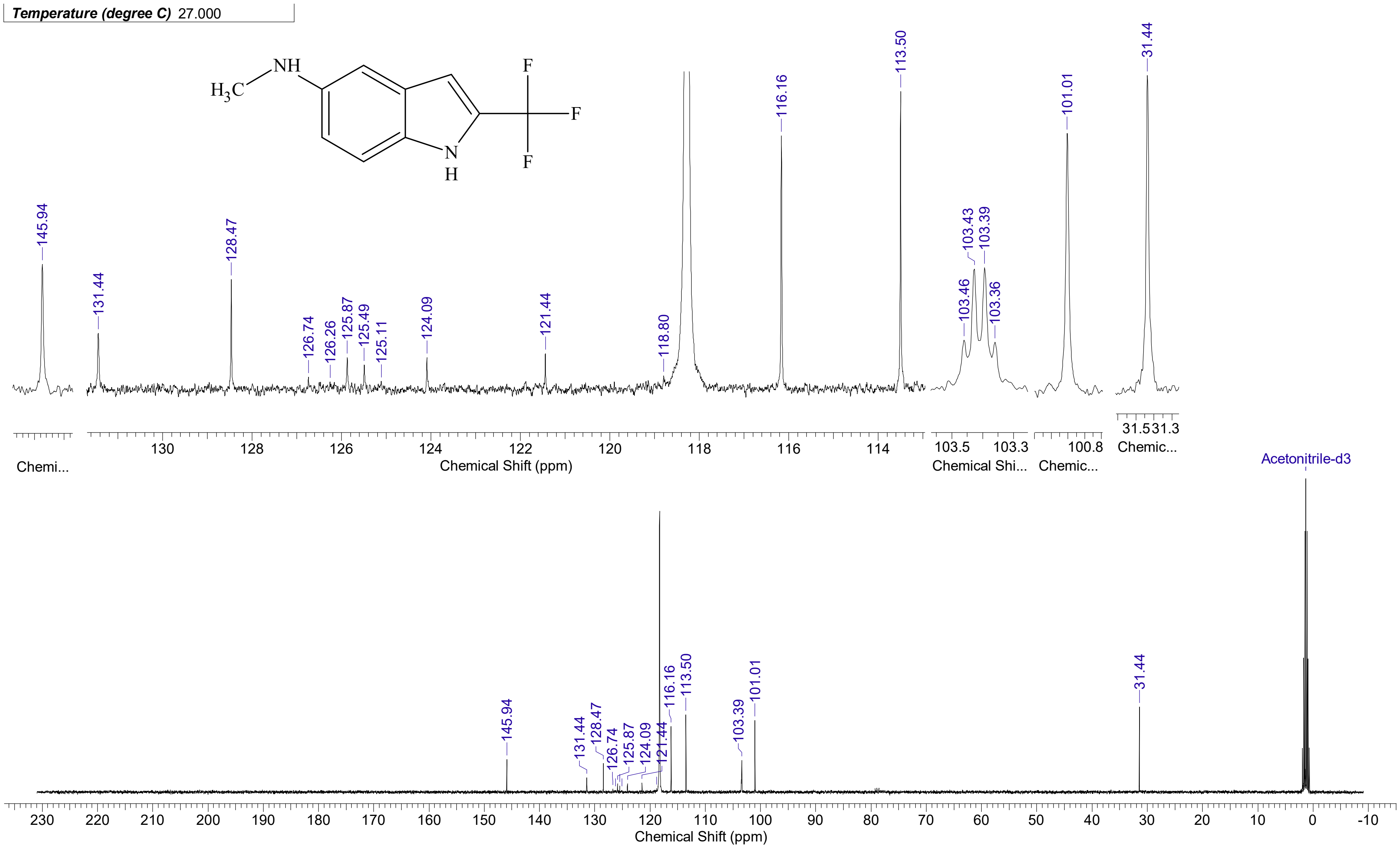

$^{13}C\{^1H\}$ NMR spectrum of **10f** (100.6 MHz, $CDCl_3$)

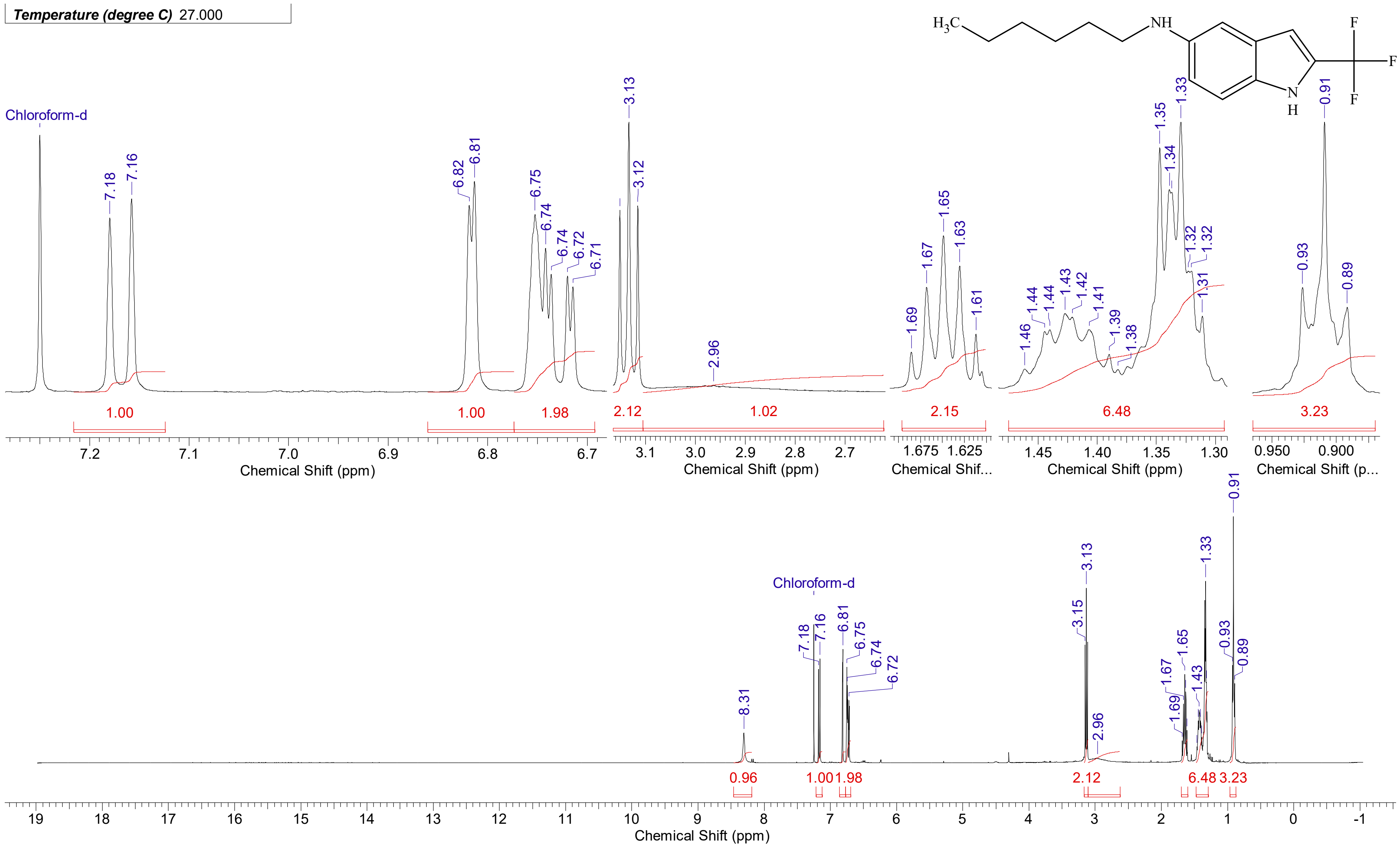

^{1}H NMR spectrum of **10g** (400.1 MHz, $CDCl_3$)

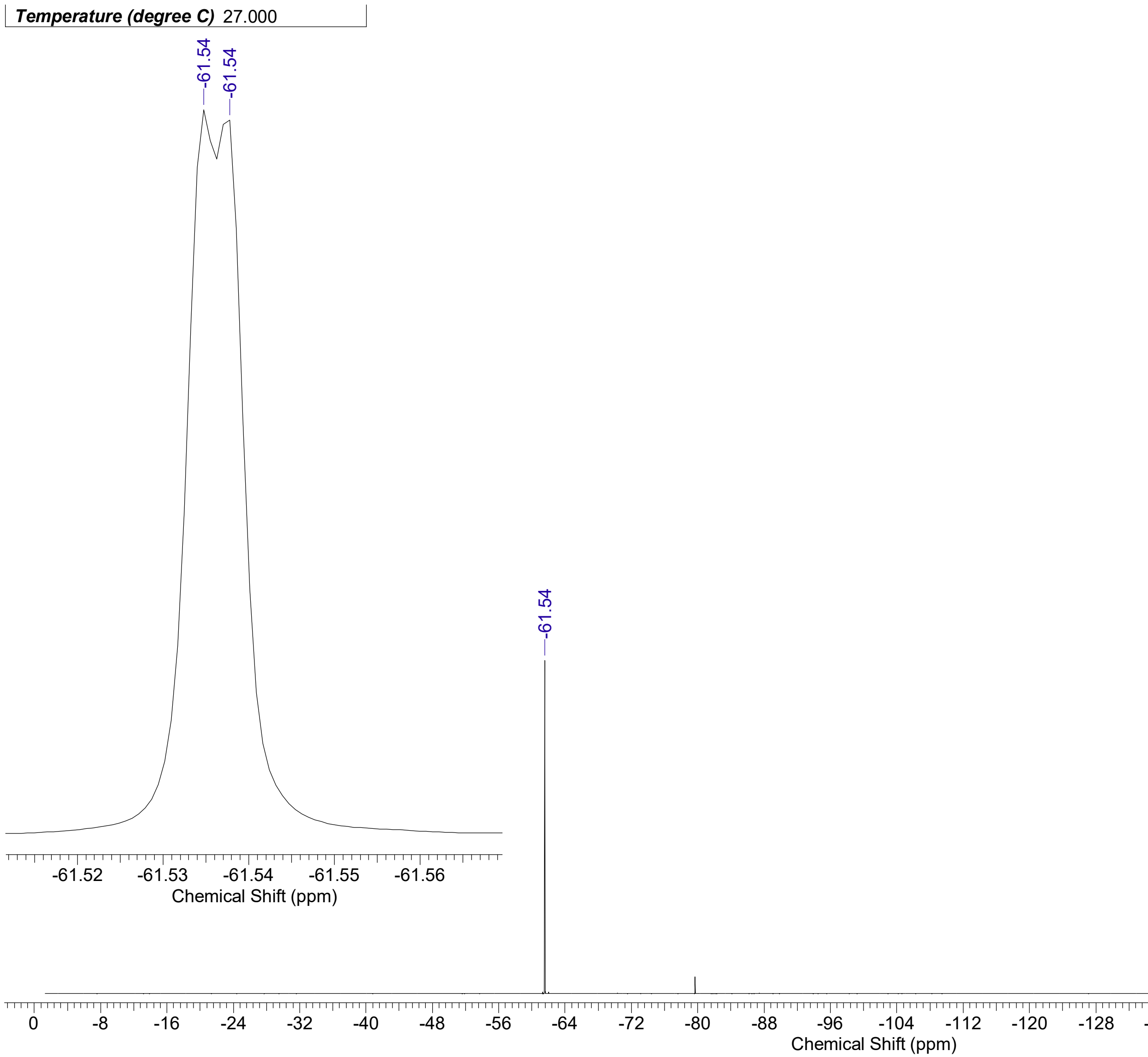

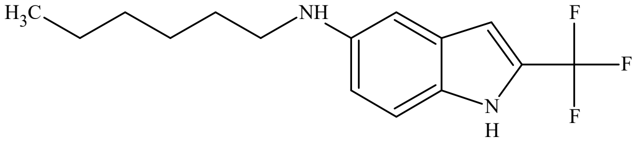

^{19}F NMR spectrum of **10g** (376.5 MHz, $CDCl_3$)

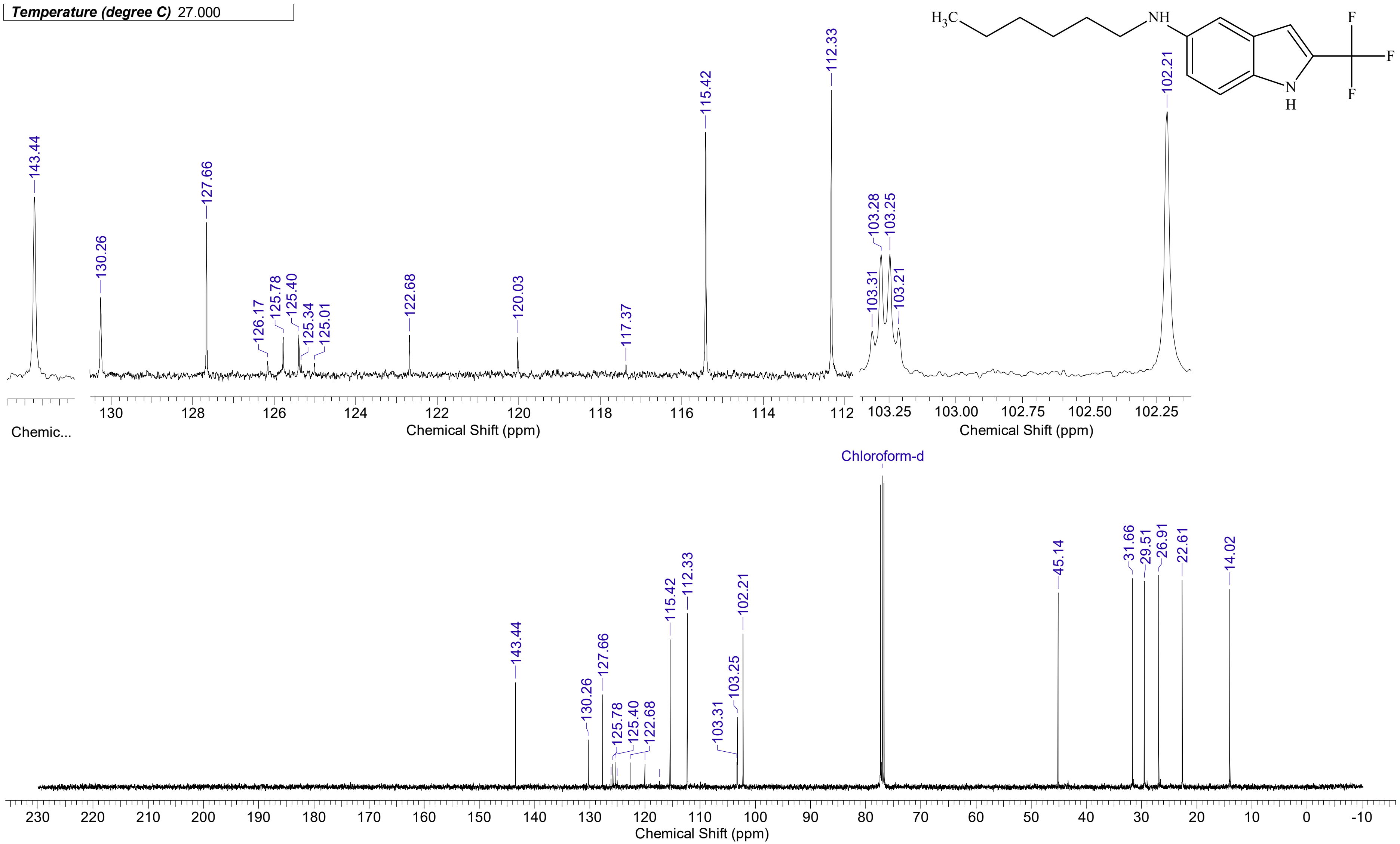

$^{13}C\{^1H\}$ NMR spectrum of **10g** (100.6 MHz, $CDCl_3$)

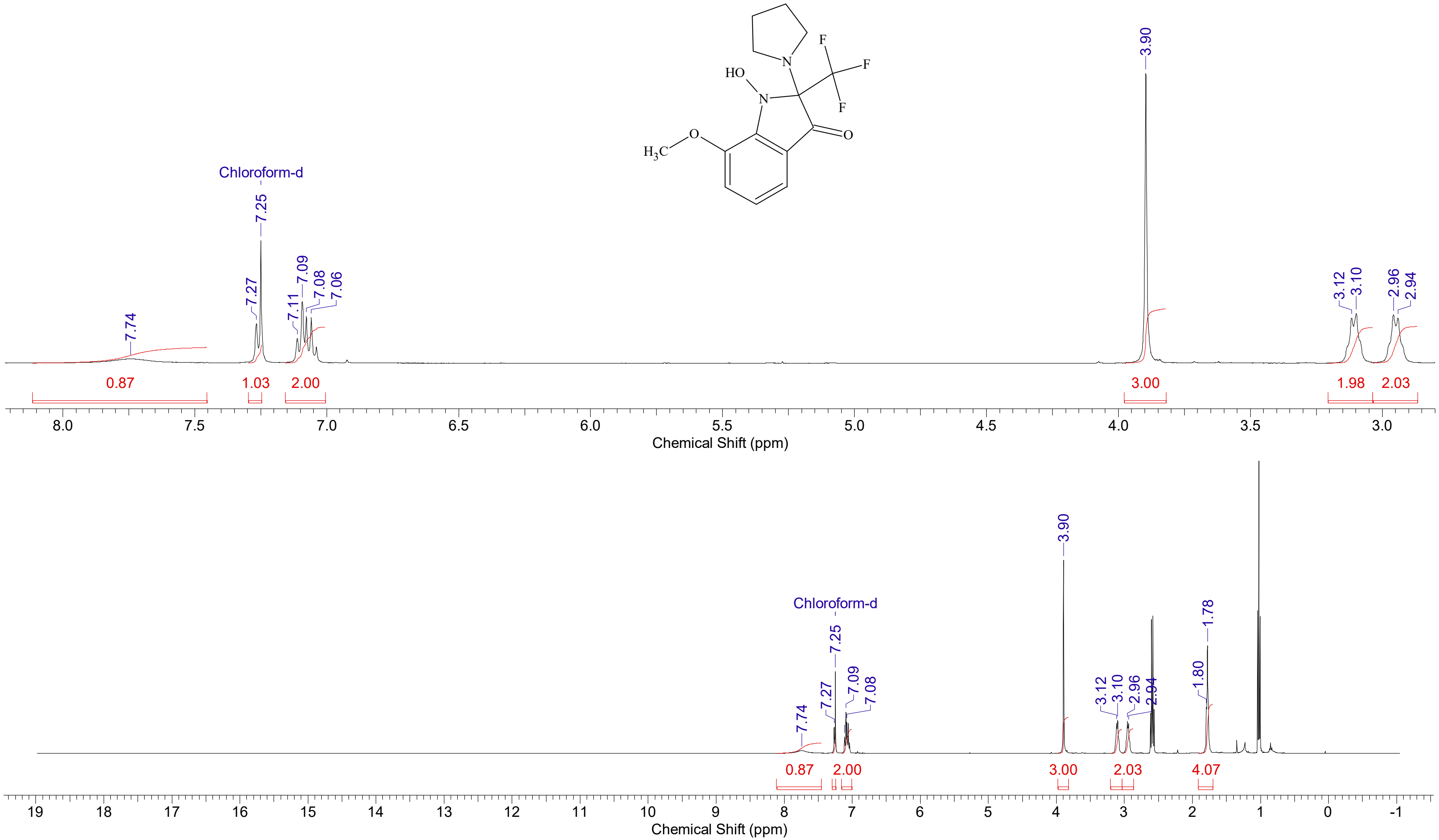

^{1}H NMR spectrum of **5c** (400.1 MHz, $CDCl_3$)

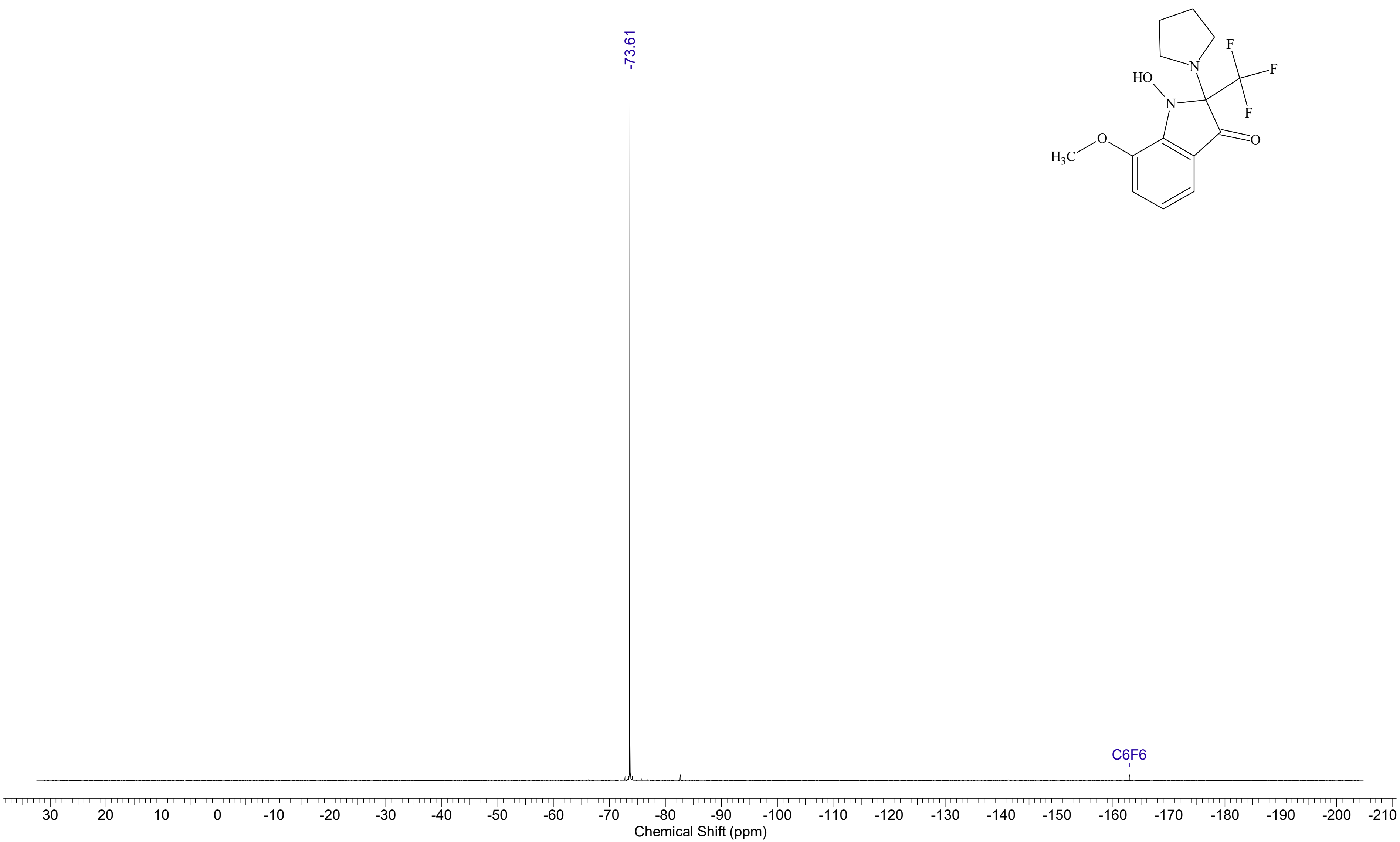

^{19}F NMR spectrum of **5c** (376.5 MHz $CDCl_3$)

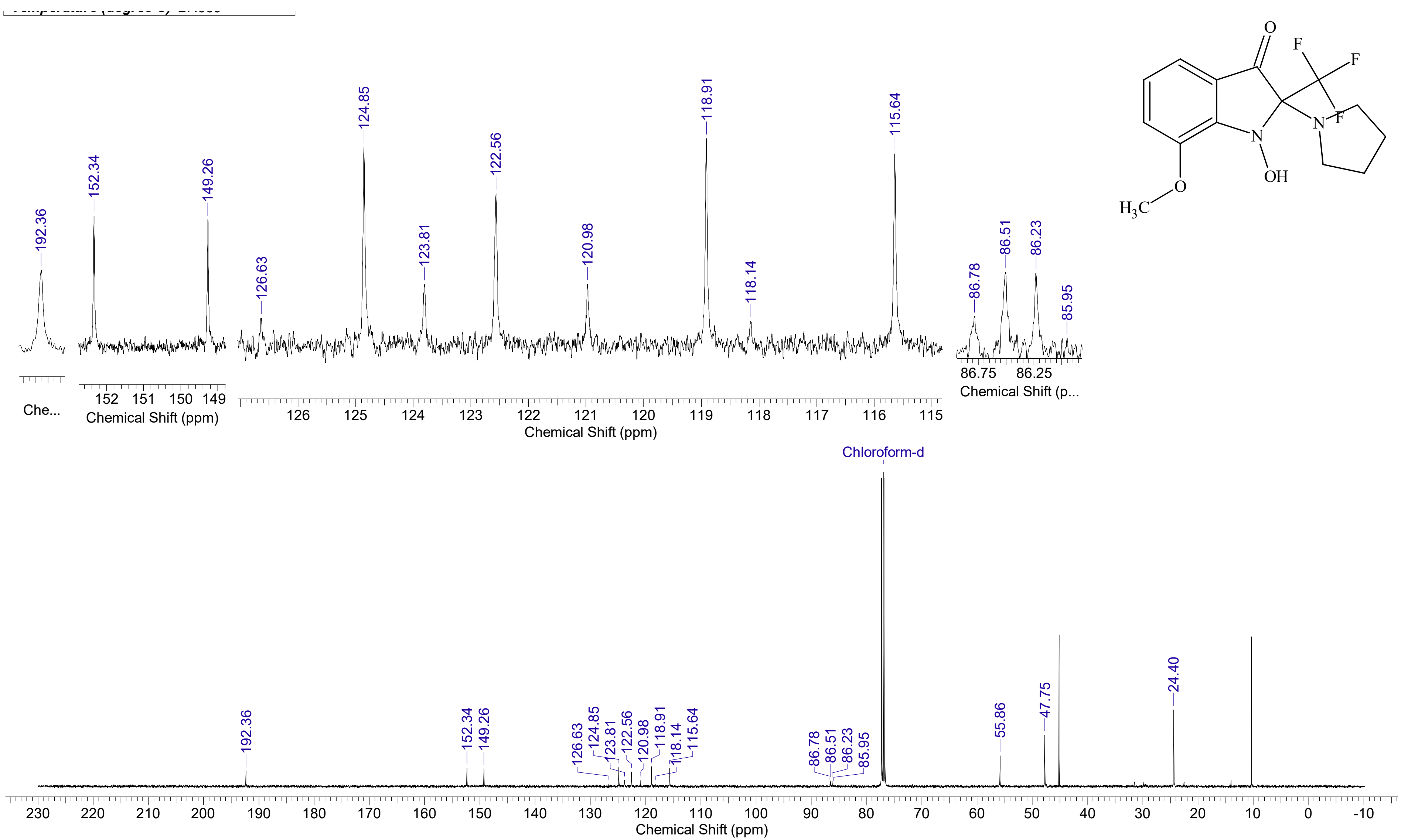

$^{13}C\{^{1}H\}$ NMR spectrum of **5c** (100.6 MHz, $CDCl_3$)

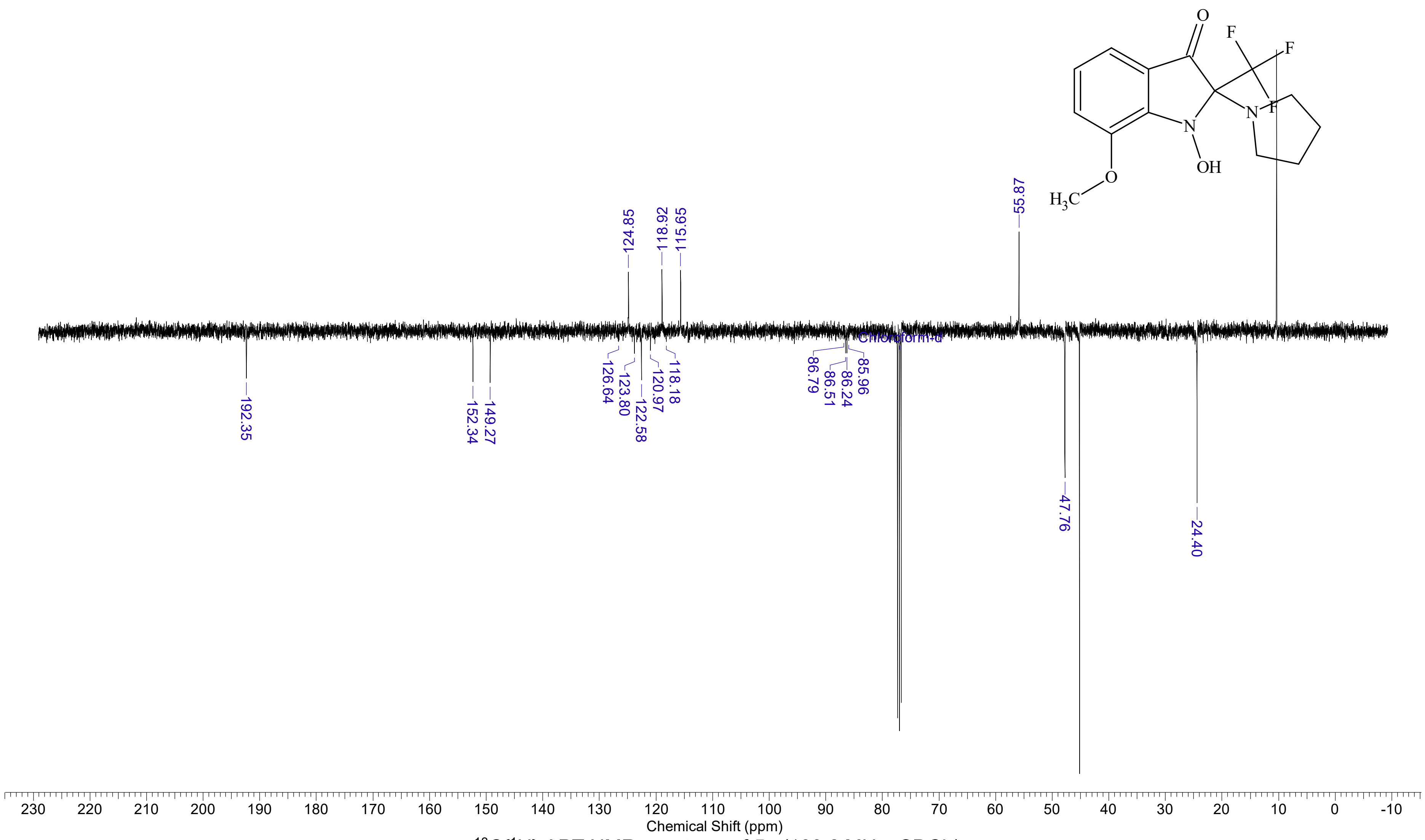

$^{13}C\{^1H\}$ APT NMR spectrum of **5c** (100.6 MHz, $CDCl_3$)

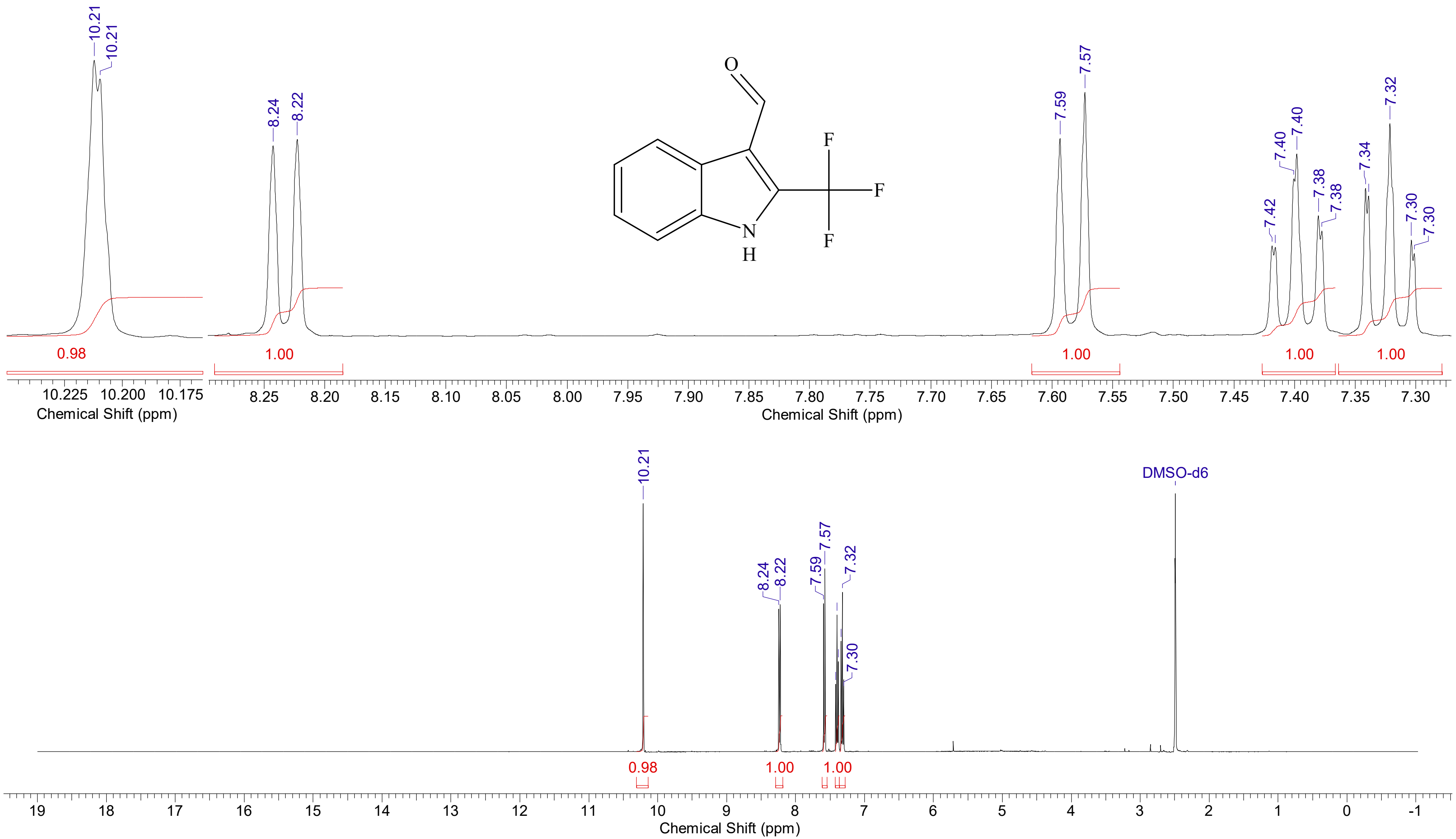

^{1}H NMR spectrum of **17** (400.1 MHz, DMSO-d_6)

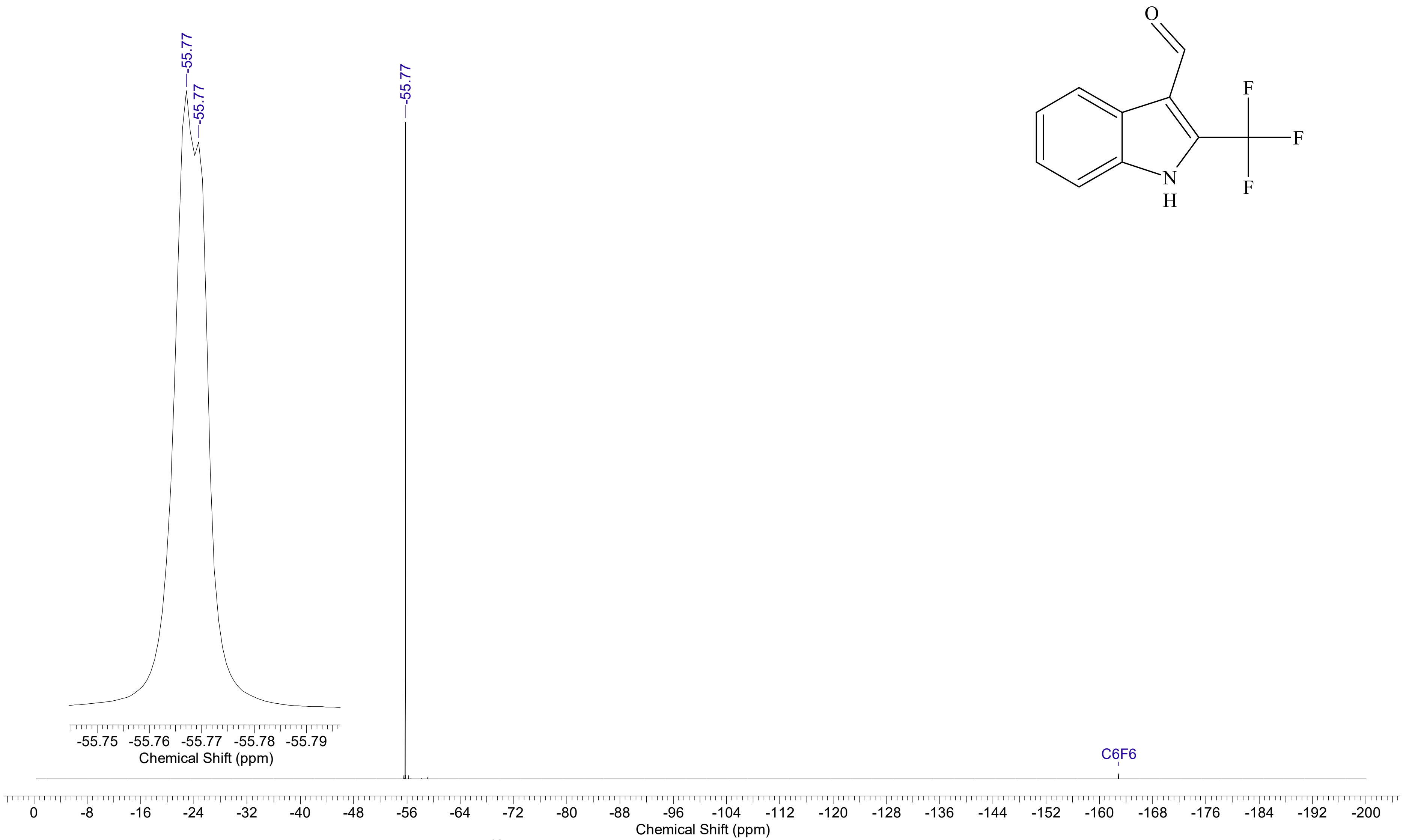

^{19}F NMR spectrum of **17** (376.5 MHz, DMSO-d_6)

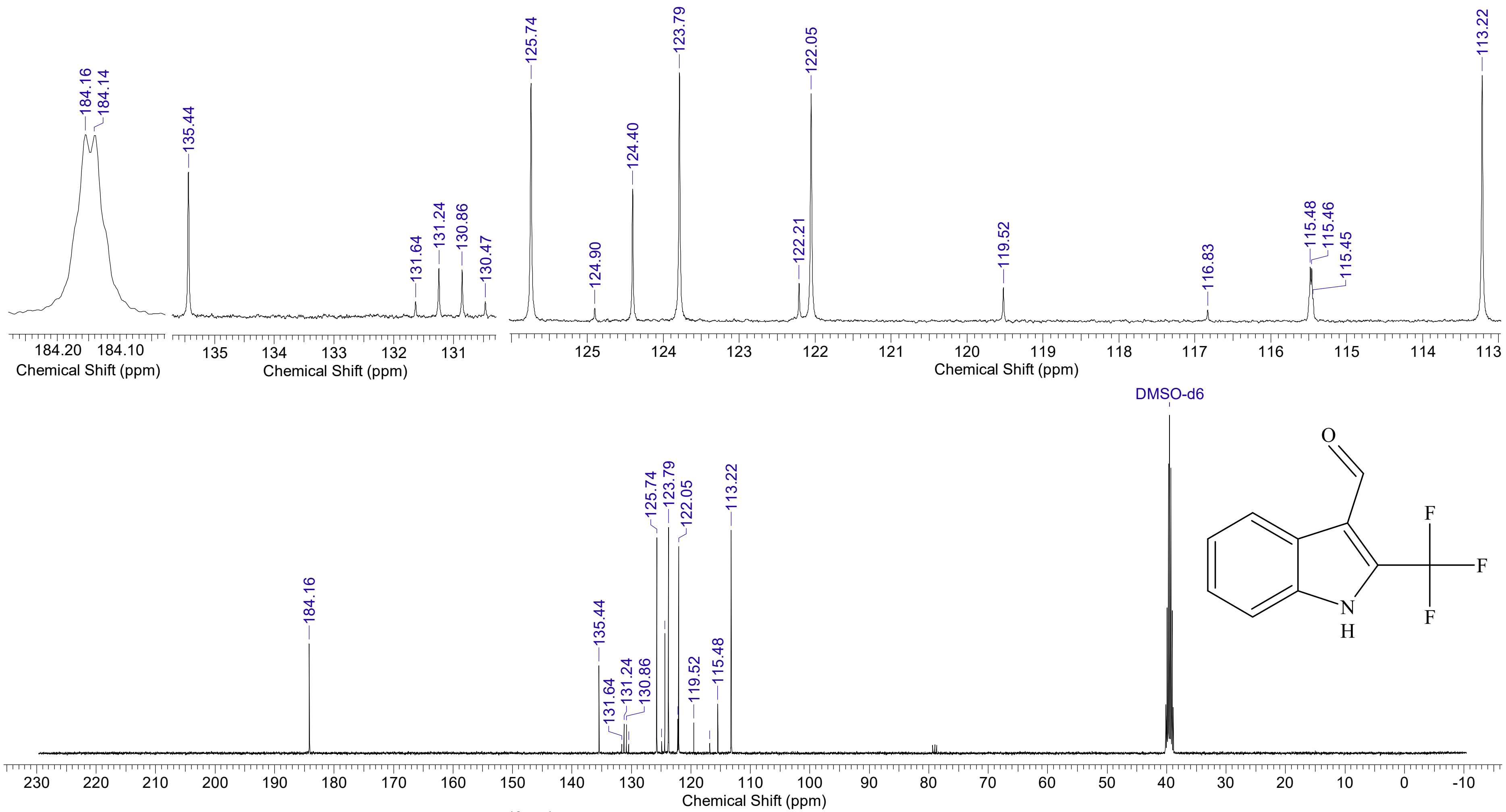

$^{13}C\{^{1}H\}$ NMR spectrum of **17** (100.6 MHz, DMSO-d_6)

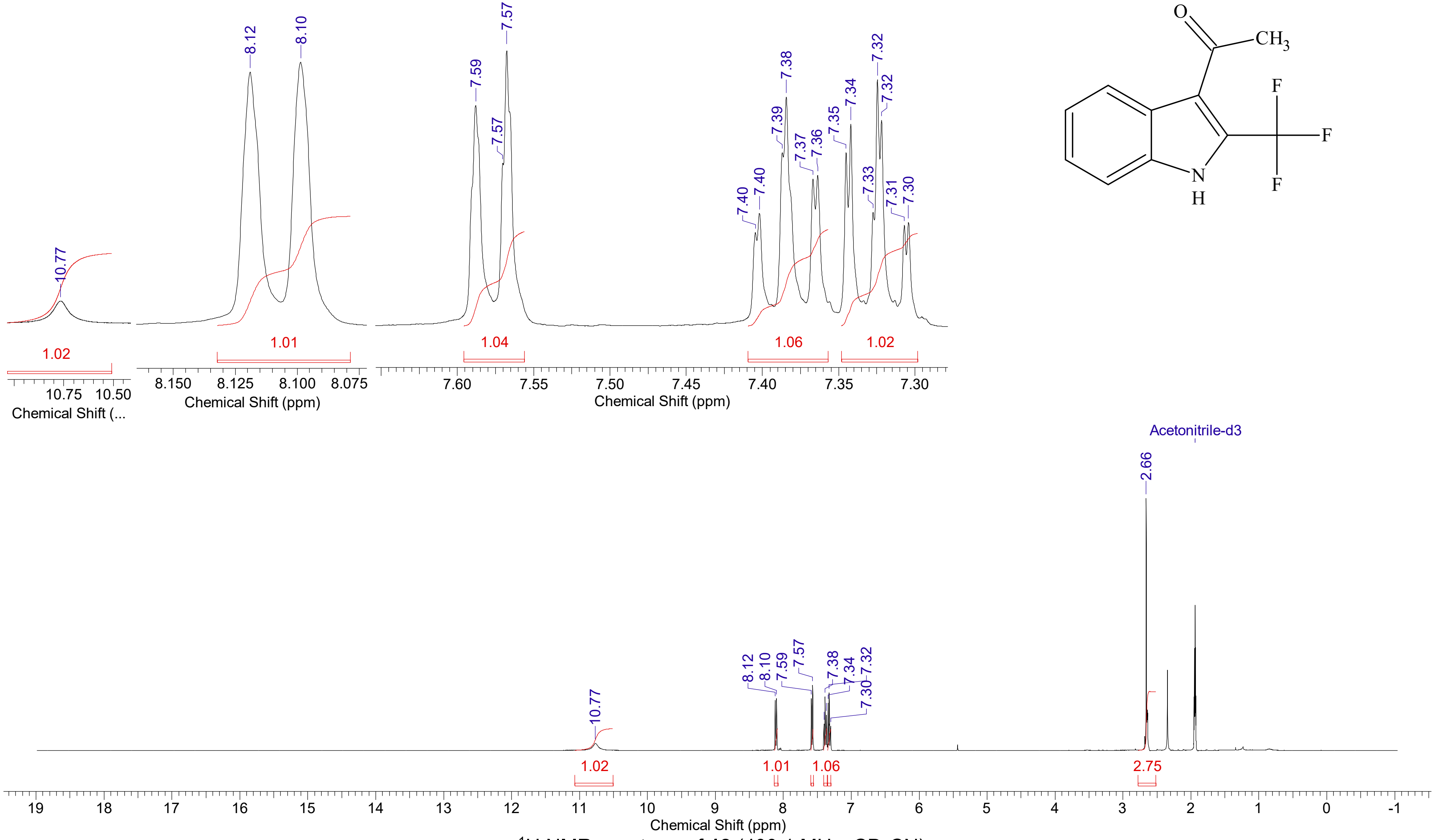

^{1}H NMR spectrum of **18** (400.1 MHz, CD_3CN)

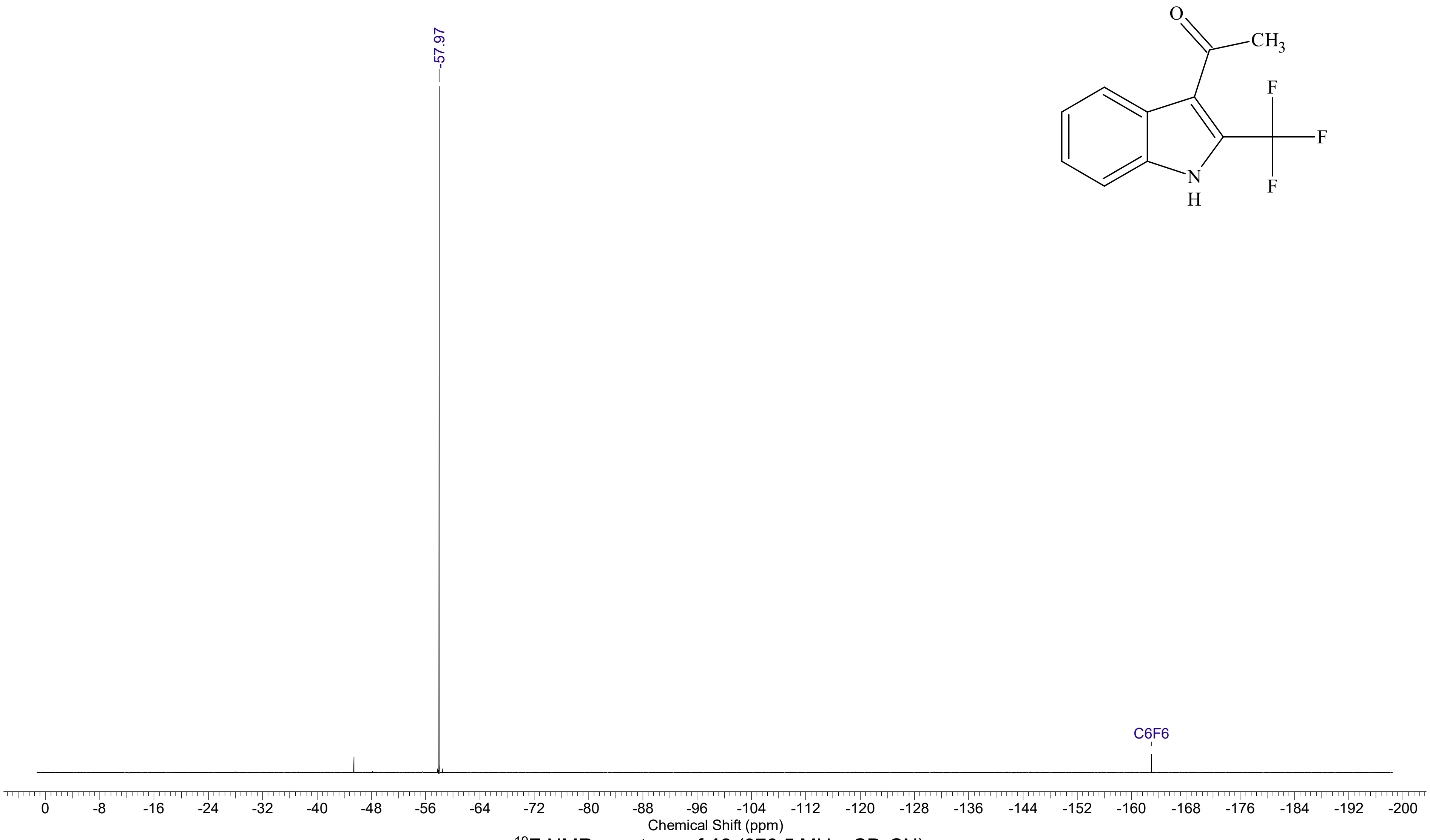

^{19}F NMR spectrum of **18** (376.5 MHz, CD_3CN)

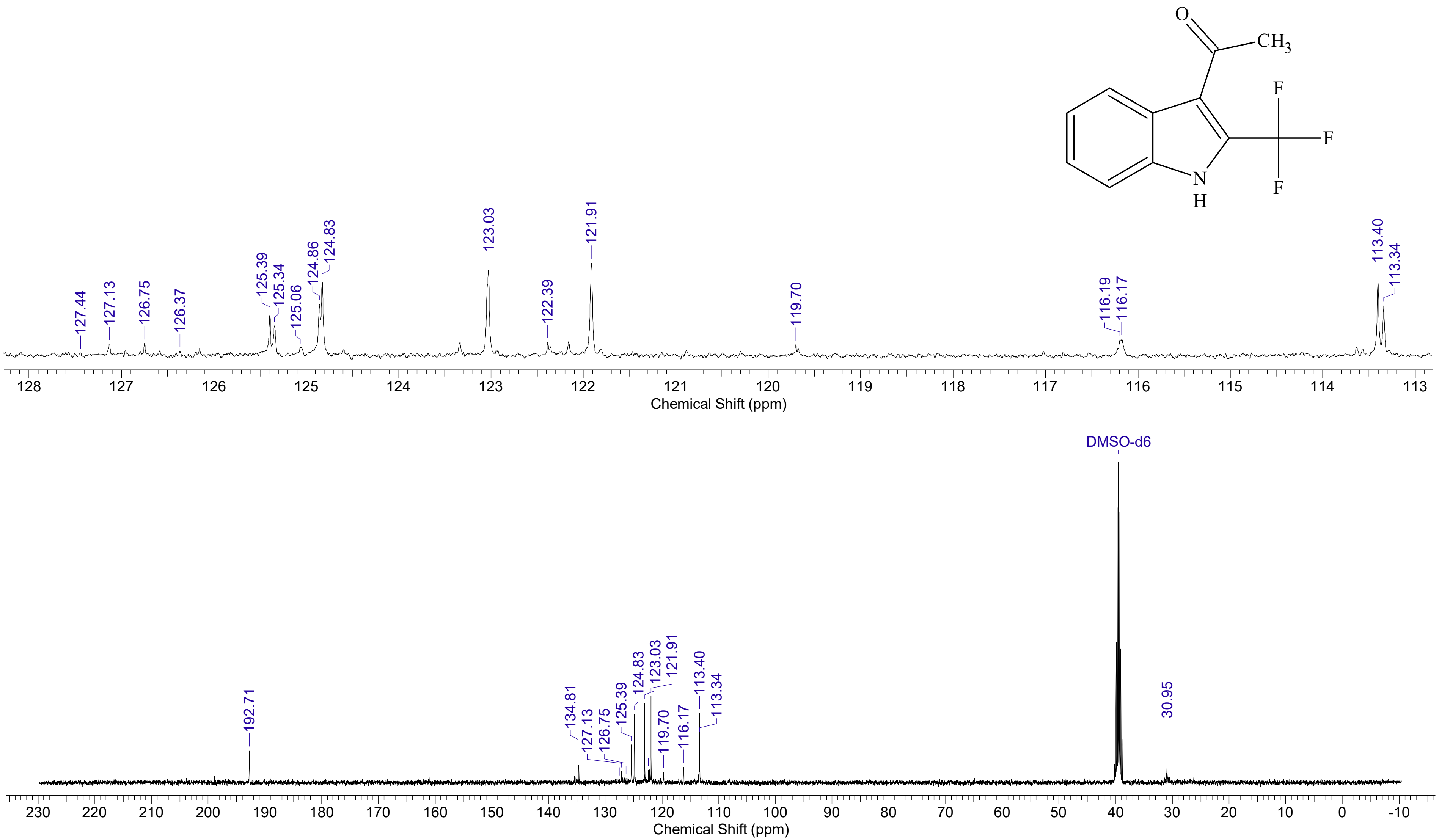

$^{13}C\{^1H\}$ NMR spectrum of **18** (100.6 MHz, CD_3CN)

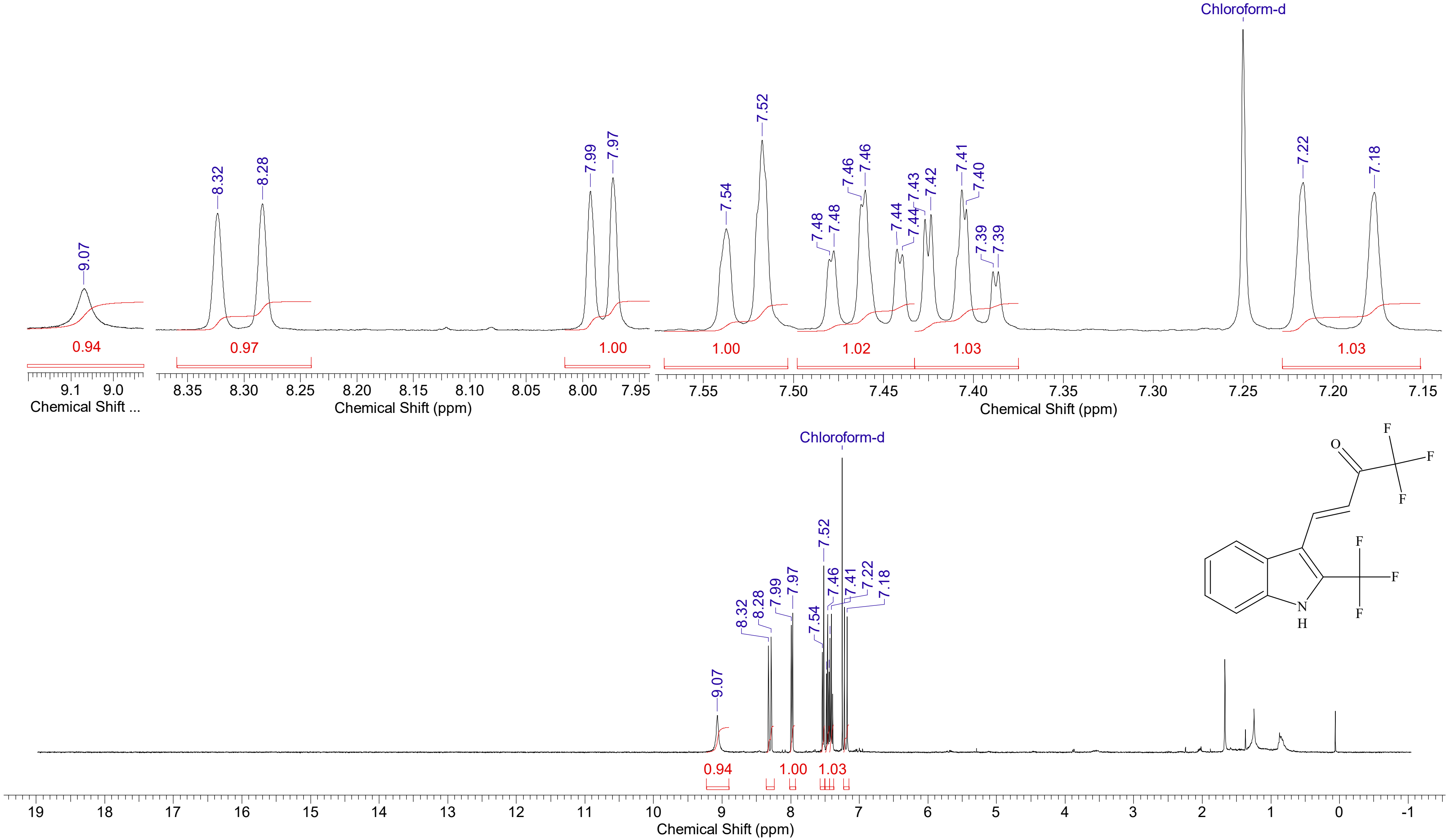

^{1}H NMR spectrum of **20** (400.1 MHz, $CDCl_3$)

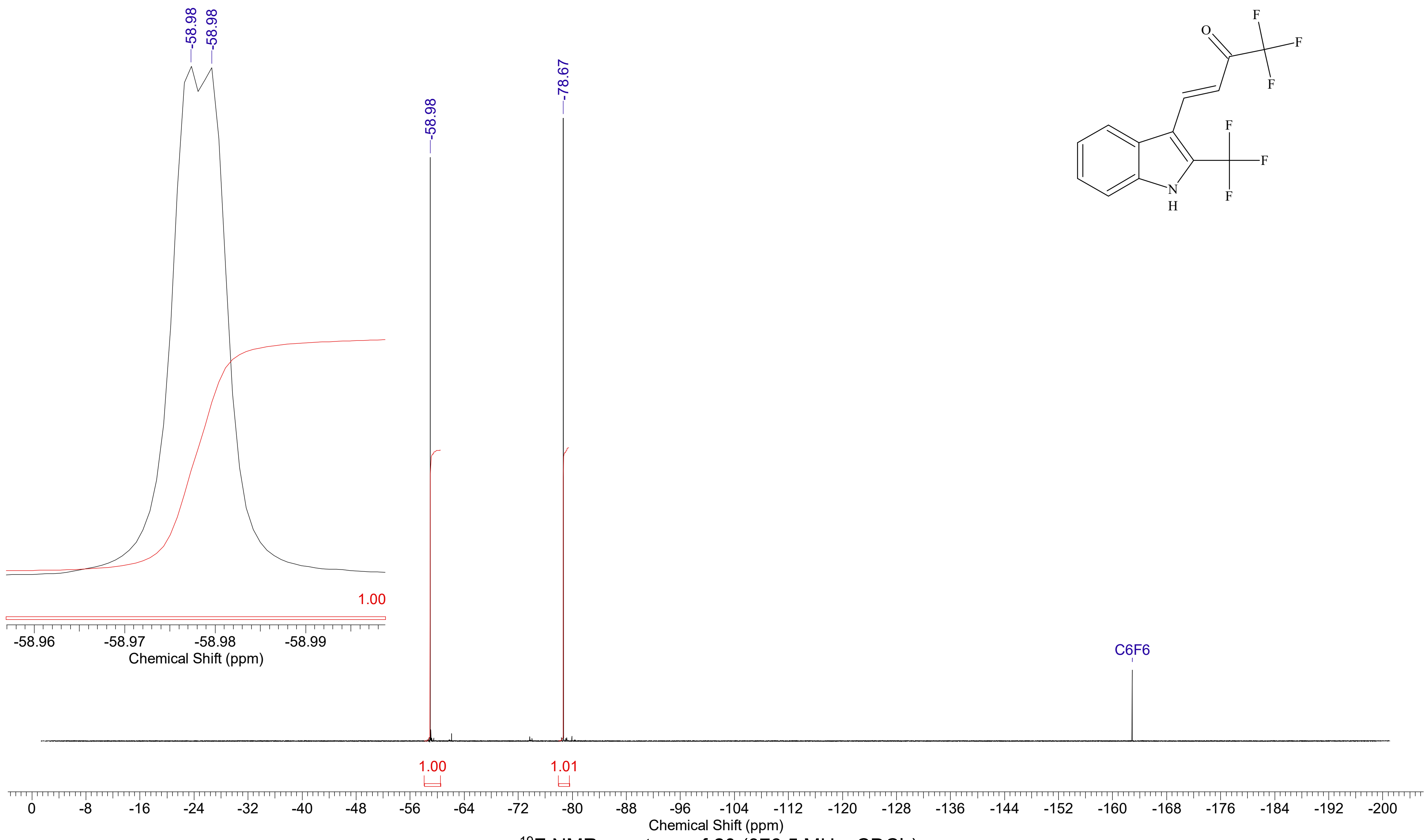

^{19}F NMR spectrum of **20** (376.5 MHz, $CDCl_3$)

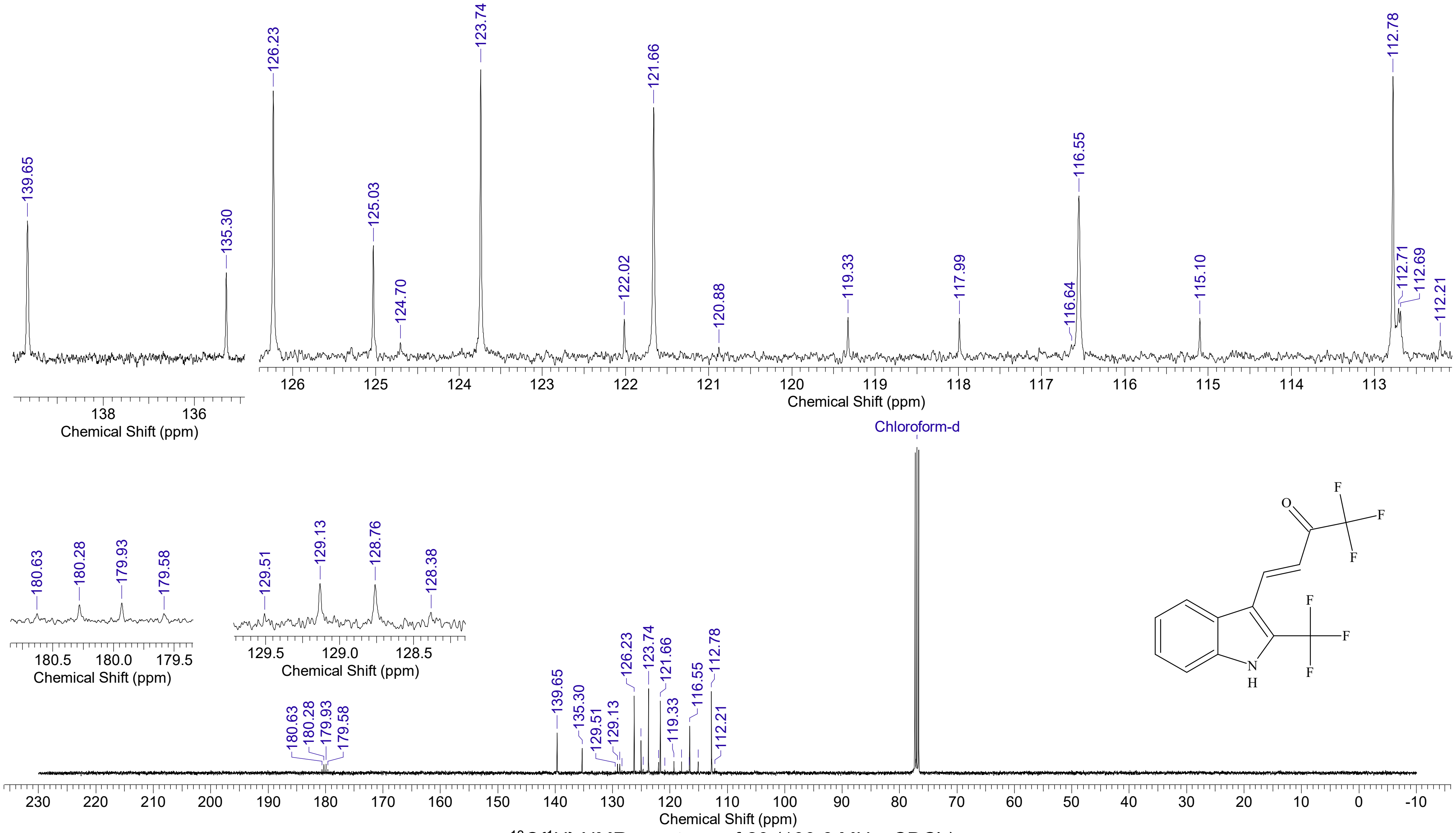

$^{13}C\{^{1}H\}$ NMR spectrum of **20** (100.6 MHz, $CDCl_3$)

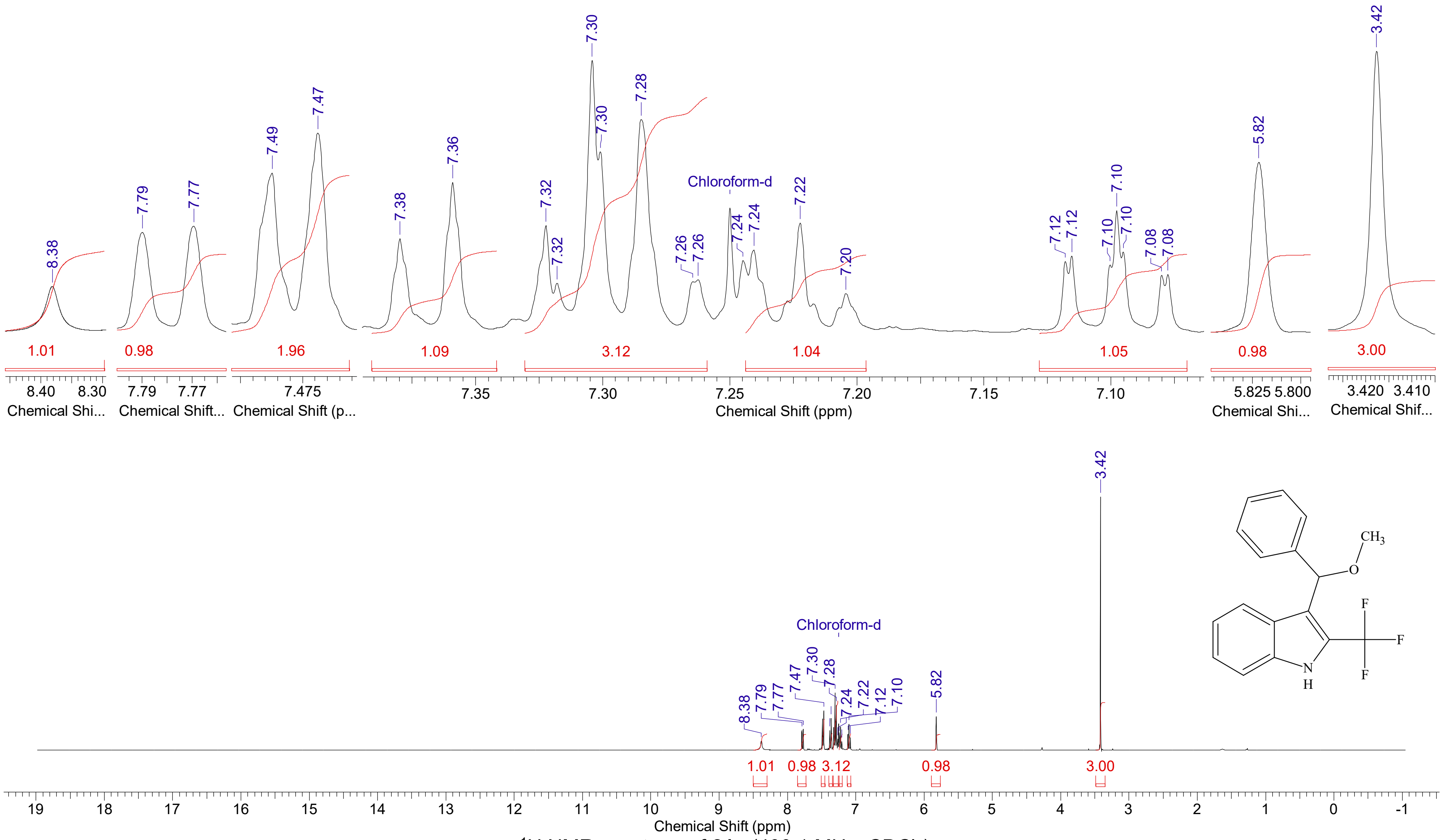

^{1}H NMR spectrum of **21a** (400.1 MHz, $CDCl_3$)

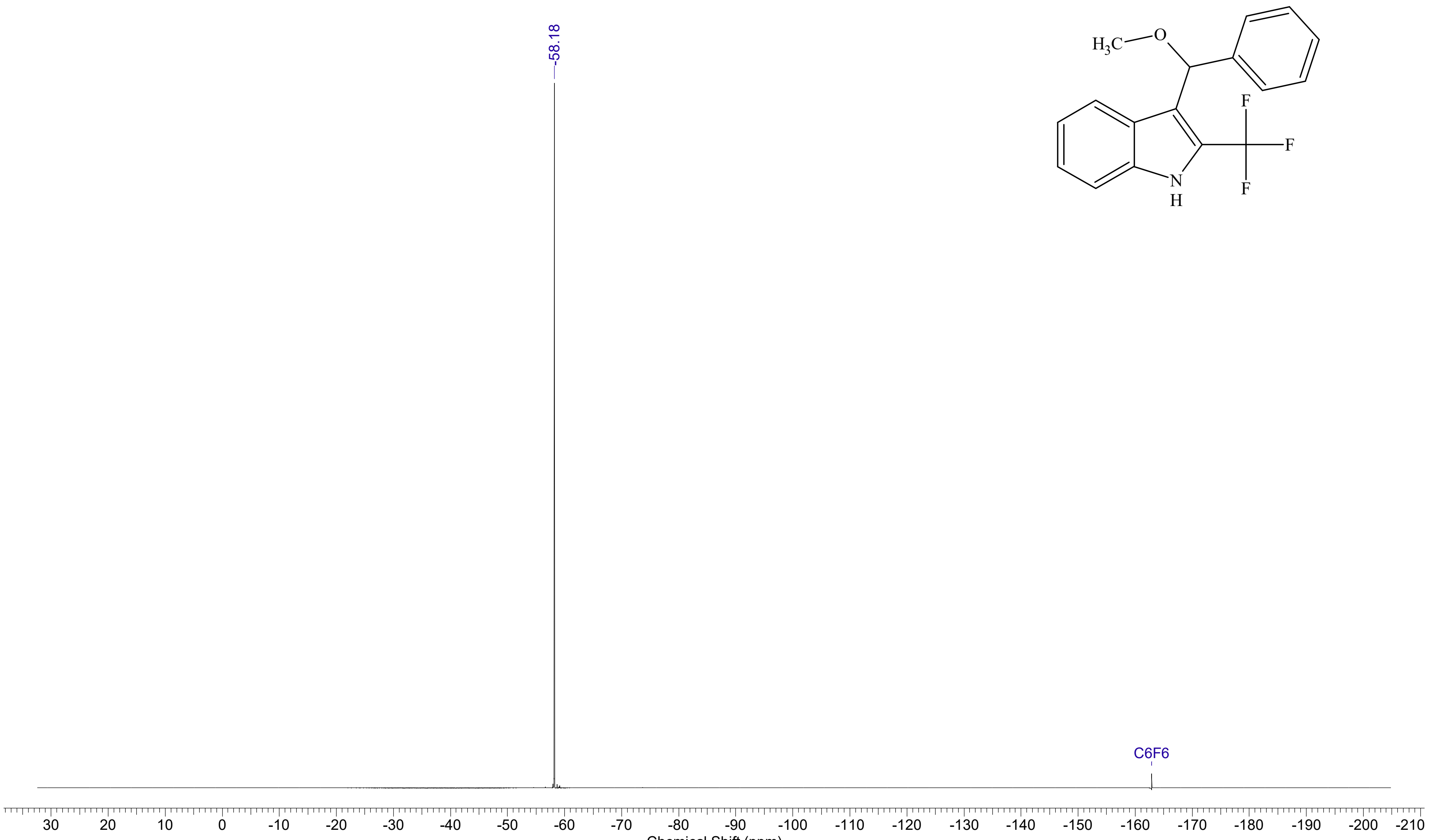

^{19}F NMR spectrum of **21a** (376.5 MHz, $CDCl_3$)

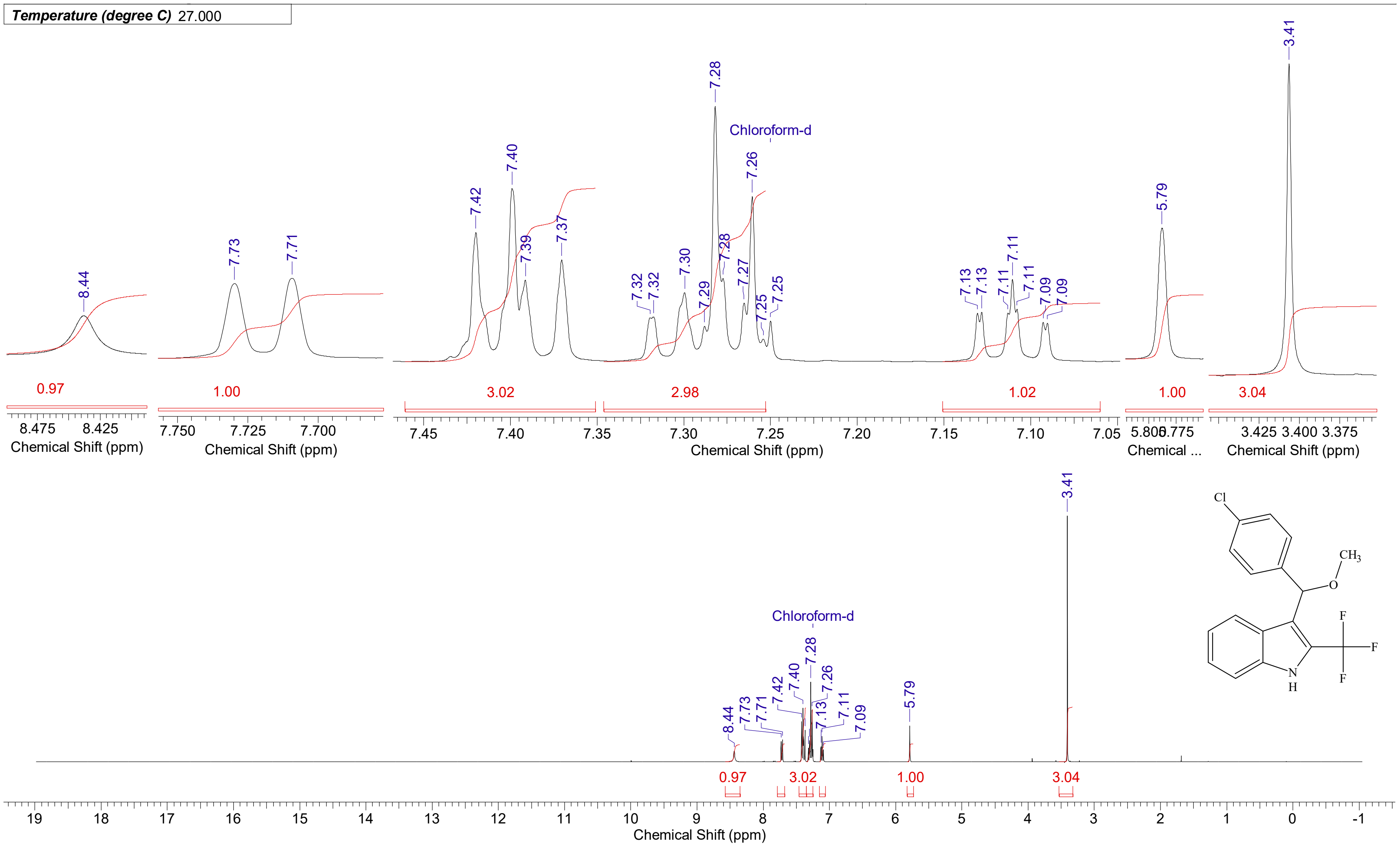

^{1}H NMR spectrum of **21b** (400.1 MHz, $CDCl_3$)

Temperature (degree C)	27.000

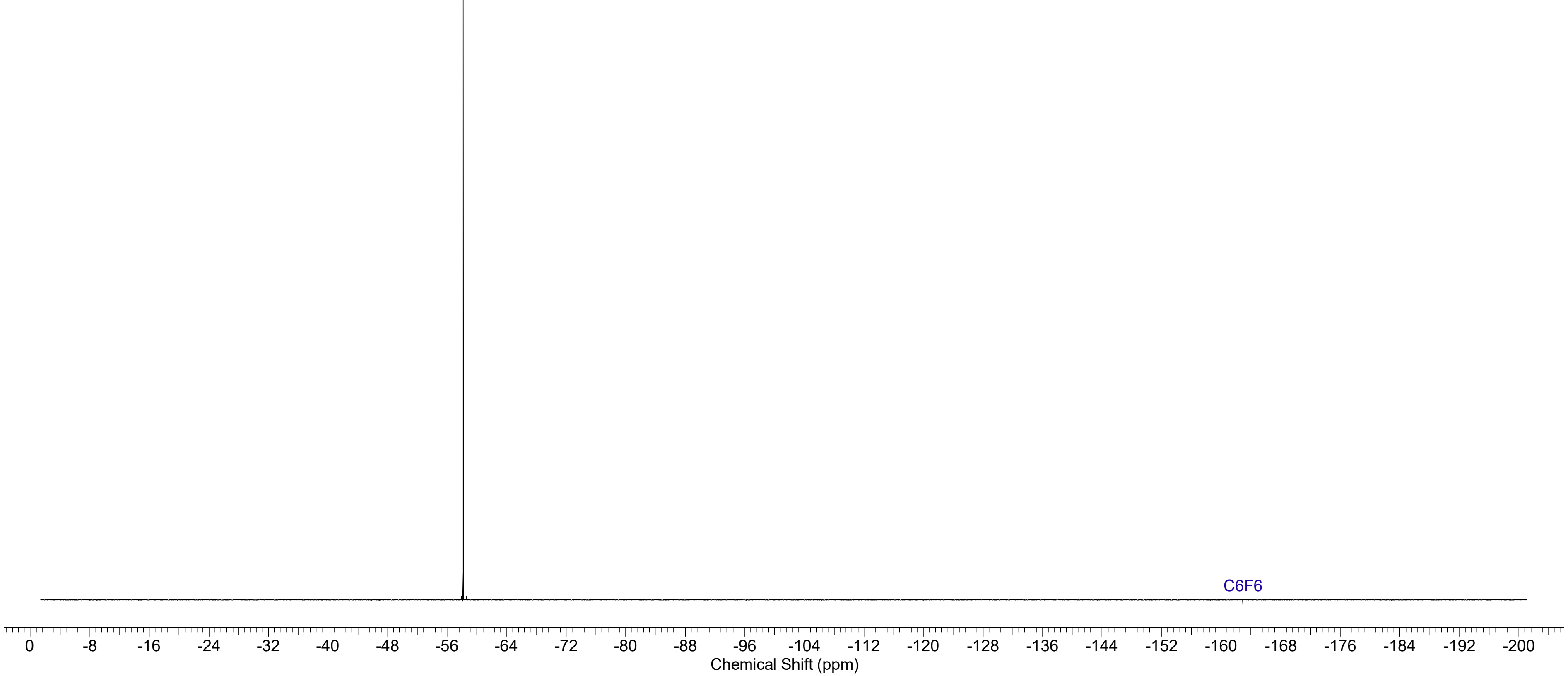

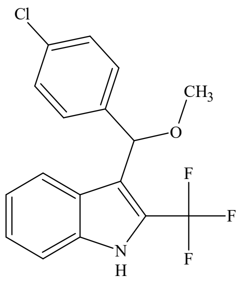

^{19}F NMR spectrum of **21b** (376.5 MHz, $CDCl_3$)

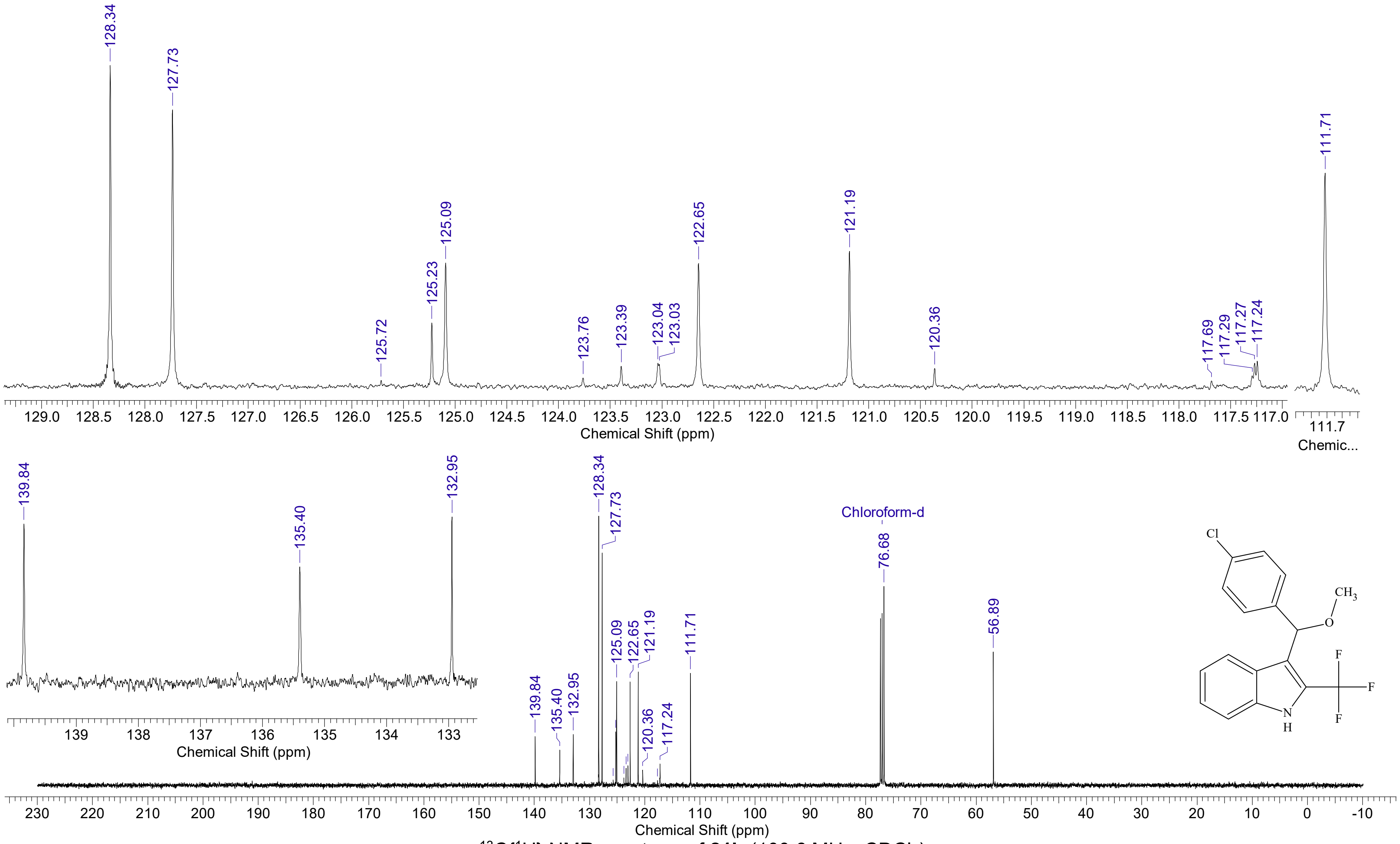

$^{13}C\{^1H\}$ NMR spectrum of **21b** (100.6 MHz, $CDCl_3$)

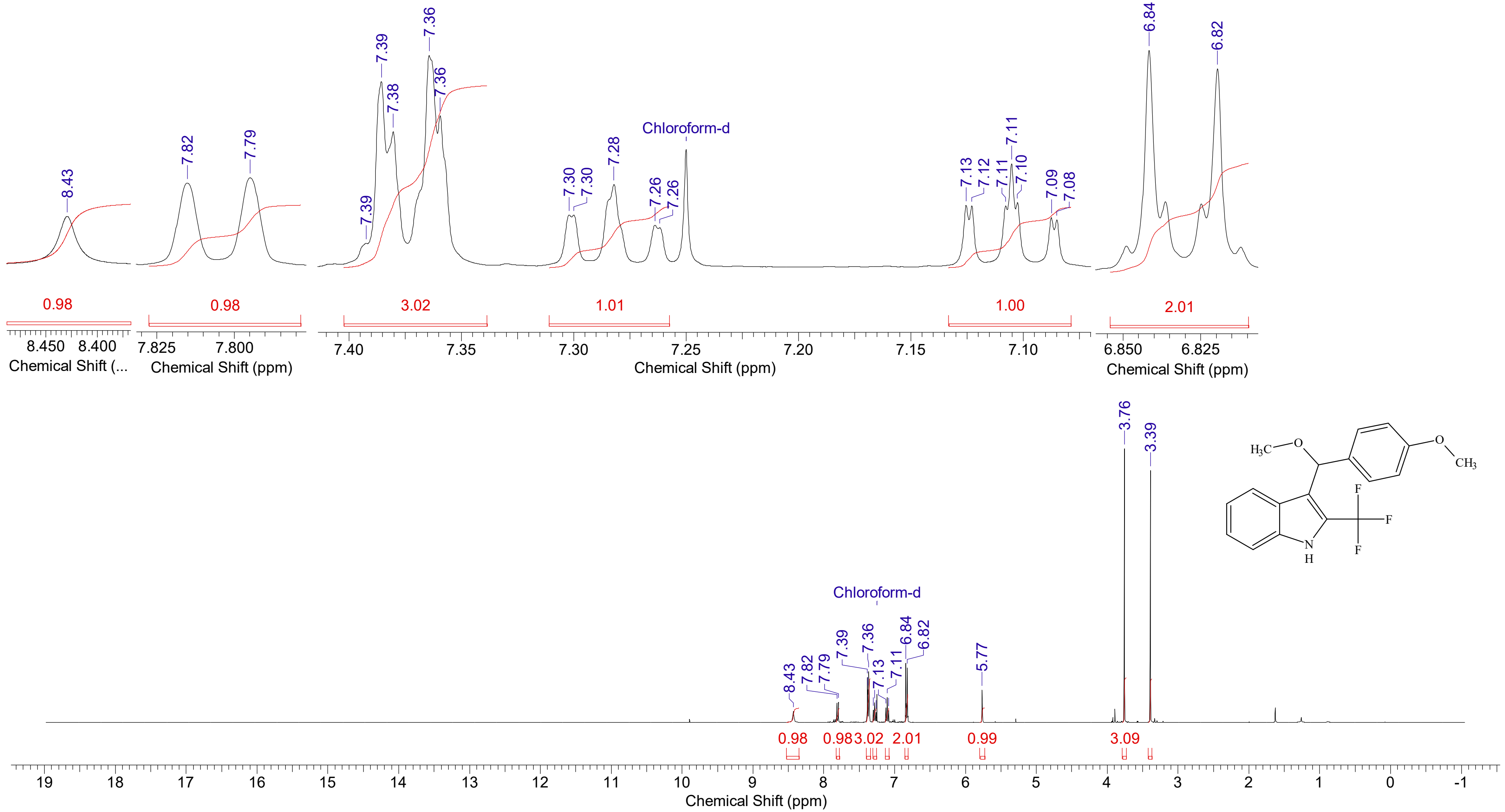

^{1}H NMR spectrum of **21c** (400.1 MHz, CDCl$_3$)

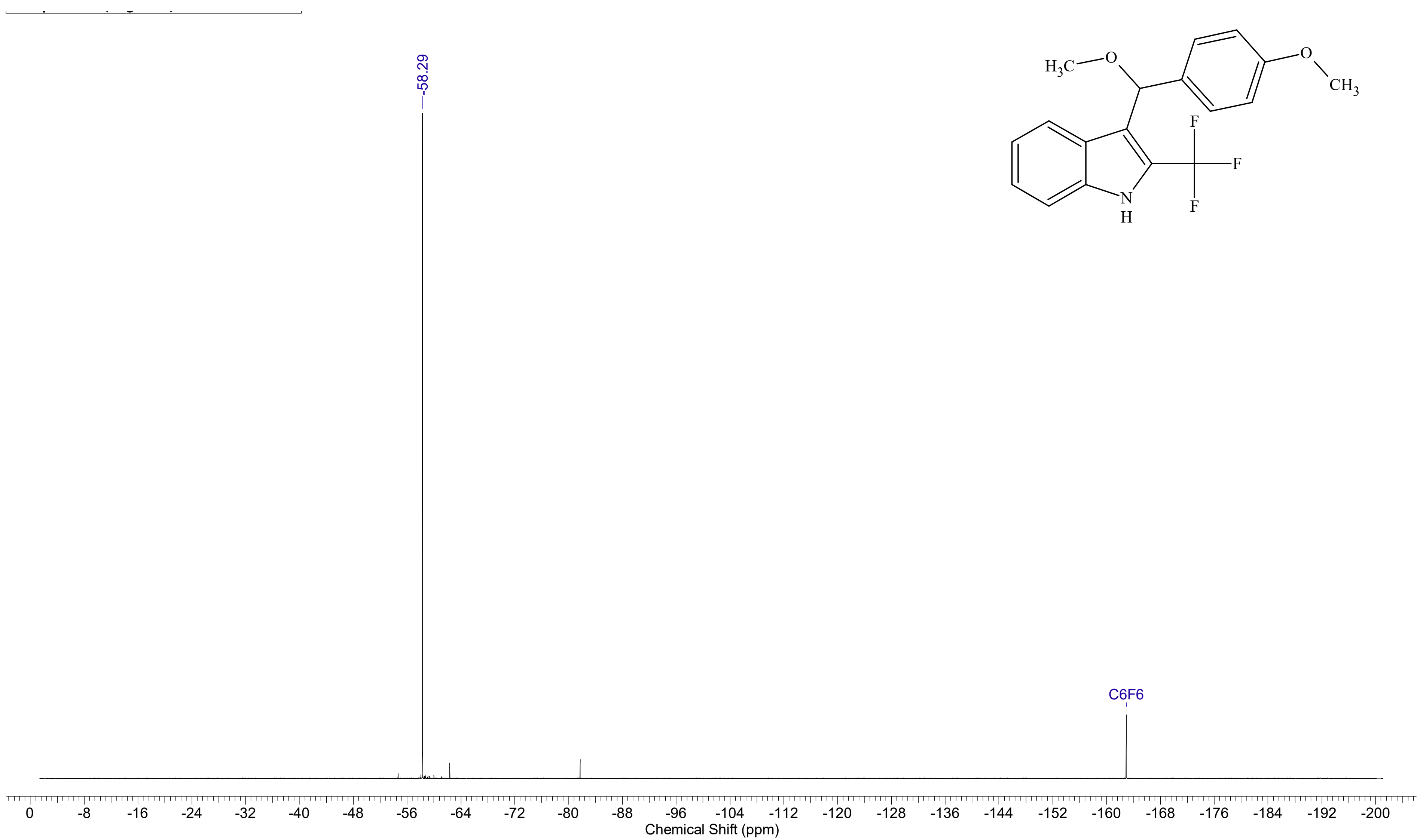

^{19}F NMR spectrum of **21c** (376.5 MHz, $CDCl_3$)

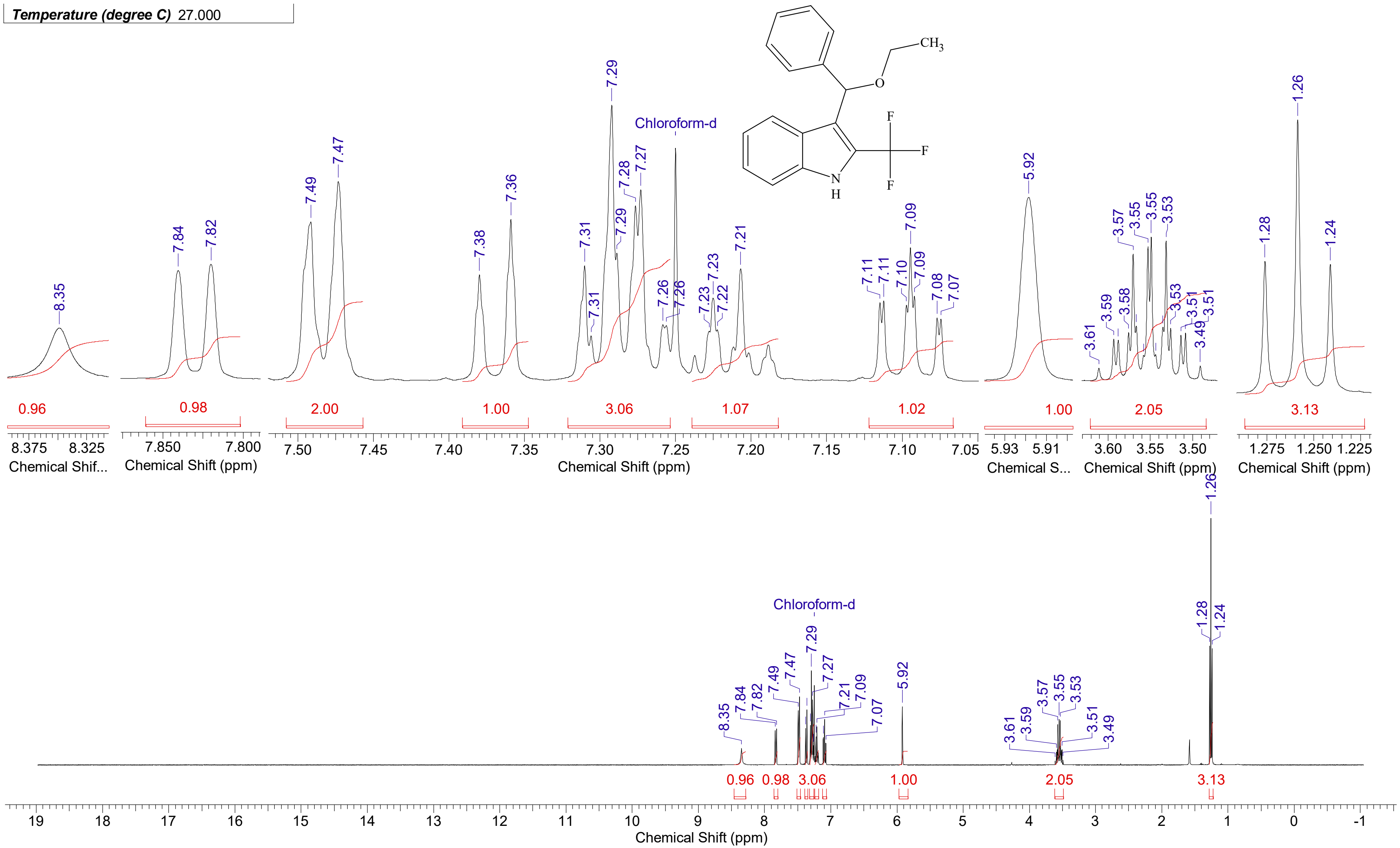

^{1}H NMR spectrum of **22** (400.1 MHz, $CDCl_3$)

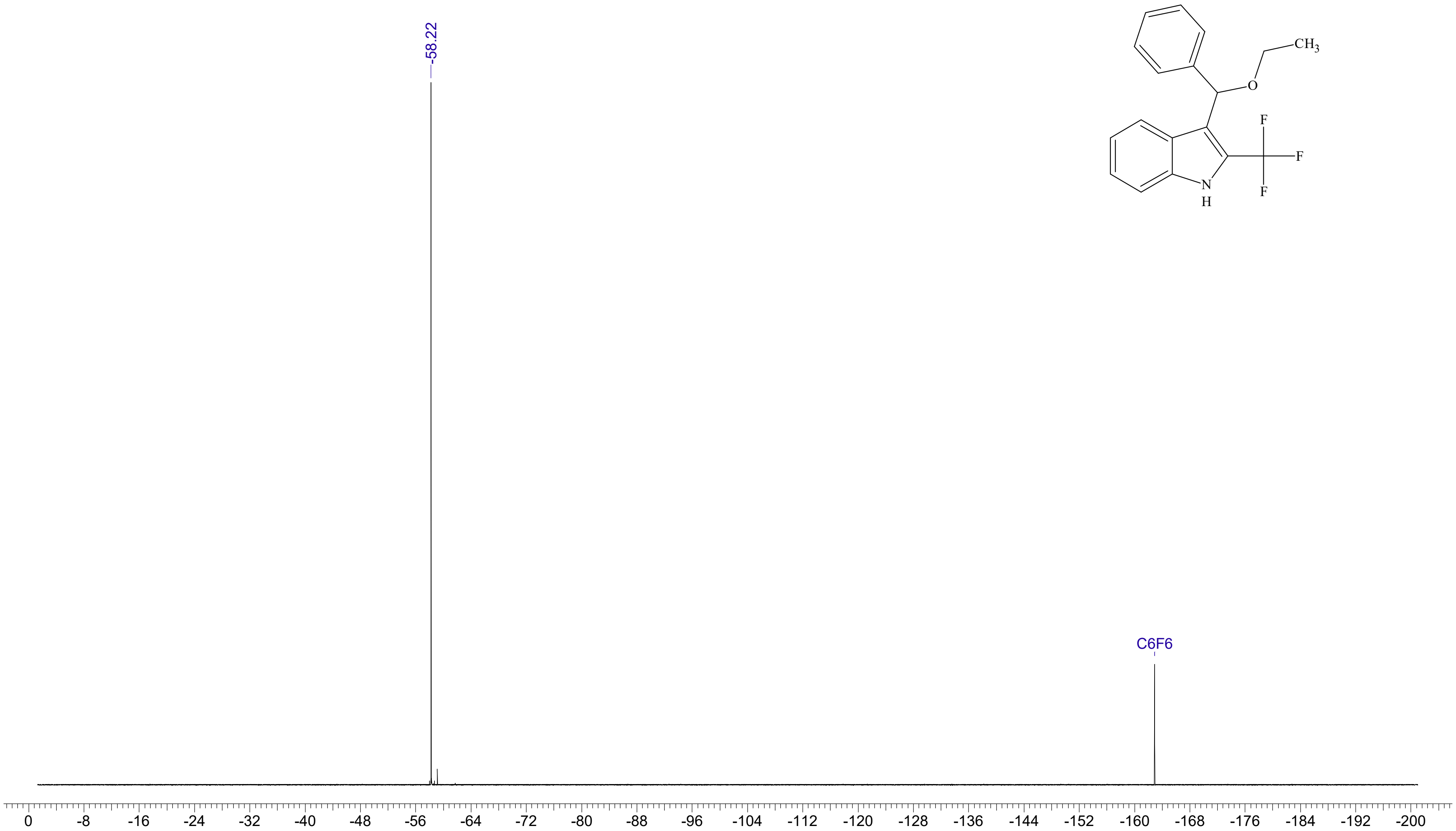

^{19}F NMR spectrum of **22** (376.5 MHz, $CDCl_3$)

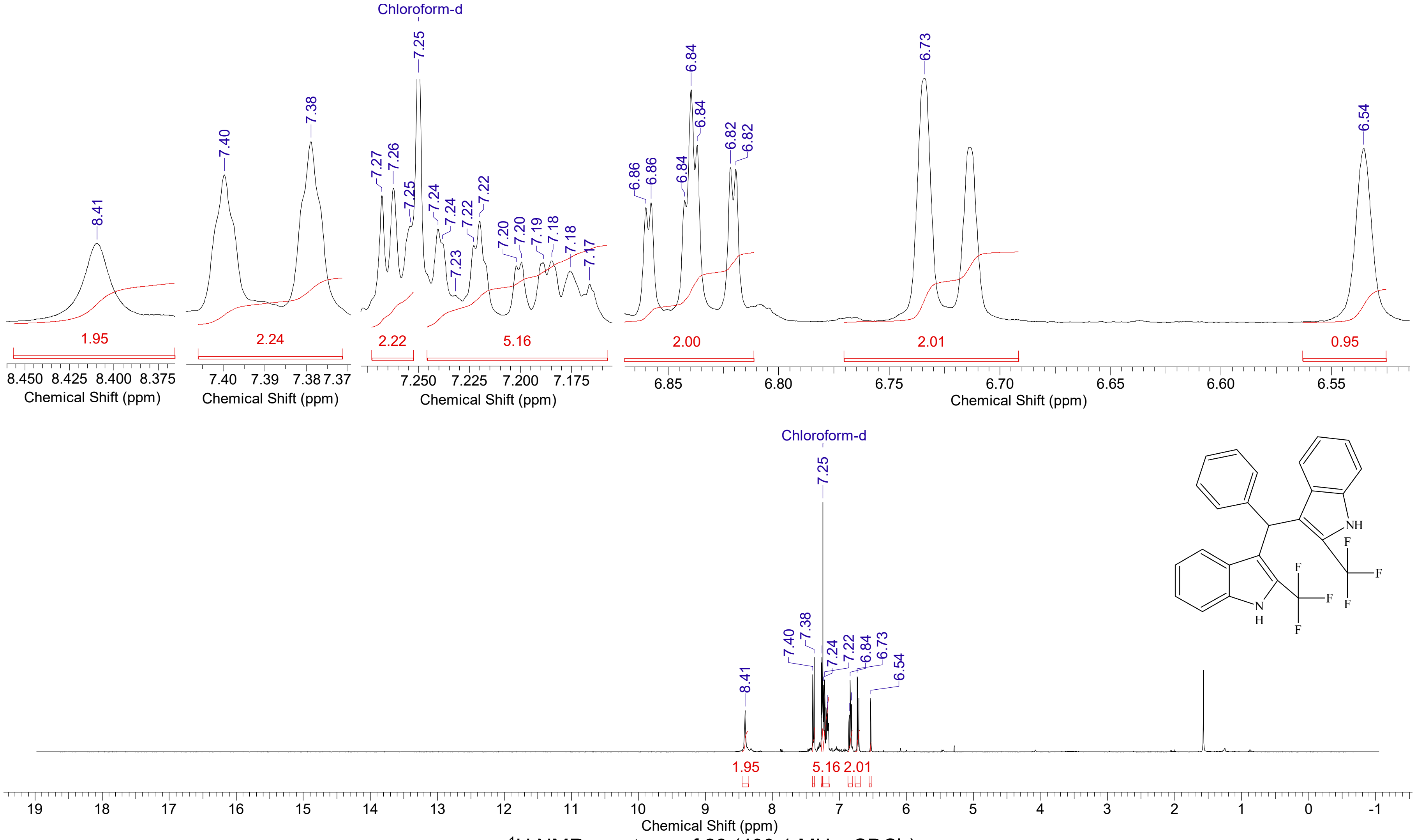

^{1}H NMR spectrum of **23** (400.1 MHz, $CDCl_3$)

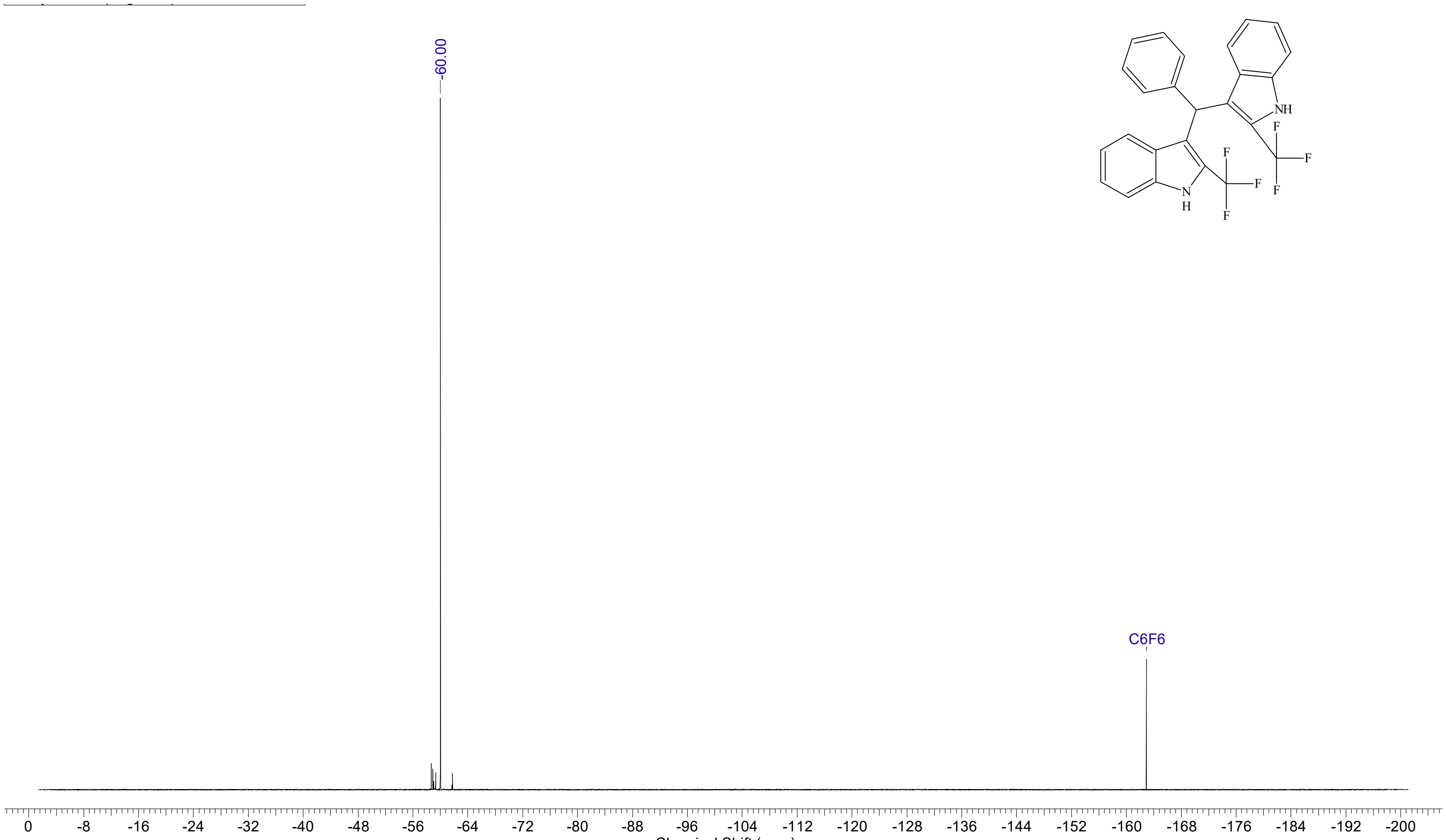

^{19}F NMR spectrum of **23** (376.5 MHz, $CDCl_3$)

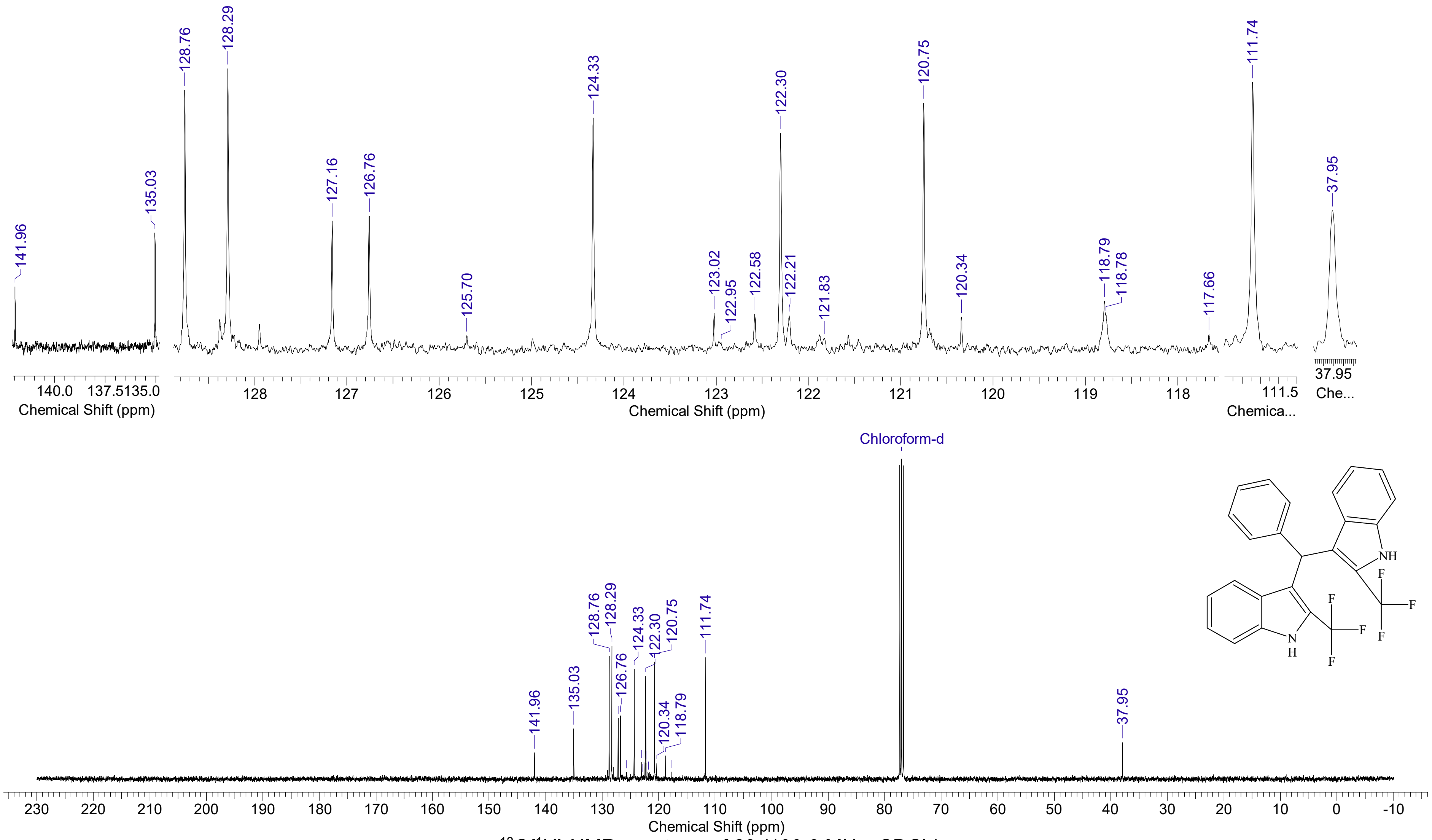

$^{13}C\{^{1}H\}$ NMR spectrum of **23** (100.6 MHz, $CDCl_3$)

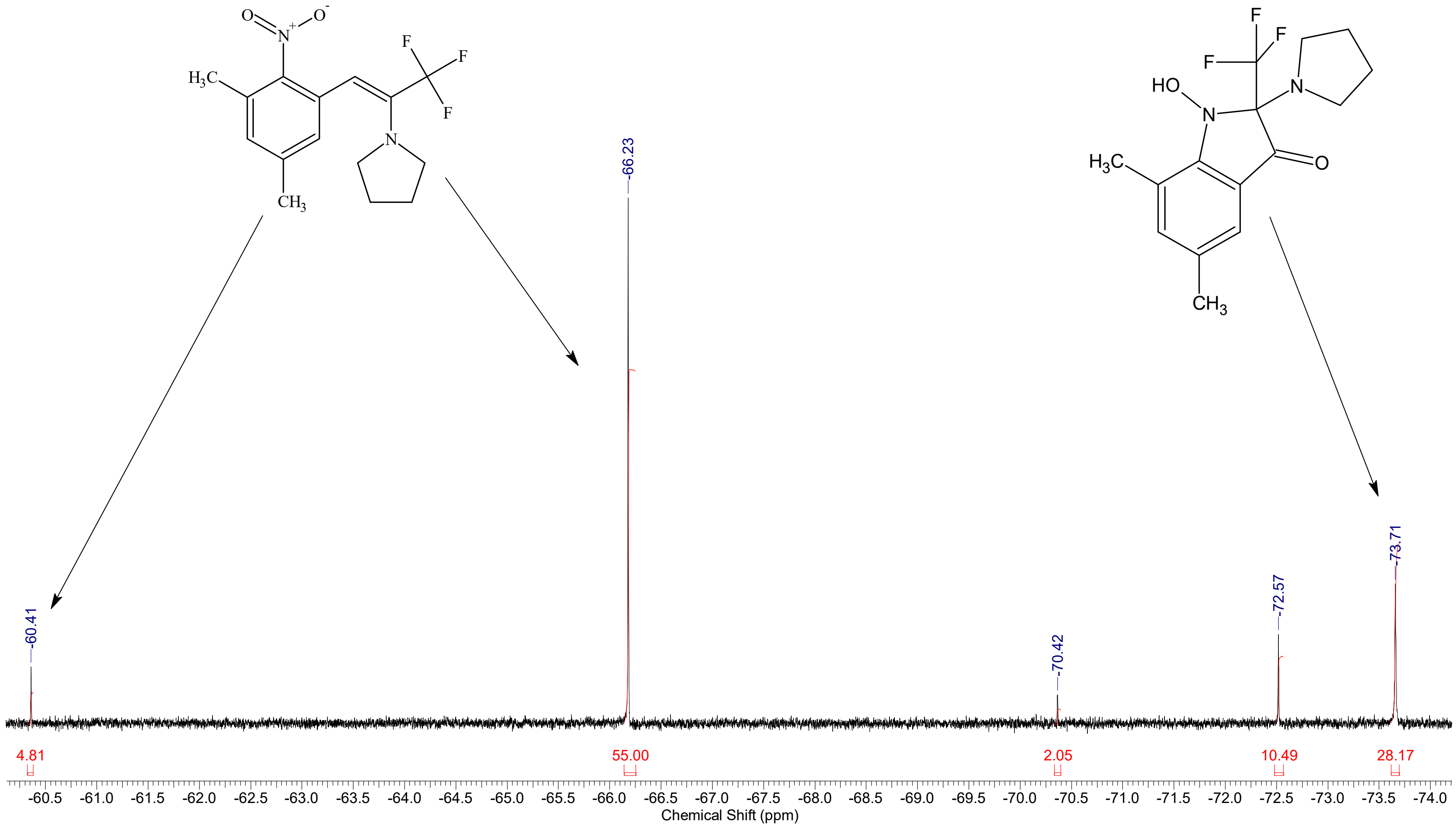

^{19}F NMR spectrum of the mixture of **3e** and by product **5e** (376.5 MHz, $CDCl_3$)

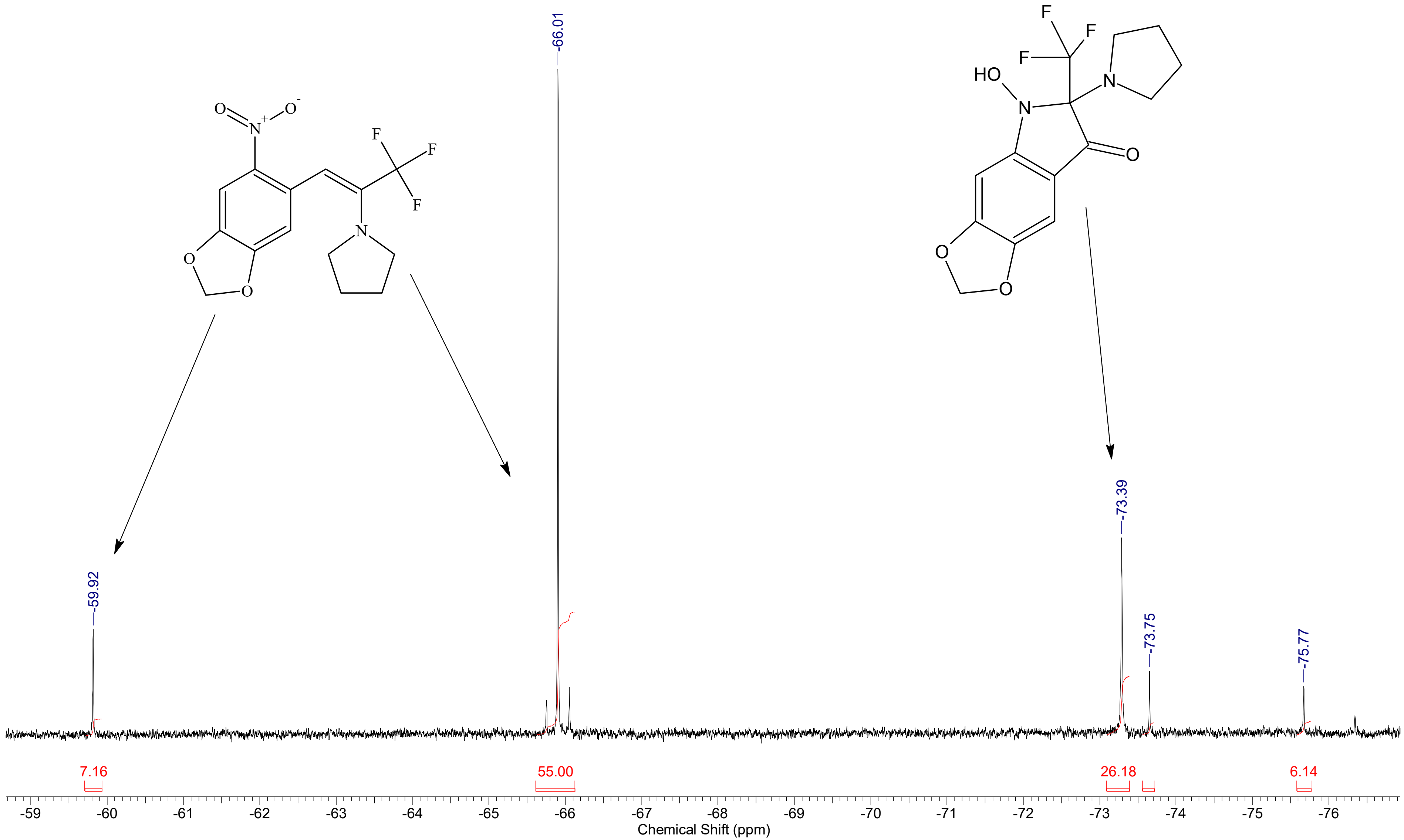

^{19}F NMR spectrum of the mixture of **3i** and by product **5i** (376.5 MHz, $CDCl_3$)

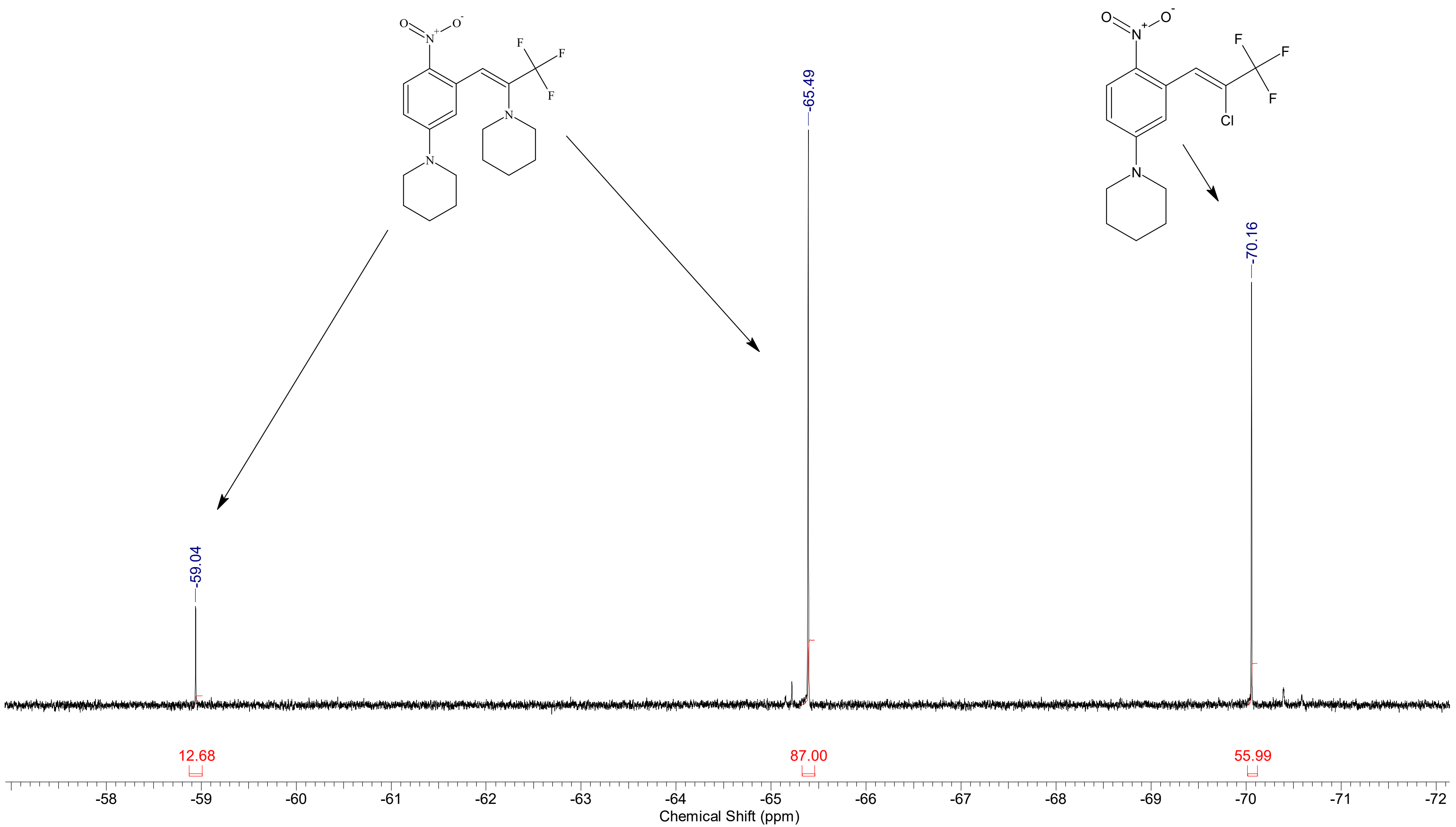

^{19}F NMR spectrum of the mixture of intermediates **11a** and **2p** (376.5 MHz, $CDCl_3$)

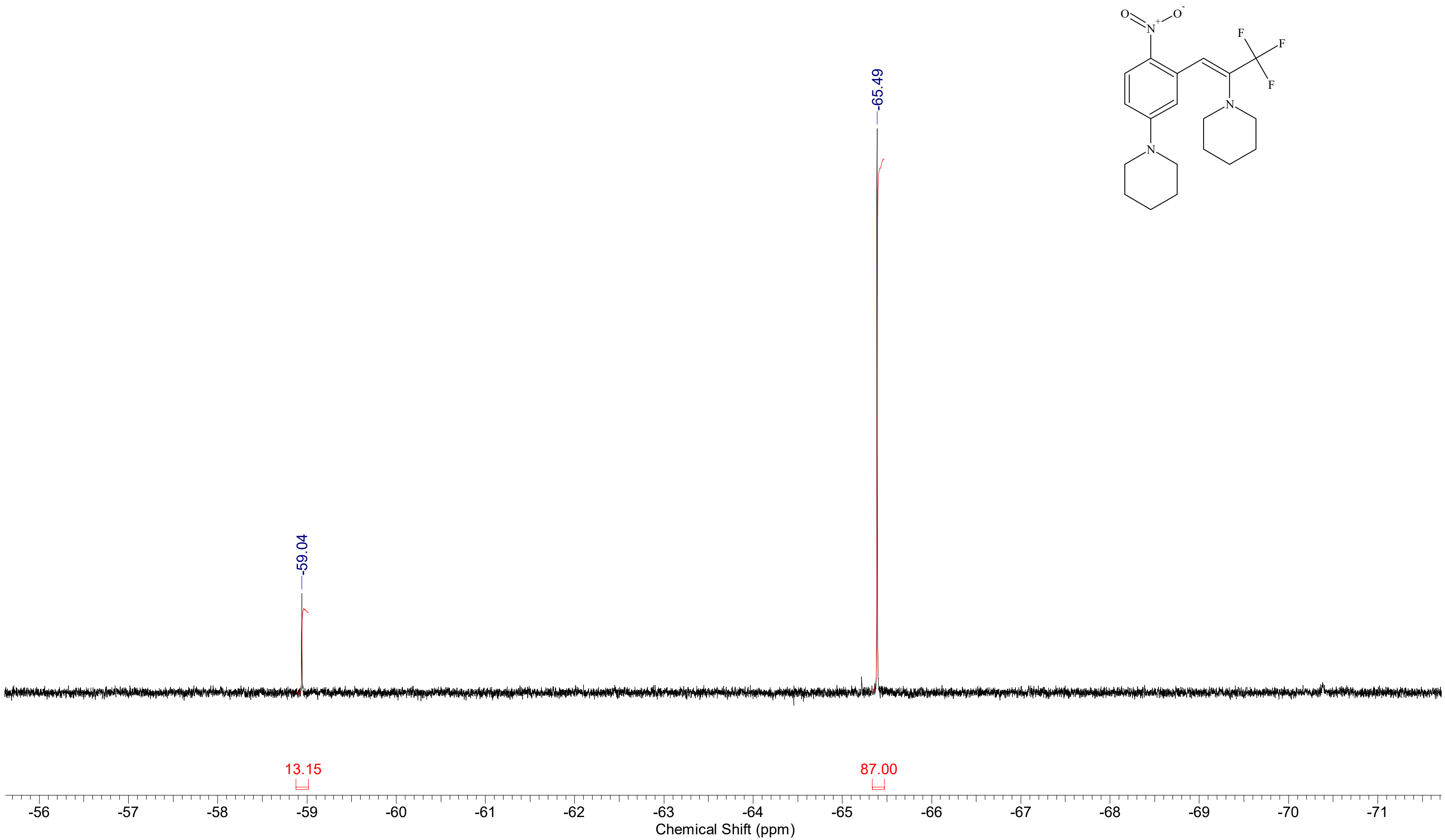

^{19}F NMR spectrum of intermediate **11a** (376.5 MHz, $CDCl_3$)

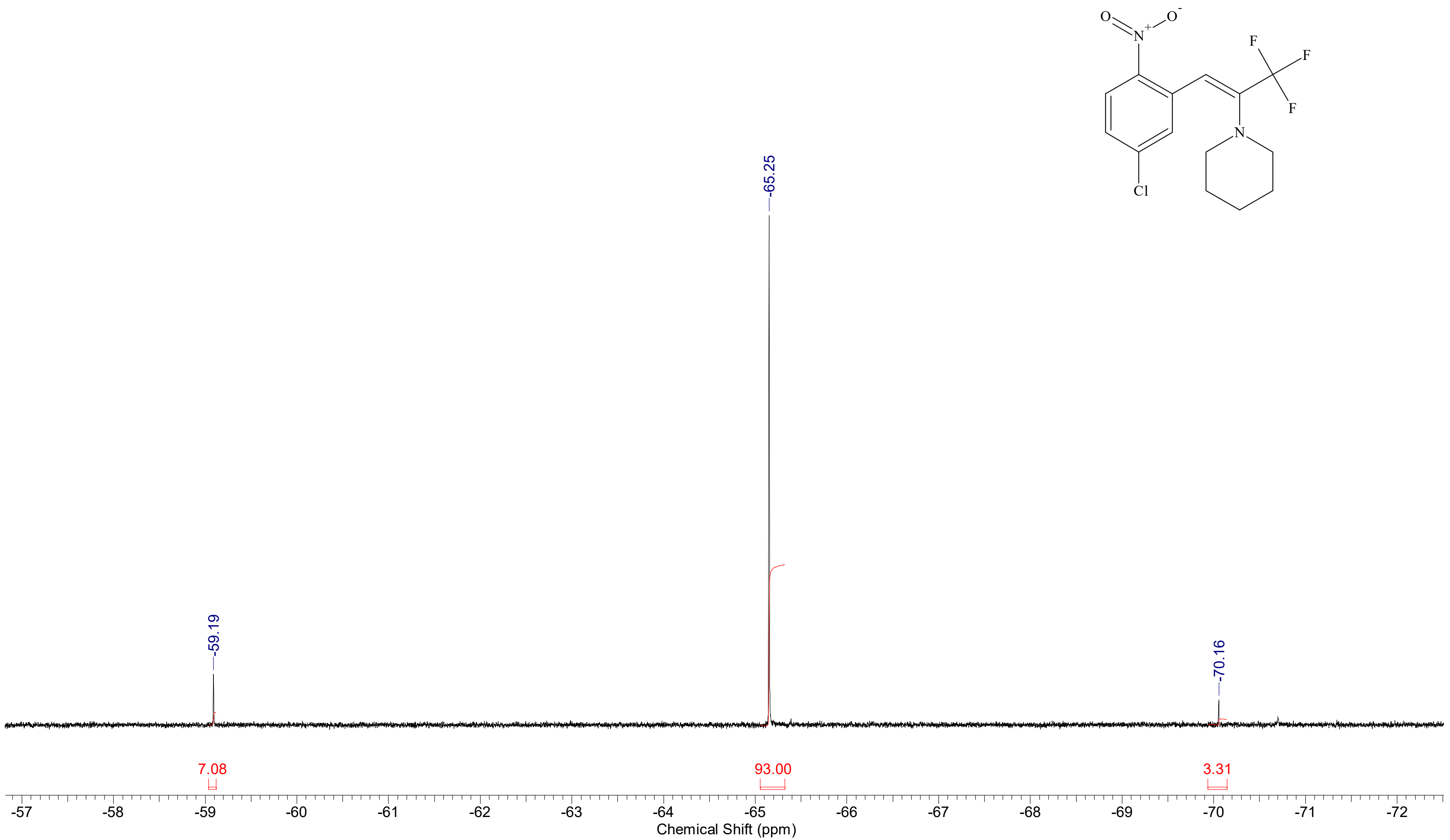

^{19}F NMR spectrum of intermediate **11b** (376.5 MHz, $CDCl_3$)

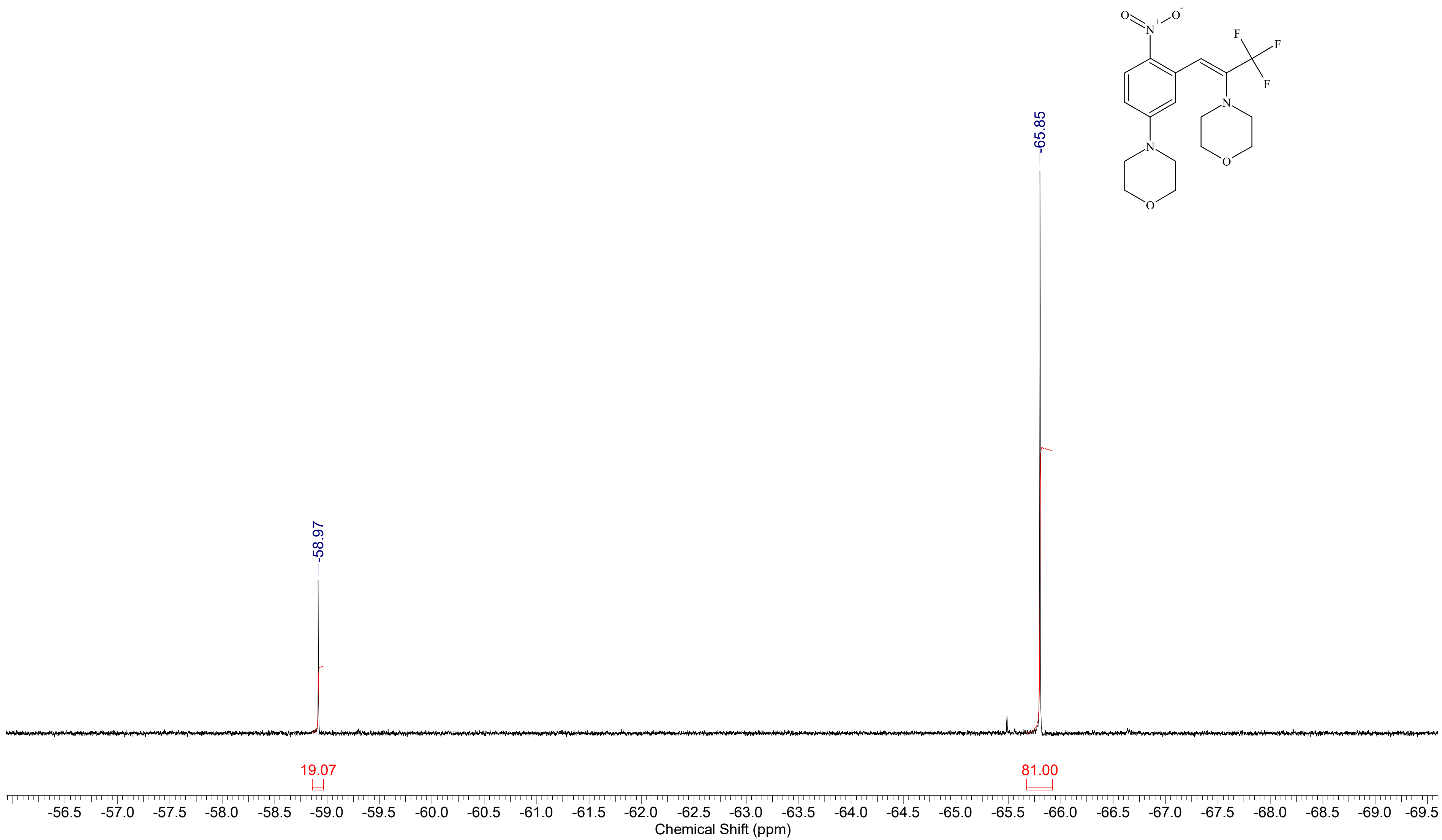

^{19}F NMR spectrum of intermediate **12** (376.5 MHz, $CDCl_3$)

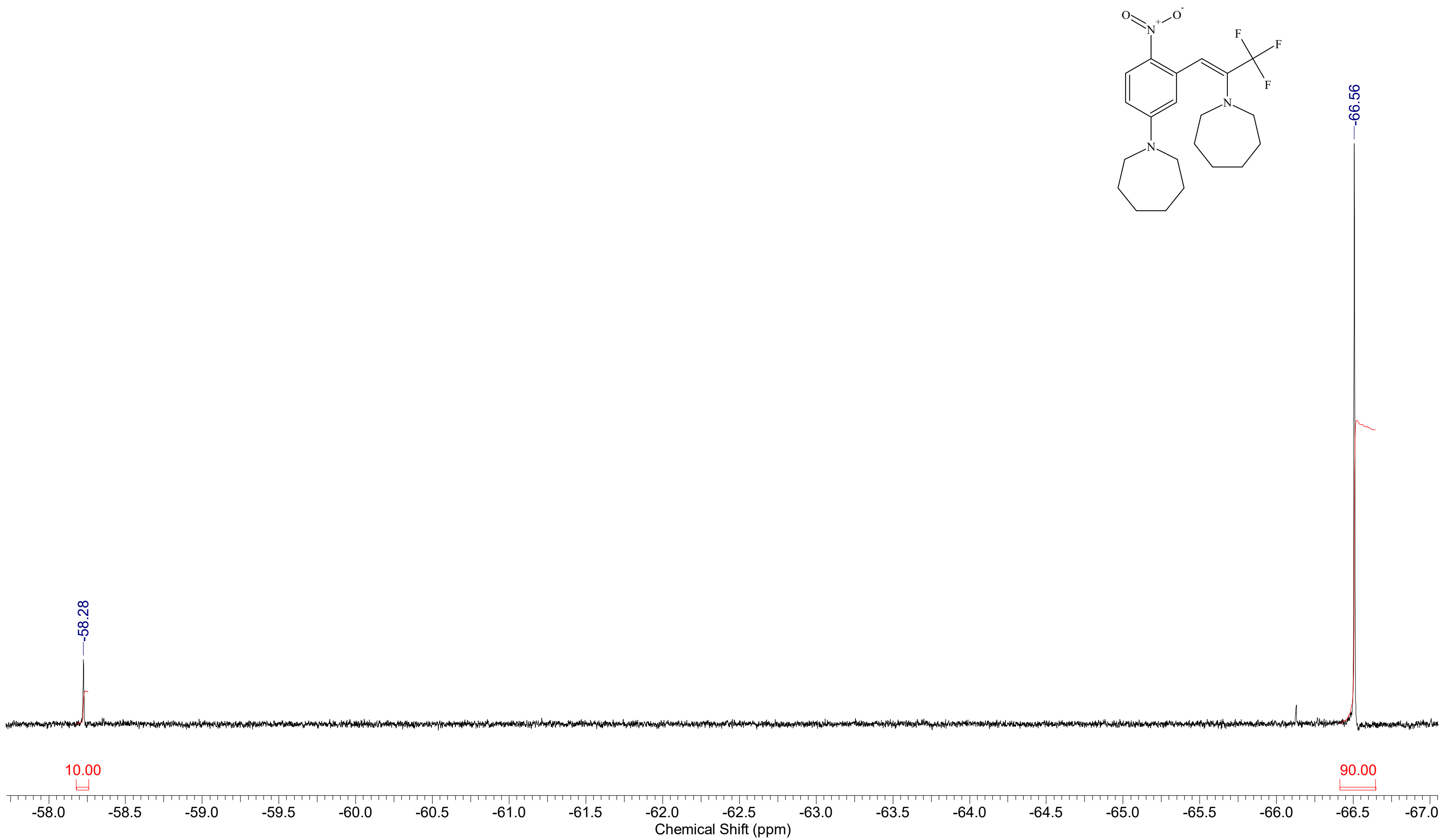

^{19}F NMR spectrum of intermediate **13** (376.5 MHz, $CDCl_3$)

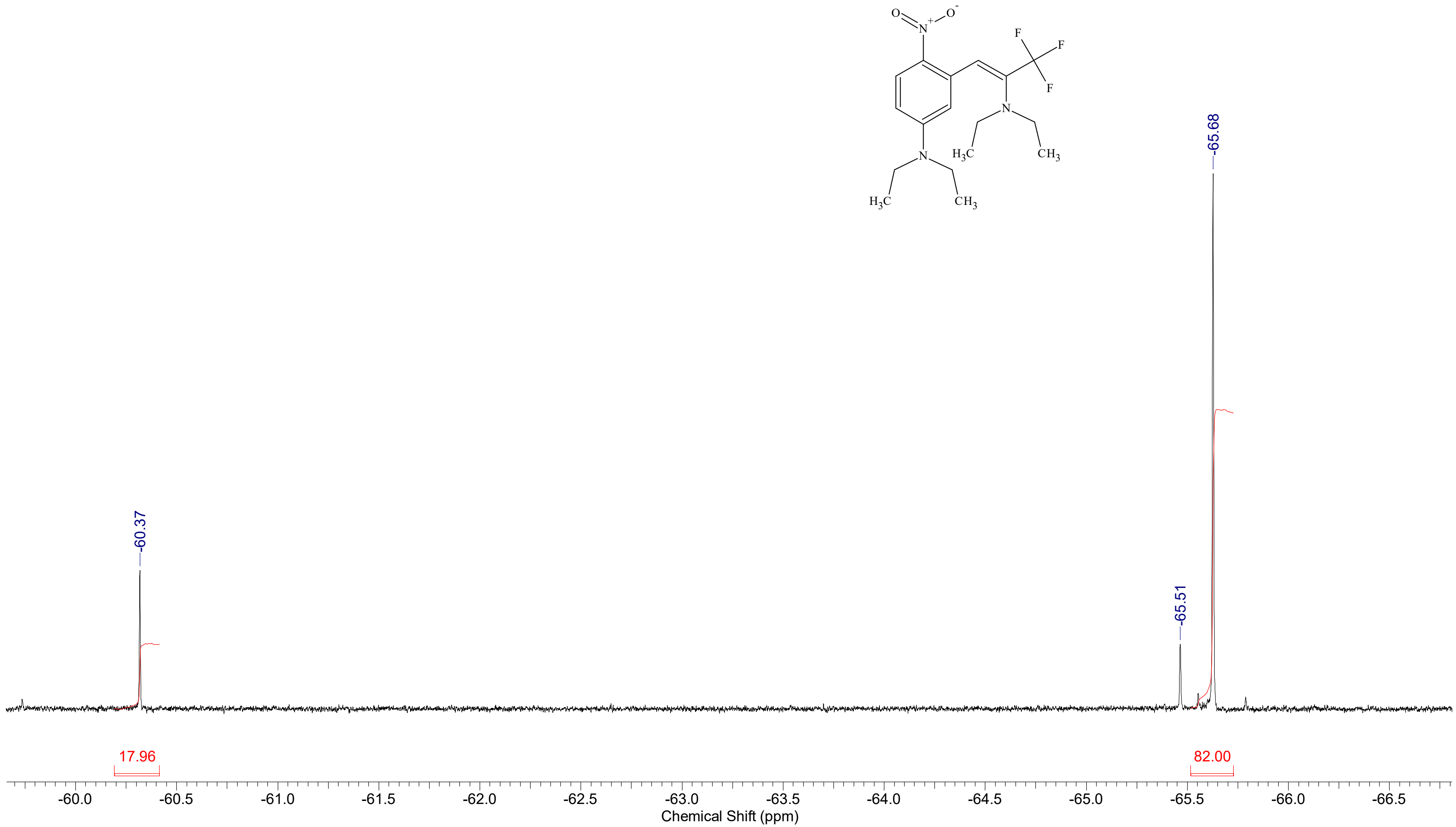

^{19}F NMR spectrum of intermediate **14** (376.5 MHz, $CDCl_3$)

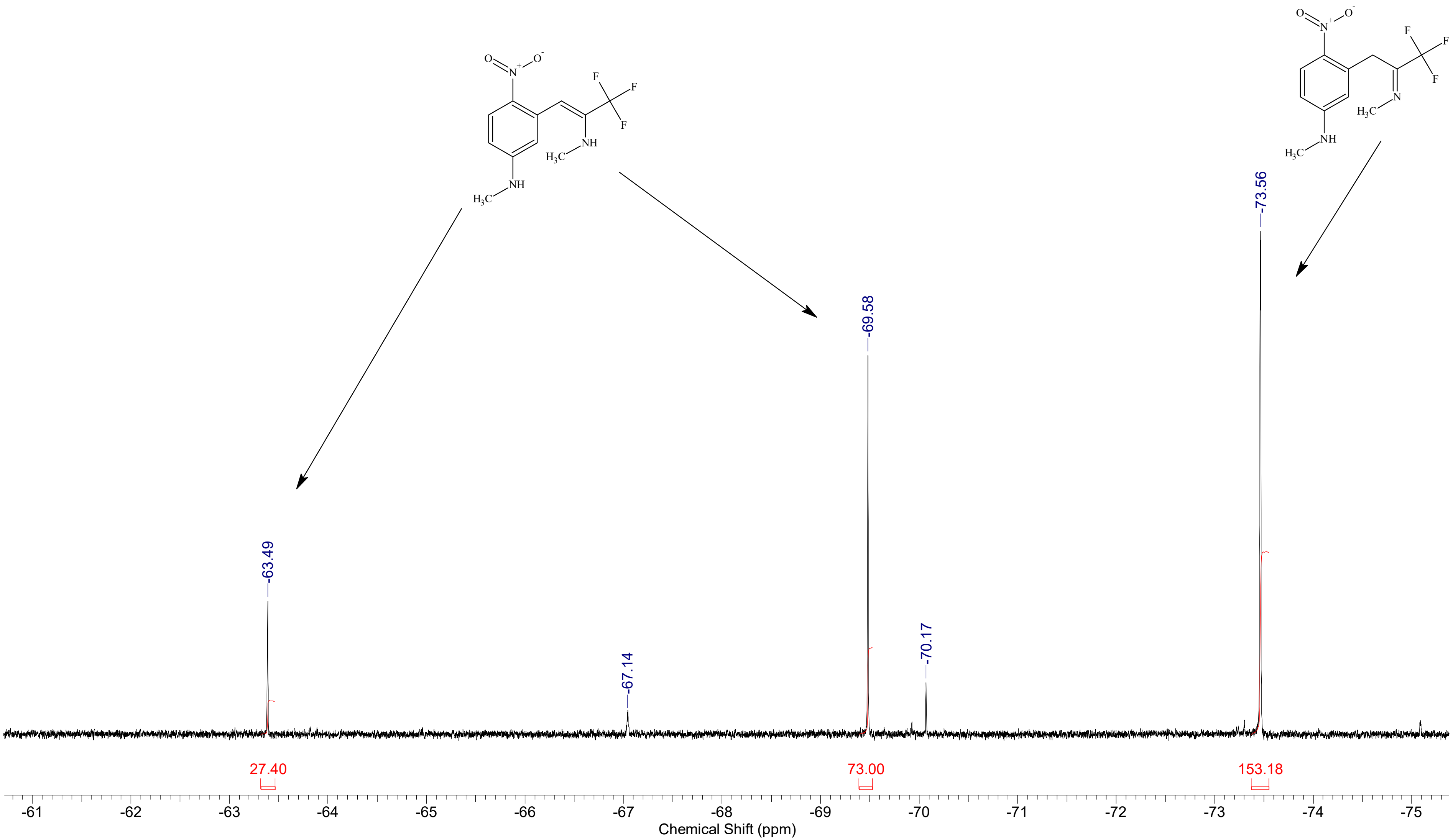

^{19}F NMR spectrum of intermediate **15** (376.5 MHz, $CDCl_3$)

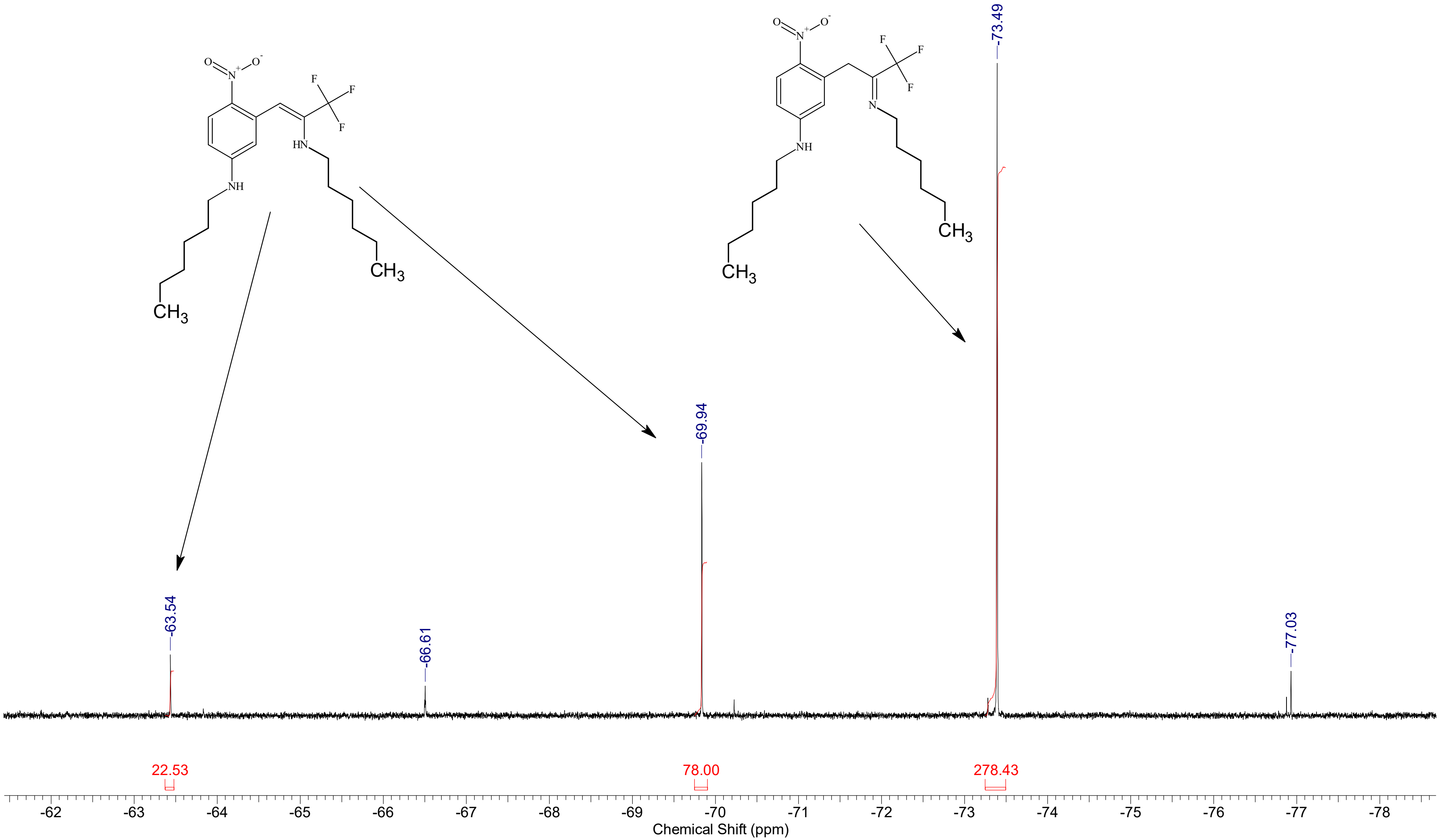

^{19}F NMR spectrum of intermediate **16** (376.5 MHz, $CDCl_3$)
